# Chiral Iodotriptycenes: Synthesis and Catalytic Applications

**DOI:** 10.1002/open.202200145

**Published:** 2022-07-13

**Authors:** Nasim Khan, Katsunori Itaya, Thomas Wirth

**Affiliations:** ^1^ School of Chemistry Cardiff University Park Place, Main Building CF10 3AT Cardiff UK

**Keywords:** iodine(III) reagents, ligand synthesis, oxidation, stereoselective synthesis, triptycenes

## Abstract

New iodotriptycenes, including some chiral derivatives, have been synthesised, and their catalytic potential towards oxidative transformations has been investigated. The enantioselectivities observed in the products using chiral iodotriptycene catalysts are low, probably owing to the large distances between the coordinating groups and the iodine moieties in these compounds.

## Graphical abstract

**Novel triptycene‐based organoiodine compounds** have been synthesised, including chiral iodotriptycenes. Their catalytic potential has been investigated in stereoselective oxidative transformations, where only low enantioselectivities were observed.

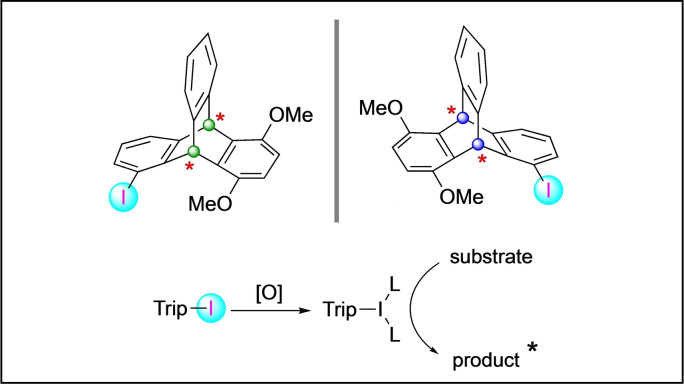

## Introduction

**Figure 1:**
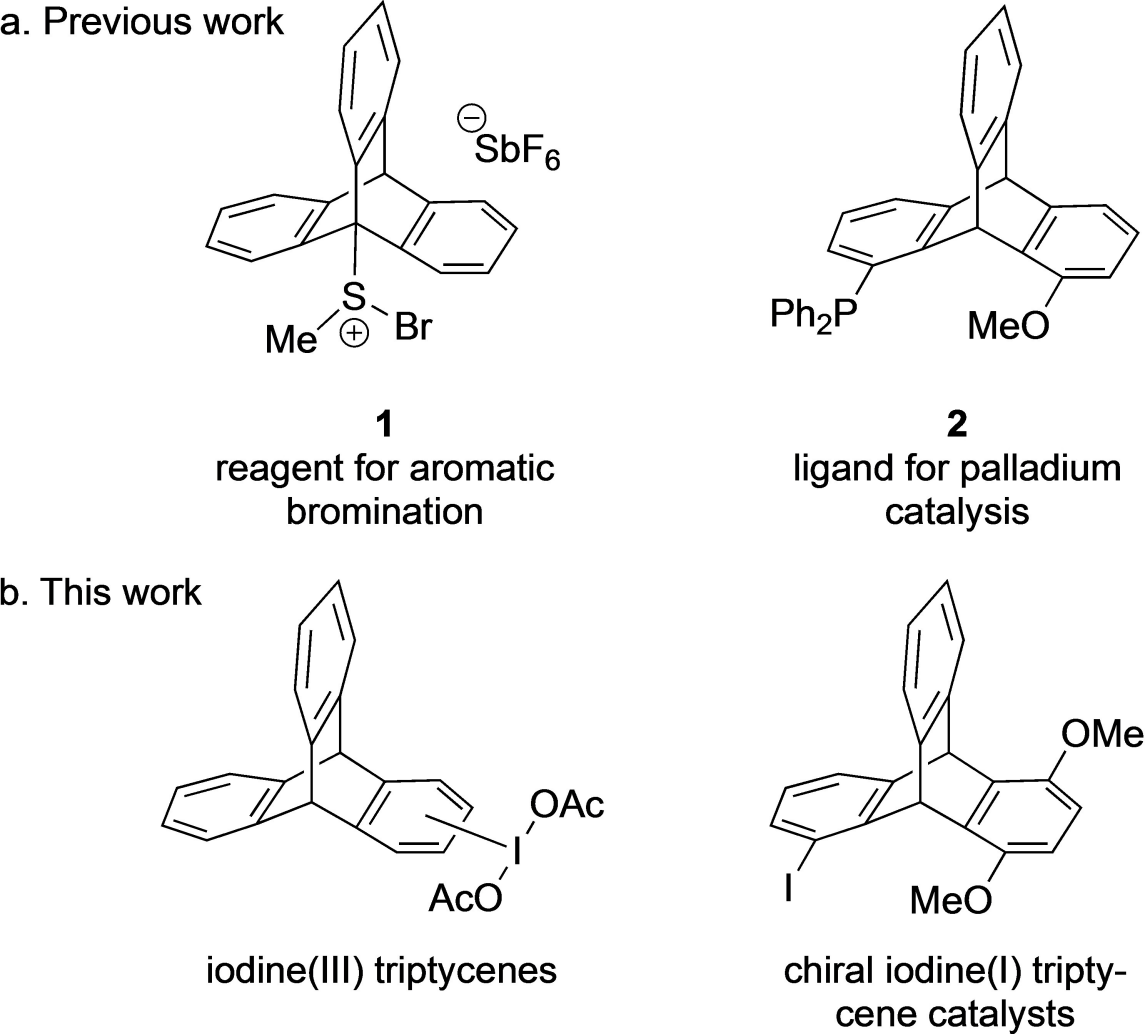
Previous and present work with triptycene catalysts and ligands.

Triptycenes have a bridged bicyclooctatriene core structure and are the simplest members of the iptycene family. These rigid molecules with *D*_3*h*_ symmetry provides a large free volume around the three aromatic rings which are at an angle of 120°.^[1]^ Mainly achiral triptycenes have been extensively studied in different areas such as polymer chemistry, materials chemistry, molecular machines, nanosized molecular cages, molecular balances, medicinal chemistry, peptide chemistry, molecular assembly and host−guest chemistry.^[2]^ Triptycenes as structures for catalysts or reagents in organic transformations have not been explored intensively. Some work highlights the use of triptycenes as a ligand/catalyst incorporating metals, mostly pincer‐type ligands/complexes in organic transformations such as alkenes^[3]^ and 2‐methyl‐3‐butenenitrile isomerization,^[4]^ chemoselective transfer‐hydrogenation of α,β‐unsaturated ketones,^[5]^ transfer dehydrogenations of alkanes,^[6]^ bis‐hydroformylation of butadiene,^[7]^ selective hydrocyanation of butadiene,^[8]^ cyanation of aryl bromides,^[9]^ and cross‐coupling reactions of aryl chlorides with phenylboronic acid.^[10]^ Very recently, triptycenyl methyl sulfide has been explored for electrophilic aromatic halogenations with *N*‐halosuccinimides through the formation of sulfonium salt **1** as the active species (Figure 1a).^[11]^ Metal‐free trifluoromethylthiolation of aromatic compounds has also been investigated.^[12]^ Mainly achiral triptycenes were explored in different areas of chemicals and material science, although the first synthesis of chiral triptycenes was reported in 1962.^[13]^ Very recently, reviews have been published on chiral triptycenes.^[14]^ Triptycene‐based ligands in enantiopure form have been prepared by lithiation of *rac*‐1‐bromo‐8‐diphenylphosphinotriptycene and subsequent quenching with enantiomerically pure (1*R*,2*S*,5*R*)‐(−)‐menthyl (*S*)‐*p*‐toluenesulfinate. The diastereomers were resolved using column chromatography and finally hydrogenated to P,S‐triptycene‐based chiral ligands.^[15a]^ Chiral ligand **2** has been applied in the palladium‐catalyzed asymmetric synthesis for the hydrosilylation of styrene (Figure 1a).^[15b]^ The rigid structure and the unique features of the triptycene scaffold for functionalisations attracted us to synthesise iodotriptycenes and chiral iodotriptycenes derivatives (Figure 1b). Hypervalent iodine reagents have attracted much attention in oxidative reactions^[16]^ and extensive efforts have been dedicated to the design and synthesis of chiral derivatives.^[17]^

## Results and Discussion

**Scheme 1:**
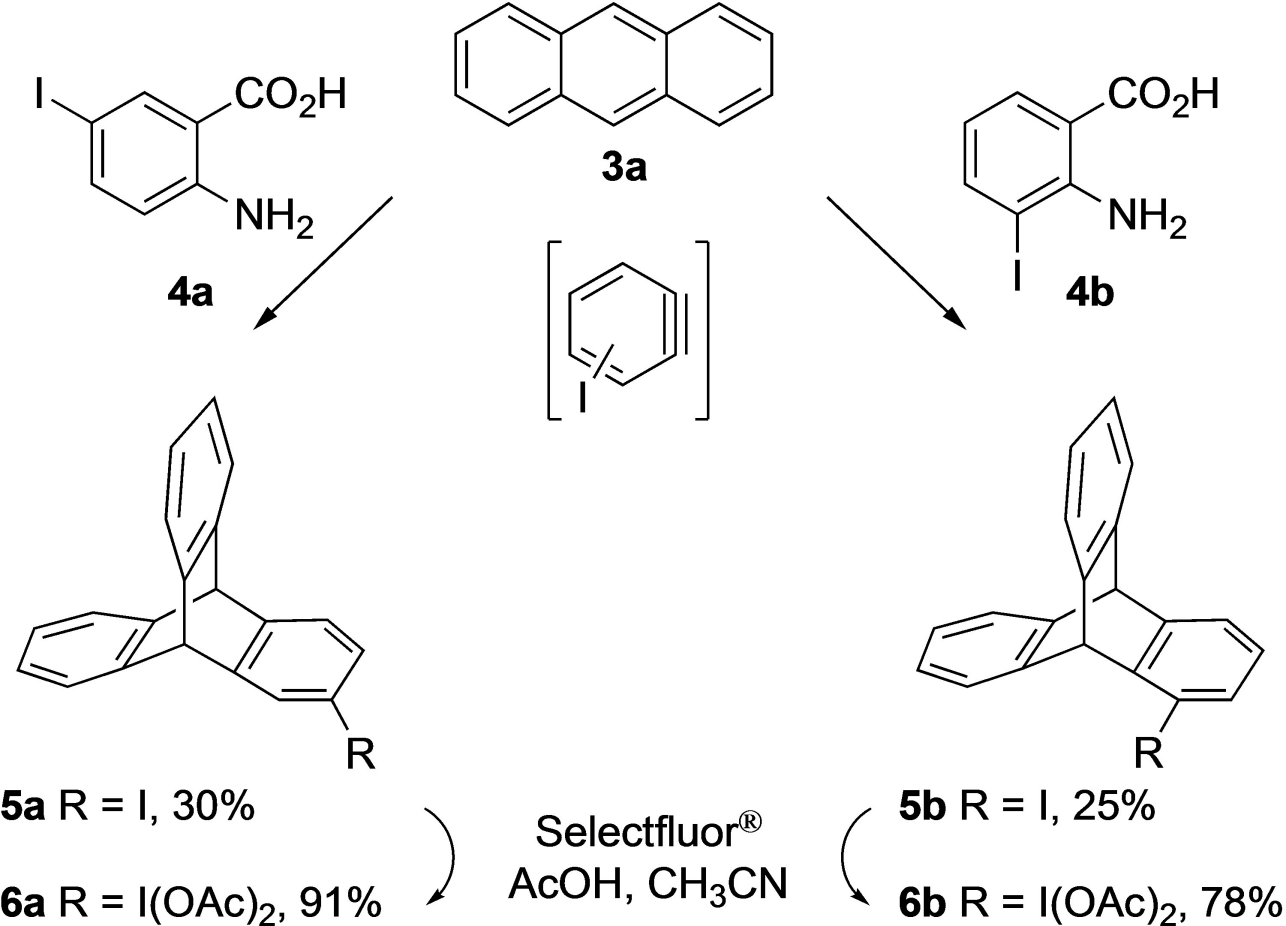
Process to obtain iodotriptycenes **5** the corresponding iodine(III) derivatives **6**.

Initially, the triptycene core structure bearing iodine at position 1 or 2 in one of the aromatic rings was prepared. For this approach, readily available 2‐amino‐5‐iodobenzoic acid **4 a** was reacted with anthracene **3 a** to form 2‐iodotriptycene **5 a** in 30 % yield (Scheme 1). Similarly, regioisomer **4 b** was reacted with **3 a** to form 1‐iodotriptycene **5 b** in 25 % yield (Scheme 1). Compounds **5 a** and **5 b** were fully characterized, including single‐crystal X‐ray structures.^[18]^

The hypervalent iodine(III) compounds **6 a** and **6 b** were synthesised in 91 % and 78 % yield, respectively, by reacting **5** with Selectfluor® in the presence of acetic acid and acetonitrile (see Supporting Information).

The 1‐ and 2‐iodo‐substituted triptycene compounds **5** were investigated as catalysts in the α‐oxytosylation of propiophenone. Propiophenone was reacted in presence of *para*‐toluenesulfonic acid, *meta*‐chloroperoxybenzoic acid and 10 mol % of iodotriptycene in acetonitrile at room temperature. Catalyst **5 a** provided the product in 62 % yield and catalyst **5 b** showed 76 % yield for the α‐oxytosylated propiophenone. These results showed that **5 a** and **5 b** can act as an iodine catalyst (see below). Catalysts **5 a** and **5 b** were recovered in about 50 % from the reaction mixture. These recovered catalysts were reused in this reaction and were found to be similarly efficient.

**Figure 2:**
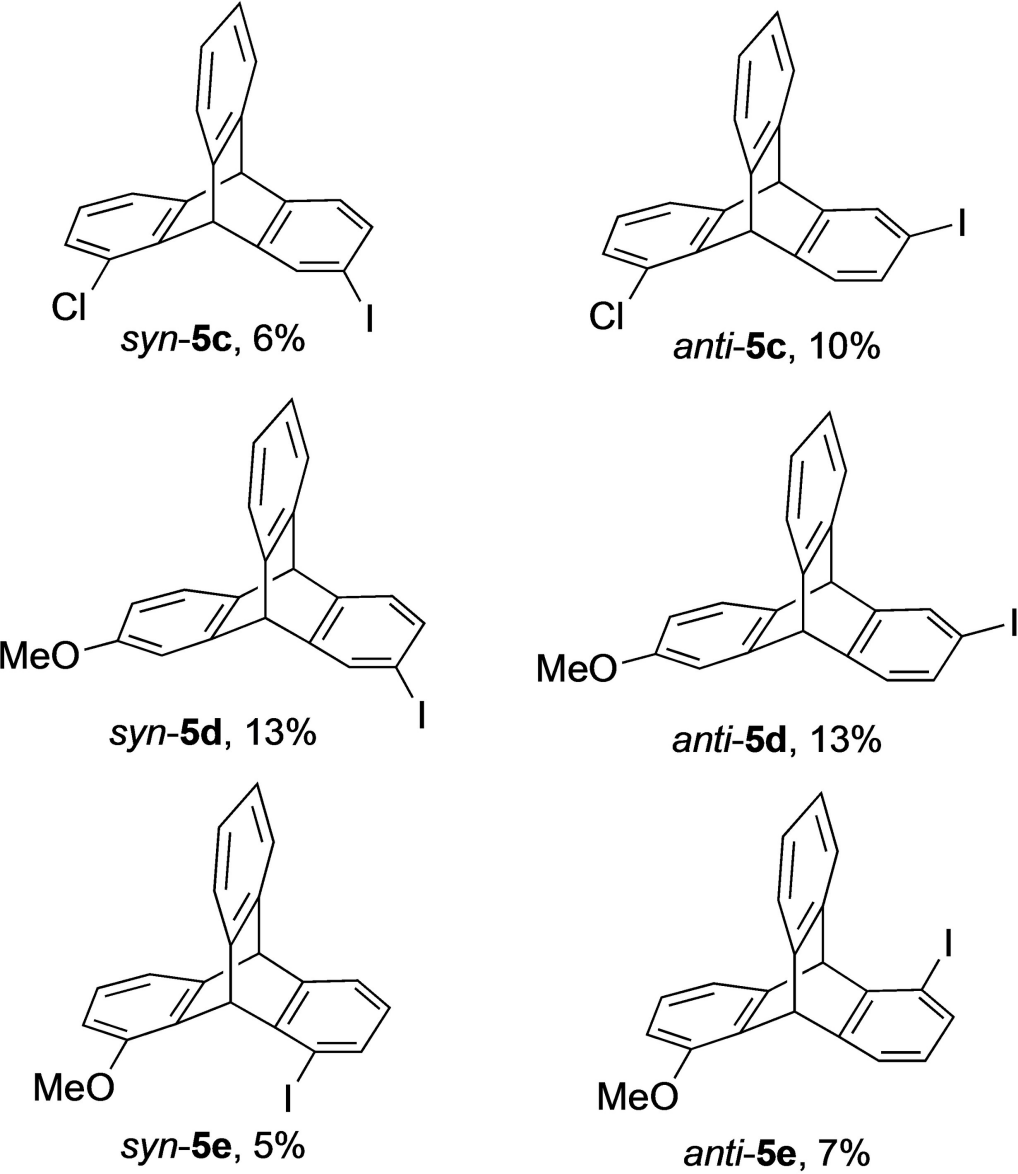
*Syn*‐ and *anti*‐ diastereomers of chiral iodotriptycene derivatives **5**.

Encouraged by these initial results, the synthesis of different chiral iodotriptycenes by reacting substituted anthracene derivatives with either **4 a** or **4 b** was carried out.^[19]^ The iodotriptycenes **5** obtained as Diels–Alder products are shown in Figure 2.

**Scheme 2:**
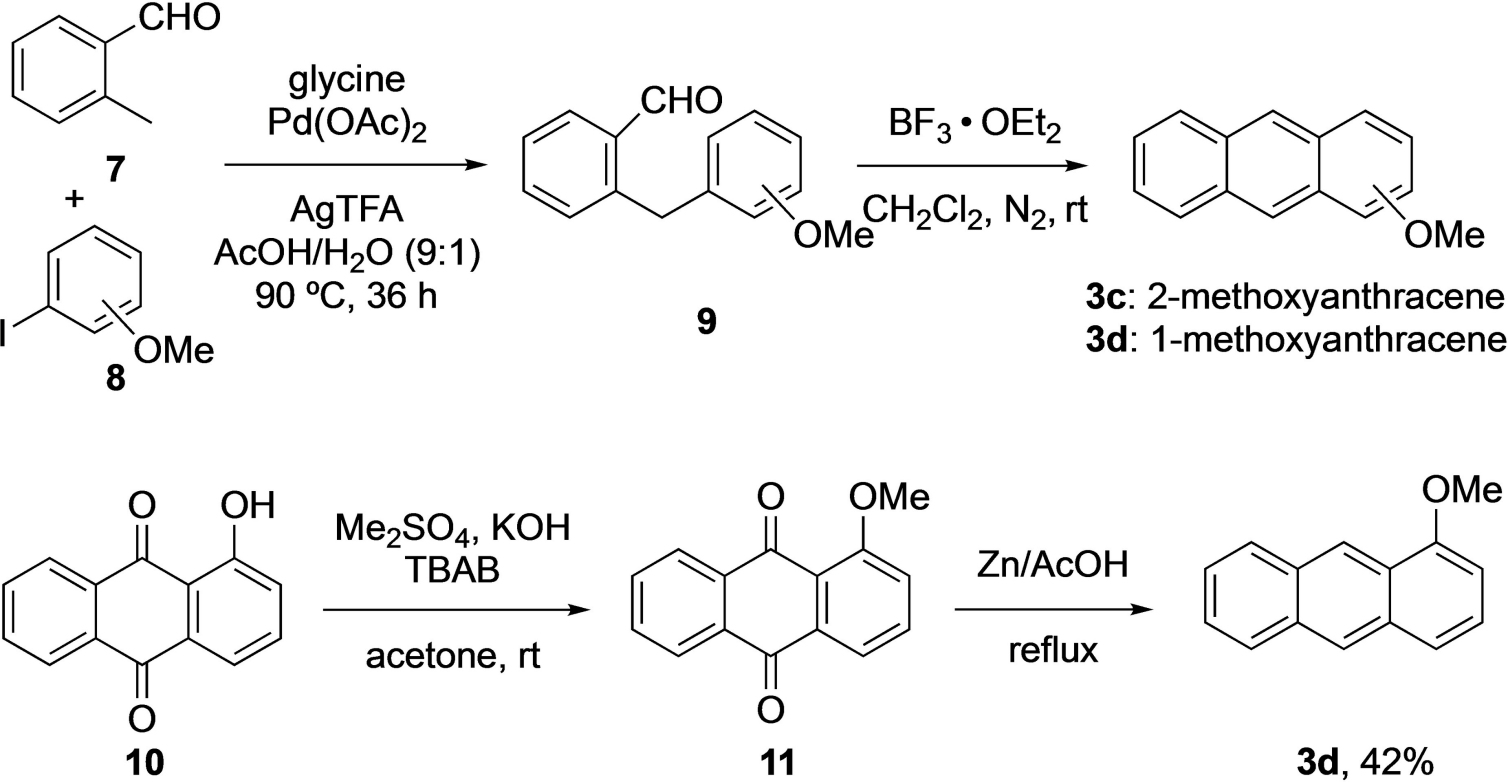
Synthetic routes to 1‐ and 2‐methoxyantracene **3 c** and **3 d** (TFA: trifluoroacetate; TBAB: tetrabutylammonium bromide).

To obtain **5 c** (Figure 2), commercially available 1‐chloroanthracene **3 b** was reacted with **4 a** under the same reaction conditions as described for compound **5 a** and **5 b**. The reaction provides a mixture of diastereomers **5 c** (*syn*:*anti*=1 : 1.6) in 16 % yield which were separated using preparative thin layer chromatography (TLC). The geometries of the isolated diastereomers were assigned by X‐ray crystallographic analysis, which identified the major compound as the *anti*‐product **5 c** (see Supporting Information). For the compound *syn*‐**5 c**, the enantiomers were separated using chiral HPLC columns to provide (−)‐*syn*‐**5 c** (*ee=*98 %) and (+)‐*syn*‐**5 c** (*ee*=67 %). To prepare other chiral iodotriptycene derivatives, 2‐methoxyanthracene **3 c** and 1‐methoxyanthracene **3 d** were synthesised in a stepwise process (Scheme 2). Compound **9** was prepared using a palladium‐catalysed C−H arylation of 2‐methylbenzaldehyde **7** with methoxyiodobenzene **8**.^[20]^ Compounds **9** were then cyclized to **3 c** and **3 d** on a gram scale using boron trifluoroetherate.^[21]^ Product **3 c** was obtained in 32 % yield whereas **3 d** was only observed in trace amounts due to the formation of a polymeric product (see Supporting Information). Other Lewis acids such as In(OTf)_3_,^[22]^ Bi(OTf)_3_, Sc(OTf)_3_, Cu(OTf)_2_, and FeCl_3_ also failed to provide **3 d**. In an alternative route, the methyl group was attached to 1‐hydroxyanthraquinone **10** with dimethyl sulfate to obtain compound **11**, which was reduced with zinc and acetic acid to yield **3 d** in 42 % yield.

Anthracene **3 c** was reacted with **4 a** to form the chiral triptycene **5 d** in 26 % yield as a 1 : 1 mixture of diastereomers. Similarly, compound **5 e** was prepared in 12 % by reacting **3 d** with **4 b**, which also provided a mixture of diastereomers in the ratio of 1 : 1.5. Multiple attempts to separate the *syn‐* and *anti*‐diastereomers of **5 d** and **5 e** were unsuccessful.

**Scheme 3:**
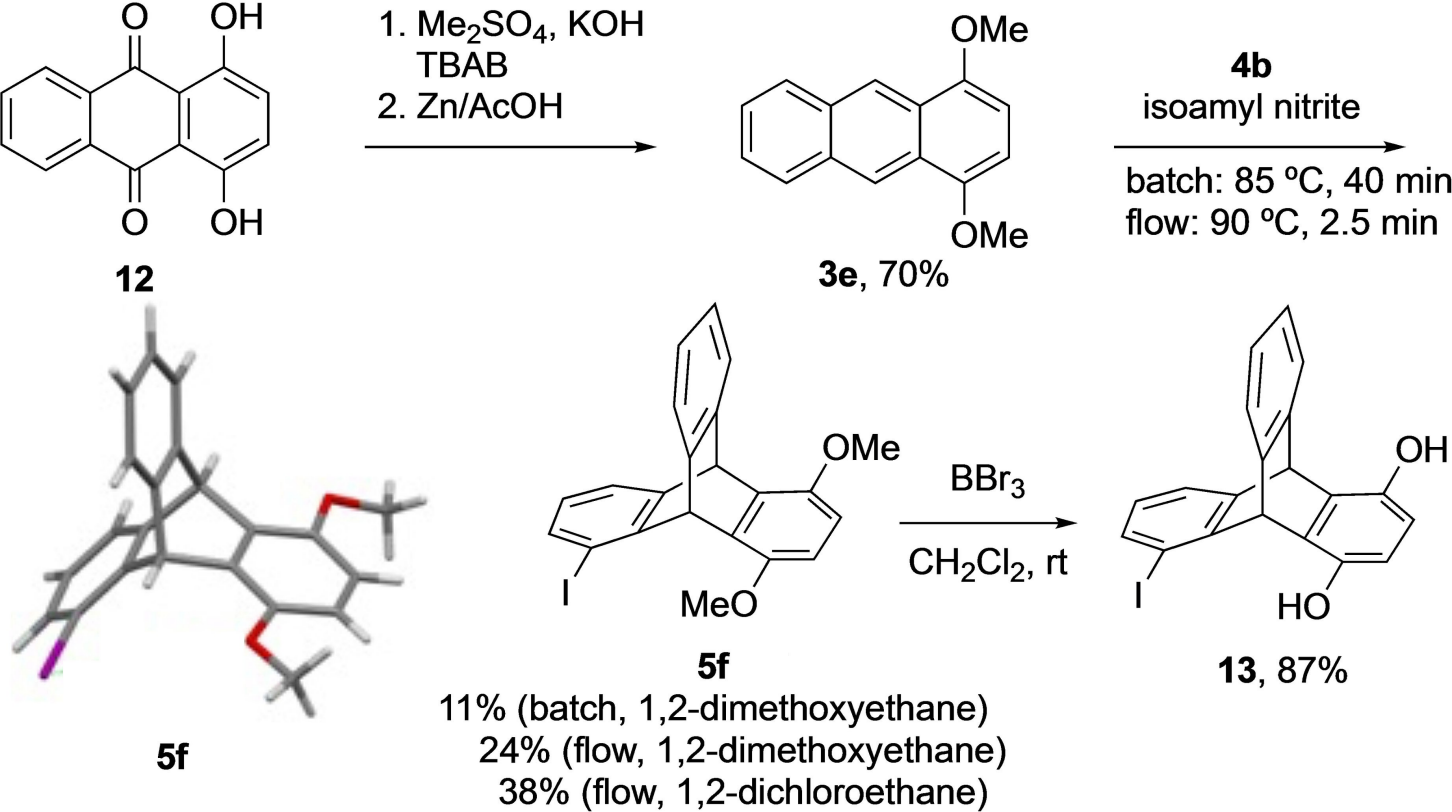
Synthesis of 5‐iodo‐1,4‐dimethoxytriptycene (**5 f**) and **13** (TBAB: tetrabutylammonium bromide).

As the position of iodine is not very close to the chiral centre in *syn*‐**5 c**, the synthesis of chiral 1‐iodosubstituted triptycenes was then envisaged. The use of symmetrical anthracenes will avoid the formation of diastereomers. For this approach, 1,4‐dimethoxyanthracene **3 e** was prepared from 1,4‐dihydroxyanthraquinone **12** through methylation and reduction similar to the reaction conditions described for **3 d** and used in the Diels–Alder reaction to obtain triptycene **5 f** (Scheme 3) in 11 % yield. Typically, the yields for triptycene products in this step are low, as a controlled addition of anthranilic acid derivatives is necessary to reduce benzyne dimerization. Owing to the advantages of controlled addition and intense mixing of the substrates and reagents in flow chemistry,^[23]^ this Diels–Alder reaction was performed in a flow system. A 0.25 m solution of **4 b** in 1,2‐dimethoxyethane and a 0.125 m solution of **3 e** in either 1,2‐dimethoxyethane or 1,2‐dichloroethane containing isoamyl nitrite were pumped with a flow rate of 0.2 mL min^−1^ (2.5 min residence time) through a PTFE coil at 90 °C. Interestingly, the reaction efficiency increased with 1,2‐dichloroethane as solvent, providing 38 % yield of **5 f** (1,2‐dimethoxyethane: 24 % yield of **5 f**). The racemic compound **5 f** obtained was fully characterised, including X‐ray analysis.^[18]^ Crystal structure shows an I⋅⋅⋅O distance of 4.58 Å. Before separating the enantiomers of **5 f**, its catalytic activity was investigated in the α‐oxytosylation of propiophenone that provided the desired product in 74 % yield. However, a separation of the enantiomers of **5 f** using chiral phase HPLC was not possible due to the very low solubility of **5 f** in solvents compatible with the chiral column.

**Scheme 4:**
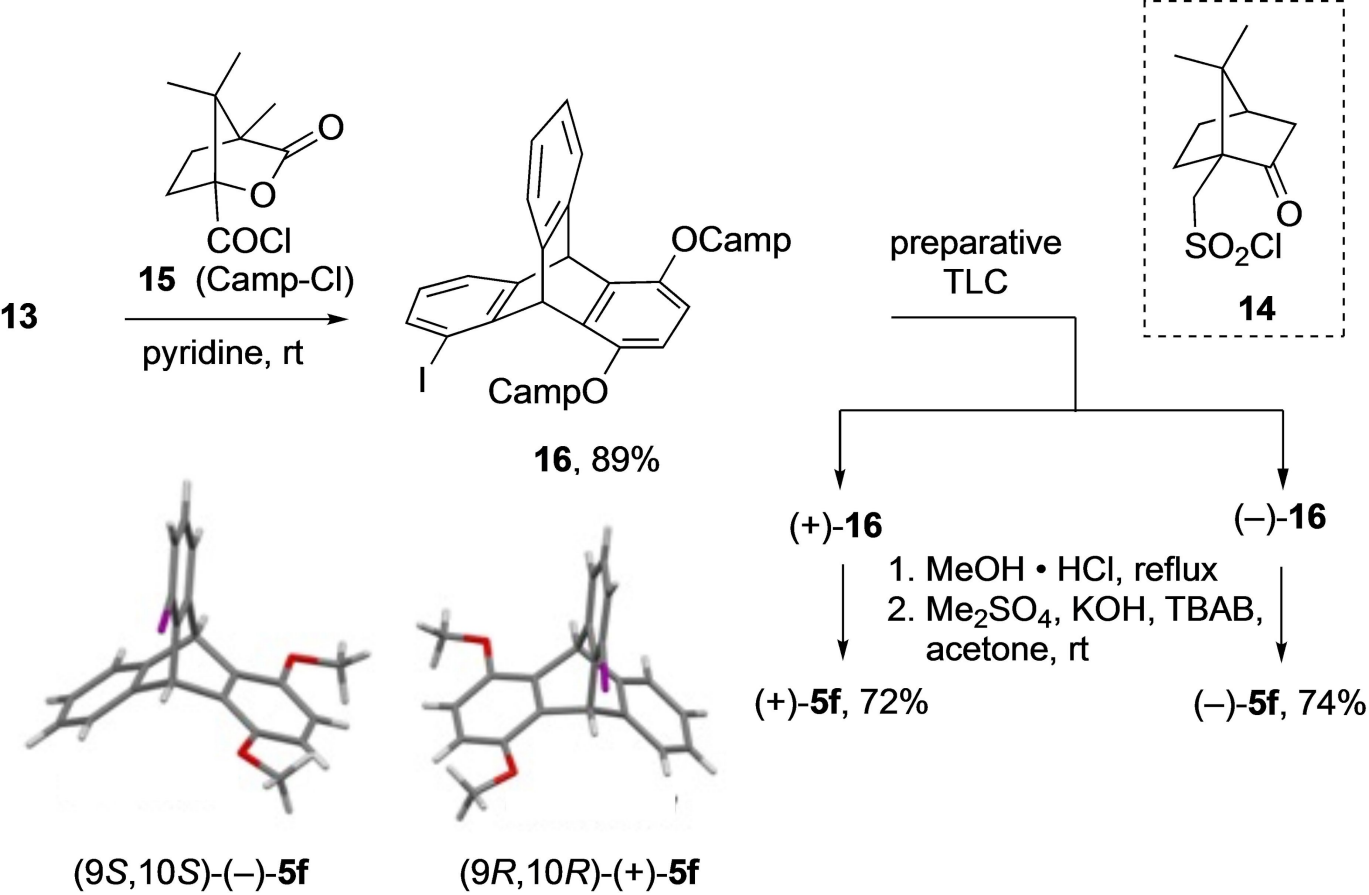
Resolution and isolation of enantiomers (+)‐**5 f** and (−)‐**5 f** (TBAB: tetrabutylammonium bromide).

To overcome this issue, both methyl groups from **5 f** were removed using BBr_3_, and 5‐iodo‐1,4‐dihydroxytriptycene **13** was obtained in 87 % yield (Scheme 3). When compound **13** was passed through the HPLC column aiming to separate the enantiomers, it was found to be unstable as it oxidized to the corresponding quinone (see Supporting Information). Therefore, **13** was initially reacted with (1*S*)‐(+)‐10‐camphorsulfonyl chloride **14** (Scheme 4) to prepare a diastereomeric mixture, but the resulting diastereomers were difficult to isolate and separate. However, when **13** was treated with (1*S*)‐(−)‐camphanic chloride **15**, the diastereomeric mixture could be separated using preparative TLC (Scheme 4). The camphyl substituents were then removed from the isolated isomers (+)‐**16** and (−)‐**16** by treatment with hydrochloric acid in methanol and the methyl groups were installed again using dimethyl sulfate in a one‐pot procedure without isolating the intermediate to afford (+)‐**5 f** and (−)‐**5 f** in 72 % and 74 % yield, respectively. The absolute configuration of each of the enantiomers was determined by their single‐crystal X‐ray structures. The HPLC purity was high and the enantiomeric excess was >99 % for both .

**Scheme 5:**
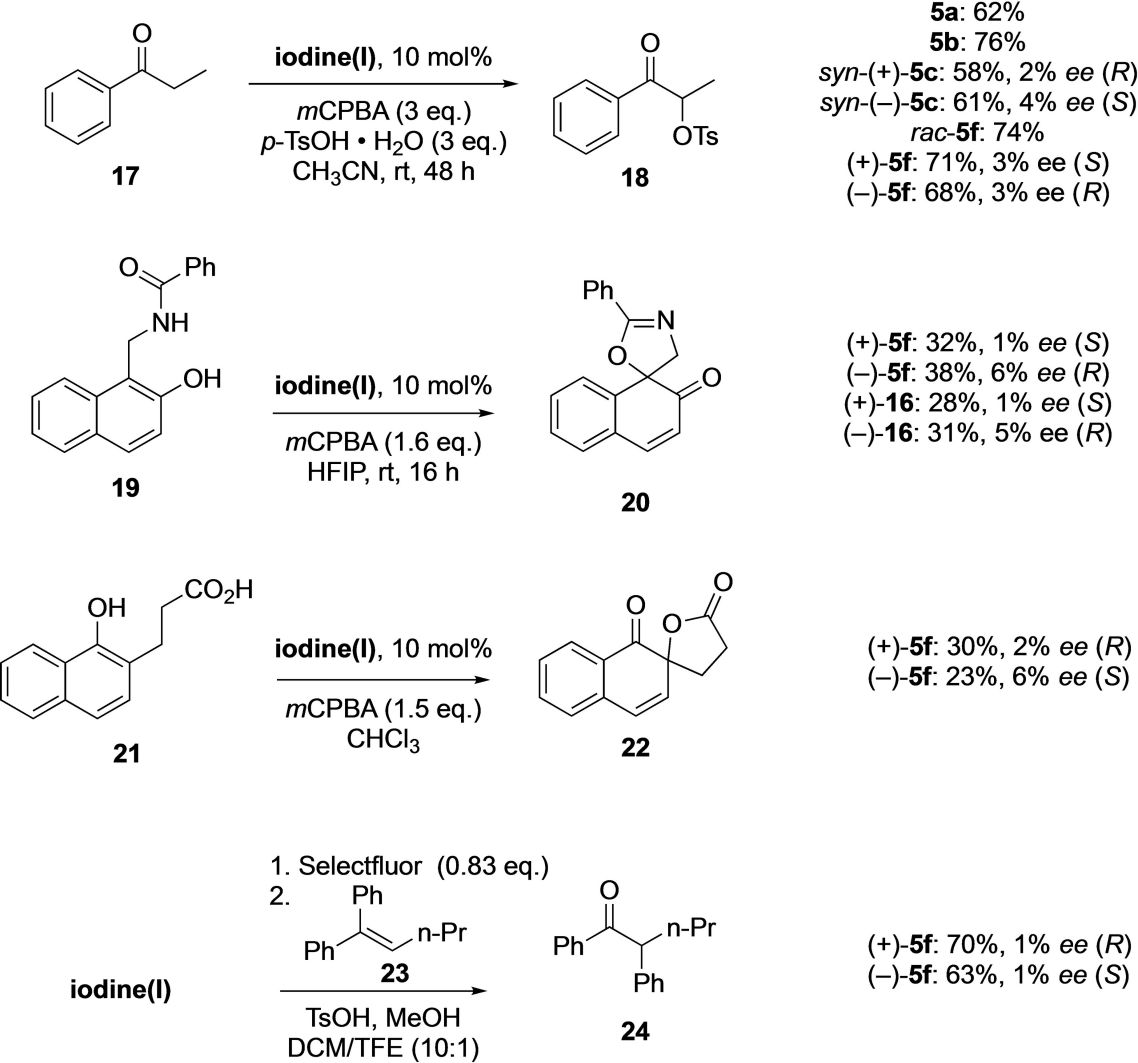
Oxidative transformations using iodine(III) compounds. *ee* determined by HPLC (*m*CPBA: *meta*‐chloroperoxybenzoic acid; HFIP: hexafluoroisopropanol).

Compounds (−)‐**5 f** and (+)‐**5 f** were subsequently investigated in stereoselective oxidative transformations as shown in Scheme 5. When used in the α‐oxytosylation of propiophenone, the product **18** was formed with 68 % (71 %) yield in only 3 % *ee*. Similarly, the use of (−)‐**5 f** and (+)‐**5 f** in the dearomatizing cyclization of **19** provided **20** in 38 % yield (6 % *ee*) and 32 % yield (1 % *ee*), respectively. (−)‐**5 f** and (+)‐**5 f** were also screened for the dearomative spirolactonization of **21**. (−)‐**5 f** provided the product **22** in 23 % yield with an enantioinduction of 6 % while (+)‐**5 f** resulted in a 30 % yield with 2 % *ee*. Furthermore, the oxidative rearrangement of pent‐1‐ene‐1,1‐diyldibenzene **23** was observed with both the catalysts (−)‐**5 f** and (+)‐**5 f**. (−)‐**5 f** showed the formation of the product **24** in 63 % yield (1 % *ee*) and (+)‐**5 f** in 70 % (1 % *ee*). The isolated intermediates (+)‐**16** and (−)‐**16** were also investigated for the dearomatizing cyclization of **19**. However, (+)‐**16** produced the compound **20** in 28 % yield (1 % *ee*) and (−)‐**16** in 31 % yield (5 % *ee*), indicating that these compounds are also not selective catalysts. The absolute configuration of the products **18**,[^17n^, ^24^] **20**,^[25]^ **22** ^[26]^ and **24** ^[27]^ were assigned using data reported in literature.

All the results obtained from the screened reactions show low enantioselectivities for the products (Scheme 5). This could be due to the rather large distance between the iodine and the coordinating methoxy group in **5 f** of about 4.58 Å. The design of a suitable coordinating ligand in closer proximity to iodine centre is desirable. New chiral iodotriptycene in stereoselective reactions are being explored and will be reported in due course.

## Conclusions

In conclusion, a reliable approach for the synthesis of iodotriptycenes has been established. Reacting either 3‐ or 5‐iodoanthranilic acid with differently substituted anthracenes provides iodotriptycenes. Iodotriptycenes were shown to be efficient catalysts in several oxidative organic transformations. For two chiral triptycene derivatives, the enantiomers were separated and investigated in enantioselective oxidative transformations. The enantioinduction in the products, however, was very low. This investigation provides new and useful information for the synthesis of chiral triptycenes including hypervalent iodine chemistry.

## Conflict of interest

The authors declare no conflict of interest.

1

## Supporting information

As a service to our authors and readers, this journal provides supporting information supplied by the authors. Such materials are peer reviewed and may be re‐organized for online delivery, but are not copy‐edited or typeset. Technical support issues arising from supporting information (other than missing files) should be addressed to the authors.

Supporting Information

## Data Availability

The data that support the findings of this study are available in the supplementary material of this article.
