# Supplementary material for: Chiral Iodotriptycenes: Synthesis and Catalytic Applications
Source: ChemistryOpen. 2022 Jul 13;11(7):e202200145. doi: 10.1002/open.202200145 (PMC9278095; doi:10.1002/open.202200145)
Supplement: Supplementary file 1 — Supporting Information [file OPEN-11-e202200145-s001.pdf]

ChemistryOpen

Supporting Information

Chiral Iodotriptycenes: Synthesis and Catalytic Applications

Nasim Khan, Katsunori Itaya, and Thomas Wirth*

Supporting Information

Table of contents Page

**1.**	**General Methods**	S3
**2.**	**General Procedures**	S5
	**General procedure for the syntheses of triptycenes (GP 1)**	S5
	Synthesis of 2-iodo-9,10-dihydro-9,10-[1,2]benzenoanthracene (**5a**)	S5
	Synthesis of 1-iodo-9,10-dihydro-9,10-[1,2]benzenoanthracene (**5b**)	S5
	**General procedure for the syntheses of (Diacetoxyiodo)triptycene (TripIDA, GP 2)**	S6
	Synthesis of (9,10-dihydro-9,10-[1,2]benzenoanthracen-2-yl)-l^3-iodanediyl diacetate (**6a**)	S6
	Synthesis of 9,10-dihydro-9,10-[1,2]benzenoanthracen-1-yl)-l3-iodanediyl diacetate (**6b**)	S7
	Synthesis of 1-chloro-7-iodo-9,10-dihydro-9,10-[1,2]benzenoanthracene (*syn*-**5c**) and 1-chloro-6-iodo-9,10-dihydro-9,10-[1,2]benzenoanthracene (*anti*-**5c**)	S7
	Synthesis of 2-iodo-7-methoxy-9,10-dihydro-9,10-[1,2]benzenoanthracene (*syn*-**5d**) and 2-iodo-6-methoxy-9,10-dihydro-9,10-[1,2]benzenoanthracene (*anti*-**5d**)	S8
	**General procedure for the synthesis of substituted 2-benzylbenzaldehyde with slight modification (GP 3)**	S9
	Synthesis of 2-(4-methoxybenzyl)benzaldehyde (**9a**)	S9
	Synthesis of 2-(2-methoxybenzyl)benzaldehyde (**9b**)	S10
	**General procedure for the synthesis of anthracene from substituted 2-benzylbenzaldehyde with slight modification (GP 4)**	S10
	Synthesis of 2-methoxyanthracene (**3c**)	S10
	Synthesis of 1',4-dimethoxy-1,9'-bianthracene (**3f**)	S11
	**General procedure for the methylation of 1-hydroxyanthracene-9,10-dione (10)/1,4-dihydroxyanthracene-9,10-dione (12) using dimethyl sulfate with slight modification (GP5)**	S11
	Synthesis of 1-methoxyanthracene-9,10-dione (**11**)	S12
	**General procedure for the synthesis of anthracenes using zinc and acetic acid with slight modification (GP6)**	S12
	Synthesis of 1-methoxyanthracene (**3d**)	S13
	**Synthesis of 1-iodo-8-methoxy-9,10-dihydro-9,10-[1,2]benzenoanthracene (*syn*-5e) and 1-iodo-5-methoxy-9,10-dihydro-9,10-[1,2]benzenoanthracene (*anti*-5e)**	S13
	Synthesis of 1,4-dimethoxyanthracene-9,10-dione (**12a**)	S14
	Synthesis of 1,4-dimethoxyanthracene (**3e**)	S14
	Synthesis of 5-iodo-1,4-dimethoxy-9,10-dihydro-9,10-[1,2]benzenoanthracene (**5f**)	S14
	Synthesis of 5-iodo-9,10-dihydro-9,10-[1,2]benzenoanthracene-1,4-diol (**13**)	S16
	Synthesis of (5-iodo-9,10-dihydro-9,10-[1,2]benzenoanthracene-1,4-diyl (1S,1'S,4S,4'S)-bis(4,7,7-trimethyl-3-oxo-2-oxabicyclo[2.2.1]heptane-1-carboxylate) (**16**)	S16
	Synthesis of (–)-**5f** and (+)-**5f** from (–)-**16** and (+)-**16** respectively in one-pot reaction	S18
	Spectroscopic data for 1-iodo-9,10-dihydro-9,10-[1,2]benzenoanthracene-13,16-dione (**13a**)	S18
	Synthesis of (1*S*,4*R*)-1-4-(((1*S*,4*S*)-7,7-dimethyl-2-oxobicyclo[2.2.1]heptan-1-yl)methoxy)-5-iodo-9,10-dihydro-9,10-[1,2]benzenoanthracen-1-yl)oxy)methyl)-7,7-dimethylbicyclo[2.2.1]heptan-2-one-sulfur(IV) oxide (**16a**)	S19
	Procedure for the α-tosylation of propiophenone **(GP 7)**	S20
	Procedure for the dearomatizing cyclization of **19**	S20

Procedure for the dearomative spirolactonization of **21** S22
Procedures for the rearrangement of pent-1-ene-1,1-diyldibenzene **23** S24
3. References S27
4. X-Ray analysis S28
X-ray diffraction for **5a** S28
X-ray diffraction for **5b** S28
X-ray diffraction for *anti*-**5c** S28
X-ray diffraction for **5f** S28
X-ray diffraction for (9*S*, 10*S*)-(-)-**5f** S29
X-ray diffraction for (9*R*, 10*R*)-(+)-**5f** S29
5. NMR and Mass Spectra S30
6. HPLC chromatograms S72

Chiral Iodotriptycenes: Synthesis and Catalytic Applications

1. General Methods

Reactions:
The reactions were performed using standard laboratory equipment. All air sensitive reactions were carried out under argon or nitrogen atmosphere using oven dried glassware. All reaction were stirred using a stirrer plate and a magnetic stirrer bar and heating, if necessary, over a hotplate with a temperature probe control and an adapted heating block. Lower temperatures were achieved using ice/water bath (0 °C), dry ice/acetone bath (–78 °C).

Solvents:
Dry acetonitrile was collected from a solvent purification system (SPS) from the company M BRAUN (MB SPS-800). Dry dichloromethane was distilled over calcium hydride under nitrogen atmosphere. Other dry solvents and chemicals were purchased from Sigma Aldrich, Alfa Aesar, Acros Organic, FluoroChem or TCI UK and were used without further purification.

Reagents:
All reagents were purchased from commercial sources such as Acros Organics, Alfa Aesar, Fisher Scientific, FluoroChem, Merck, Sigma Aldrich and TCI and were used as received unless mentioned otherwise.

Solvent evaporators:
Rotary Evaporators were used for solvent evaporations (reduced pressure up to 15 mbar) and a high vacuum apparatus was used to further dry the products.

Thin layer chromatography (TLC):
All reactions were monitored by thin-layer chromatography (TLC) which was performed on precoated aluminium sheets of Merck silica gel 60 F254 (0.20 m) and visualised by UV radiation (254 nm) or/and by staining with ceric ammonium molybdate solution (235 mL distilled H_2O, 12 g ammonium molybdate, 0.5 g ceric ammonium molybdate, 15 mL concentrated sulfuric acid), potassium permanganate solution (1.5 g $KMnO_4$, 10 g K_2CO_3, 1.25 mL 10% NaOH, 200 mL distilled H_2O) or iodine.

Flash column chromatography:
Flash column chromatography was performed using *Merck* silica gel 60 (40-63 µm) to purify products applying nitrogen pressure or on a *Biotage Isolera Four* using *Biotage* cartridges *SNAP Ultra 10 g*, *SNAP Ultra 25g*, *SNAP Ultra 50g* and *SNAP Ultra 100g*. The solvents were used as laboratory grade.

NMR:
NMR spectra were recorded at 298 K on a *Bruker DPX 400* or *Bruker DPX 500*. All resonances are reported relative to TMS. Spectra were calibrated relative to solvents' residual proton and carbon chemical shifts: ($CDCl_3$: δ = 7.26 ppm for ^1H NMR and δ = 77.16 for ^{13}C NMR). Chemical shifts δ were given in ppm and the multiplicity of the signals was reported as: s = singlet, d = doublet, t = triplet, q = quartet, dd = doublet of doublets, dt = doublet of triplet, m = multiplet, brs = broad singlet. The coupling constants (*J*) in Hertz.

Mass:
Mass spectrometric measurements were performed by R. Jenkins, R. Hick, T. Williams, and S. Waller at Cardiff University on a Water LCR Premier XE. Ions were generated by

Electrospray (ES) or Electron Ionisation (EI). The molecular ion peak values quoted for molecular ion plus hydrogen $[M+H]^+$.

IR:
IR spectra were recorded on Shimadzu IR Affinity-1S apparatus. Wavenumbers are quoted in cm^{-1}.

Melting points:
Melting points were measured using a Gallenkamp variable heater with samples in open capillary tubes and are not corrected.

HPLC:
The HPLC measurements were performed on a Shimadzu apparatus. The different modules were SIL-10ADVP (auto injector), LC-10ATVP (liquid chromatograph), FCV-10ALVP (pump), DGU-14A (degasser), CTO-10ASVP (column oven), SCL-10AVP (system controller) and SPD-M10A (diode array detector). The solvents used were hexane, 2-propanol and THF as HPLC grade. The chiral column used for the separation of the enantiomers were YMC CHIRAL Amylose-C S-5µm (0.46 cm Ø×25 cm) and CHIRALCEL OD-H S-5µm (0.46 cm Ø×25 cm). Polaris-Si S-5µm (0.46 cm Ø×25 cm) was used for the peak resolutions of the diastereomers.

Optical rotation:
Optical rotation was measured with a SCHMIDT and HAENSCH UniPol L polarimeter at 20 °C in a cuvette of 50 mm length with a sodium light (589.30 nm).

X-Ray crystallography:
X-Ray crystallographic studies were carried out at the X-Ray Crystallography Service at Cardiff University. The data were collected on an Agilent SuperNova Dual Atlas diffractometer with a mirror monochromator, equipped with an Oxford cryosystems cooling apparatus. Crystal structures were solved and refined using SHELX. Nonhydrogen atoms were refined with anisotropic displacement parameters. Hydrogen atoms were inserted in idealised positions. The structure was solved by a direct Method and refined by a full matrix least-squares procedure on F2 for all reflections (SHELXL-97).

2. General procedures

General Procedure for the Synthesis of Triptycenes (GP 1):

All iodotriptycene and their derivatives were synthesised according to the literature procedures[S1] with slight modifications.

To a 50 mL two necked flask with magnetic stir bar placed on sand bath in reflux mode under nitrogen was added anthracene (4.49 mmol, 800 mg), 1,2-dimethoxyethane (10 mL) and heated until all solid material is dissolved. To the clear solution isoamyl nitrite (5.95 mmol, 698 mg) was added at the reflux temperature. In an additional flask, 3- or 5-iodoanthranilic acid (7.59 mmol, 1996 mg) was dissolved in 7 mL 1,2-dimethoxyethane and was added drop wise over 20 minutes to the clear solution. After complete addition, further isoamyl nitrite (5.95 mmol, 697 mg) was added with 1 mL 1,2-dimethoxyethane and the reaction mixture was refluxed for 10 minutes. After completion of the reaction, it was cooled to room temperature. **Work-up:** At room temperature under stirring 10 mL ethanol was added to the reaction mixture. Additional 20 mL 3 N NaOH solution was added and stirred for 15 minutes. The precipitates were filtered and washed with 10 mL solvent mixture (ethanol / water = 1:1). The solid residue was discarded. The solvent in the filtrate was removed using a rotary evaporator. 20 mL water and 30 mL ethyl acetate were added and the solution was stirred. The organic layer was separated using a separating funnel. The organic layer was dried over anhydrous magnesium sulfate. The solvent was removed using a rotary evaporator. The product was isolated using column chromatography with 4% ethyl acetate in hexane. High purity of the compound was achieved using preparative TLC using in 100% hexane or 2% ethyl acetate in hexane.

Synthesis of 2-iodo-9,10-dihydro-9,10-[1,2]benzenoanthracene (5a):

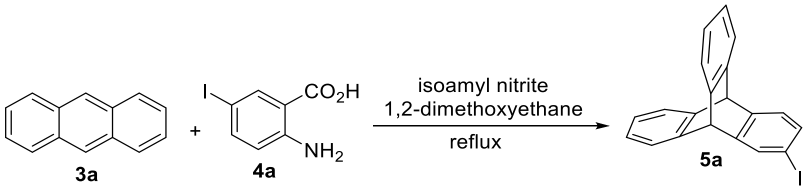

White solid; 516 mg, 30% yield; M.p.: 195–196 °C; R_f = 0.34 (hexane : ethyl acetate = 9.6:0.4).

^{1}H NMR (500 MHz, $CDCl_3$): δ = 7.68 (d, J = 1.5 Hz, 1H), 7.35–7.31 (m, 4H), 7.28–7.27 (dd, J = 7.5, 1.5 Hz, 1H), 7.08 (d, J = 7.5 Hz, 1H), 6.98–6.94 (m, 4H), 5.33 (s, 1H, CH), 5.32 (s, 1H, CH) ppm.

^{13}C NMR (126 MHz, $CDCl_3$): δ = 148.0, 145.4, 144.9, 144.6, 134.2, 132.7, 125.6. 125.5, 123.9, 123.8, 90.1, 53.7, 53.6 ppm.

HRMS (AP$^+$) Calcd for $C_{20}H_{13}I$ = 380.0062 and Found = 380.0063.

IR (neat): 3068, 3047, 3005, 2958, 2924, 2850, 1454, 1400, 1274, 1261, 1190, 1163, 1053, 1020, 813, 798, 744, 626, 509 cm^{-1}.

Synthesis of 1-iodo-9,10-dihydro-9,10-[1,2]benzenoanthracene (5b):

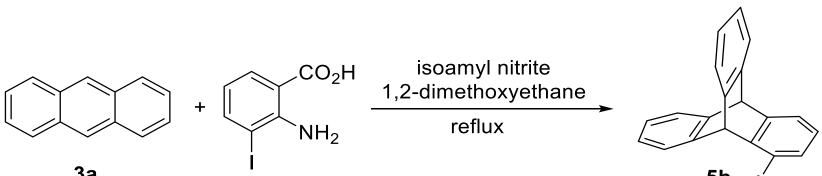

3a 4b 5b

White solid; 225 mg, 25% yield; M.p.: 184–185 °C; R_f = 0.31 (hexane : ethyl acetate = 9.6 : 0.4).

^{1}H NMR (400 MHz, $CDCl_3$): δ = 7.49-7.46 (m, 2H), 7.44–7.38 (m, 3H), 7.32 (d, *J* = 6.8 Hz, 1H), 7.06–7.01 (m, 4H), 6.71–6.67 (td, *J* = 7.2, 0.8 Hz, 1H), 5.79 (s, 1H, CH), 5.40 (s, 1H, CH) ppm.

^{13}C NMR (101 MHz, $CDCl_3$): δ = 148.6, 147.3, 145.1, 144.5, 135.0, 127.1, 125.6, 125.5, 124.2, 123.8, 123.7, 94.2, 58.2, 54.8 ppm.

HRMS (EI$^+$) Calcd for $C_{20}H_{13}I$ = 380.0062 and Found = 380.0069.

IR (neat): 3066, 3039, 3020, 2970, 1456, 1421, 1192, 1161, 1095, 906, 815, 794, 750, 742, 725, 667, 630 cm^{-1}.

General procedure for the synthesis of (diacetoxyiodo)triptycene (TripIDA, GP 2):

1- or 2-(Diacetoxyiodo)triptycenes were synthesised according to the literature procedures [S2] with slight modifications.

General Procedure (GP 2):
To a solution of iodotriptycene (50 mg, 0.131 mmol) and acetonitrile (1 mL) in a 10 mL round bottom single neck flask placed on sand bath under stirring acetic acid (0.1 mL) was added followed by Selectfluor® (120 mg, 0.338 mmol). The reaction mixture was stirred for 5 minutes at room temperature then heated to 40 ºC until the reaction shows disappearance of iodotriptycene on TLC. **Non-aqueous work-up and isolation of the product:** After completion of the reaction, the solvent was removed on a rotary evaporator under reduced pressure, then ethyl acetate 5 mL was added and the solution stirred at room temperature for 10 minutes. The precipitated solid was filtered off and washed twice with ethyl acetate. The solid residue was discarded. The ethyl acetate filtrate was collected in a round bottom flask and the solvent was removed on a rotary evaporator to obtain the product.

Synthesis of (9,10-dihydro-9,10-[1,2]benzenoanthracen-2-yl)-λ^3-iodanediyl diacetate (6a):

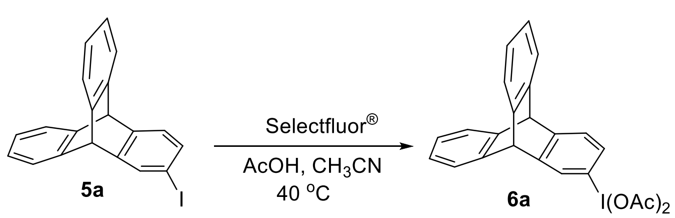

5a 6a

Following **GP 2**, 2-iodotriptycene **5a** (50 mg, 0.131 mmol), acetonitrile (1 mL) acetic acid (0.1 mL) and Selectfluor® (120 mg, 0.338 mmol) in 5 h afforded the product **6a**.
Beige solid; 59 mg, 91% yield; M.p.: 143–144 °C.

^{1}H NMR (500 MHz, CDCl$_3$): δ = 8.08 (d, J = 1.5 Hz, 1H), 7.76–7.74 (dd, J = 8.0, 2.0 Hz, 1H), 7.48 (d, J = 8.0 Hz, 1H), 7.44–7.40 (m, 4H), 7.07–7.03 (m, 4H), 5.51 (s, 1H, CH), 5.49 (s, 1H, CH), 1.96 (s, 6H, 2 x CH$_3$) ppm.

^{13}C NMR (126 MHz, CDCl$_3$): δ = 176.6, 149.6, 148.5, 144.2, 144.1, 132.9, 130.2, 126.2, 125.9, 125.5, 124.2, 124.1, 117.4, 54.0, 53.8, 20.5 ppm.

IR (neat): 3066, 3015, 2964, 2932, 1646, 1587, 1456, 1362, 1288, 1268, 1240, 1217, 1193, 1163, 1005, 916, 822, 740, 665, 627 cm^{-1}.

Synthesis of 9,10-dihydro-9,10-[1,2]benzenoanthracen-1-yl)-λ^3-iodanediyl diacetate (6b)

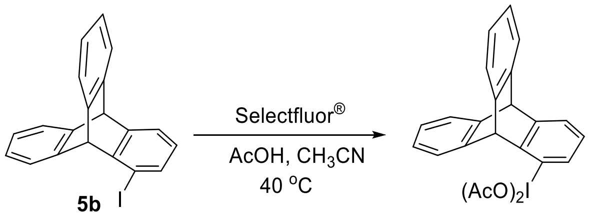

6b

Following **GP 2**, 1-iodotriptycene **5b** (50 mg, 0.131 mmol), acetonitrile (1 mL) acetic acid (0.1 mL) and Selectfluor® (120 mg, 0.338 mmol) in 3 h afforded the product **6b**.

White solid; 51 mg, 78% yield; M.p.: 110-112 °C.

^{1}H NMR (500 MHz, CDCl$_3$): δ = 7.76 (d, J = 8.0 Hz, 1H), 7.58 (d, J = 7.5 Hz, 1H), 7.48–7.44 (m, 2H), 7.43–7.40 (m, 2H), 7.06–7.03 (m, 5H), 5.76 (s, 1H, CH), 5.52 (s, 1H, CH), 1.95 (s, 6H, 2 x CH$_3$) ppm.

^{13}C NMR (126 MHz, CDCl$_3$): δ = 176.6, 148.5, 148.1, 144.5, 143.7, 132.5, 127.8, 127.6, 126.0, 125.8, 124.7, 123.9, 121.0, 57.3, 54.7, 20.5 ppm.

IR (neat): 3072, 3022, 2983, 2931, 1643, 1458, 1451, 1433, 1363, 1292, 1269, 1196, 1162, 1056, 1003, 924, 846, 801, 757, 748, 734, 665, 630 cm^{-1}.

Synthesis of 1-chloro-7-iodo-9,10-dihydro-9,10-[1,2]benzenoanthracene (*syn*-5c) and 1-chloro-6-iodo-9,10-dihydro-9,10-[1,2]benzenoanthracene (*anti*-5c)

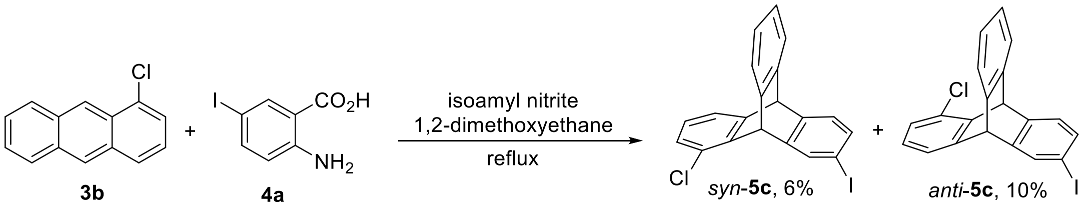

syn : *anti* = 1:1.6

Following **GP 1**: 1-chloroanthracene **3b** (4.7 mmol, 1 g), 1,2-dimethoxyethane (10 mL), isoamyl nitrite (6.22 mmol, 730 mg), 2-amino-5-iodobenzoic acid **4a** (7.94 mmol, 2.09 g) in 1,2-dimethoxyethane (7 mL), isoamyl nitrite (6.22 mmol, 0.73 g).

The crude compounds subjected for preparative TLC using 4% ethyl acetate in hexane to obtain *anti*-**5c** (impure) = 217 mg (yield: 11%) and *syn*-**5c** (impure) = 339 mg (yield: 17%). Each diastereoisomer was further purified to remove close impurities through a second purification using preparative TLC (hexane).

anti-**5c**
Off white solid; 190 mg, 10% yield; M.p.: 130–131 °C; R_f = 0.65 (hexane : ethyl acetate = 9.6 : 0.4).

^{1}H NMR (500 MHz, $CDCl_3$): δ = 7.65 (brs, 1H), 7.35 (brs, 1H), 7.30 (s, 1H), 7.26 (d, J = 7.5 Hz, 1H), 7.17 (d, J = 7.0 Hz, 1H), 7.10 (d, J = 7.5 Hz, 1H), 7.00–6.90 (brs, 3H), 6.85–6.82 (m, 1H), 5.78 (s, 1H, CH), 5.28 (s 1H, CH) ppm.

^{13}C NMR (126 MHz, $CDCl_3$): δ = 147.7, 147.1, 144.4, 144.3, 143.8, 142.3, 134.5, 132.8, 129.8, 126.7, 126.1, 126.0, 125.9, 125.8, 124.3, 124.0, 122.3, 90.4, 53.8, 50.1 ppm.

HRMS (AP^+) Calcd for $C_{20}H_{12}ClI$ = 413.9672 and Found = 413.9679.

IR (neat): 3065, 3018, 2982, 2960, 2919, 2849, 1571, 1454, 1431, 1400, 1262, 1187, 1161, 1108, 1055, 935, 905, 861, 816, 786, 749, 689, 636 cm^{-1}.

syn-**5c**
Off white solid; 115 mg, 6% yield; Mp; 105–106 °C; R_f = 0.52 (hexane : ethyl acetate = 9.6 : 0.4).

^{1}H NMR (500 MHz, $CDCl_3$): δ = 7.79 (d, J = 1.5 Hz, 1H), 7.46–7.42 (m, 1H), 7.40–7.37 (m, 1H), 7.36–7.34 (dd, J = 8.0, 1.5 Hz, 1H), 7.26 (d, J = 6.0 Hz, 1H, merged with solvent peak), 7.14 (d, J = 7.5 Hz, 1H), 7.06–7.01 (m, 3H), 6.94–6.91 (m, 1H), 5.86 (s, 1H, CH), 5.39 (s, 1H, CH) ppm.

^{13}C NMR (126 MHz, $CDCl_3$): δ = 147.4, 146.9, 145.2, 144.7, 143.6, 142.1, 134.5, 133.1, 129.8, 126.7, 126.1, 125.9, 125.8, 125.7, 124.4, 123.9, 122.2, 90.3, 53.9, 50.0 ppm.

HRMS (AP^+) Calcd for $C_{20}H_{12}ClI$ = 413.9672 and Found = 413.9670.

IR (neat): 3064, 3017, 2982, 2961, 2926, 2853, 1560, 1454, 1432, 1400, 1262, 1186, 1161, 1108, 1054, 935, 905, 872, 819, 751, 686, 636 cm^{-1}.

Synthesis of 2-iodo-7-methoxy-9,10-dihydro-9,10-[1,2]benzenoanthracene (*syn*-5d) and 2-iodo-6-methoxy-9,10-dihydro-9,10-[1,2]benzenoanthracene (*anti*-5d)

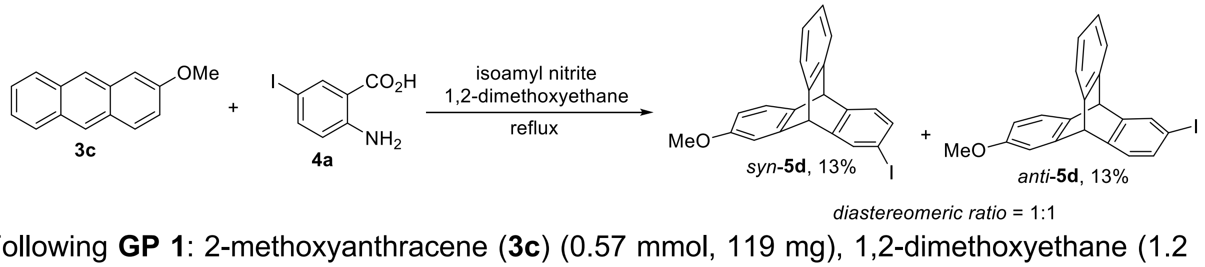

Following **GP 1**: 2-methoxyanthracene (**3c**) (0.57 mmol, 119 mg), 1,2-dimethoxyethane (1.2 mL), isoamyl nitrite (0.75 mmol, 89 mg), 2-amino-5-iodobenzoic acid (**4a**) (0.96 mmol, 253 mg) in 0.88 mL 1,2-dimethoxyethane, isoamyl nitrite (0.75 mmol, 89 mg) in 0.1 mL 1,2-

dimethoxyethane and the reaction mixture was refluxed for 10 minutes to afford the product (a mixture of diastereomers in the ratio of 1:1).

Pale brown solid; 58 mg, 26% yield. R_f = 0.24 (hexane : ethyl acetate = 9.6 : 0.4).

^{1}H NMR (500 MHz, $CDCl_3$): δ = 7.71 (d, J = 2.0 Hz, 1H), 7.69 (d, J = 1.5 Hz, 1H), 7.36-7.33 (m, 4H), 7.32-7.31 (dd, J = 3.0, 1.5 Hz, 1H), 7.31-7.30 (dd, J = 3.0, 1.5 Hz, 1H), 7.26-7.24 (2H, merged with $CHCl_3$ residual peak), 7.11-7.10 (m, 2H), 7.00 – 6.97 (m, 6H), 6.51 – 6.50 (dd, J = 2.5, 1.0 Hz, 1H), 6.49 – 6.48 (dd, J = 2.5, 1.0 Hz, 1H), 5.31 (s, 1H, CH), 5.30 (s, 1H, CH), 5.29 (s, 1H, CH), 5.28 (s, 1H, CH), 3.73 (6H, 2 x OCH_3) ppm.

^{13}C NMR (126 MHz, $CDCl_3$): δ = 157.5, 148.4, 147.8, 146.4, 146.1, 145.8, 145.3, 145.2, 145.0, 144.7, 144.4, 137.2, 136.9, 134.2, 134.1, 132.7, 132.5, 128.6, 125.6 (2C), 125.5, 125.4, 125.3, 124.4, 124.3, 123.9, 123.8, 123.7, 123.6, 121.2, 111.1, 111.0, 109.7, 109.5, 90.2, 89.9, 55.7, 55.6, 53.9, 53.8, 52.9, 52.8 ppm.

General procedure for the synthesis of substituted 2-benzylbenzaldehydes (GP 3):[S3]

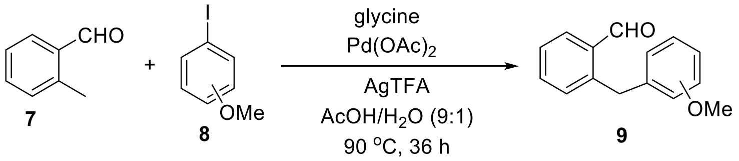

To a 250 mL single neck round bottom flask with magnetic stir bar placed on sand bath in reflux mode was added 2-methylbenzaldehyde (20 mmol, 2.4 g, 2.3 mL), 4-iodoanisole / 2-iodoanisole (16.68 mmol), $Pd(OAc)_2$ (1.67 mmol, 0.37 g), glycine (6.66 mmol, 0.5 g), AgTFA (25 mmol, 5.52 g), acetic acid (150 mL) and water (16 mL). The reaction mixture was allowed to stir at ambient temperature for 30 minutes and after that it was heated to 90 °C for 36 hours. **Work-up:** After completion of the reaction, it was cooled to room temperature and acetic acid was removed on rotatory evaporator. To the reaction mixture, added 100 mL water and 50 mL ethyl acetate and was filtered to remove the suspended solid. Organic layer (top layer) was separated with the help of a separating funnel and dried over anhydrous magnesium sulfate, further ethyl acetate was removed. Pure product was isolated using flash column chromatography with 2% ethyl acetate in hexane.

Synthesis of 2-(4-methoxybenzyl)benzaldehyde (9a)

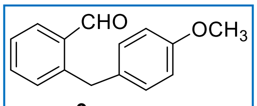

9a

Following **GP 3:** 2-methyl benzaldehyde **7** (20 mmol, 2.4 g, 2.32 mL), 4-iodoanisole (16.68 mmol, 3.9 g), $Pd(OAc)_2$ (1.668 mmol, 0.37 g), glycine (6.66 mmol, 0.5 g), AgTFA (25 mmol, 5.52 g), acetic acid (150 mL) and water (17 mL) afford the product **9a**.

Yellow oil; 2.9 g; 65% yield; R_f = 0.62 (hexane : ethyl acetate = 9.4 : 0.6).

^{1}H NMR (500 MHz, $CDCl_3$): δ = 10.25 (s, 1H, CHO), 7.86-7.84 (dd, J = 8.0, 1.5 Hz, 1H), 7.53-7.50 (dt, J = 8.0, 2.0 Hz, 1H), 7.42-7.38 (m, 1H), 7.25 (d, J = 7.5 Hz, 1H, merged with $CHCl_3$

residual peak), 7.07–7.04 (m, 2H), 6.83–6.80 (m, 2H), 4.38 (s, 2H, CH_2), 3.77 (s, 3H, OCH_3) ppm.

^{13}C NMR (126 MHz, $CDCl_3$): δ = 192.6, 158.2, 143.6, 134.0, 133.9, 132.5, 132.0, 131.6, 129.9, 127.0, 114.1, 55.4, 37.3 ppm.

The spectroscopic data are in agreement with the literature.[S3].

Synthesis of 2-(2-methoxybenzyl)benzaldehyde (9b)

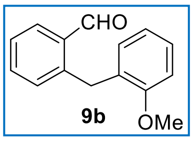

Following **GP 3:** 2-methyl benzaldehyde **7** (20 mmol, 2.4 g, 2.3 mL), 2-iodoanisole (16.68 mmol, 3.9 g), $Pd(OAc)_2$ (1.67 mmol, 0.37 g), glycine (6.66 mmol, 0.5 g), AgTFA (25 mmol, 5.5 g), acetic acid (150 mL) and water (17 mL) afford the product.

Yellow oil; 1.82 g; 40% yield; R_f = 0.64 (hexane : ethyl acetate = 9.4 : 0.6).

^{1}H NMR (500 MHz, $CDCl_3$): δ = 10.27 (s, 1H, CHO), 7.80–7.79 (dd, J = 7.5, 1.0 Hz, 1H), 7.40–7.38 (dt, J = 7.5, 1.5 Hz, 1H), 7.29–7.26 (m, 1H), 7.13–7.11 (m, 2H), 6.87–6.85 (m, 1H), 6.79–6.76 (m, 2H), 4.31 (s, 2H, CH_2), 3.72 (s, 3H, OCH_3) ppm.

^{13}C NMR (126 MHz, $CDCl_3$): δ = 192.6, 157.1, 143.5, 134.2, 134.0, 131.5, 130.4, 130.2, 128.9, 127.9, 126.8, 120.8, 110.5, 55.4, 32.0 ppm.

The spectroscopic data are in agreement with the literature.[S3].

General procedure for the synthesis of anthracene from substituted 2-benzylbenzaldehyde with slight modification (GP 4)[S4]

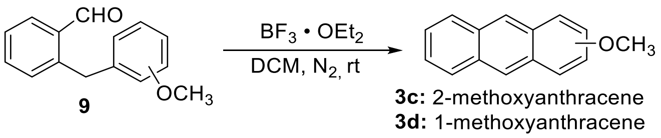

To a 25 mL two neck round bottom flask under nitrogen, substituted 2-benzylbenzaldehyde **9** (1.43 mmol), dry dichloromethane (10 mL) and $BF_3 \cdot OEt_2$ (0.28 mmol, 42 mg) was added and stirred at room temperature for 10 minutes. **Work-up and isolation of the product:** After completion of the reaction 10 mL water was added dropwise. The dichloromethane layer was separated and dried over anhydrous magnesium sulfate and concentrated. To the reaction mixture 10 mL methanol was added and heated to 40 °C. The precipitated solid was filtered and dried to obtain the product.

Synthesis of 2-methoxyanthracene (3c)

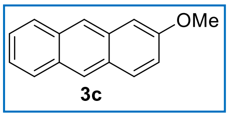

Following **GP 4:** 2-(4-methoxybenzyl)benzaldehyde **9a** (1.78 mmol, 403 mg), dry dichloromethane (12 mL) and $BF_3 \cdot OEt_2$ (0.348 mmol, 52 mg) to afford the product.

Off-white solid; 120 mg; 32% yield; M.p.: 176–177 ºC [Lit[S5] 175–177 ºC]; R_f = 0.54 (hexane : ethyl acetate = 9.8 : 0.2).

^{1}H NMR (500 MHz, $CDCl_3$): δ = 8.34 (s, 1H), 8.27 (s, 1H), 7.97–7.93 (m, 2H), 7.90–7.88 (dd, J = 9.5, 1.0 Hz, 1H), 7.46–7.43 (m, 1H), 7.42–7.38 (m, 1H), 7.20 (d, J = 2.5 Hz, 1H), 7.17–7.15 (dd, J = 9.5, 2.5 Hz, 1H), 3.97 (s, 3H, OCH_3) ppm.

^{13}C NMR (126 MHz, $CDCl_3$): δ = 157.3, 132.8, 132.3, 130.5, 123.0, 128.4, 128.3, 127.7, 126.4, 125.7, 124.6, 124.3, 120.7, 103.7, 55.4 ppm.

The spectroscopic data are in agreement with the literature.[S5].

Synthesis of 1',4-dimethoxy-1,9'-bianthracene (3f)

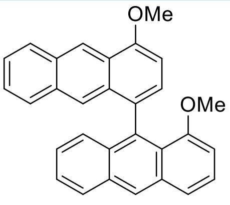

3f

Following **GP 4:** 2-(2-methoxybenzyl)benzaldehyde **9b** (1.78 mmol, 403 mg), dry DCM (12 mL) and $BF_3 \cdot OEt_2$ (0.348 mmol, 52 mg) to afford the product.

Light yellow solid; 135 mg; 36% yield; M.p.: 262–264 ºC; R_f = 0.42 (hexane : ethyl acetate = 9.6 : 0.4).

^{1}H NMR (500 MHz, $CDCl_3$): δ = 9.06 (s, 1H), 9.00 (s, 1H), 8.15 (d, J = 8.5 Hz, 1H), 8.05 (d, J = 9.0 Hz, 1H), 7.57 (s, 1H), 7.49–7.48 (m, 1H), 7.47–7.46 (m, 1H), 7.45–7.42 (m, 1H), 7.39 (s, 1H), 7.37 (s, 1H), 7.28–7.27 (m, 1H), 7.25–7.20 (m, 1H), 7.13–7.10 (m, 1H), 7.07–7.04 (m, 1H), 6.95 (d, J = 7.5 Hz, 1H), 6.73 (d, J = 7.0 Hz, 1H), 4.21 (s, 3H, OCH_3), 4.14 (s, 3H, OCH_3) ppm.

^{13}C NMR (126 MHz, $CDCl_3$): δ = 155.7, 155.6, 134.9, 132.9, 132.6, 132.1, 132.0, 131.3, 131.2, 129.2, 129.1, 128.7, 128.5, 128.4, 127.1, 125.9, 125.6, 125.5, 125.4, 125.3, 125.2, 125.1 (2C), 121.6, 121.4, 119.8, 101.7, 101.6, 55.9 (2C, OCH_3) ppm.

IR (neat): 3055, 3007, 2955, 2934, 2909, 2834, 1622, 1567, 1544, 1457, 1437, 1375, 1318, 1267, 1241, 1213, 1179, 1143, 1103, 1079, 1049, 1029, 971, 875, 834, 742, 664, 640, 623 cm^{-1}.

General procedure for the methylation of 1-hydroxyanthracene-9,10-dione (10)/1,4-dihydroxyanthracene-9,10-dione (12) using dimethyl sulfate (GP5) [S6]

Reaction mixture of 1-hydroxyanthracene-9,10-dione (**10**))/1,4-dihydroxyanthracene-9,10-dione (**12**) (1 equiv.), tetrabutylammonium bromide (0.5 equiv. for **10** and 1 equiv. for **12**), KOH (3 equiv. for **10** and 6 equiv. for **12**) and acetone (1.6 times volume of **10** or **12** in mL) was

stirred at room temperature. Dimethyl sulfate (2.5 equiv. for **10** and 5.0 equiv. for **12**) was added dropwise using a pressure-equalizing dropping funnels (***Note: as pressure develops, addition should be slow***). After complete addition, reaction mass was stirred for 24 h at room temperature. **Work-up and isolation:** After completion of the reaction, acetone was removed on rotavapor. Added demineralised water (2 times volume of **10** or **12** in mL) and stirred the reaction mass for 15 minutes. Maintained pH 13-14 with 10 N aq. KOH solution and stirred for 15 minutes. Precipitated solid was filtered through a Büchner funnel and washed with demineralised water till filtrate shows a neutral pH and was dried at room temperature to obtain the pure product.

Synthesis of 1-methoxyanthracene-9,10-dione (11)

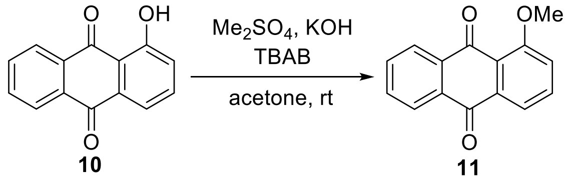

Following **GP 5**:[S6] 1-Hydroxyanthracene-9,10-dione **10** (6.72 g, 30 mmol, 1 equiv.), tetrabutylammonium bromide (4.83 g, 15 mmol, 0.5 equiv.), KOH (5 g, 90 mmol, 3 equiv.), acetone (48 mL), dimethyl sulfate (9.45 g, 7 mL, 75 mmol, 2.5 equiv.) to afford **11.**

Yellow solid; 6.85 g; 96% yield; M.p.: 167–168 °C [Lit[S7] 168–170 °C]**;** R_f = 0.31 (hexane : ethyl acetate = 1 : 1).

^{1}H NMR (500 MHz, $CDCl_3$): δ = 8.25 (d, J = 7.5 Hz, 1H), 8.20 (d, J = 7.5 Hz, 1H), 7.94 (d, J = 7.5 Hz, 1H), 7.79–7.74 (m, 1H), 7.73–7.65 (m, 2H), 7.33 (d, J = 8.5 Hz, 1H), 4.03 (s, 3H, OCH_3) ppm.

^{13}C NMR (126 MHz, $CDCl_3$): δ = 183.5, 182.6, 160.5, 135.8, 135.1, 134.4, 133.4, 132.6, 127.3, 126.7, 121.6, 119.9, 118.1, 56.7 ppm.

The spectroscopic data are in agreement with the literature.[S7].

General procedure for the synthesis of anthracenes using zinc and acetic acid (GP6)[S8]

To a 1 L single neck round bottom flask placed on a sand bath, acetic acid (800 mL) was added and degassed with nitrogen for 10 minutes. Under nitrogen environment added 27.6 mmol of either 1-methoxyanthracene-9,10-dione (**11**) or 1,4-dimethoxyanthracene-9,10-dione (**12a**) and zinc dust (47 g, 720 mmol). The reaction mixture was refluxed for 24 h under nitrogen environment. **Work-up and isolation of the product:** After 24 h reflux, the reaction mixture was cooled down to room temperature. Acetic acid was removed on a rotary evaporator under reduced pressure. Then ethyl acetate (200 mL) was added and stirred for 10 minutes. The precipitated solid was removed by filtration. The solid was washed twice with 100 mL ethyl acetate. The solid residue was discarded, and the ethyl acetate filtrates were combined. Further, ethyl acetate was removed on rotavapor and loaded on silica. The product from the crude was isolated by using automated flash chromatography (gradient elution: 100% hexane to 4% ethyl acetate in hexane).

Synthesis of 1-methoxyanthracene (3d)

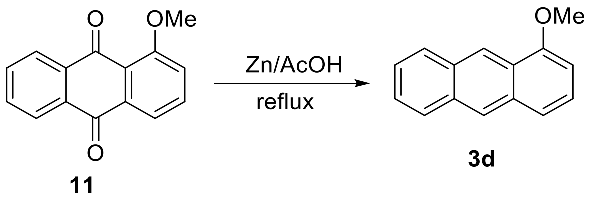

Following **GP 6**: [S8] 1-methoxyanthracene-9,10-dione **11** (6 g, 25.2 mmol), acetic acid (730 mL) and zinc (43 g, 658 mmol) afford the product.

Light yellow green solid; 2.24 g, 42% yield; M.p.; 71–72 ºC [Lit[S9] 70–71 ºC]; R_f = 0.82 (hexane : ethyl acetate = 9.6 : 0.4).

^{1}H NMR (500 MHz, $CDCl_3$): *δ* = 8.87 (s, 1H), 8.38 (s, 1H), 8.08–8.05 (m, 1H), 8.02–7.99 (m, 1H), 7.61–7.59 (dt, *J* = 1.0, 0.5 Hz, 1H), 7.51–7.46 (m, 2H), 7.39–7.37 (dd, *J* = 8.5, 7.5 Hz, 1H), 6.74 (d, *J* = 7.5 Hz, 1H), 4.09 (s, 3H, OCH_3) ppm.

^{13}C NMR (126 MHz, $CDCl_3$): *δ* = 155.6, 132.7, 132.1, 131.4, 128.9, 128.0, 125.8, 125.4, 125.2, 125.1, 121.2, 120.6, 101.7, 55.6 ppm.

The spectroscopic data are in agreement with the literature.[S9].

Synthesis of 1-iodo-8-methoxy-9,10-dihydro-9,10-[1,2]benzenoanthracene (*syn*-5e) and 1-iodo-5-methoxy-9,10-dihydro-9,10-[1,2]benzenoanthracene (*anti*-5e)

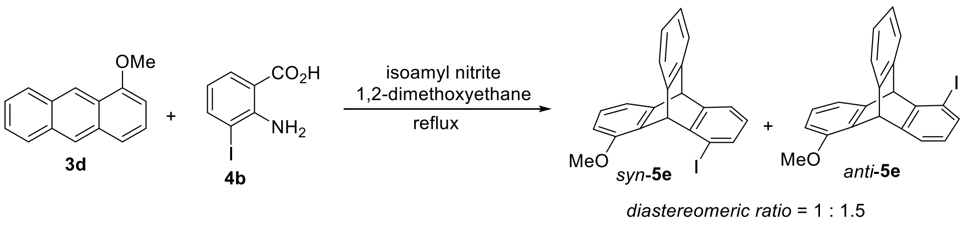

Following **GP 1**: 1-methoxyanthracene **3d** (8.39 mmol, 1.74 g), 1,2-dimethoxyethane (18.5 mL), isoamyl nitrite (11.1 mmol, 0.13 g), 2-amino-3-iodobenzoic acid **4b** (14.17 mmol, 0.37 g) in 1,2-dimethoxyethane (13 mL), isoamyl nitrite (11 mmol, 0.13 g) afforded the crude product (mixture of diastereomers in the ratio of 1:1.5).

Colourless solid, 417 mg (*syn* + *anti*); 12% yield; R_f = 0.26 (hexane : ethyl acetate = 9.6 : 0.4).

^{1}H NMR (400 MHz, $CDCl_3$): *δ* = 7.55–7.53 (m, 1H), 7.51–7.48 (m, 1H), 7.47–7.42 (m, 4H), 7.38 (d, *J* = 7.2 Hz, 1H), 7.34 (d, *J* = 7.2 Hz, 1H), 7.16 (d, *J* = 7.2 Hz, 1H), 7.09–6.99 (m, 7H), 6.72–6.68 (m, 2H), 6.66–6.63 (m, 2H), 6.32 (s, 1H, CH), 5.92 (s, 1H, CH), 5.82 (s, 1H, CH), 5.43 (s, 1H, CH), 3.92 (s, 3H, OCH_3), 3.87 (s, 3H, OCH_3) ppm.

^{13}C NMR (101 MHz, $CDCl_3$): *δ* = 154.9, 154.6, 149.0, 148.9, 147.7, 147.3 (2C), 146.7, 145.6, 145.2, 144.9, 144.6, 135.0, 134.8, 132.8, 132.4, 127.0, 126.9, 126.5, 126.4, 125.5, 125.4 (2C), 125.3, 124.3, 124.1, 123.9, 123.8, 123.7, 116.9, 116.4, 108.9, 108.7, 94.2, 94.1, 58.3, 56.1, 55.7, 55.0, 51.2, 47.7 ppm.

Synthesis of 1,4-dimethoxyanthracene-9,10-dione (12a)

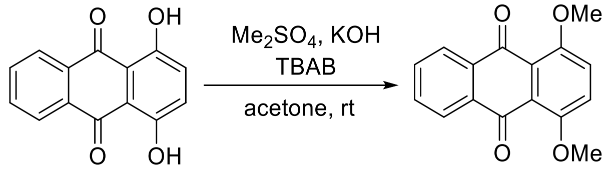

12 **12a**

Following **GP 5**: [S6] 1,4-dihydroxyanthracene-9,10-dione **12** (7.2 g, 30 mmol, 1 equiv.), tetrabutylammonium bromide (9.66 g, 30 mmol, 1 equiv.), KOH (10 g, 180 mmol, 6 equiv.), acetone (48 mL), dimethyl sulfate (19 g, 14 mL, 150 mmol, 5 equiv.) to afford **12a.**

Yellow solid; 7.9 g, 98% yield; M.p.: 171–172 ºC [Lit[S10] 170−174ºC]; R_f = 0.15 (hexane : ethyl acetate = 1 : 1).

^{1}H NMR (500 MHz, $CDCl_3$): δ = 8.15–8.14 (dd, J = 3.5, 2.0 Hz, 1H), 7.70–7.68 (m, 1H), 7.32 (s, 1H), 3.98 (s, 3H, OCH_3) ppm.

^{13}C NMR (126 MHz, $CDCl_3$): δ = 183.5, 154.2, 134.3, 133.4, 126.5, 123.1, 120.3, 57.1 ppm.

The spectroscopic data are in agreement with the literature.[S7,S10]

Synthesis of 1,4-dimethoxyanthracene (3e)

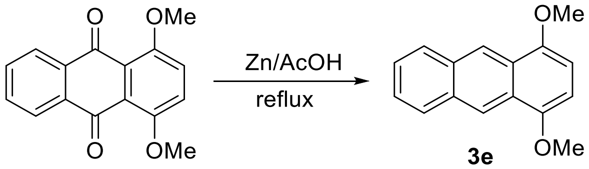

12a **3e**

Following **GP 6**: Acetic acid (800 mL), 1,4-dimethoxyanthracene-9,10-dione **12a** (7.4 g, 27.6 mmol), zinc dust (47 g, 720 mmol) to afford **3e**.

Yellow green solid; 4.6 g, 70% yield; M.p.: 135–136 ºC [Lit[S9] 134–136 ºC]; R_f = 0.71 (hexane : ethyl acetate = 9.6 : 0.4).

^{1}H NMR (500 MHz, $CDCl_3$): δ = 8.79 (s, 1H), 8.05–8.04 (dd, J = 6.5, 3.0 Hz, 1H), 7.49–7.47 (dd, J = 7.0, 3.5 Hz, 1H), 6.60 (s, 1H), 4.04 (s, 3H, –OCH_3) ppm.

^{13}C NMR (126 MHz, $CDCl_3$): δ = 149.6, 131.6, 128.7, 125.7, 120.9, 101.0, 55.8 ppm.

The spectroscopic data are in agreement with the literature.[S9].

Synthesis of 5-iodo-1,4-dimethoxy-9,10-dihydro-9,10-[1,2]benzenoanthracene (5f)

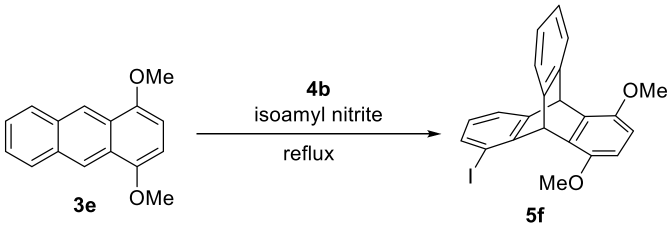

3e **5f**

Batch Procedure:
Following **GP 1**: 1,4-dimethoxyanthracene **3e** (2 g, 8.39 mmol), 1,2-dimethoxyethane (19 mL), isoamyl nitrite (11 mmol, 0.13 g), 2-amino-3-iodobenzoic acid **4b** (0.37 g, 14.2 mmol), in 1,2-dimethoxyethane (13 mL), isoamyl nitrite (11 mmol, 0.13 g) afforded product **5f** by using flash chromatography (gradient elution: 100% hexane to 30% ethyl acetate in hexane).

Beige solid; 407 mg, 11% yield; M.p.: 264–265 °C; R_f = 0.18 (hexane : ethyl acetate = 9.6 : 0.4).

^{1}H NMR (400 MHz, $CDCl_3$): δ = 7.50–7.47 (m, 1H), 7.42–7.39 (m, 2H), 7.35–7.27 (m, 1H), 7.04–6.98 (m, 2H), 6.70–6.65 (m, 1H), 6.57-6.51 (m, 2H), 6.23 (d, J = 4.0 Hz, 1H, CH), 5.85 (d, J = 4.4 Hz, 1H, CH), 3.86 (d, J = 4.0 Hz, 3H, OCH_3), 3.81 (d, J = 4.0 Hz, 3H, OCH_3) ppm.

^{13}C NMR (101 MHz, $CDCl_3$): δ = 149.4, 149.3, 149.1, 147.8, 145.6, 145.0, 135.1, 134.9, 134.8, 126.9, 125.4, 125.3, 124.4, 123.9, 109.7, 109.2, 94.2, 57.1, 56.5, 51.7, 48.2 ppm.

HRMS (ESI) calcd for $C_{22}H_{18}O_2I$ $[M+H]^+$ 441.0351, found 441.0353.

IR (neat): 3070, 2991, 2956, 2926, 2850 2833, 1558, 1492, 1456, 1435, 1327, 1253, 1226, 1188, 1159, 1068, 948, 785, 771, 758, 734, 727, 709, 671, 648, 570 cm^{-1}.

Flow Procedure:

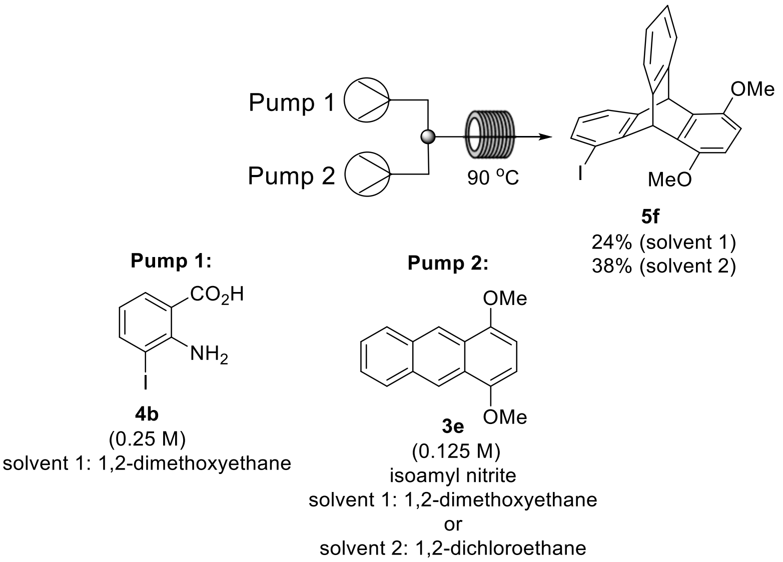

Scheme S1: Design of experiments set-up. Flow rate: 2 x 0.2 mL/min to achieve 2.5 minutes residence time. Column: PTFE i.d. 0.8 mm, CV: 1 mL submerged in hot bath at 90 °C.

The 2-amino-3-iodobenzoic acid **4b** (0.33 g, 1.25 mmol, 2 equiv.) was dissolved in 1,2-dimethoxyethane (5 mL, 0.25 M) and transferred to a syringe. 1,4-dimethoxyanthracene **3e** (0.15 g, 0.63 mmol, 1 equiv.) was dissolved in either 1,2-dimethoxyethane or 1,2-dichloroethane (5 mL, 0.125 M) then added isoamyl nitrite (0.17 mL, 1.25 mmol, 2 equiv.) and was transferred to a syringe. Both syringes pumped at 0.2 mL/min using syringe pumps through a T-piece mixer and to PTFE tubing (i.d. 0.8 mm, volume: 1 mL, residence time: 2.5 min). The PTFE coil was submerged in a hot water bath at 90 °C. The first column volume of reactant was discarded, and the following three column volumes were collected and

concentrated in vacuo at 40 °C. Following **GP 1**, the product from the crude mixture was isolated by using flash chromatography (gradient elution: 100% hexane to 30% ethyl acetate in hexane) to afford compound **5f** as a cream solid (solvent 1: 66 mg, 24% yield; solvent 2: 104 mg, 38% yield).

Synthesis of 5-iodo-9,10-dihydro-9,10-[1,2]benzenoanthracene-1,4-diol (13)

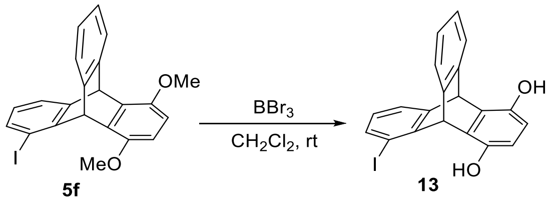

5f **13**

Following the literature procedure,[S11] compound **13** was prepared with a slight modification. To a 25 mL single neck RBF added 5-iodo-1,4-dimethoxy-9,10-dihydro-9,10-[1,2]benzeno-anthracene **5f** (0.25 mmol, 110 mg) and dry dichloromethane (4 mL) at room temperature under nitrogen. It was stirred to obtain a clear solution. Added BBr_3 solution (1 M in dichloromethane; 0.5 mmol, 0.5 mL, 124 mg) dropwise under nitrogen atmosphere. After complete addition the reaction mixture was stirred at room temperature for 24 h. **Work-up and isolation of the product:** After completion of the reaction, added 12 mL water dropwise which showed the perception of solid. After complete addition of water, reaction mass was stirred for 15 min. Further, dichloromethane present in the solution mixture was removed on rotavapor. Again 10 mL water was added and stirred for 15 min. The precipitated solid was filtered under suction and washed twice with 10 mL water and dried.

Off white solid; 90 mg, 87% yield; M.p.: 297–298 °C; R_f = 0.54 (hexane : ethyl acetate = 8 : 2).

^{1}H NMR (500 MHz, $CDCl_3$): δ = 7.50–7.46 (m, 1H), 7.44–7.40 (m, 2H), 7.34 (d, J = 7.5 Hz, 1H), 7.05–7.01 (m, 2H), 6.70 (t, J = 7.5 Hz, 1H), 6.39–6.32 (m, 2H), 6.13 (s, 1H, CH), 5.78 (s, 1H, CH), 4.51 (brs, 1H, OH), 4.44 (brs, 1H, OH) ppm.

^{13}C NMR (126 MHz, $CDCl_3$): δ = 148.9, 147.3, 145.1, 144.8, 144.5, 144.4, 135.0, 133.1, 132.7, 127.1, 125.7, 125.6, 124.4, 124.0 (2C), 113.8, 113.7, 94.2, 51.9, 48.3 ppm.

HRMS (EI) Calcd for $C_{20}H_{13}O_2I$ = 411.99547 and Found = 411.9955.

IR (neat): 3392, 3265 (broad), 3126, 2976, 2804, 1579, 1523, 1489, 1421, 1396, 1363, 1340, 1261, 1242, 1211, 1091, 991, 802, 723, 644, 532 cm^{-1}.

Synthesis of (5-iodo-9,10-dihydro-9,10-[1,2]benzenoanthracene-1,4-diyl (1S,1'S,4S,4'S)-bis(4,7,7-trimethyl-3-oxo-2-oxabicyclo[2.2.1]heptane-1-carboxylate) (16)

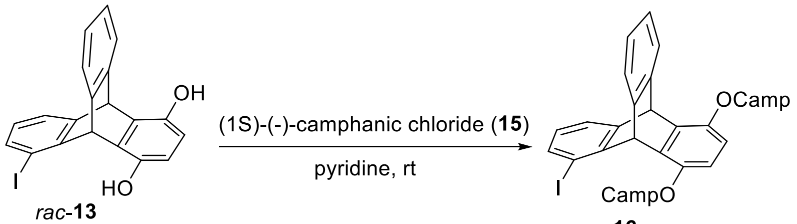

rac-**13** **16**

To a 5 mL glass vial, 5-iodo-9,10-dihydro-9,10-[1,2]benzenoanthracene-1,4-diol *rac*-**13** (0.145 mmol, 60 mg), (1*S*)-(-)-camphanic chloride **15** (0.347 mmol, 75 mg) and pyridine (1 mL) was added and kept it as such without stirring for 2 days. **Work-up and isolation of the product:** After completion of the reaction, reaction mass was transferred to a 25 mL separating funnel using 10 mL dichloromethane. Added 10 mL demineralised water to the separating funnel. Shaked well and organic layer (bottom layer) was separated. Aqueous layer was washed two times with 10 mL dichloromethane and all collected dichloromethane fractions were combined. Further, the collected dichloromethane fraction was washed with 1 M HCl solution till the pH of the aqueous layer showed acidic (2-3). Next, collected dichloromethane fraction was washed with 10 mL demineralised water two times. Finally, it was dried with anhydrous $MgSO_4$ and removed on rotavapor to obtain the diastereomeric product. White solid; 100 mg; 89% yield.

Separation of the diastereomers using preparative TLC:
Compound **16** (diastereomeric product) was dissolved in 0.5 mL dichloromethane and loaded on TLC plates. The TLC plates were dipped into the solvent mixture of $CHCl_3$ and CH_3CN in the ratio of 9.6 : 0.4, The two spots observed were isolated and collected separately. The compound from silica was isolated using dichloromethane and dried on rotavapor.

Diastereomer 1 (Top spot isolated compound from preparative TLC) (–)–**16**:
White solid; 33 mg; 29% yield; M.p.: 331–332 °C; R_f = 0.49 ($CHCl_3$: CH_3CN = 9.6 : 0.4).

^{1}H NMR (500 MHz, $CDCl_3$): δ = 7.46–7.44 (dd, *J* = 8.0, 1.0 Hz, 1H), 7.43–7.41 (m, 1H), 7.36–7.32 (m, 1H), 7.27 (d, *J* = 7.0 Hz, 1H), 7.08–7.04 (m, 2H), 6.88–6.86 (dd, *J* = 11.5, 8.5 Hz, 2H), 6.74–6.72 (dd, *J* = 8.0, 7.5 Hz, 1H), 5.93 (s, 1H), 5.44 (s, 1H), 2.82–2.77 (m, 1H), 2.71–2.64 (m, 1H), 2.46–2.41 (m, 1H), 2.37–2.32 (m, 1H), 2.10–2.03 (m, 2H), 1.89–1.82 (m, 2H), 1.24 (d, *J* = 3.0 Hz, 6H, 2 x CH_3), 1.23 (s, 6H, 2 x CH_3), 1.22 (s, 6H, 2 x CH_3) ppm.

^{13}C NMR (126 MHz, $CDCl_3$): δ = 178.0, 177.9, 165.9, 165.8, 147.4, 145.7, 143.5, 143.0, 142.9, 142.7, 139.1, 138.6, 135.6, 127.6, 126.2, 126.1, 124.8, 124.3, 124.2, 119.8, 119.7, 94.4, 91.1, 91.0, 55.1 (2C), 55.0, 54.8, 52.7, 49.6, 31.6, 31.2, 29.3, 29.1, 17.3, 17.2, 17.1, 9.9 (2C) ppm.

HRMS (ES$^+$) Calcd for $C_{40}H_{38}O_8I$ = 773.1611 and Found = 773.1615.

IR (neat): 3032, 3006, 2967, 2921, 2874, 2851, 1791, 1774, 1700, 1684, 1651, 1557, 1539, 1507, 1475, 1455, 1338, 1313, 1260, 1204, 1164, 1094, 1046, 932, 750, 667 cm^{-1}.

Diastereomer 2 (Bottom spot isolated compound from TLC) (+)–**16:**
White solid; 22 mg; 19% yield; M.p.: 336–337 °C; R_f = 0.47 ($CHCl_3$: CH_3CN = 9.6 : 0.4).

^{1}H NMR (500 MHz, $CDCl_3$): δ = 7.46–7.44 (dd, *J* = 8.0, 0.5 Hz, 1H), 7.41–7.40 (m, 1H), 7.35–7.33 (m, 1H), 7.27 (d, *J* = 7.0 Hz, 1H), 7.07–7.05 (m, 2H), 6.96 (d, *J* = 9.0 Hz, 1H), 6.88 (d, *J* = 9.0 Hz, 1H), 6.74–6.73 (dd, *J* = 8.0, 7.5 Hz, 1H), 5.94, (s, 1H), 5.45 (s, 1H), 2.85–2.79 (m, 1H), 2.70–2.64 (m, 1H), 2.48–2.42 (m, 1H), 2.37–2.31 (m, 1H), 2.12–2.04 (m, 2H), 1.89–1.84 (m, 2H), 1.30 (s, 3H, CH_3), 1.25 (s, 3H, CH_3), 1.23 (d, *J* = 2.5 Hz, 6H, 2 x CH_3), 1.21 (s, 3H, CH_3), 1.19 (s, 3H, CH_3) ppm.

^{13}C NMR (126 MHz, $CDCl_3$): δ = 178.0, 177.9, 165.9, 165.7, 147.3, 145.7, 143.6, 143.1 (2C), 142.7, 139.0, 138.3, 135.7, 127.6, 126.2, 126.1, 124.6, 124.3 (2C), 119.8, 119.7, 94.4, 91.1, 91.0, 55.1 (2C), 54.9, 54.7, 52.8, 49.5, 31.4, 31.3, 29.2, 29.1, 17.4, 17.3, 17.1, 9.9 (2C) ppm.

HRMS (ES^+) Calcd for $C_{40}H_{38}O_8I$ = 773.1611 and Found = 773.1628.

IR (neat): 3062, 2961, 2919, 2850, 1789, 1771, 1651, 1559, 1539, 1491, 1474, 1458, 1261, 1208, 1159, 1095, 1050, 1017, 932, 898, 854, 798, 752, 732, 667 cm^{-1}.

Synthesis of (–)-5f and (+)-5f from (–)-16 and (+)-16 in a one-pot reaction

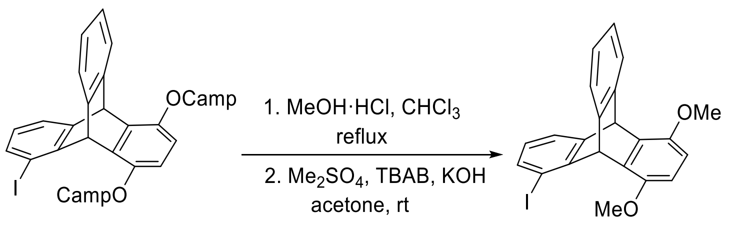

To a 10 mL single neck round bottom flask compound (–)–**16** (0.042 mmol, 33 mg) was dissolved in 3 M methanolic · HCl solution (3 mL) and $CHCl_3$ (1 mL). The reaction mixture was placed on sand bath and heated to reflux for 48 h under argon. After completion of the reaction, the solvent was removed under vacuo. 20 mg Tetrabutylammonium bromide (TBAB) was added, 0.1 mL dimethyl sulfate, 90 mg potassium hydroxide (KOH), 0.2 mL acetone and stirred at room temperature for 24 h. After completion of the reaction acetone was removed. Added 5 mL demineralised water and pH 13-14 was adjusted with aq. 10 N KOH solution and stirred for 15 minutes. The precipitated solid was filtered under suction, washed with demineralised water until filtrate showed neutral pH and was dried.

White solid; 14 mg; 74% yield. The product obtained is (–)–**5f** with $[\alpha]_D^{20}$ = − 18.57 (c, 0.28 in $CHCl_3$).

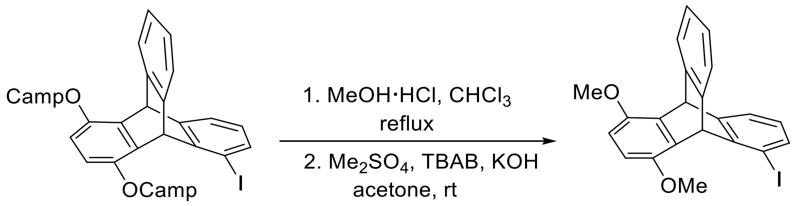

As above, (+)–**16** (0.028 mmol, 22 mg), 3 M methanolic · HCl solution (2 mL) and $CHCl_3$ (0.67 mL).
White solid; 9 mg; 72% yield. Product obtained as (+)–**5f**; $[\alpha]_D^{20}$ = + 28.88 (c, 0.18 in $CHCl_3$).

Spectroscopic data for 1-iodo-9,10-dihydro-9,10-[1,2]benzenoanthracene-13,16-dione (13a)

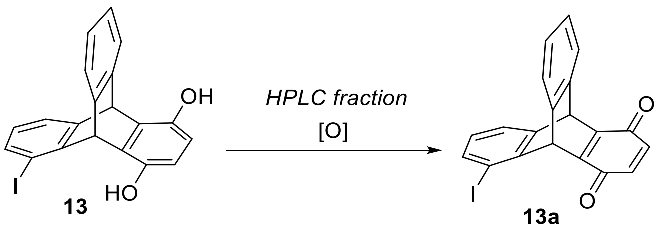

Yellow solid; (Obtained from HPLC fraction); M.p.: > 300 °C; R_f = 0.61 (hexane : ethyl acetate = 8 : 2).

^{1}H NMR (500 MHz, $CDCl_3$): δ = 7.52–7.49 (m, 1H), 7.47–7.46 (dd, J = 8.0, 1.0 Hz, 1H), 7.45–7.42 (m, 1H), 7.36 (d, J = 7.0 Hz, 1H), 7.08–7.06 (m, 2H), 6.75–6.73 (dd, J = 8.0, 7.5 Hz, 1H), 6.66–6.61 (m, 2H), 6.13 (s, 1H), 5.76 (s, 1H) ppm.

^{13}C NMR (126 MHz, $CDCl_3$): δ = 183.5, 183.2, 152.0, 151.7, 147.2, 145.3, 143.5, 142.8, 135.6, 135.4, 127.4, 126.0 (2C), 125.0, 124.6, 124.5, 94.2, 51.9, 48.1 ppm.

HRMS (EI) Calcd for $C_{20}H_{11}O_2I$ = 409.97982 and Found = 409.9806.

IR (neat): 3054, 3026, 2921, 2853, 1667, 1610, 1558, 1456, 1435, 1392, 1287, 1258, 1166, 1009, 747, 698, 650 cm^{-1}.

Synthesis of (1*S*,4*R*)-1-4-(((1*S*,4*S*)-7,7-dimethyl-2-oxobicyclo[2.2.1]heptan-1-yl)methoxy)-5-iodo-9,10-dihydro-9,10-[1,2]benzenoanthracen-1-yl)oxy)methyl)-7,7-dimethylbicyclo[2.2.1]heptan-2-one-sulfur(IV) oxide (16a)

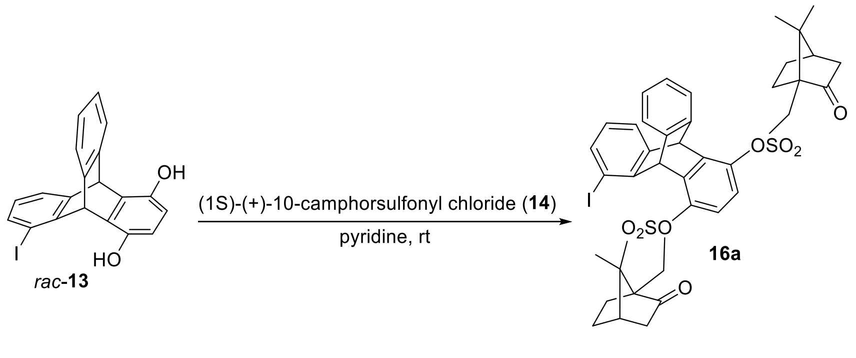

Followed the procedure of **16** [S12] compound **16a** (diastereomeric product) was prepared. 5-iodo-9,10-dihydro-9,10-[1,2]benzenoanthracene-1,4-diol *rac*–**13** (0.048 mmol, 20 mg), (1S)–(+)–10-camphorsulfonyl chloride **14** (0.115 mmol, 29 mg) and pyridine (0.33 mL).

Colourless solid; 37 mg; 92% yield; M.p.: > 300 °C; R_f = 0.43 ($CHCl_3$: CH_3CN = 9.6 : 0.4).

^{1}H NMR (500 MHz, $CDCl_3$): δ = 7.58–7.57 (m, 1H), 7.53–7.30 (m, 1H), 7.49–7.48 (dd, J = 5.0, 3.0 Hz, 1H), 7.46–7.40 (m, 5H), 7.13 (d, J = 7.5 Hz, 1H), 7.11 (d, J = 7.0 Hz, 1H), 7.08–7.02 (m, 6H), 6.76–6.71 (m, 2H), 6.27 (s, 1H, CH), 6.22 (s, 1H, CH), 5.91 (s, 1H, CH), 5.90 (s, 1H, CH), 4.08 (d, J = 15.0 Hz, 1H, CH), 4.00 (d, J = 15.0 Hz, 1H, CH), 3.93 (d, J = 4.5 Hz, 1H, CH), 3.90 (d, J = 4.0 Hz, 1H, CH), 3.45 (d, J = 14.5 Hz, 1H, CH), 3.39 (d, J = 15.0 Hz, 1H, CH), 3.27 (d, J = 10.5 Hz, 1H, CH), 3.24 (d, J = 10.5 Hz, 1H, CH), 2.66–2.57 (m, 2H, 2 x CH), 2.56–2.52 (m, 1H, CH), 2.48–2.46 (m, 2H, 2 x CH), 2.45–2.42 (m, 2H, 2 x CH), 2.18–2.15 (m, 5H, 1H + 2 x CH_2), 2.14–2.07 (m, 4H, 2 x CH_2), 2.04–2.02 (m, 2H, 2 x CH), 2.00–1.98 (m, 2H,

2 x CH), 1.87–1.76 (m, 4H, 2 x CH_2), 1.52–1.45 (m, 4H, 2 x CH_2), 1.22 (s, 3H, CH_3), 1.17 (s, 3H, CH_3), 1.16 (s, 3H, CH_3), 1.15 (s, 3H, CH_3), 0.95 (s, 3H, CH_3), 0.94 (s, 3H, CH_3), 0.91 (s, 3H, CH_3), 0.90(s, 3H, CH_3) ppm.

^{13}C NMR (126 MHz, $CDCl_3$): δ = 214.2, 214.1 (2C), 214.0, 147.4, 146.0, 145.9, 143.6, 143.5, 143.2, 143.0, 142.4, 142.3, 142.2 (2C), 141.5, 141.4, 140.9, 140.7, 135.6, 135.5, 127.6, 126.1 2C), 126.0 (2C), 125.2, 125.0, 124.9, 124.8, 124.7, 124.5, 121.0 (2C), 120.8, 120.7, 94.3, 94.2, 58.4 (2C), 58.3 (2C), 53.6, 52.9, 49.4, 48.8, 48.7, 48.5, 48.4, 48.3, 48.2, 48.1, 43.0 (3C), 42.0 (2C), 27.1, 27.0 (2C), 25.5, 25.4, 25.3, 25.2, 20.2, 20.1, 20.0 (2C), 19.9 (2C), 19.8 (2C) ppm.

Procedures for the α-tosylation of propiophenone (GP 7)[S13]
Iodine catalyst [**5a** or **5b** or *syn*–(+)–**5c** or *syn*–(–)–**5c** or *rac*–**5f** or (+)–**5f** or (–)–**5f**; 0.025 mmol], propiophenone **17** (0.25 mmol, 0.033 mL), *m*CPBA (0.75 mmol, 129.4 mg), *p*-TsOH · H_2O (0.75 mmol, 143 mg) in acetonitrile (3 mL) was added to a 25 mL round bottom flask and stirred at room temperature for 48 h. After completion of the reaction acetonitrile was removed on rotavapor and the crude mixture was washed with sat. aq. $NaHCO_3$ solution and sat. aq. $Na_2S_2O_3$ solution and extracted three times with 10 mL dichloromethane. The combined organic layers were dried over anhydrous $MgSO_4$, filtered, concentrated under reduced pressure. The crude products were purified by flash chromatography on silica gel (hexane : ethyl acetate = 90 : 10) to afford the desired pure product **18**.
Procedure to recover the catalyst: After completion of the reaction, acetonitrile was removed on rotavapor. Added sat. aq. $NaHCO_3$ solution to dissolve *m*-Chlorobenzoic acid and undissolved salts. The aqueous layer was washed three times with 10 mL dichloromethane. The combined organic layers were dried over anhydrous $MgSO_4$, filtered, concentrated under reduced pressure. The desired catalyst was recovered using flash chromatography (gradient elution: 100% hexane to 30% ethyl acetate in hexane); about 50% of the catalysts **5a** and **5b** were recovered.

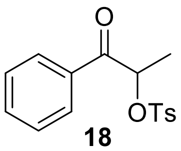

18

1-Oxo-1-phenylpropan-2-yl 4-methylbenzenesulfonate **18:**[S13] Colourless oil.

^{1}H NMR (400 MHz, $CDCl_3$): δ = 7.87–7.85 (dd, *J* = 8.4, 1.2 Hz, 2H), 7.74 (d, *J* = 8.8 Hz, 2H), 7.60–7.56 (m, 1H), 7.46–7.42 (m, 2H), 7.27–7.24 (m, 2H), 5.78 (q, *J* = 6.8 Hz, 1H, CH), 2.40 (s, 3H, CH_3), 1.58 (d, *J* = 7.2 Hz, 3H, CH_3) ppm.

^{13}C NMR (101 MHz, $CDCl_3$): δ = 194.9, 145.1, 134.0, 133.8, 133.5, 129.9, 128.9, 128.8, 128.0, 77.5, 21.7, 18.8 ppm.

The spectroscopic data are in agreement with the literature.[S13].

HPLC analysis (YMC Chiral Amylose-C, hexane/i-PrOH = 85/15, flow rate 0.7 mL/min, λ = 254 nm, 25 °C); t_{R1} = 15.215 min, t_{R2} = 16.894 min. Configuration of the product was determined from the reported HPLC data[S13b-c]

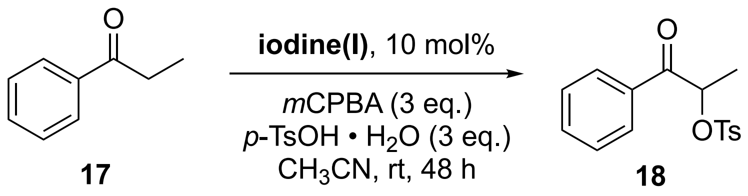

5a: 62%
5b: 76%
syn-(+)-**5c**: 58%, 2% *ee* (*R*)
syn-(–)-**5c**: 61%, 4% *ee* (*S*)
rac-**5f**: 74%
(+)-**5f**: 71%, 3% ee (*S*)
(–)-**5f**: 68%, 3% ee (*R*)

Procedure for the synthesis of 19[S14-S15]

2-Naphthol (1 g, 7 mmol) and *N*-(hydroxymethyl)benzamide (1.04 g, 6.9 mmol) were dissolved in anhydrous ethanol (100 mL). Concentrated sulfuric acid (10 mL) was added dropwise and the reaction mixture was stirred for 7 hours at 50 °C. The reaction mixture was cooled to room temperature, washed with 1 M NaOH solution (50 mL) and extracted by ethyl acetate. The combined organic layers were dried with $MgSO_4$, filtered, and concentrated under reduced pressure. The residue was recrystallized (ethyl acetate/petroleum ether) to afford the desired product as a solid **19** (1.33 g, 72% yield).

N-((2-Hydroxynaphthalen-1-yl)methyl)benzamide **19**:

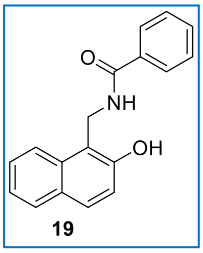

Brown solid; M.p.: 176–177 °C [Lit[S15] 173−175 ºC]; ; R_f = 0.18 (hexane: ethyl acetate = 8 : 2).

^{1}H NMR (400 MHz, $CDCl_3$): δ = 10.25 (bs, 1H, NH), 7.95 (d, J = 8.4 Hz, 1H), 7.82 (d, J = 8.0 Hz, 1H), 7.78–7.75 (m, 3H), 7.54–7.47 (m, 2H), 7.40–7.38 (m, 2H), 7.36–7.34 (m, 1H), 7.28 (d, J = 8.8 Hz, 1H), 5.02 (d, J = 6.4 Hz, 2H, CH_2), 4.85 (bs, 1H, –OH) ppm.

^{13}C NMR (101 MHz, $CDCl_3$): δ = 170.2, 154.6, 133.2, 132.9, 132.4, 130.4, 129.2, 129.1, 128.8, 127.3, 127.1, 123.2, 121.3, 120.7, 115.9, 35.8 ppm.

The spectroscopic data are in agreement with the literature.[S15].

Procedures for dearomatizing cyclization of 19[S14-15]

To a solution of amide **19** (0.03 g, 0.11 mmol, 1 equiv.) in HFIP (1 mL) at room temperature the iodine reagent [10 mol% of (+)-**5f** or (–)-**5f** or (+)-**16** or (–)-**16**] and *m*CPBA (0.03 g, 0.18 mmol, 1.6 equiv.) was added. The reaction mixture was stirred for 16 h. After completion of the reaction, solvent was removed on rotavapor. The resulting residue was purified by column chromatography (hexane/ethyl acetate = 8/2) to afford **20**.

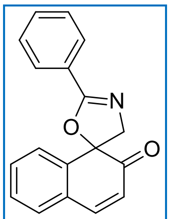

20

2'-Phenyl-2H,4'H-spiro[naphthalene-1,5'-oxazol]-2-one **20**: Yellow oil; R_f = 0.16 (hexane: ethyl acetate = 8 : 2).

^{1}H NMR (400 MHz, CDCl$_3$): δ = 8.07 (d, J = 7.2 Hz, 2H), 7.56–7.52 (m, 1H), 7.50–7.48 (m, 2H), 7.47–7.46 (m, 1H), 7.45–7.43 (m, 1H), 7.42–7.39 (m, 1H), 7.38–7.35 (m, 2H), 6.22 (d, J = 10.0 Hz, 1H), 4.48 (d, J = 14.8 Hz, 1H, CH), 4.02 (d, J = 14.8 Hz 1H, CH) ppm.

^{13}C NMR (101 MHz, CDCl$_3$): δ = 197.9, 164.4, 145.9, 142.4, 132.0, 131.1, 129.8, 129.1, 129.0, 128.9, 128.7, 127.0, 125.7, 123.8, 86.6, 69.9 ppm.

The spectroscopic data are in agreement with the literature.[S14-15].

HPLC analysis (Chiralcel OD-H column, hexane/i-PrOH = 80/20 or 88/12, flow rate 1.0 mL/min, λ = 254 nm, 25 °C); t_{R1} = 9.393 min, t_{R2} = 13.008 min. Racemic compound of **20** was prepared using 2-iodoanisole according to the literature procedure and configuration of the product was determined from the reported HPLC data.[S15]

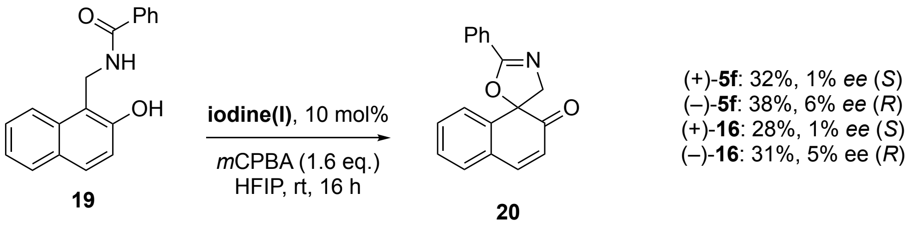

Procedure for the dearomative spirolactonization of 21

Procedure for the synthesis of 3,4-dihydro-2*H*-benzo[h]chromen-2-one (21a)[S16]
To a 250 mL single neck RBF added 1-naphthol (5 g, 34.7 mmol), Amberlyst® 15-H (3.5 g), acrylic acid (4.98 g, 4.74 mL, 69.2 mmol), toluene (120 mL) and placed on sand bath in reflux mode. The reaction mixture was heated to reflux for 12 h. **Work-up and isolation of the product:** After 12 h reflux, reaction mass was cooled to room temperature and the catalyst was filtered off over a short silica pad, then washed with hot toluene. Toluene was removed on rotavapor to obtained crude mass which was purified by using automated flash column chromatography (gradient elution: hexane/ethyl acetate = 100/0 to 85/15).

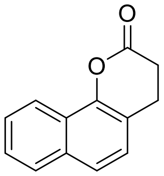

21a

3,4-Dihydro-2H-benzo[h]chromen-2-one **21a**:

White solid; 1.12 g; 17% yield; M.p.: 75–76 °C [Lit[S18] 76−77 ºC]; R_f = 0.58 (hexane : ethyl acetate = 7 : 3).

^{1}H NMR (500 MHz, $CDCl_3$): *δ* = 8.15 (d, *J* = 8.0 Hz, 1H), 7.74 (d, *J* = 7.5 Hz, 1H), 7.51 (d, *J* = 8.0 Hz, 1H), 7.48–7.41 (m, 2H), 7.18 (d, *J* = 8.5 Hz, 1H), 3.08–3.05 (m, 2H, CH_2), 2.83–2.80 (m, 2H, CH_2) ppm.

^{13}C NMR (126 MHz, $CDCl_3$): *δ* = 168.5, 146.8, 133.4, 127.6, 126.6, 126.5, 125.4, 124.0, 123.7, 120.9, 117.2, 29.2, 23.9 ppm.

The spectroscopic data are in agreement with the literature.[S18].

Procedure for the synthesis of 3-(1-hydroxynaphthalen-2-yl)propanoic acid (21)[S17]
To a 50 mL single neck round bottom flask, 3,4-dihydro-2*H*-benzo[h]chromen-2-one **21a** (0.25 g, 1.26 mmol), dry THF (9 mL) was added and stirred to get a clear solution. To this clear solution, added dropwise 4.5 mL 1 N aqueous solution of LiOH. After complete addition, the colour of the reaction mass changes to red-brown solution which was stirred vigorously at room temperature for 12 h. After completion of the reaction, pH of the solution was adjusted to 3 with an aqueous 2 N HCl solution. After that, the mixture was extracted with ethyl acetate. The obtained ethyl acetate was washed with brine, dried over anhydrous magnesium sulfate, and concentrated under reduced pressure to afford crude compound, which was further purified by flash column chromatography (gradient elution: hexane/ethyl acetate = 100/0 to 20/80) to afford the product **21.** (0.21 g, 77%).

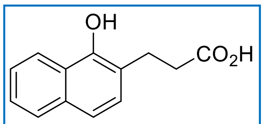

21

3-(1-Hydroxynaphthalen-2-yl)propanoic acid **21:**[17]

White solid; 210 mg; 77% yield; M.p.: 105–106 °C [Lit[S2] 104−107 ºC]; R_f = 0.51 (hexane : ethyl acetate + acetic acid = 9.6 : 0.4 + 2 drops acetic acid).

^{1}H NMR (500 MHz, $CDCl_3$): *δ* = 8.65 (brs, 1H, –COOH), 8.30–8.22 (m, 1H), 7.83–7.81 (m, 1H), 7.59–7.38 (m, 3H), 7.22–7.16 (m, 1H), 3.06–3.00 (m, 2H), 2.86–2.81 (m, 2H) ppm.

^{13}C NMR (126 MHz, $CDCl_3$): *δ* = 180.1, 149.3, 133.6, 128.2, 127.4, 125.8, 125.7, 125.3, 122.2, 120.5, 120.4, 34.8, 24.3 ppm.

The spectroscopic data are in agreement with the literature.[S2].

Procedure for dearomative spirolactonization of 21[S17]
Iodine catalyst [(+)–**5f** or (–)–**5f**; 2.2 mg, 0.005 mmol], **21** (11 mg, 0.05 mmol, 1 equiv.), *m*CPBA (13 mg, 0.075 mmol, 1.5 equiv) in $CHCl_3$ (0.5 mL) was added to a 5 mL round bottom flask at room temperature and stirred for 4 h. After completion of the reaction, chloroform was removed under vacuum. The crude mixture was washed with sat. aq. $NaHCO_3$ solution and sat. aq. $Na_2S_2O_3$ solution and extracted three times with 10 mL dichloromethane. The combined organic layers were dried over anhydrous $MgSO_4$ and was concentrated under

reduced pressure. The crude products were purified by flash chromatography on silica gel (hexane : ethyl acetate = 90/10 to 50/50) to afford the desired pure product **22**.

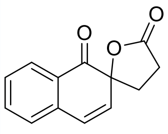

22

3,4-dihydro-1'H,5H-spiro[furan-2,2'-naphthalene]-1',5-dione **22**:

White solid; M.p.: 105–106 °C C [Lit[S2] 104−105 ºC]; R_f = 0.21 (hexane: ethyl acetate = 7 : 3).

^{1}H NMR (400 MHz, $CDCl_3$): δ = 7.96 (d, J = 7.6 Hz, 1H), 7.62–7.58 (dt, J = 7.6, 1.2 Hz, 1H), 7.39–7.35 (dt, J = 7.6, 0.8 Hz, 1H), 7.24 (d, J = 7.6 Hz, 1H), 6.63 (d, J = 10.0 Hz, 1H), 6.19 (d, J = 10.0 Hz, 1H), 2.91–2.81 (m, 1H), 2.62–2.54 (m, 1H), 2.43–2.36 (m, 1H), 2.23–2.14 (m, 1H) ppm.

^{13}C NMR (101 MHz, $CDCl_3$): δ = 196.7, 176.7, 136.8, 135.8, 132.2, 129.0, 128.0, 127.9, 127.7, 127.3, 83.6, 31.2, 26.5 ppm.

The spectroscopic data are in agreement with the literature.[S2].

HPLC analysis (Chiralcel OD-H, hexane/i-PrOH = 85/15, flow rate: 1.0 mL/min, λ = 254 nm, 25 ºC); t_{R1} = 15.654 min, t_{R2} = 20.013 min. Racemic compound of **22** was prepared using $PhI(OCOCF_3]_2$ as per the method reported in the literature[S18] and configuration of the product was determined from the reported HPLC data.[S2]

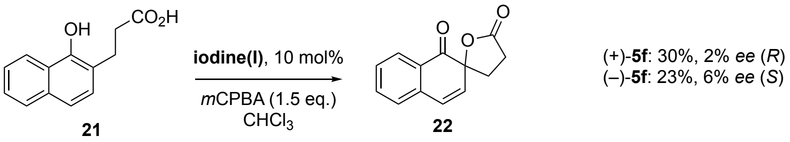

Procedures for the rearrangement of pent-1-ene-1,1-diyldibenzene 23

Procedures for the synthesis of 23[S19]

To the suspension of butyltriphenylphosphonium bromide (2.6 g, 6.6 mmol, 1.2 equiv) in THF at 0 ºC was added dropwise *n*-BuLi (2.6 mL, 6.6 mmol, 1.2 equiv) and the reaction stirred until dissolution of the salt. The benzophenone (1 g, 5.5 mmol, 1 equiv.) was added dropwise in THF (10 mL) and the reaction allowed to warm to room temperature and stirred until completion monitored by TLC. The reaction was quenched with sat. aq. NH_4Cl and extracted with diethyl ether. Combined organic extracts were washed with brine, dried with anhydrous $MgSO_4$, filtered and concentrated under reduced pressure. Column chromatography (petroleum) afforded the product alkene (1 g, 85%).

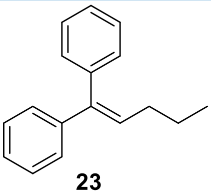

23

Pent-1-ene-1,1-diyldibenzene **23**:[S19]

Colourless oil; R_f = 0.32 (hexane: ethyl acetate = 9 : 1).

^{1}H NMR (400 MHz, $CDCl_3$): δ = 7.60–7.57 (m, 2H), 7.53–7.47 (m, 5H), 7.45–7.40 (m, 3H), 6.35 (t, J = 7.6 Hz, 1H, CH), 2.38–2.34 (q, J = 7.6 Hz, 2H, CH_2), 1.74–1.71 (m, 2H, CH_2), 1.15 (t, J = 7.6 Hz, 3H, CH_3) ppm.

^{13}C NMR (101 MHz, $CDCl_3$): δ = 143.0, 141.8, 140.5, 130.2, 130.1, 128.3, 128.2, 127.4, 127.0, 126.9, 32.0, 23.3, 14.0 ppm.

The spectroscopic data are in agreement with the literature.[S19].

Procedure for the rearrangement of 23[S20]

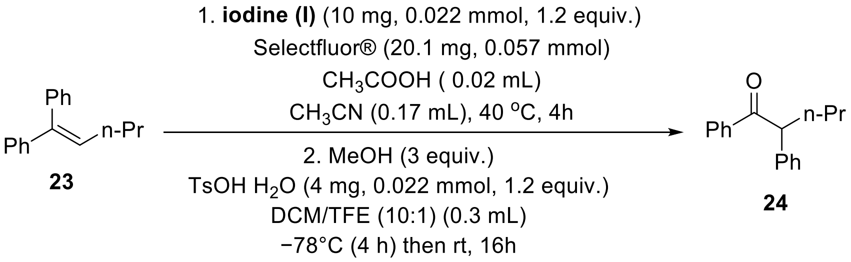

To a 5 mL round bottom flask iodine compound (+)-**5f** or (–)-**5f** (10 mg, 0.022 mmol, 1.2 equiv.), acetic acid (0.02 mL), Selectfluor® (20 mg, 0.056 mmol) and CH_3CN (0.17 mL) were added and heated at 40 °C for 4 h. After completion of the reaction solvent was removed on rotavapor. To this reaction mixture added MeOH (0.052 mL, 3 equiv.), CH_2Cl_2 : TFE (10 : 1 v/v) (0.3 mL), **23** (0.018 mmol) and cooled to −78 °C. At this temperature $TsOH{\cdot}H_2O$ (4 mg, 0.022 mmol, 1.2 equiv.) was added. The reaction was stirred for 4 h at −78°C and 16 h at room temperature and then quenched with a 1:1 mixture of aqueous sat. $NaHCO_3$ and sat. $Na_2S_2O_3$. Water (4 mL) was added, and the aqueous phase was extracted with CH_2Cl_2 (3 × 5 mL). The combined organic layers were dried with anhydrous $MgSO_4$ and concentrated under reduced pressure to give the crude product. The product **24** was isolated by preparative TLC (hexane/ethyl acetate 9:1).

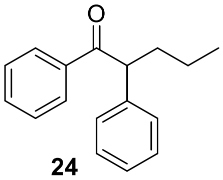

24

1,2-Diphenylpentan-1-one **24**:[S20]

Colourless oil; R_f = 0.21 (hexane: ethyl acetate = 9 : 1).

^{1}H NMR (400 MHz, $CDCl_3$): δ = 8.00–7.98 (m, 2H), 7.50–7.48 (m, 1H), 7.43–7.39 (m, 2H), 7.35–7.29 (m, 4H), 7.24–7.22 (m, 1H), 4.59 (t, J = 7.6 Hz, 1H), 2.20–2.15 (m, 1H), 1.88–1.83 (m, 1H), 1.36–1.27 (m, 2H), 0.94 (t, J = 7.2 Hz, 3H) ppm.

^{13}C NMR (101 MHz, $CDCl_3$): δ = 200.3, 140.0, 137.2, 132.9, 129.0, 128.8, 128.7, 128.4, 127.1, 53.6, 36.3, 21.0, 14.2 ppm.

The spectroscopic data are in agreement with the literature.[S20].

HPLC analysis (YMC Chiral Amylose-C, hexane/i-PrOH = 99.5/0.5, flow rate 1.0 mL/min, λ = 254 nm, 25 °C); t_{R1} = 6.898 min, t_{R2} = 9.106 min. Racemic ketone was prepared using $PhI(OAc)_2$ in place of chiral catalyst as per the method reported in the literature[S20] and configuration of the product was determined from the reported HPLC data[S20]

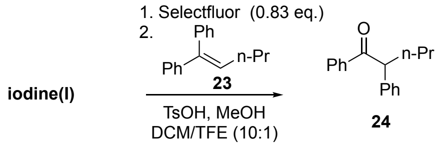

(+)-**5f**: 70%, 1% *ee* (*R*)
(–)-**5f**: 63%, 1% *ee* (*S*)

3. References

S1 a) J. Chmiel, I. Heesemann, A. Mix, B. Neumann, H.-G. Stammler, N. W. Mitzel, *Eur. J. Org. Chem*. **2010**, 3897–3907; b) M. E. Rogers, B. A. Averill, *J. Org. Chem*. **1986**, *51*, 3308–3314.

S2 T. Dohi, A. Maruyama, N. Takenaga, K. Senami, Y. Minamitsuji, H. Fujioka, S. B. Caemmerer, Y. Kita, *Angew. Chem. Int. Ed*. **2008**, *47*, 3787–3790.

S3 F.-L. Zhang, K. Hong, T.-J. Li, H. Park, J. -Q. Yu, *Science* **2016**, *351*, 252–256.

S4 S. M. Rafiq, R. Sivasakthikumaran, J. Karunakaran, A. K. Mohanakrishnan, *Eur. J. Org. Chem*. **2015**, 5099–5114.

S5 L. Lei, P. Wu, Z. Liu, J. Lou, *Tetrahedron Lett*. **2021**, *67*, 152865.

S6 D. J. Paghdar, M. R. Kolekar, T. N. Deshpandey, S. P. Patil, Y. A. Chavan, P. C. Ray, G. P. Singh. An improved process for preparation of amisulpride. WO 2011/158084 A1.

S7 K. B. S. Magar, L. Xia, Y. R. Lee, *Chem. Commun*. **2015**, *51*, 8592–8595.

S8 B. Shi, Z. Li, Y. Liu, L. Shangguan, H. Zhu, H. Ju, F. Huang, *Tetrahedron Lett*. **2018**, *59*, 3477–3480.

S9 L. Lu, Q. Chen, X. Zhu, C. Chen, *Synthesis* **2003**, 2464–2466.

S10 H. L. Newson, D. A. Wild, S. Y. Yeung, B. W. Skelton, G. R. Flematti, J. E. Allan, M. J. Piggott, *J. Org. Chem*. **2016**, *81*, 3127−3135.

S11 J. F. W. McOmie, M. L. Watts, D. E. West, *Tetrahedron* **1968**, *24*, 2289-2292.

S12 Y. Shimizu, T. Naito, F. Ogura, M. Nakagawa, *Bull. Chem. Soc. Jpn*. **1973**, *46*, 1520–1525.

S13 a) T. Hokamp, T. Wirth, *J. Org. Chem*. **2019**, *84*, 8674–8682; b) H. Alharbi, M. Elsherbini, J. Qurban, T. Wirth, *Chem. Eur. J*. **2021**, *27*, 4317–4321; c) S. M. Altermann, R. D. Richardson, T. K. Page, R. K. Schmidt, E. Holland, U Mohammed, S. M. Paradine, A. N. French, C. Richter, A. M. Bahar, B. Witulski, T. Wirth, *Eur. J. Org. Chem*. **2008**, 5315–5328.

S14 M. U. Tariq, W. J. Moran, *Eur. J. Org. Chem*. **2020**, 5153–5160.

S15 A.H. Abazida, B. J. Nachtsheim, *Chem. Commun*. **2021**, *57*, 8822–8825.

S16 M. Uyanik, S. Ishizaki, K. Ishihara, *Org. Synth*. **2021**, *98*, 28–50.

S17 M. Bekkaye, G. Masson, *Synthesis*, **2016**, *48*, 302–312.

S18 H. Zhang, R. A. Cormanich, T. Wirth, *Chem. Eur. J*. **2022**, *28*, e202103623.

S19 M. Brown, R. Kumar, J. Rehbein, T. Wirth, *Chem. Eur. J*. **2016**, *22*, 4030–4035.

S20 J. Qurban, M. Elsherbini, T. Wirth, *J. Org. Chem*. **2017**, *82*, 11872–11876.

4. X-Ray analysis

**2-Iodotriptycene (5a)**		**Crystal structure**
Molecular Formula	$C_{20}H_{13}I$	
Space group	Cc	
Cell lengths	**a** 17.9826(10) **b** 10.5276(8) **c** 16.4325(12)	
Cell angles	**α** 90 **β** 101.519(6) **γ** 90	
Cell volume	3048.24	
Z, Z"	Z: 8 Z': 0	
R-Factor (%)	2.53	CCDC: 2132845

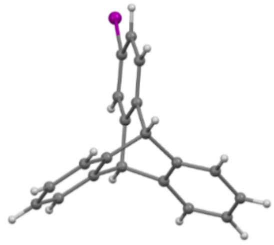

Solvent used for crystallization: Ethanol at room temperature slow evaporation.

**1-Iodotriptycene (5b)**		**Crystal structure**
Molecular Formula	$C_{20}H_{13}I$	
Space group	P 2_1/n	
Cell lengths	**a** 14.0012(10) **b** 8.1387(4) **c** 14.2709(10)	
Cell angles	**α** 90 **β** 112.248(8) **γ** 90	
Cell volume	1505.13	
Z, Z"	Z: 4 Z': 0	
R-Factor (%)	5.08	CCDC: 2132846

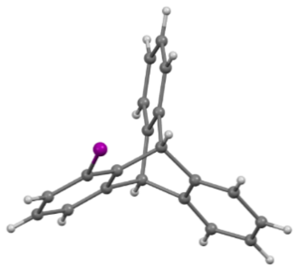

Solvent used for crystallization: Ethanol at room temperature slow evaporation.

**1-chloro-6-iodo-9,10-dihydro-9,10-[1,2]benzenoanthracene (*anti*-5c)**		**Crystal structure**
Molecular Formula	C_{20} H_{12} Cl I	
Space group	P -1	
Cell lengths	**a** 8.1237(3) **b** 8.9905(3) **c** 12.2913(4)	
Cell angles	a 93.515(3) b 105.142(3) g 111.364(3)	
Cell volume	794.679	
Z, Z"	Z: 2 Z': 0	
R-Factor (%)	3.52	CCDC: 2132847

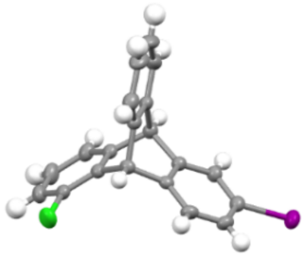

Solvent used for crystallization: Acetonitrile at room temperature slow evaporation. Crystal observed from racemic mixture.

**5-iodo-1,4-dimethoxy-9,10-dihydro-9,10-[1,2]benzenoanthracene (5f)**		**Crystal structure**
Molecular Formula	C_{22} H_{17} I O_2	
Space group	P 21/c	
Cell lengths	**a** 8.1985(3) **b** 20.0397(7) **c** 11.2762(5)	
Cell angles	α 90 β 106.854(4) γ 90	
Cell volume	1773.05	
Z, Z"	Z: 4 Z': 0	
R-Factor (%)	5.13	CCDC: 2132844

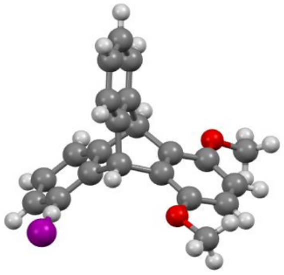

*Solvent used for crystallization***:** Ethyl acetate at room temperature slow evaporation. Crystal observed from racemic mixture.

**(9*S*, 10*S*)-(-)-5f**		**Crystal structure**
Molecular Formula	$C_{22}H_{17}IO_2$	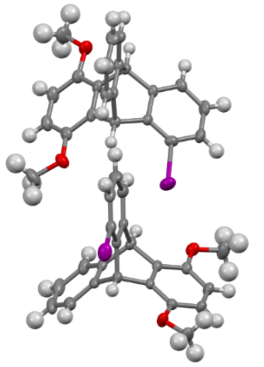
Space group	$P\,2_1$	
Cell lengths	**a** 13.9586(3) **b** 8.1746(2) **c** 15.7126(3)	
Cell angles	α 90 β 92.091(2) γ 90	
Cell volume	1791.71	
Z, Z”	Z: 4 Z': 0	
R-Factor (%)	5.08	CCDC: 2132843

Solvent used for crystallization: Ethyl acetate at room temperature slow evaporation.

**(9*R*, 10R)-(+)-5f**		**Crystal structure**
Molecular Formula	$C_{22}H_{17}IO_2$	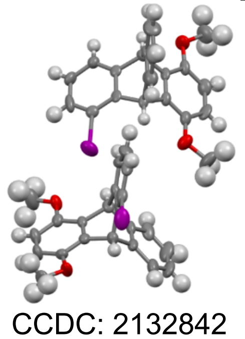
Space group	$P\,2_1$	
Cell lengths	**a** 14.0009(10) **b** 8.1691(5) **c** 15.8376(11)	
Cell angles	α 90 β 92.037(6) γ 90	
Cell volume	1810.28	
Z, Z”	Z: 4 Z': 0	
R-Factor (%)	6.02	CCDC: 2132842

Solvent used for crystallization: Ethyl acetate at room temperature slow evaporation.

5. NMR and Mass Spectra

^{1}H and ^{13}C-NMR spectra of 2-iodo-9,10-dihydro-9,10-[1,2]benzenoanthracene (5a)

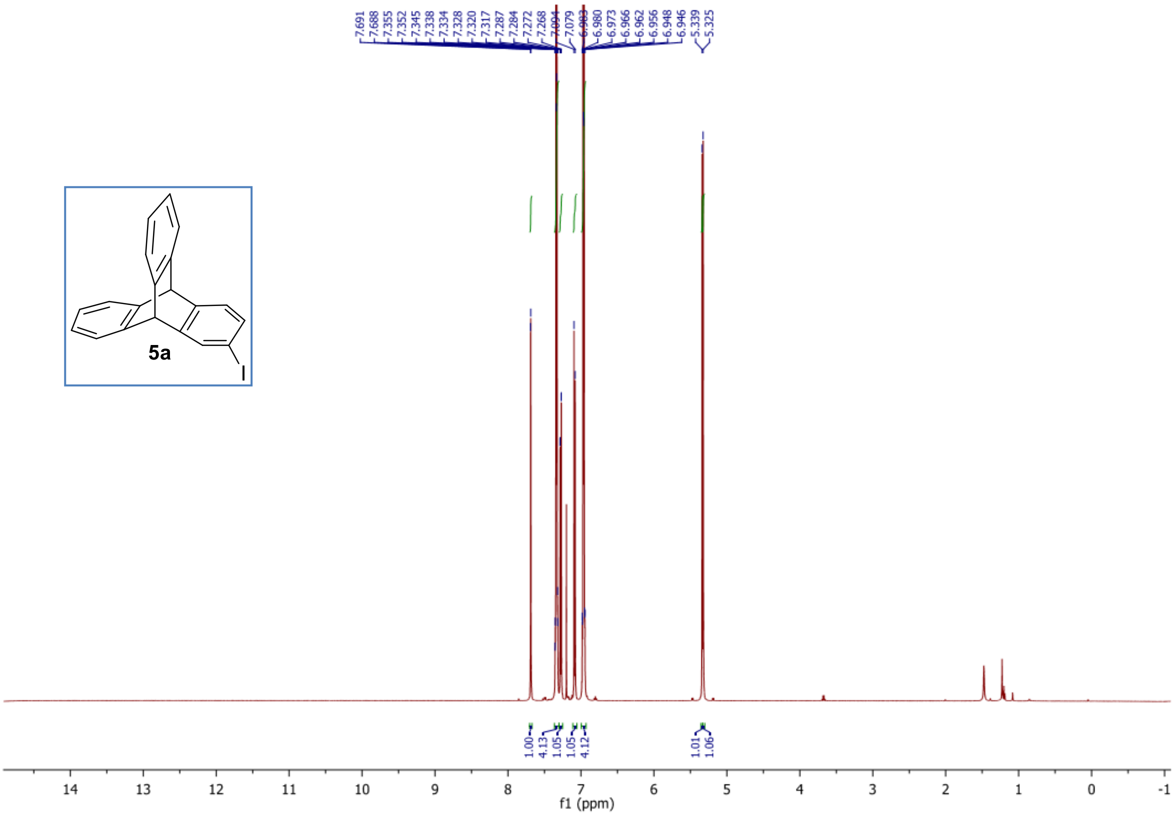

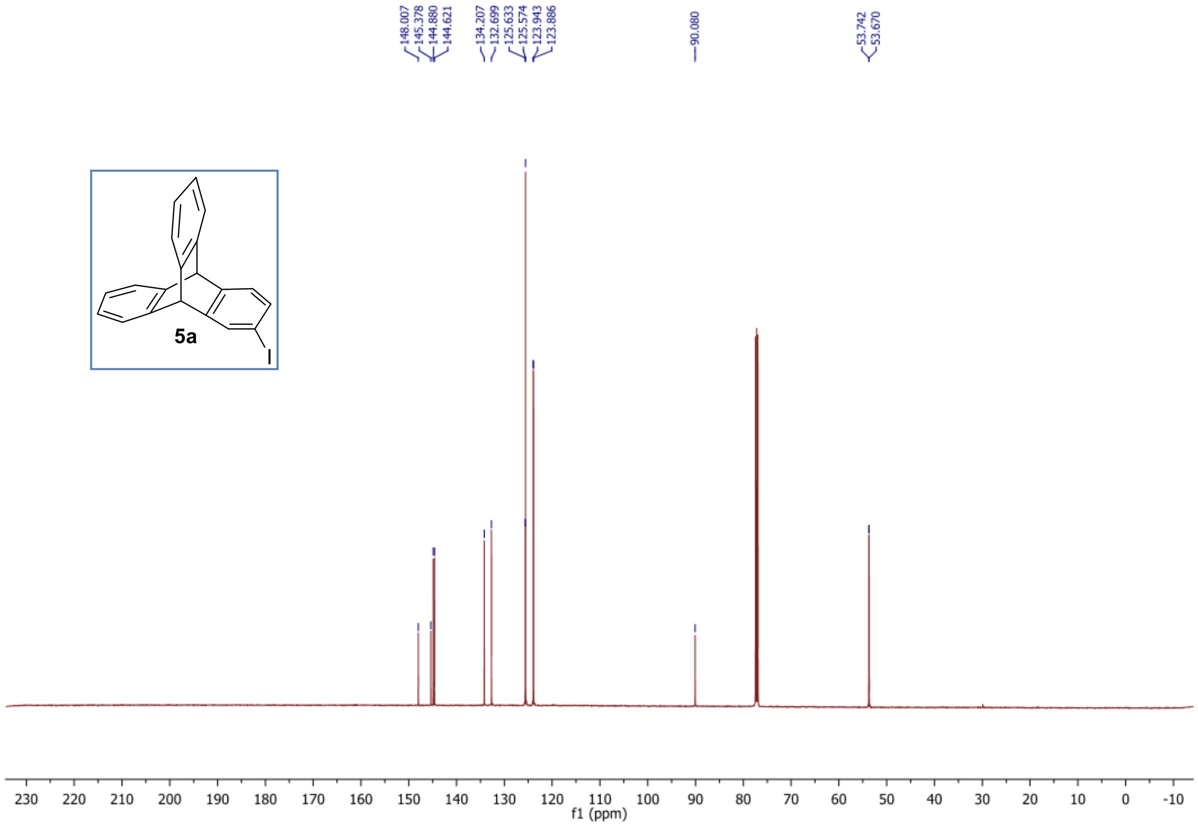

Mass spectra of 2-iodo-9,10-dihydro-9,10-[1,2]benzenoanthracene (5a)

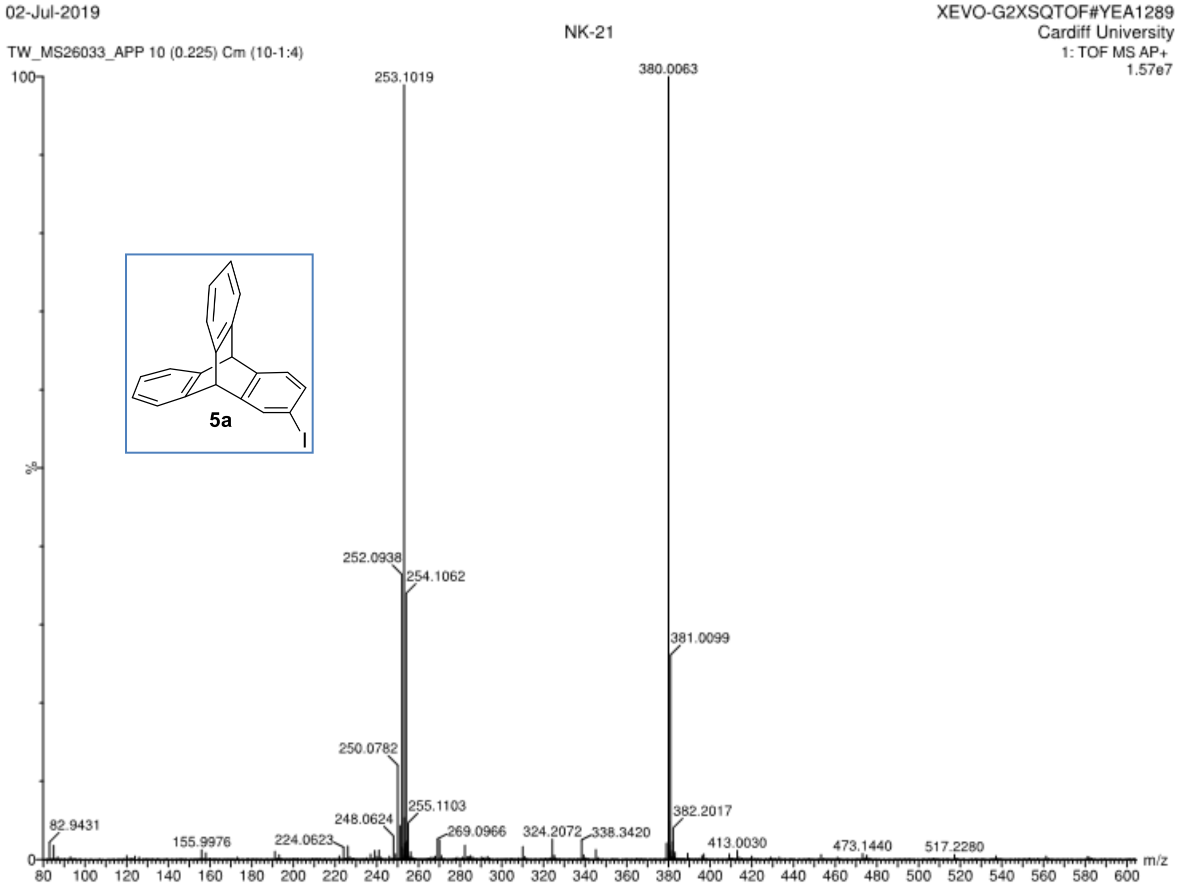

Elemental Composition Report **Page 1**

Single Mass Analysis
Tolerance = 5.0 PPM / DBE: min = -1.5, max = 200.0
Element prediction: Off
Number of isotope peaks used for i-FIT = 3

Monoisotopic Mass, Odd and Even Electron Ions
3 formula(e) evaluated with 1 results within limits (up to 50 closest results for each mass)
Elements Used:
C: 0-20 H: 0-13 127I: 0-2

Minimum:				-1.5				
Maximum:		5.0	5.0	200.0				
Mass	Calc. Mass	mDa	PPM	DBE	i-FIT	Norm	Conf(%)	Formula
380.0063	380.0062	0.1	0.3	14.0	753.8	n/a	n/a	C20 H13 127I

^{1}H and ^{13}C-NMR spectra of 1-iodo-9,10-dihydro-9,10-[1,2]benzenoanthracene (5b)

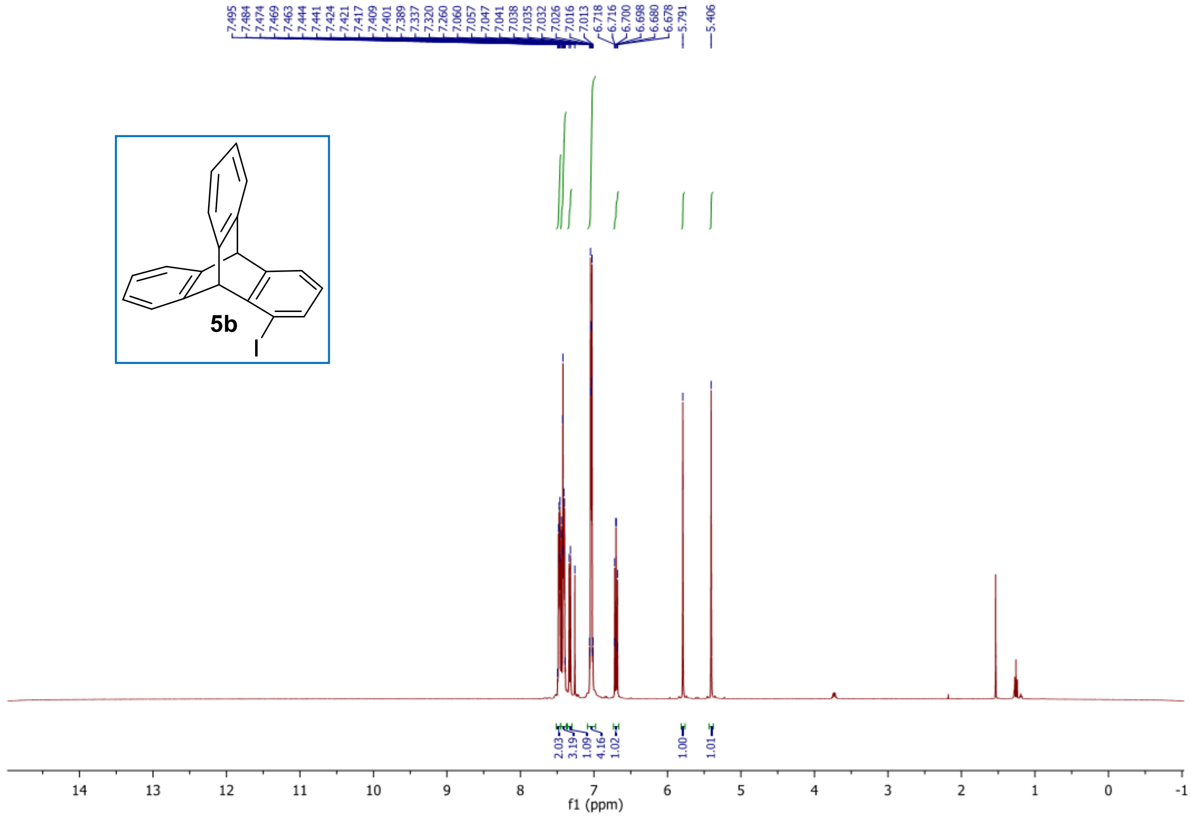

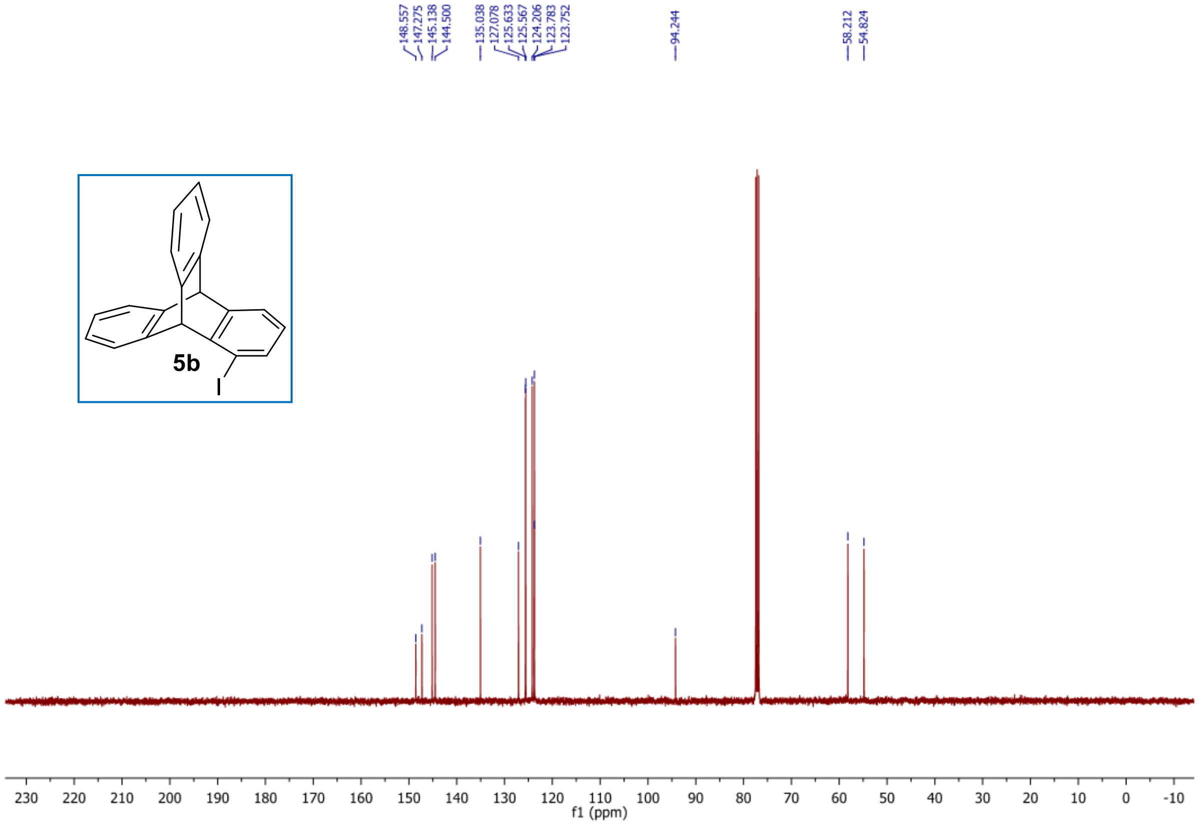

Mass spectra of 1-iodo-9,10-dihydro-9,10-[1,2]benzenoanthracene (5b)

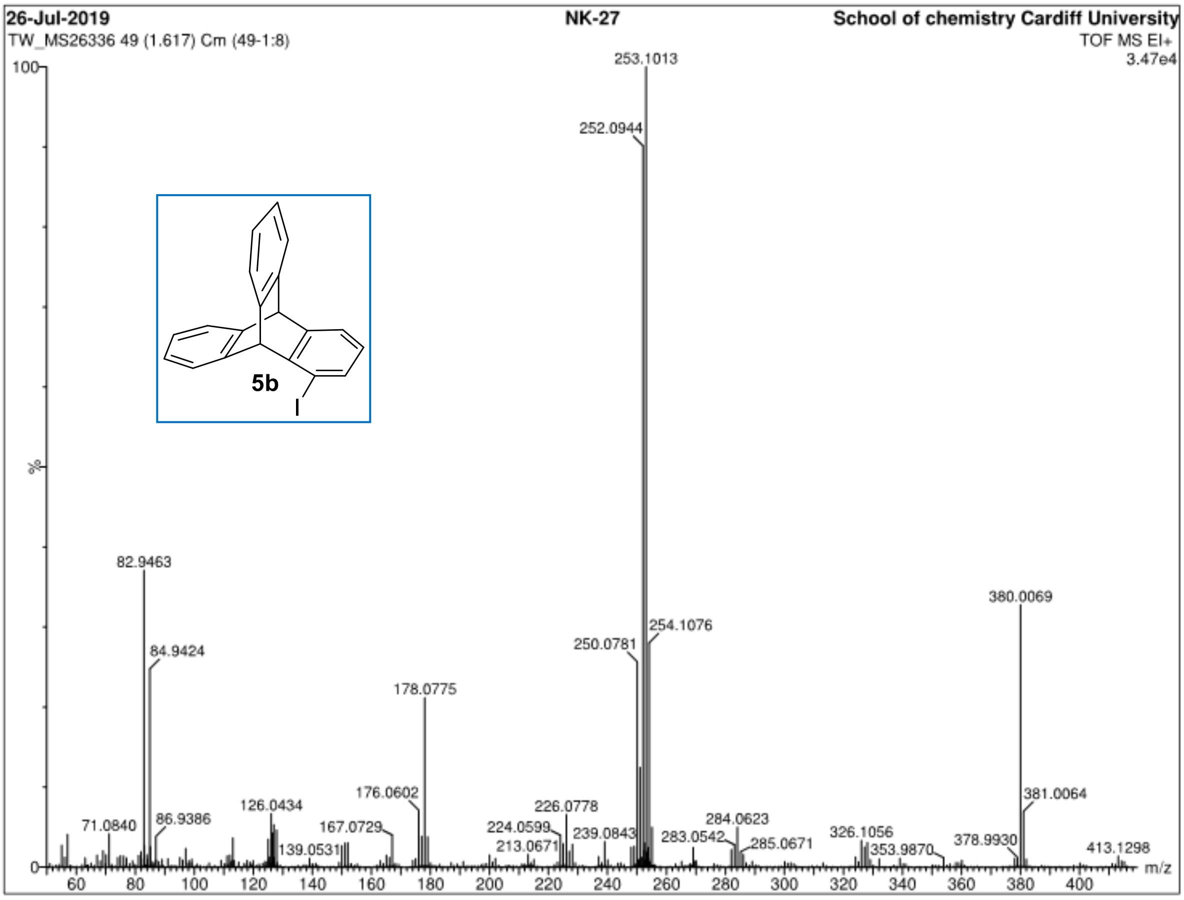

Elemental Composition Report

Page 1

Single Mass Analysis

Tolerance = 10.0 PPM / DBE: min = -1.5, max = 50.0
Element prediction: Off

Monoisotopic Mass, Odd and Even Electron Ions
2 formula(e) evaluated with 1 results within limits (up to 50 best isotopic matches for each mass)
Elements Used:
C: 0-20 H: 0-13 127I: 0-1

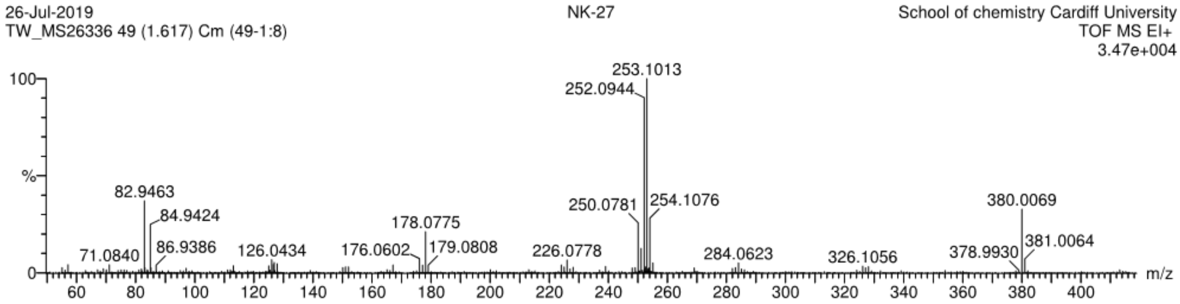

Minimum:				-1.5		
Maximum:		5.0	10.0	50.0		
Mass	Calc. Mass	mDa	PPM	DBE	i-FIT	Formula
380.0069	380.0062	0.7	1.8	14.0	12.3	C20 H13 127I

^{1}H and ^{13}C-NMR spectra of 9,10-dihydro-9,10-[1,2]benzenoanthracen-2-yl)-λ[3]-iodanediyl diacetate (6a)

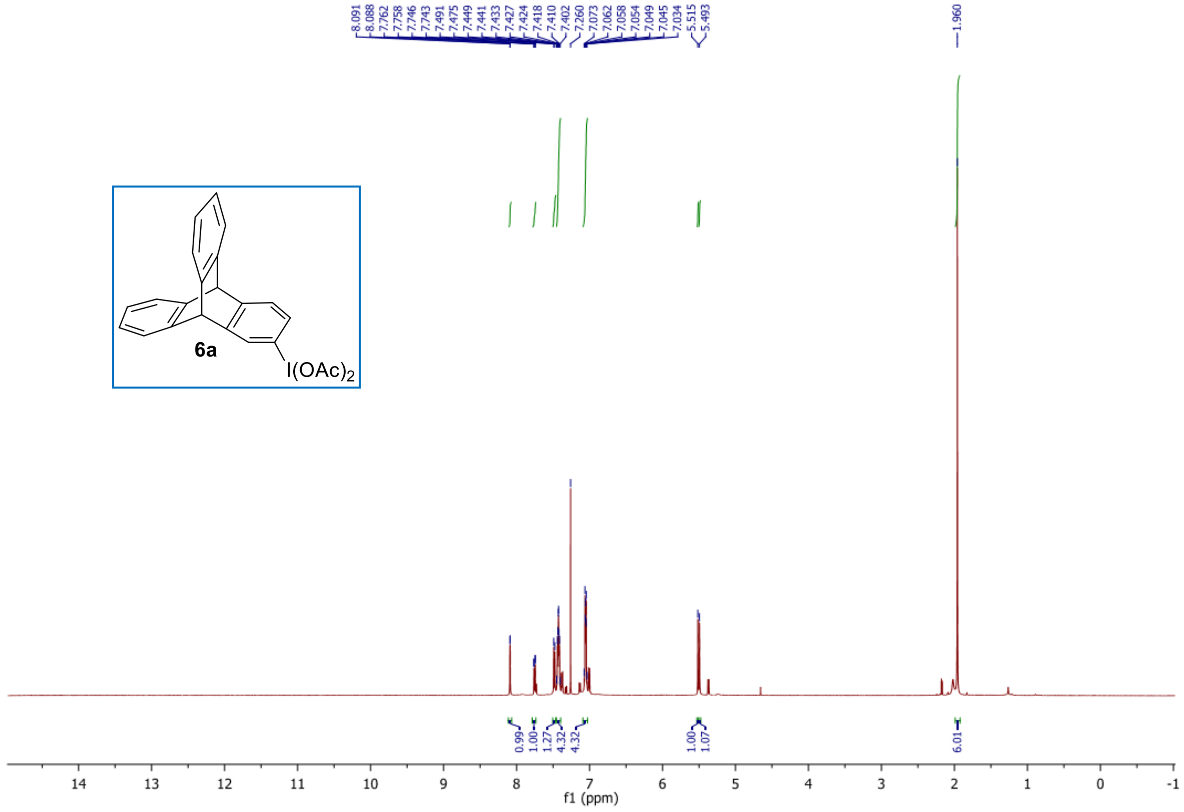

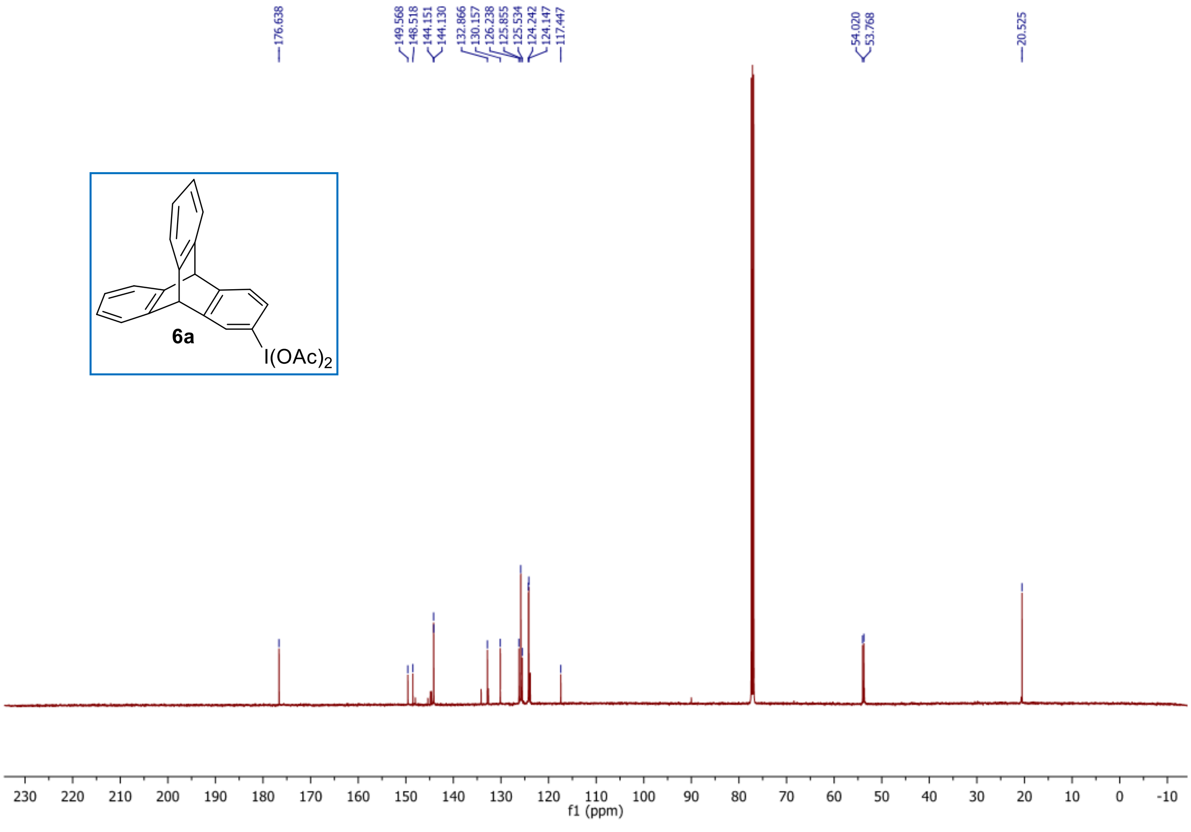

^{1}H and ^{13}C-NMR spectra of 9,10-dihydro-9,10-[1,2]benzenoanthracen-1-yl)-λ^{3}-iodanediyl diacetate (6b)

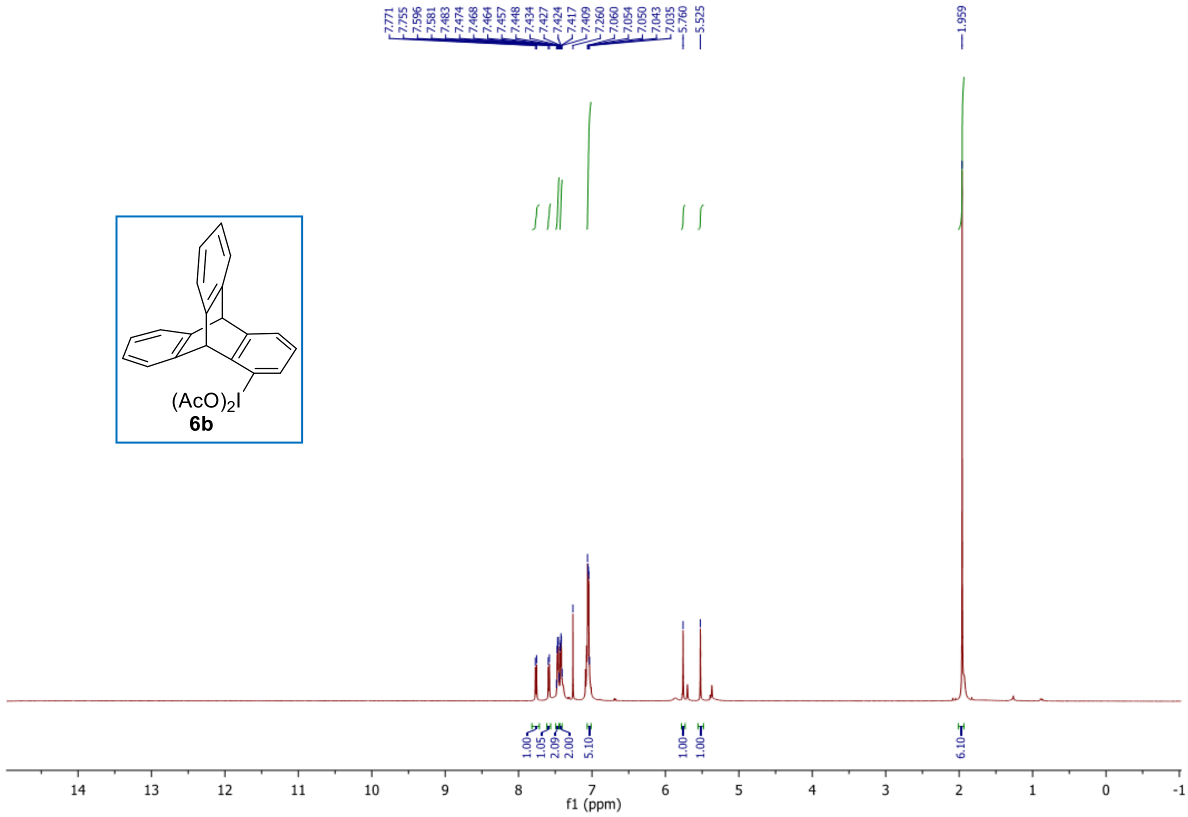

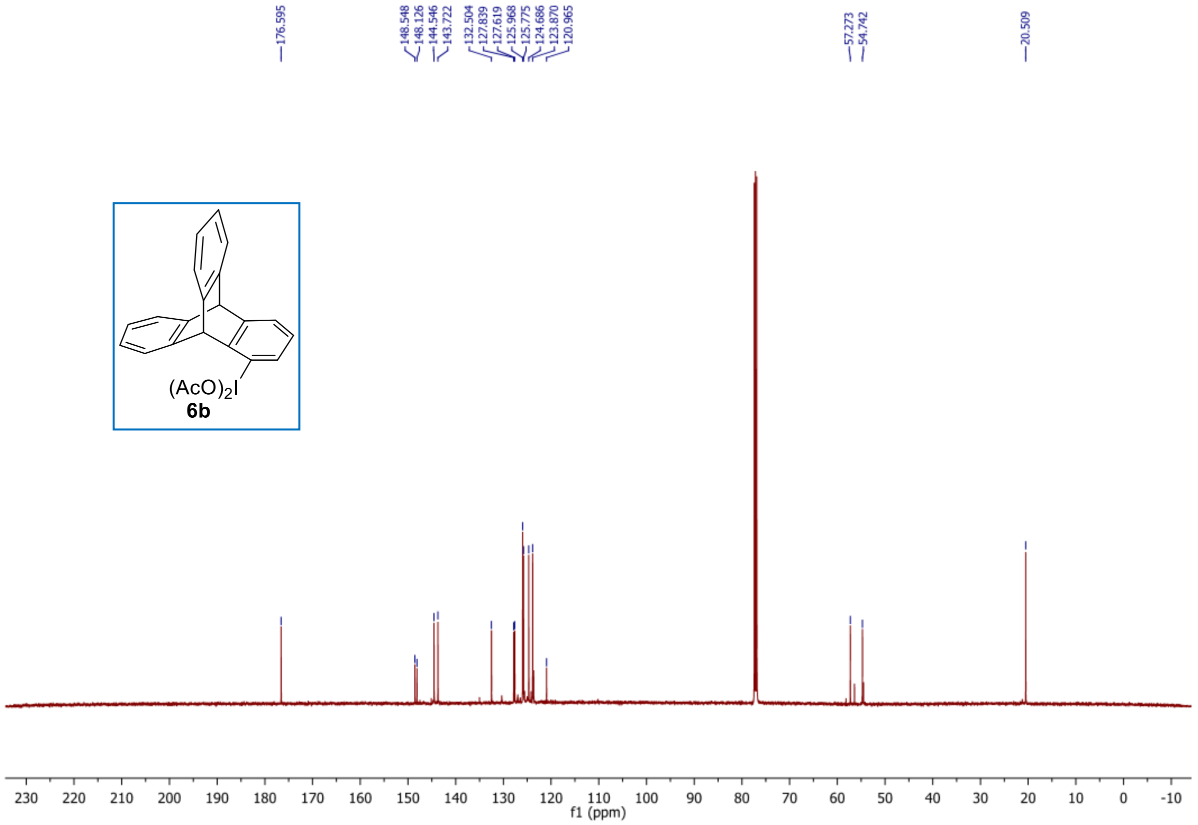

^{1}H and ^{13}C-NMR spectra of 1-chloro-6-iodo-9,10-dihydro-9,10-[1,2]benzenoanthracene (*anti*-**5c**)

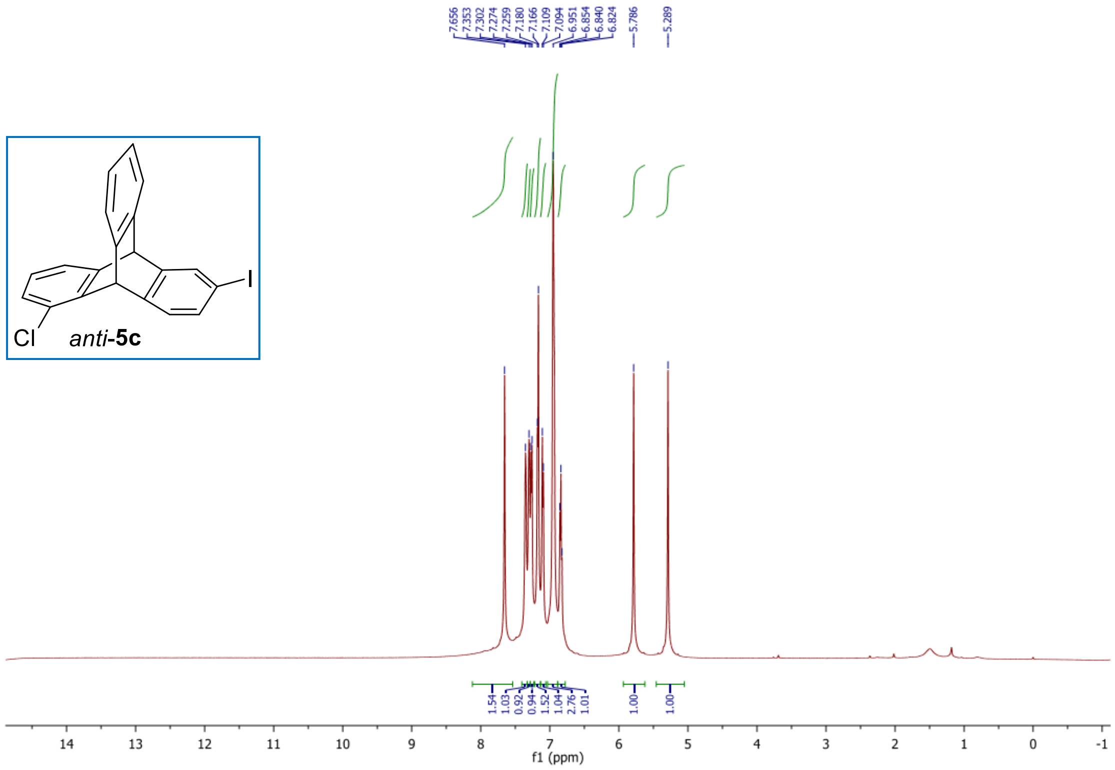

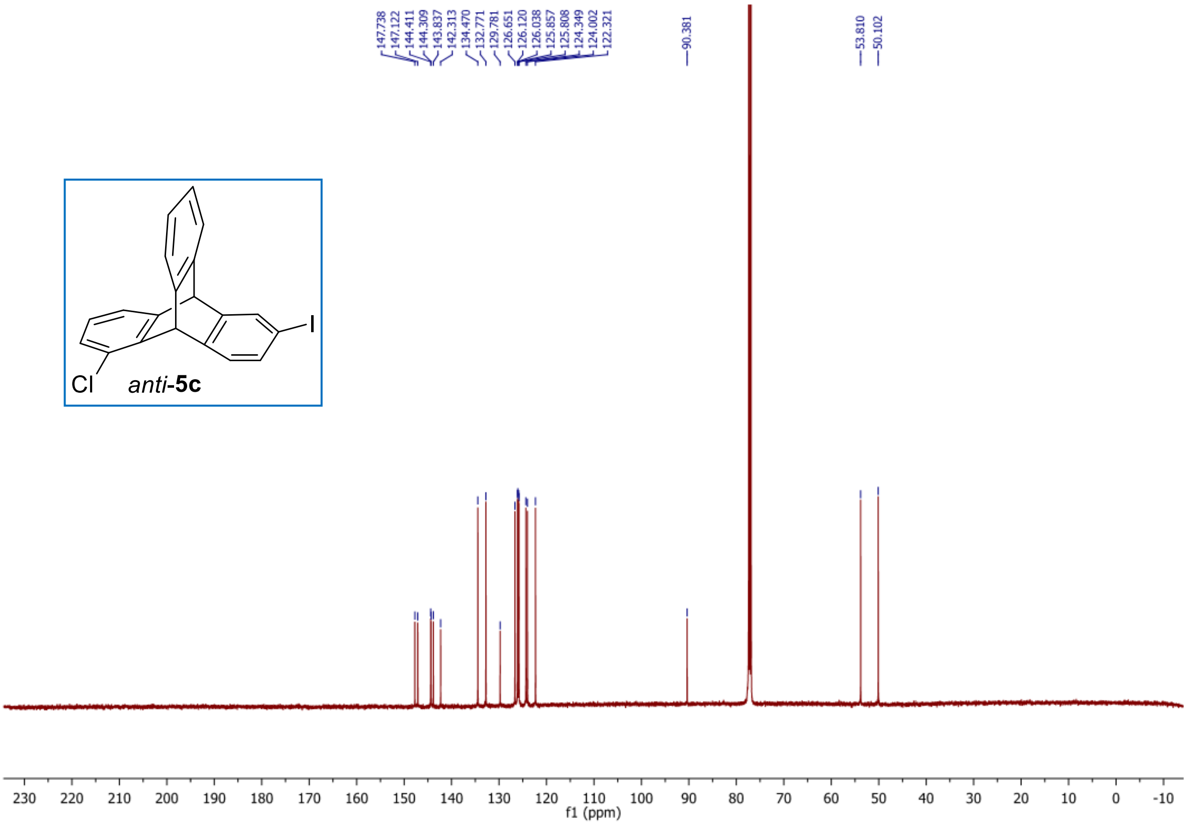

Mass spectra of 1-chloro-6-iodo-9,10-dihydro-9,10-[1,2]benzenoanthracene (*anti*-5c)

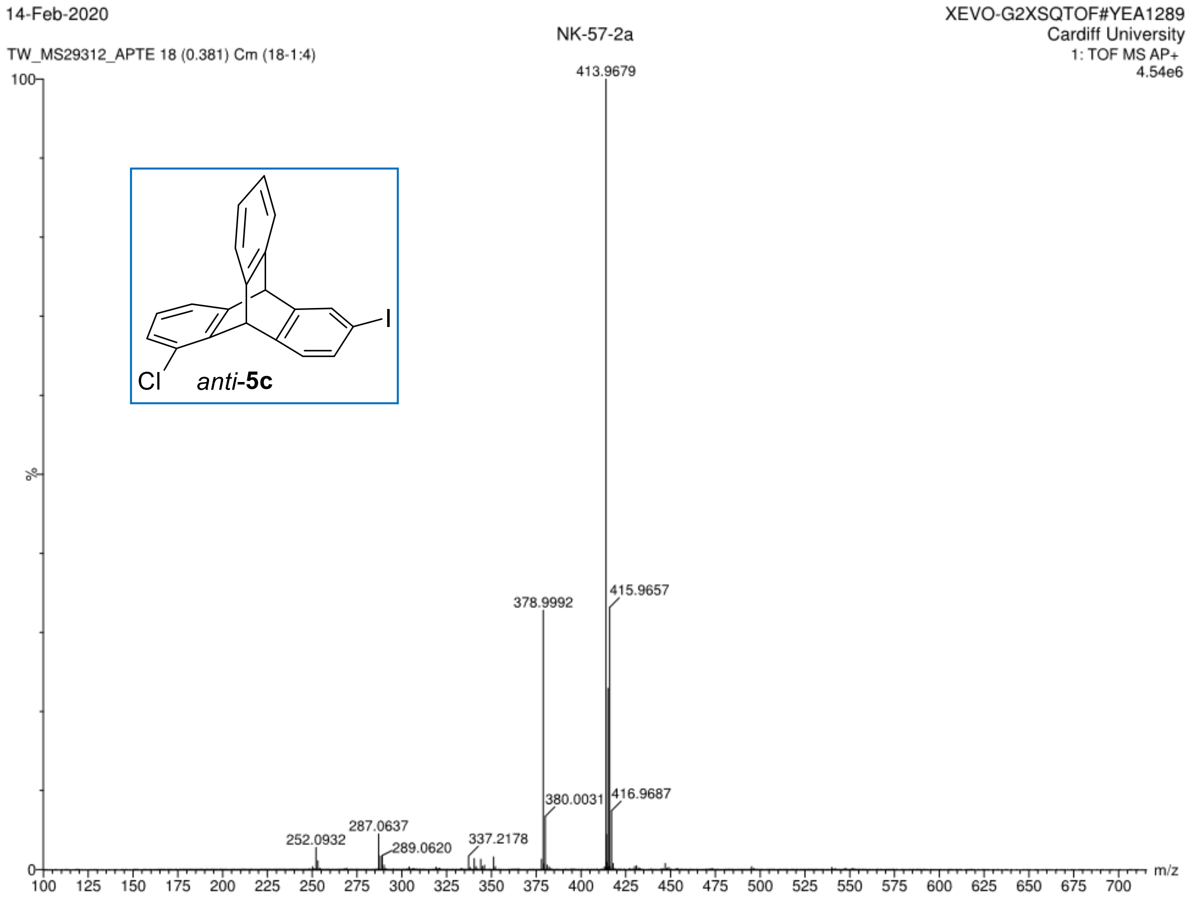

Elemental Composition Report **Page 1**

Single Mass Analysis
Tolerance = 5.0 PPM / DBE: min = -1.5, max = 200.0
Element prediction: Off
Number of isotope peaks used for i-FIT = 3

Monoisotopic Mass, Odd and Even Electron Ions
3 formula(e) evaluated with 1 results within limits (up to 50 closest results for each mass)
Elements Used:
C: 0-20 H: 0-12 Cl: 0-1 127I: 0-1

Minimum:				-1.5				
Maximum:		5.0	5.0	200.0				
Mass	Calc. Mass	mDa	PPM	DBE	i-FIT	Norm	Conf(%)	Formula
413.9679	413.9672	0.7	1.7	14.0	521.5	n/a	n/a	C20 H12 Cl 127I

^{1}H and ^{13}C-NMR spectra of 1-chloro-7-iodo-9,10-dihydro-9,10-[1,2]benzenoanthracene (*syn*-5c)

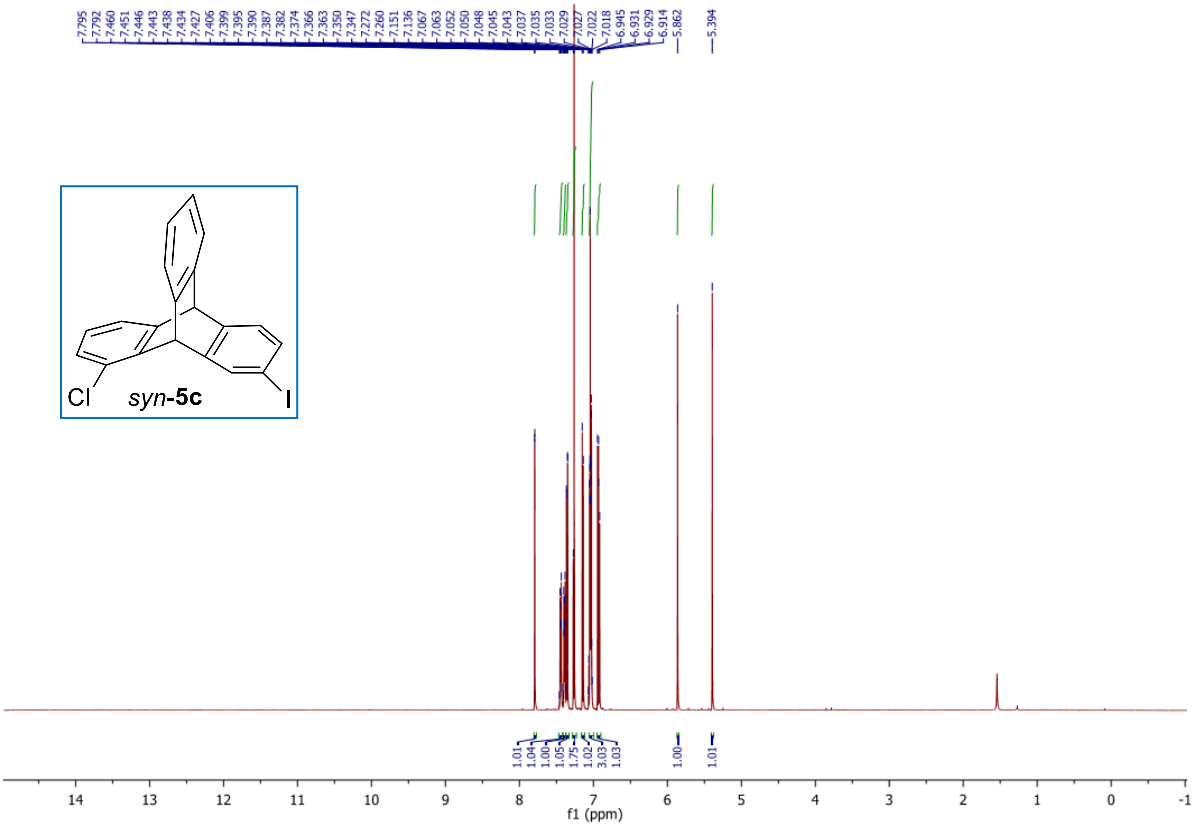

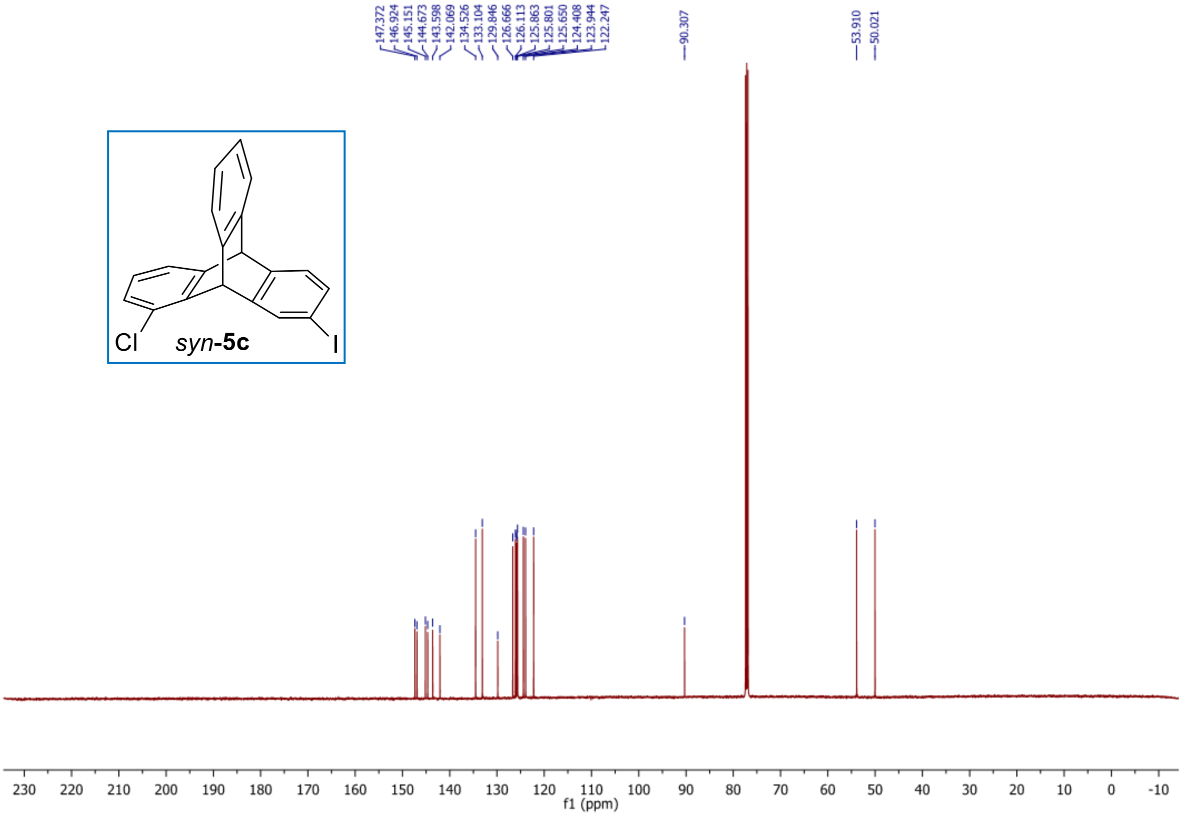

Mass spectra of 1-chloro-7-iodo-9,10-dihydro-9,10-[1,2]benzenoanthracene (*syn*-5c)

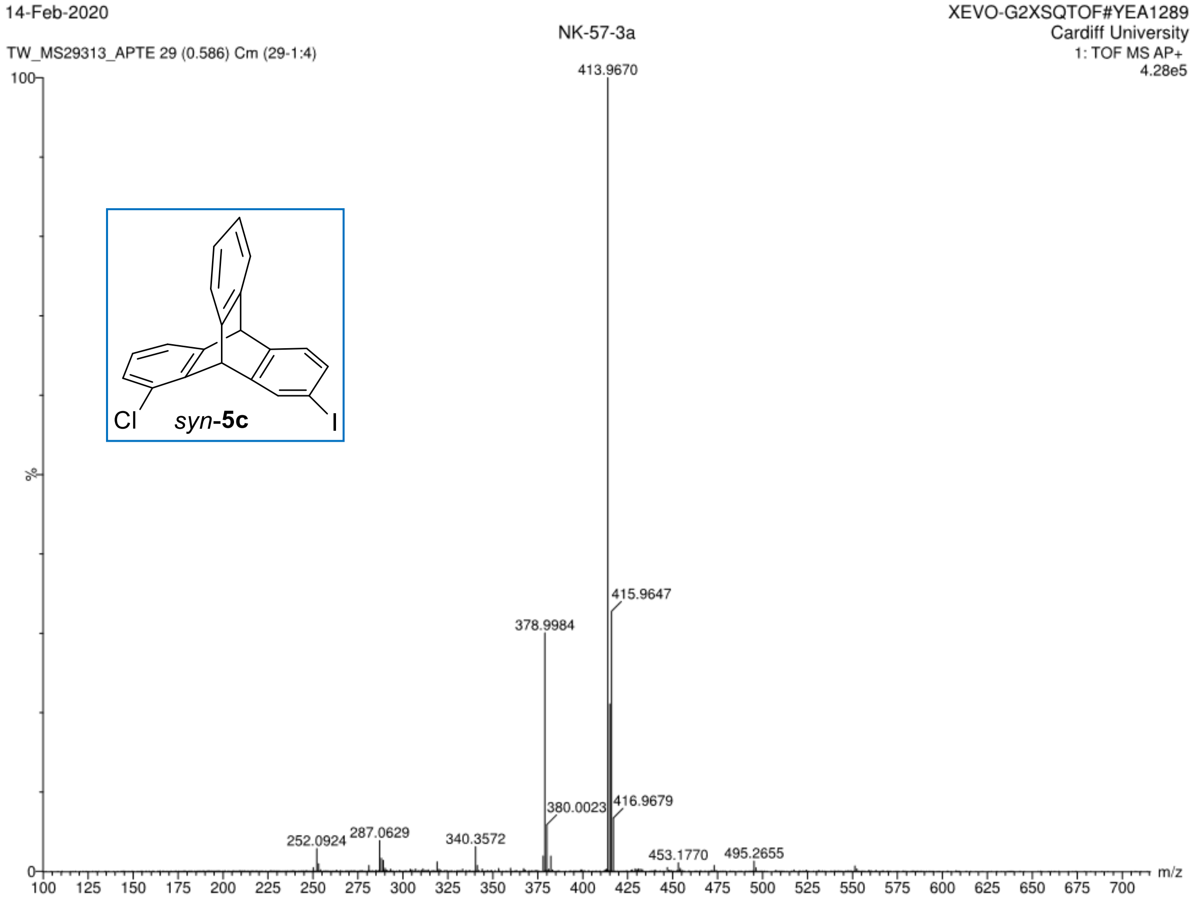

Elemental Composition Report

Page 1

Single Mass Analysis

Tolerance = 5.0 PPM / DBE: min = -1.5, max = 200.0
Element prediction: Off
Number of isotope peaks used for i-FIT = 3

Monoisotopic Mass, Odd and Even Electron Ions
3 formula(e) evaluated with 1 results within limits (up to 50 closest results for each mass)
Elements Used:
C: 0-20 H: 0-12 Cl: 0-1 127I: 0-1

Minimum:				-1.5				
Maximum:		5.0	5.0	200.0				
Mass	Calc. Mass	mDa	PPM	DBE	i-FIT	Norm	Conf(%)	Formula
413.9670	413.9672	-0.2	-0.5	14.0	168.8	n/a	n/a	C20 H12 Cl 127I

^{1}H and ^{13}C-NMR spectra of 2-iodo-7-methoxy-9,10-dihydro-9,10-[1,2]benzenoanthracene (*syn*-5d) and 2-iodo-6-methoxy-9,10-dihydro-9,10-[1,2]benzenoanthracene (*anti*-5d)

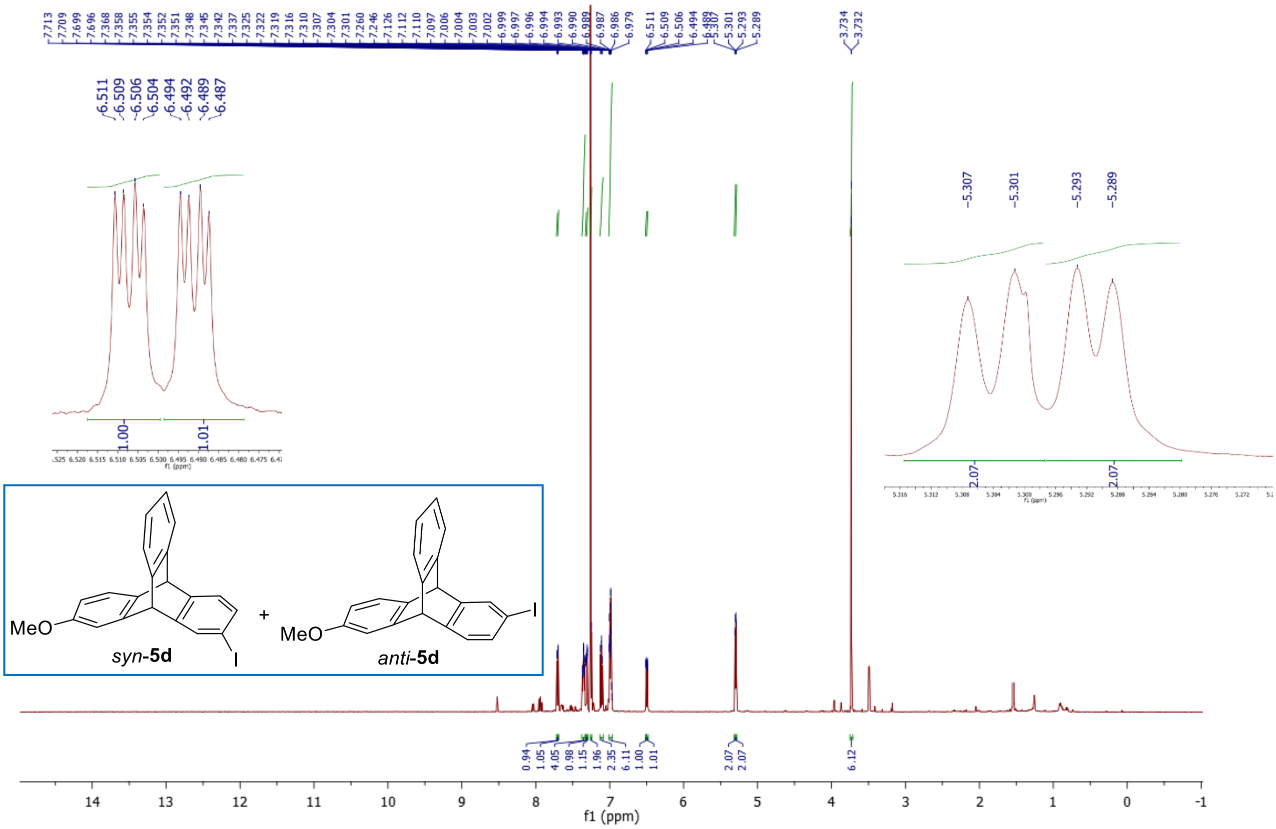

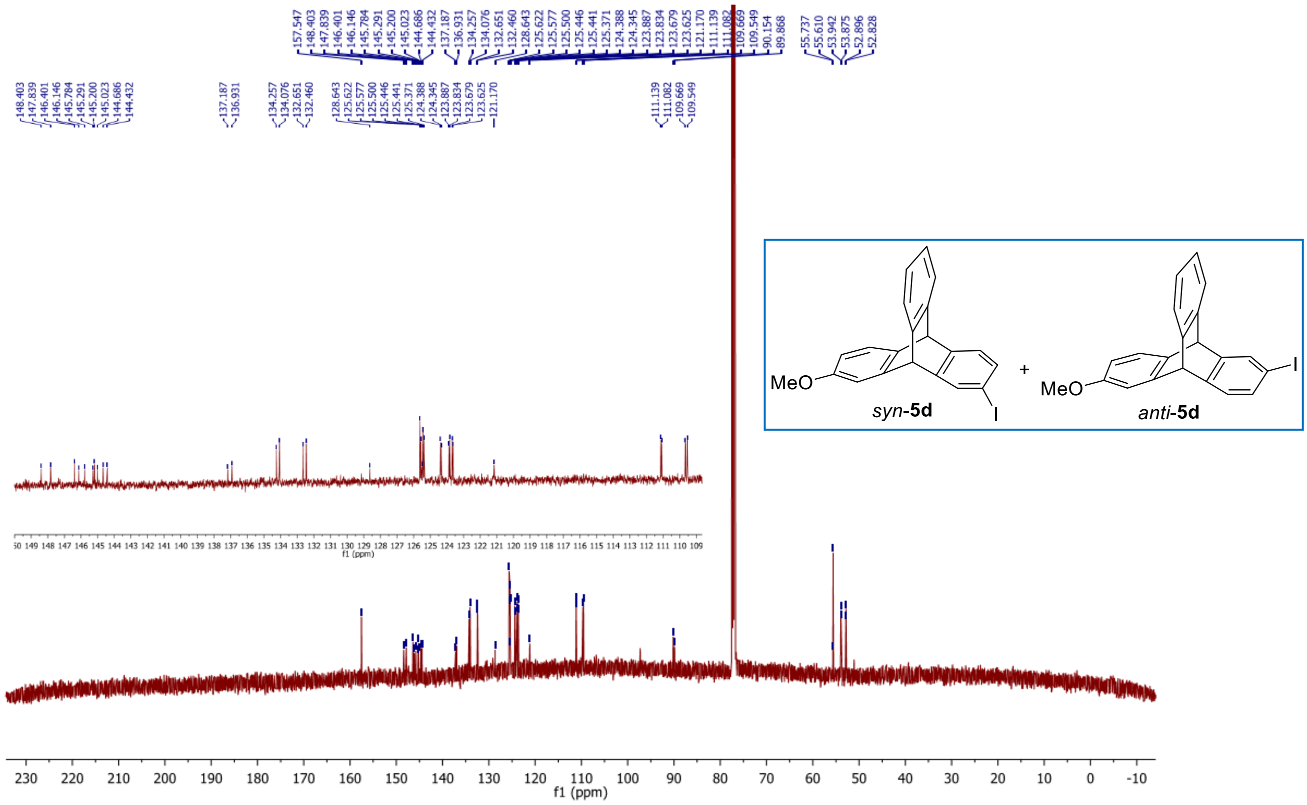

^{1}H and ^{13}C-NMR spectra of 2-(4-methoxybenzyl)benzaldehyde (9a)

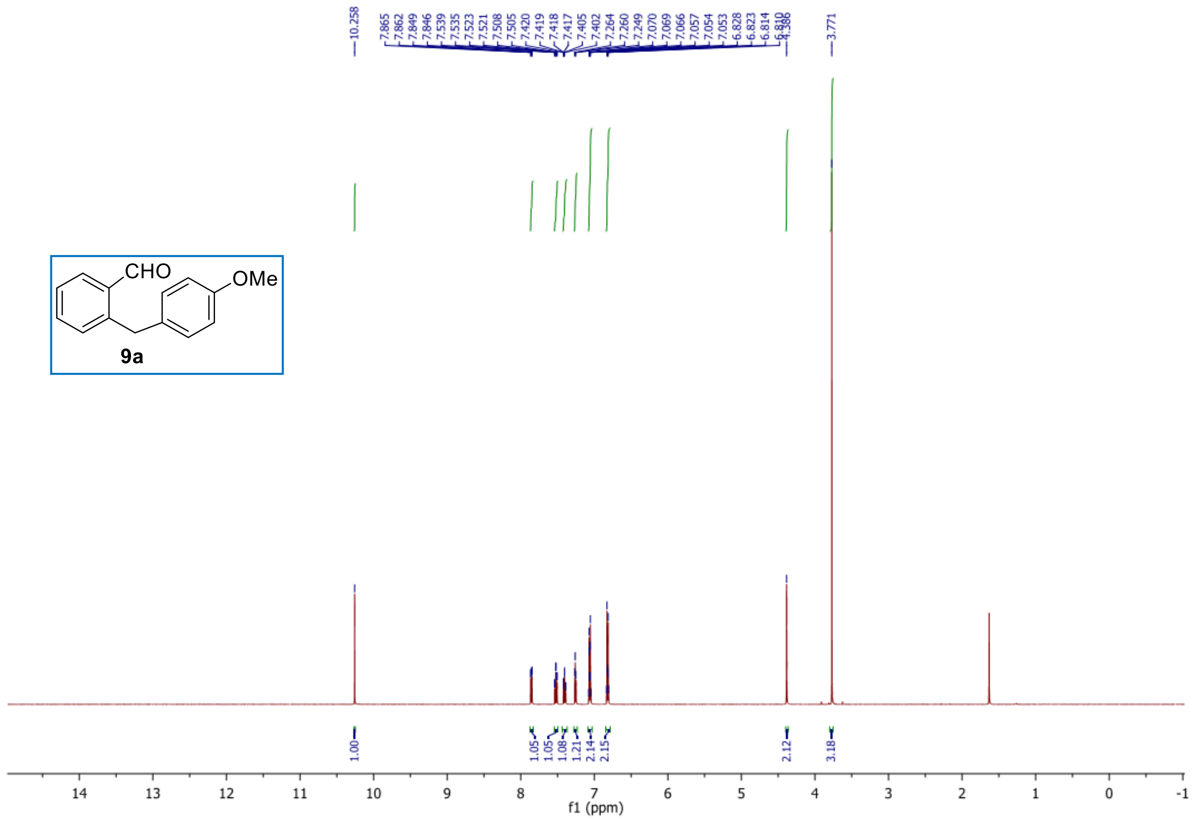

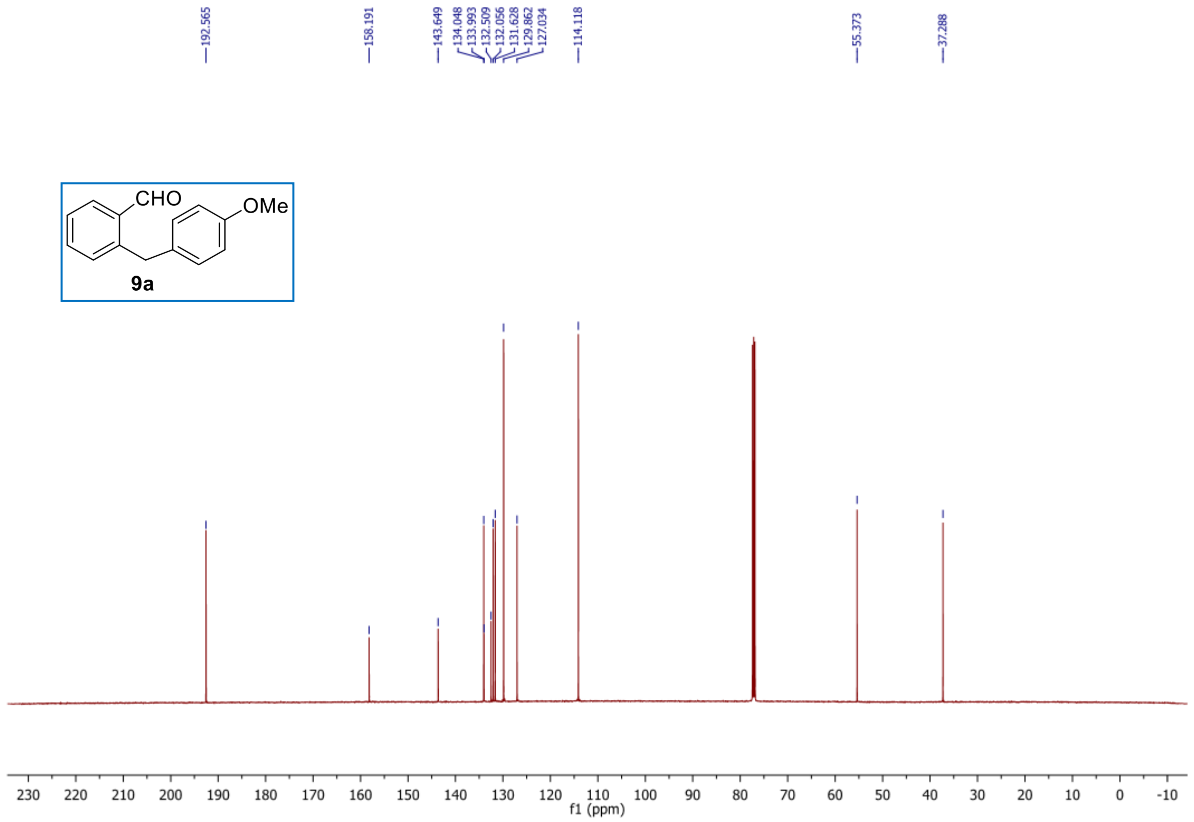

^{1}H and ^{13}C-NMR spectra of 2-(2-methoxybenzyl)benzaldehyde (9b)

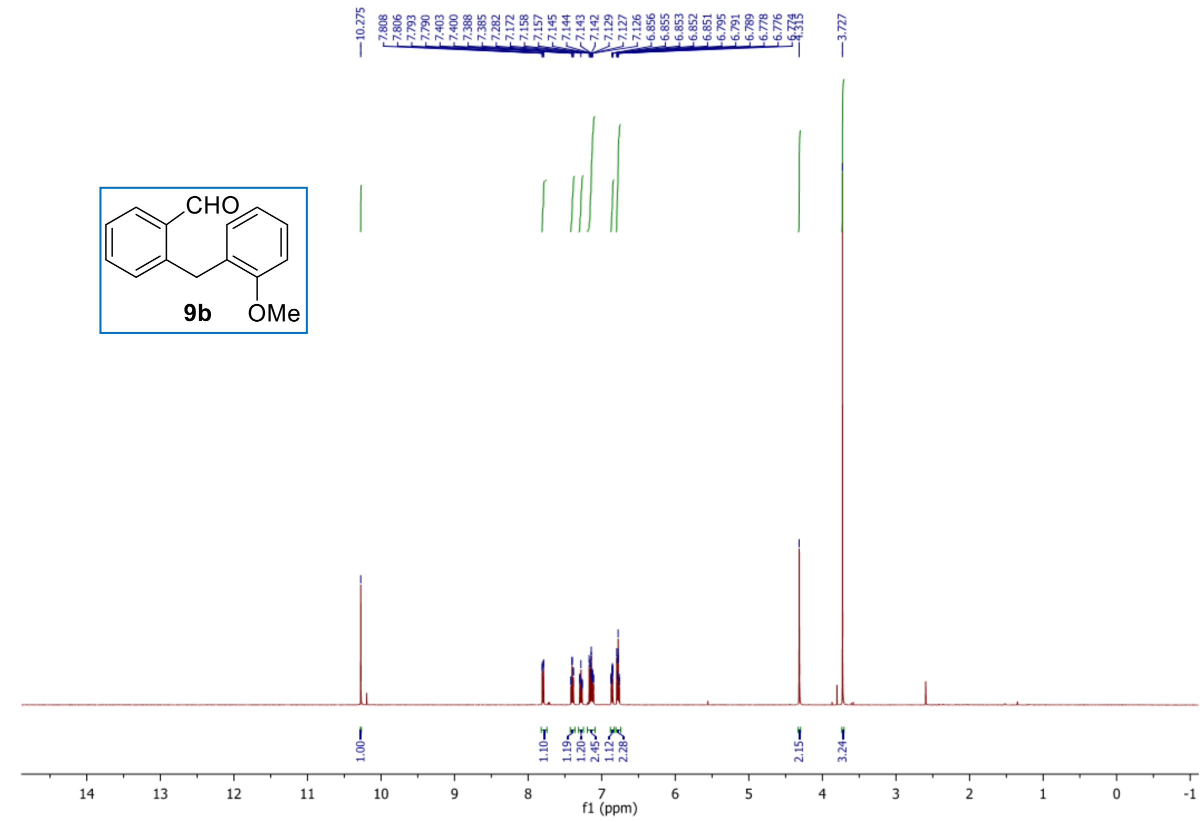

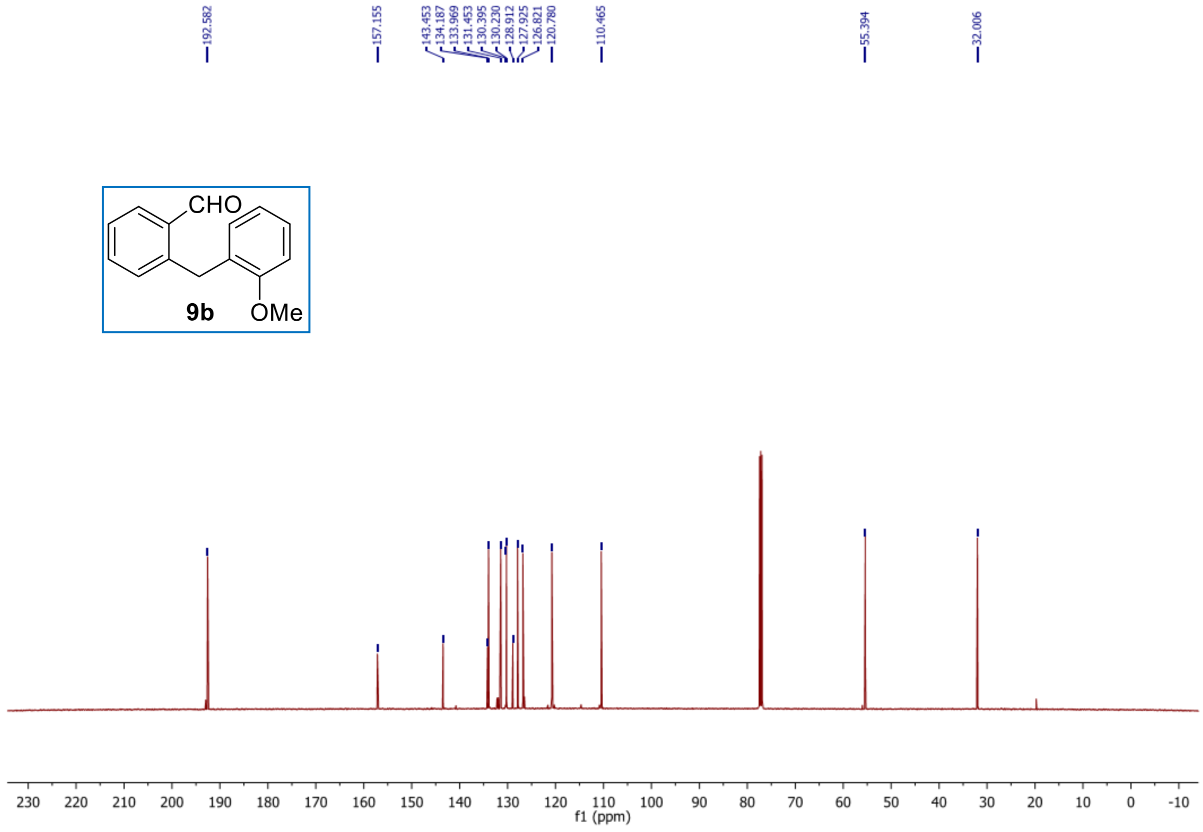

^{1}H and ^{13}C-NMR spectra of 2-methoxyanthracene (3c)

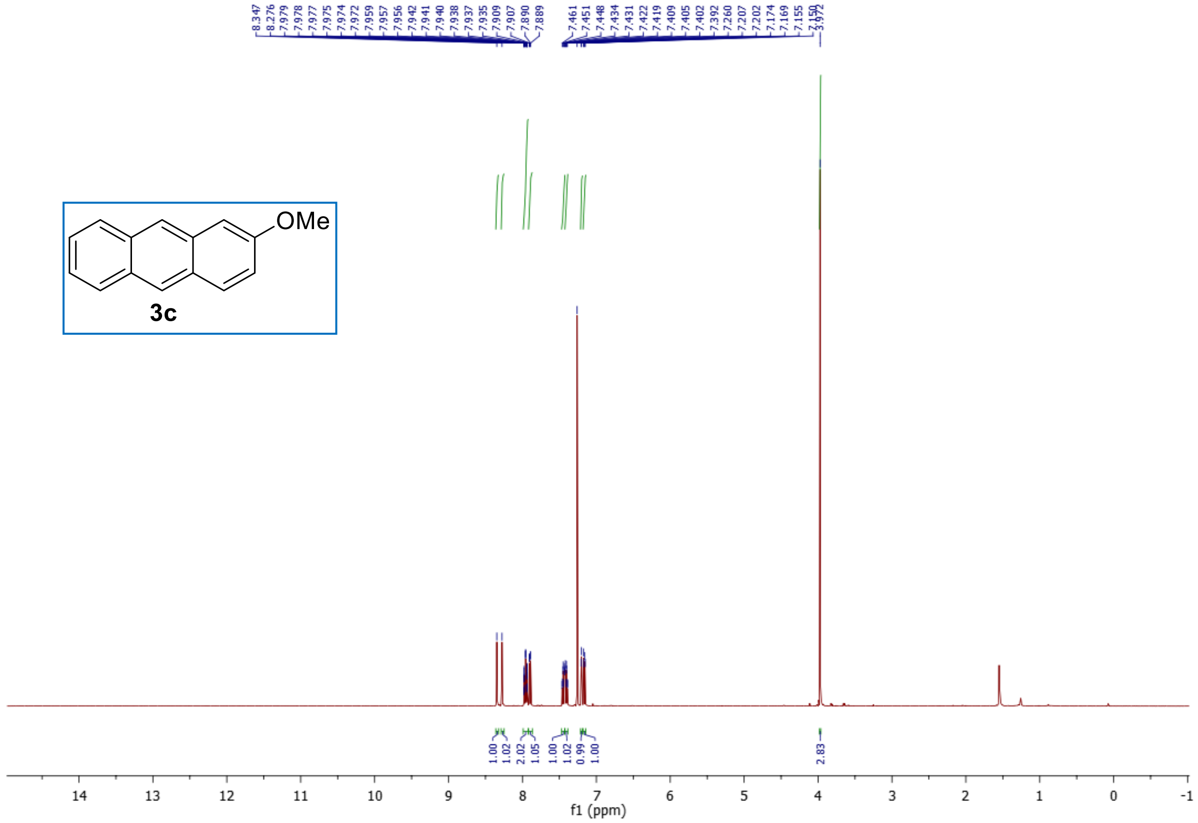

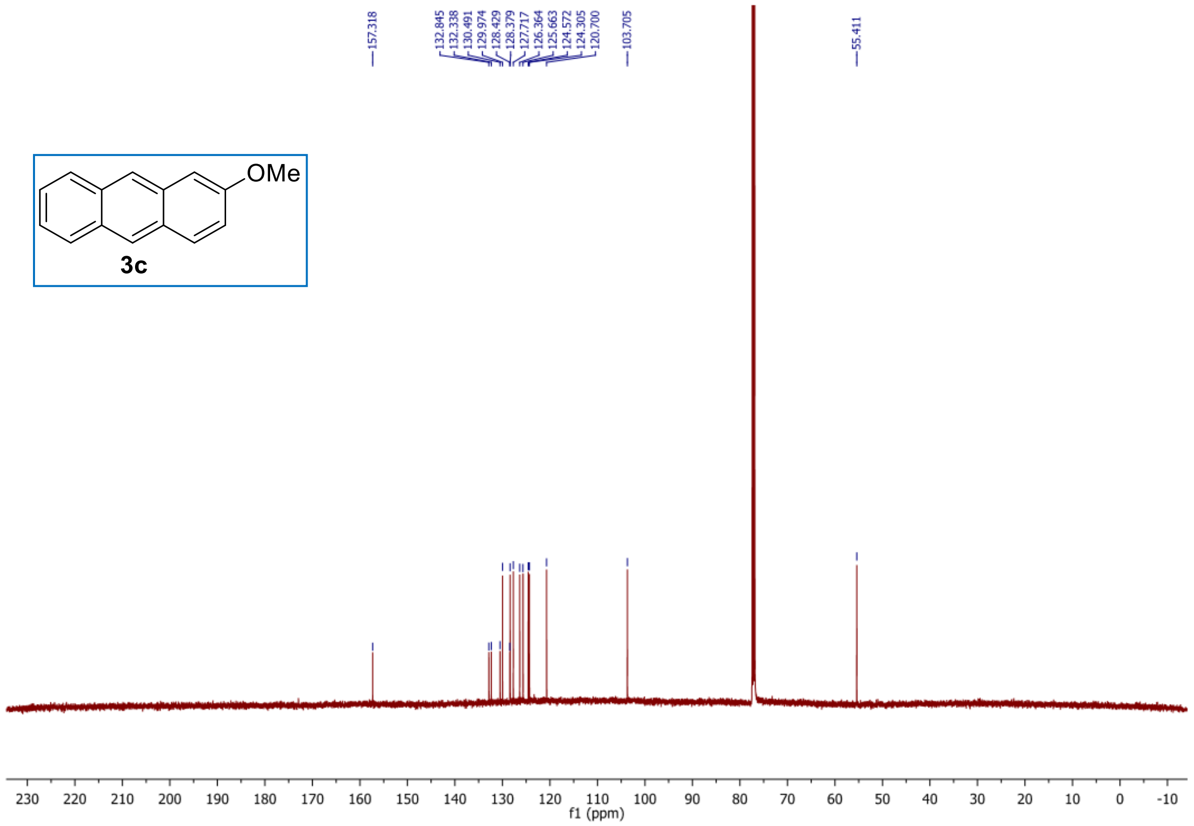

^{1}H and ^{13}C-NMR spectra of 1',4-dimethoxy-1,9'-bianthracene (3f)

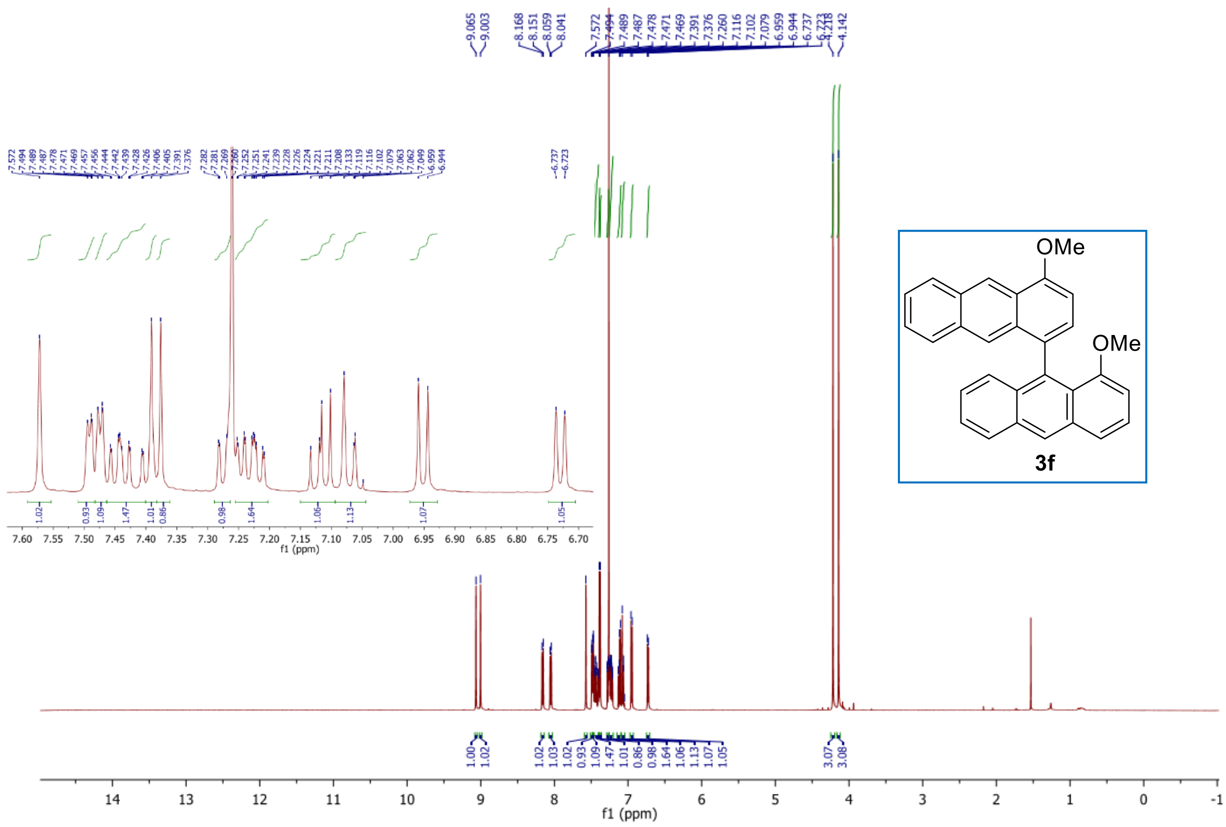

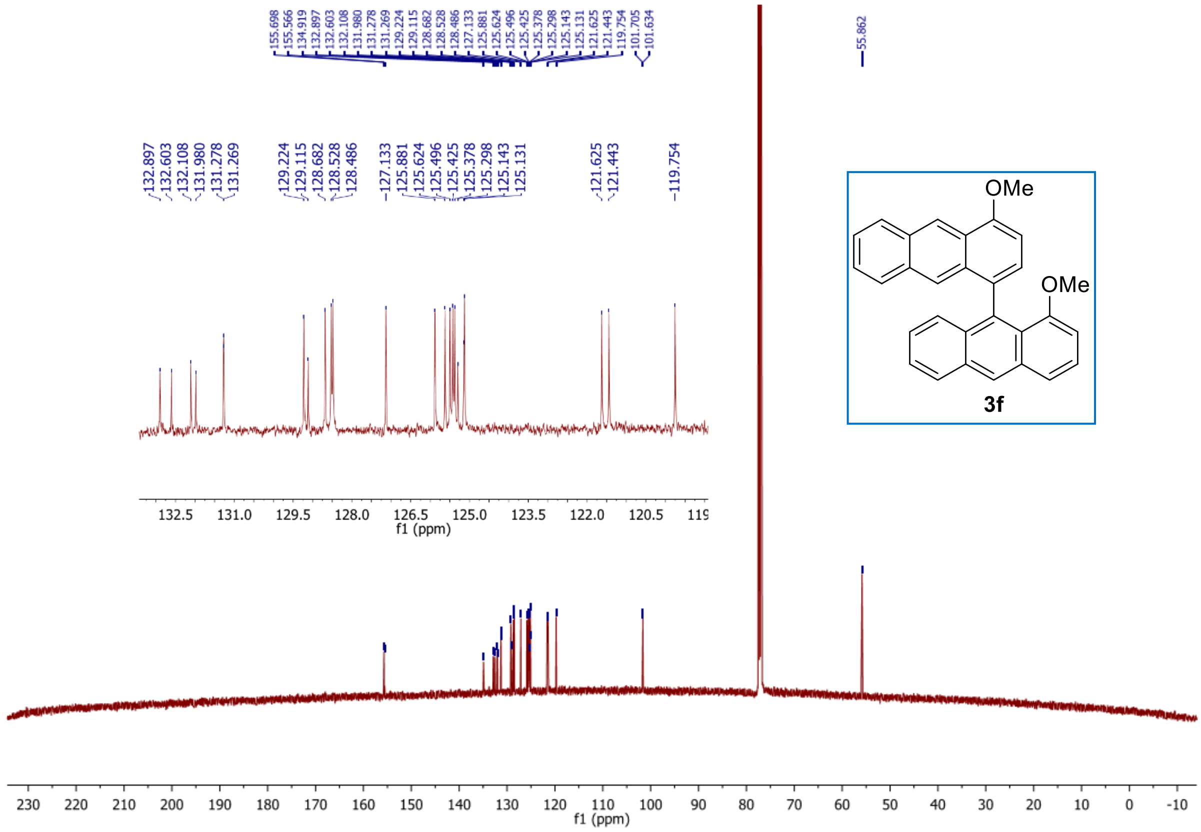

Mass spectra of 1',4-dimethoxy-1,9'-bianthracene (3f)

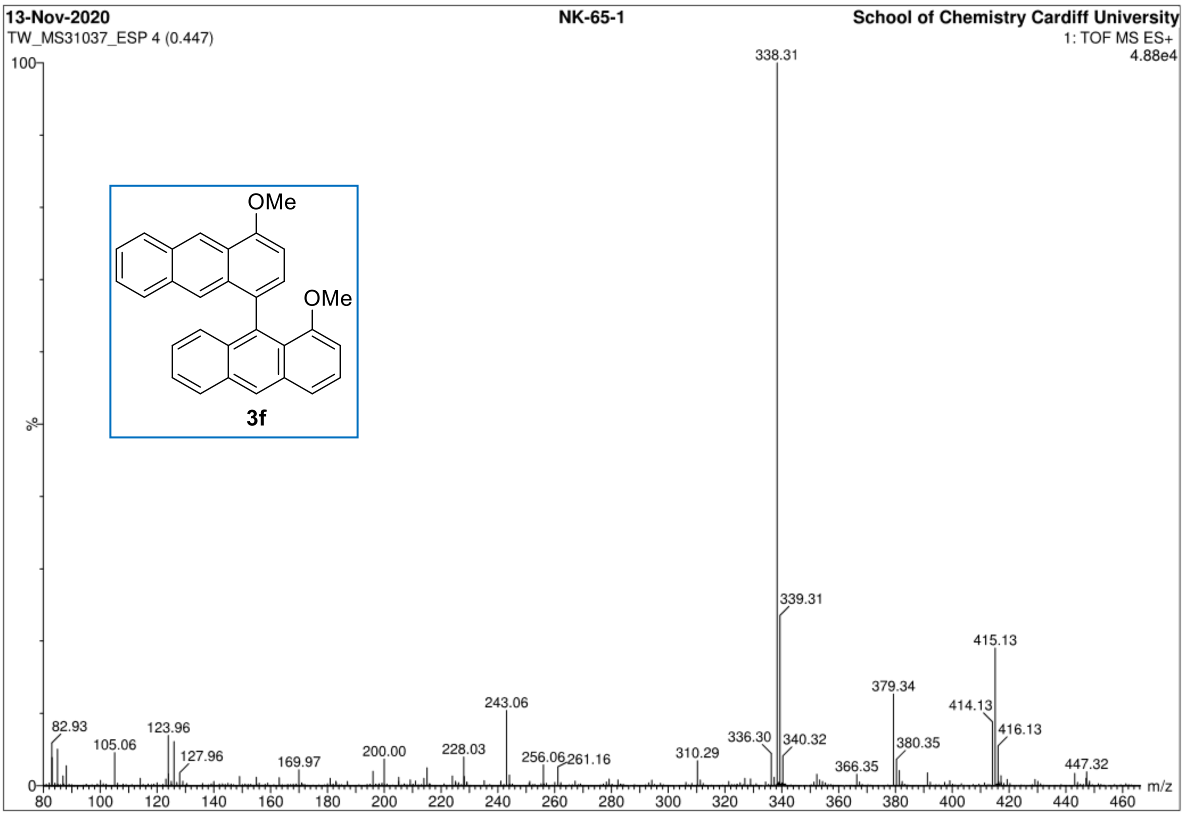

^{1}H and ^{13}C-NMR spectra of 1-methoxyanthracene-9,10-dione (11)

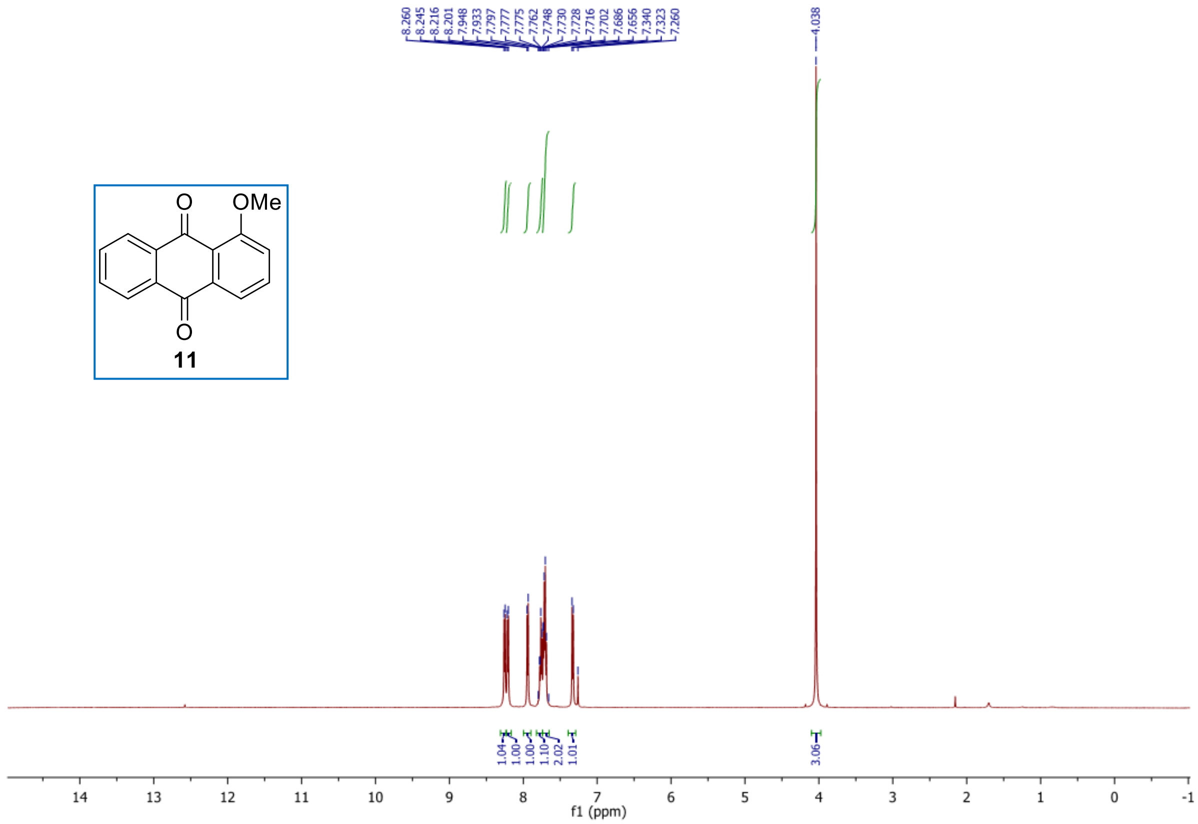

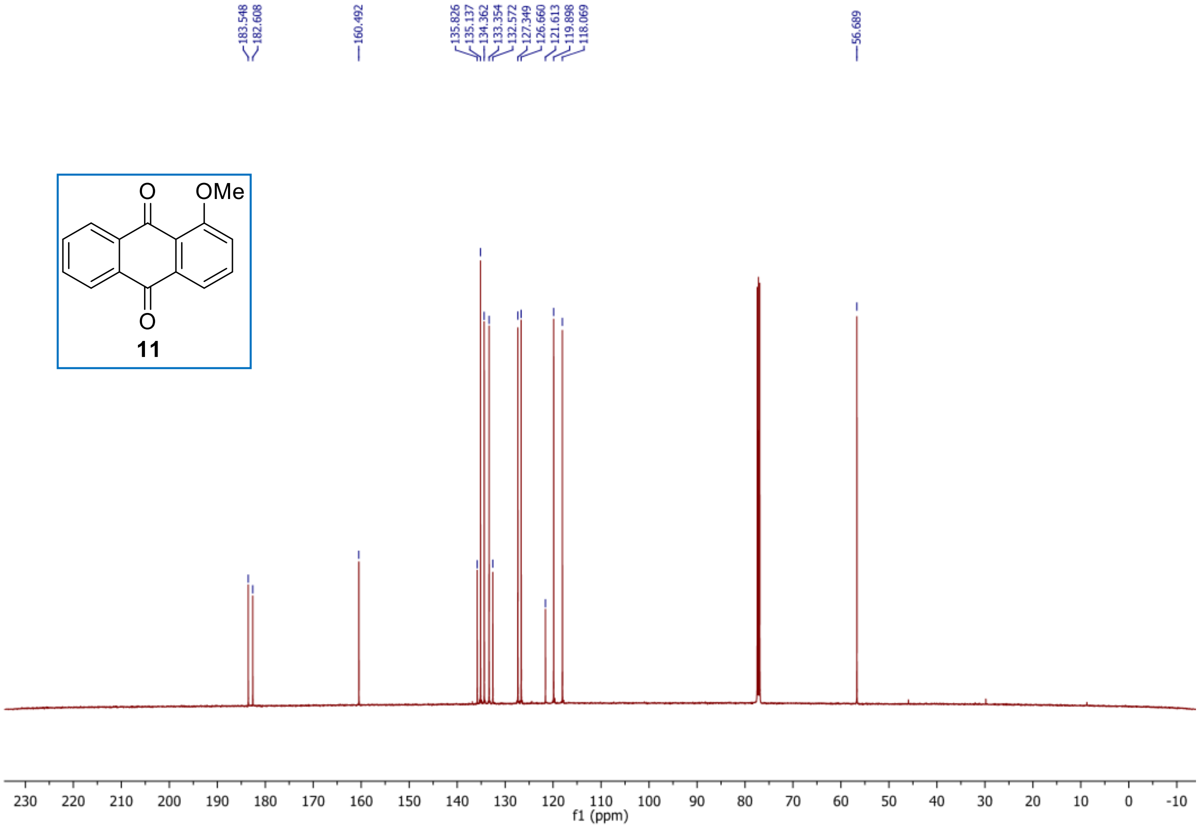

^{1}H and ^{13}C-NMR spectra of 1-methoxyanthracene (3d)

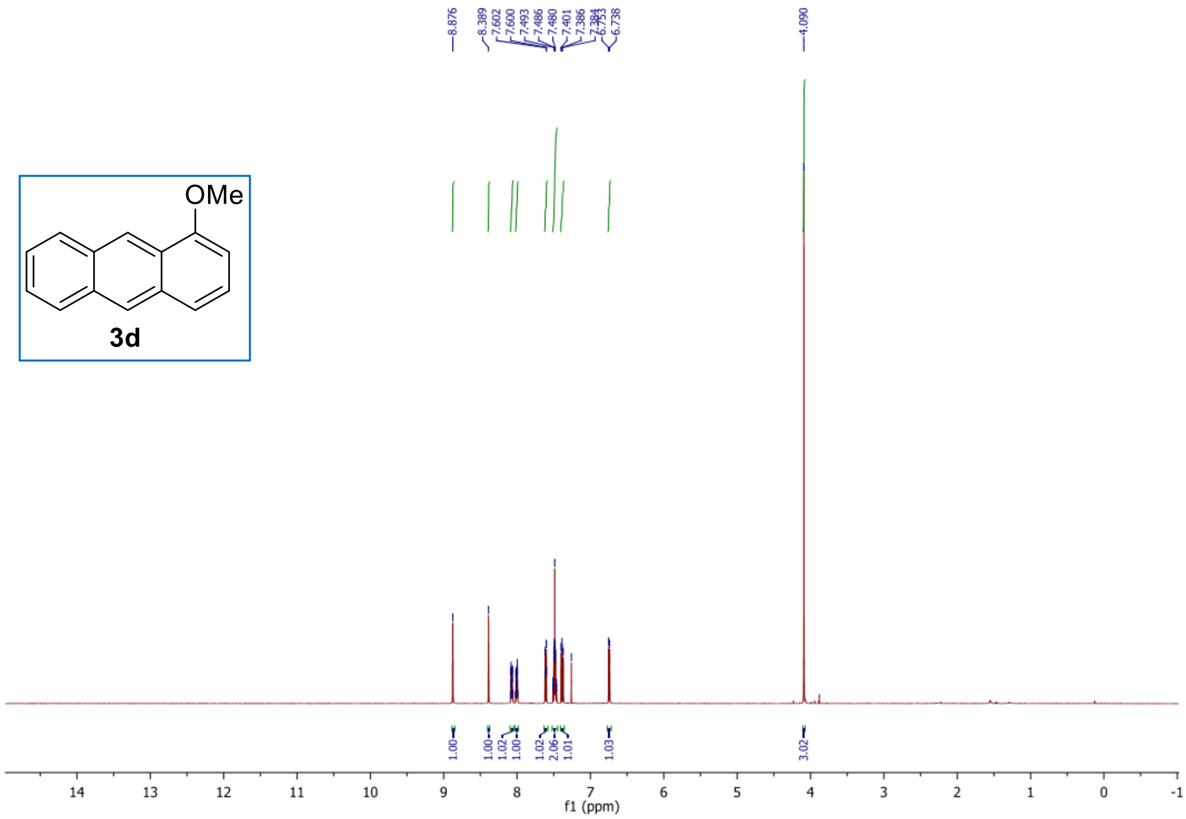

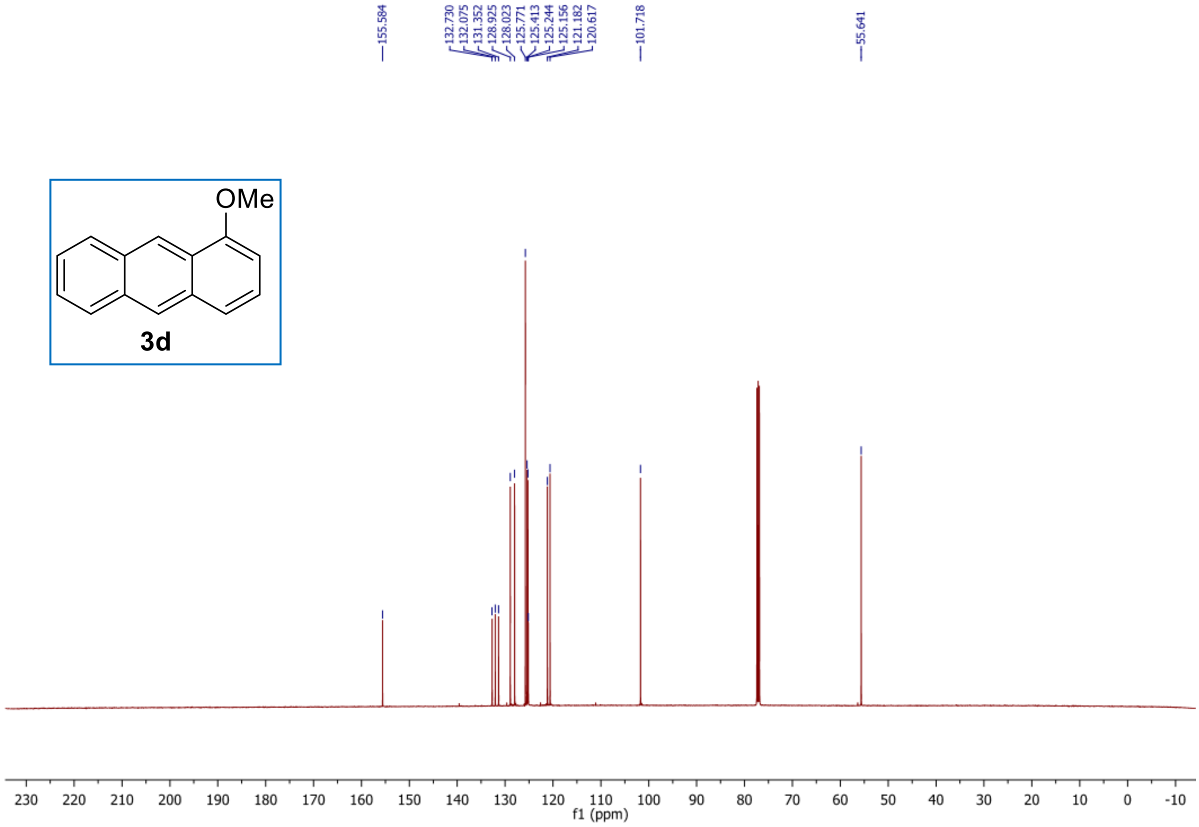

^{1}H and ^{13}C-NMR8 spectra of 1-iodo-8-methoxy-9,10-dihydro-9,10-[1,2]benzenoanthracene (*syn*-5e) + 1-iodo-5-methoxy-9,10-dihydro-9,10-[1,2]benzenoanthracene (*anti*-5e)

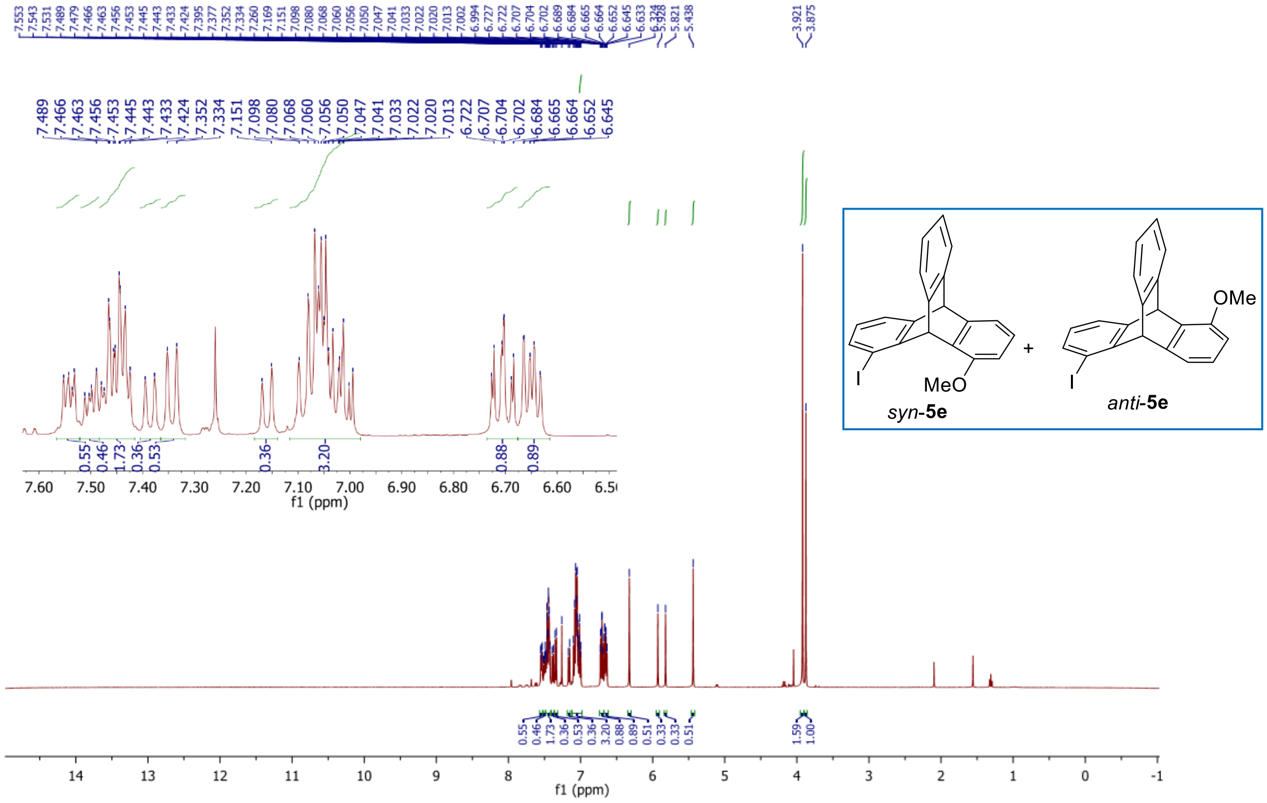

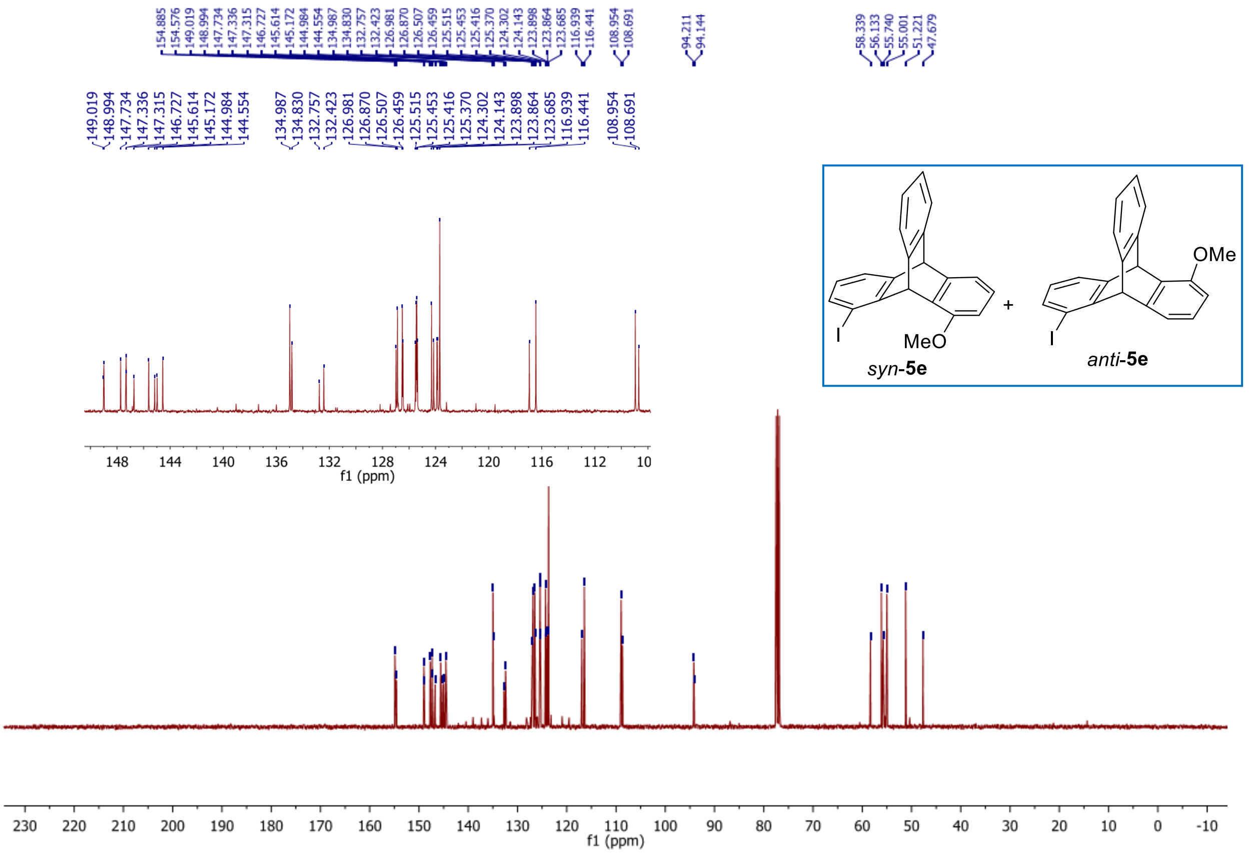

^{1}H and ^{13}C-NMR spectra of 1,4-dimethoxyanthracene-9,10-dione (12a)

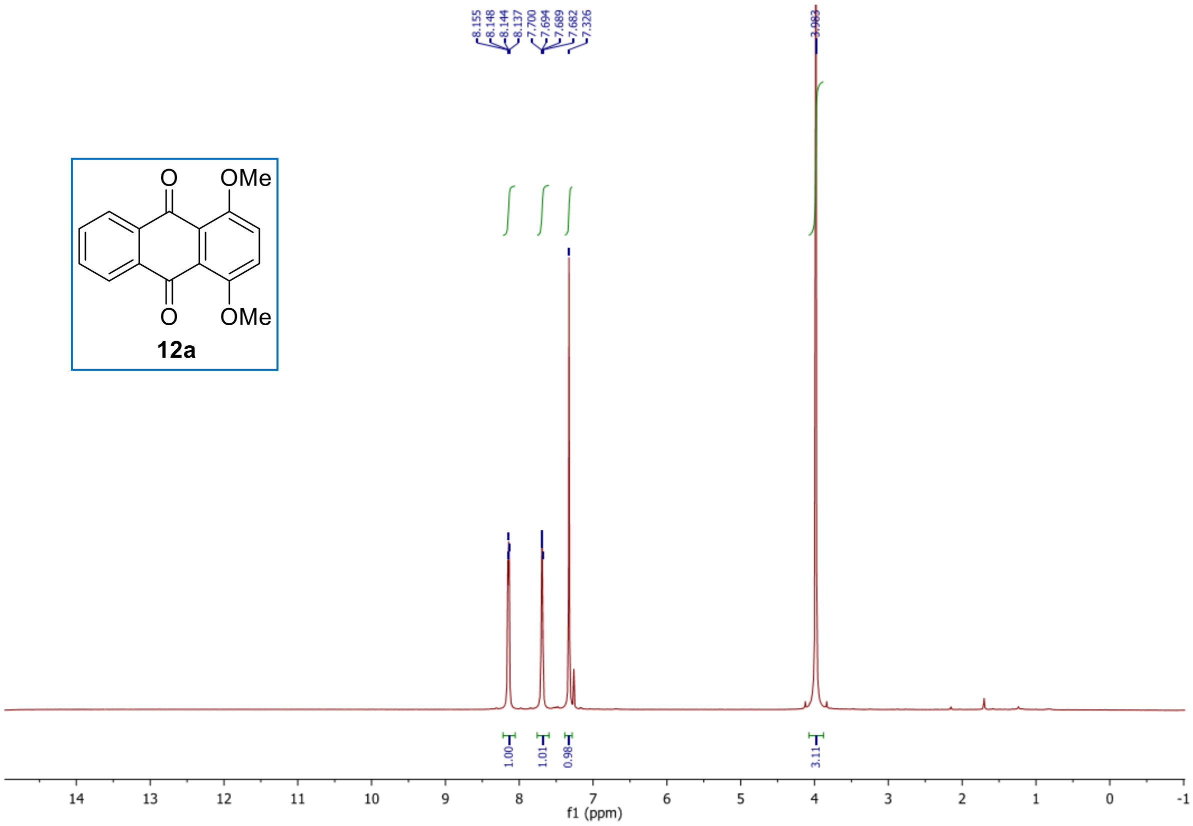

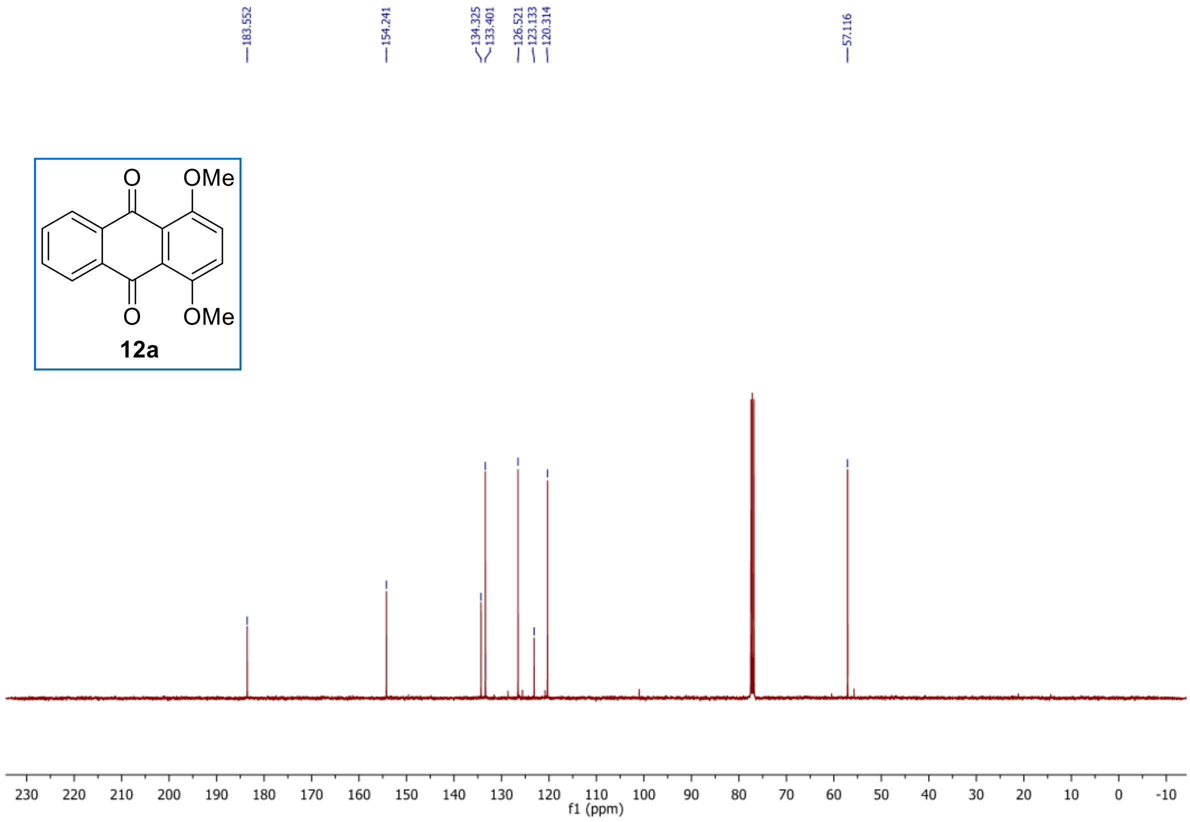

^{1}H and ^{13}C-NMR spectra of 1,4-dimethoxyanthracene (3e)

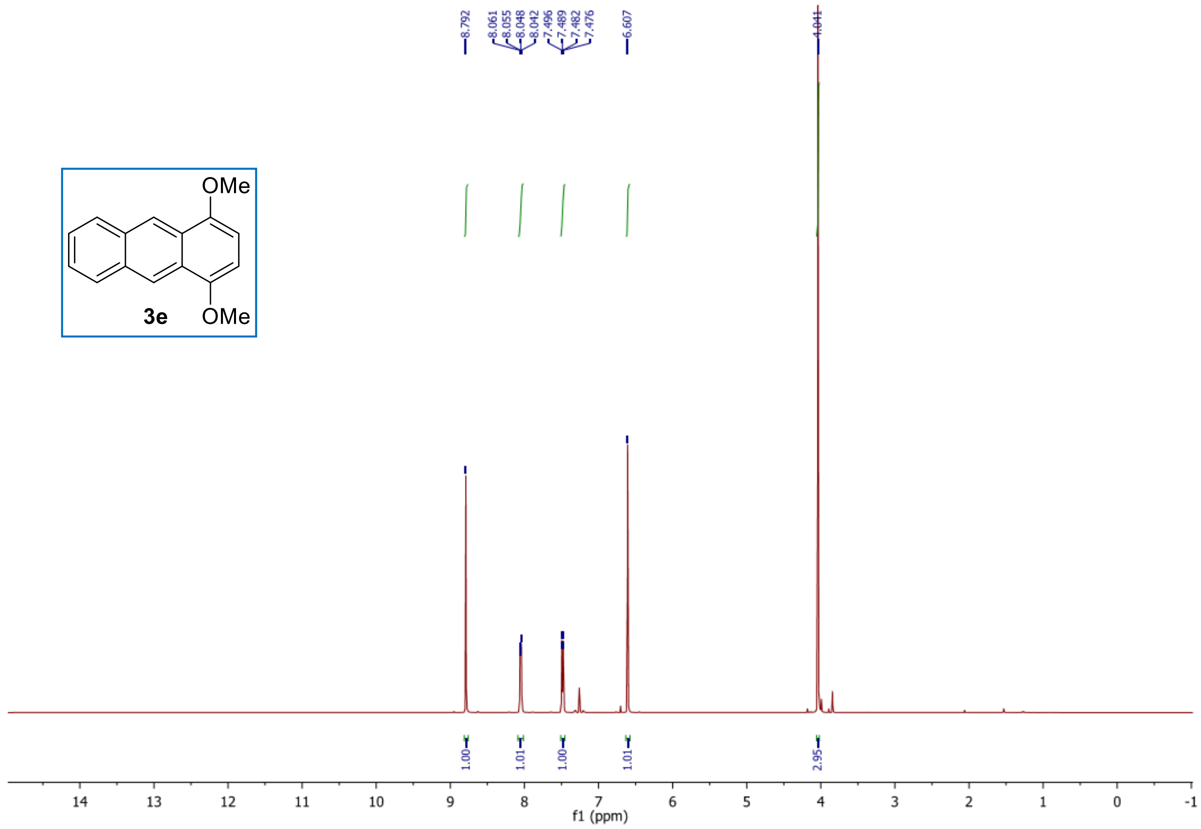

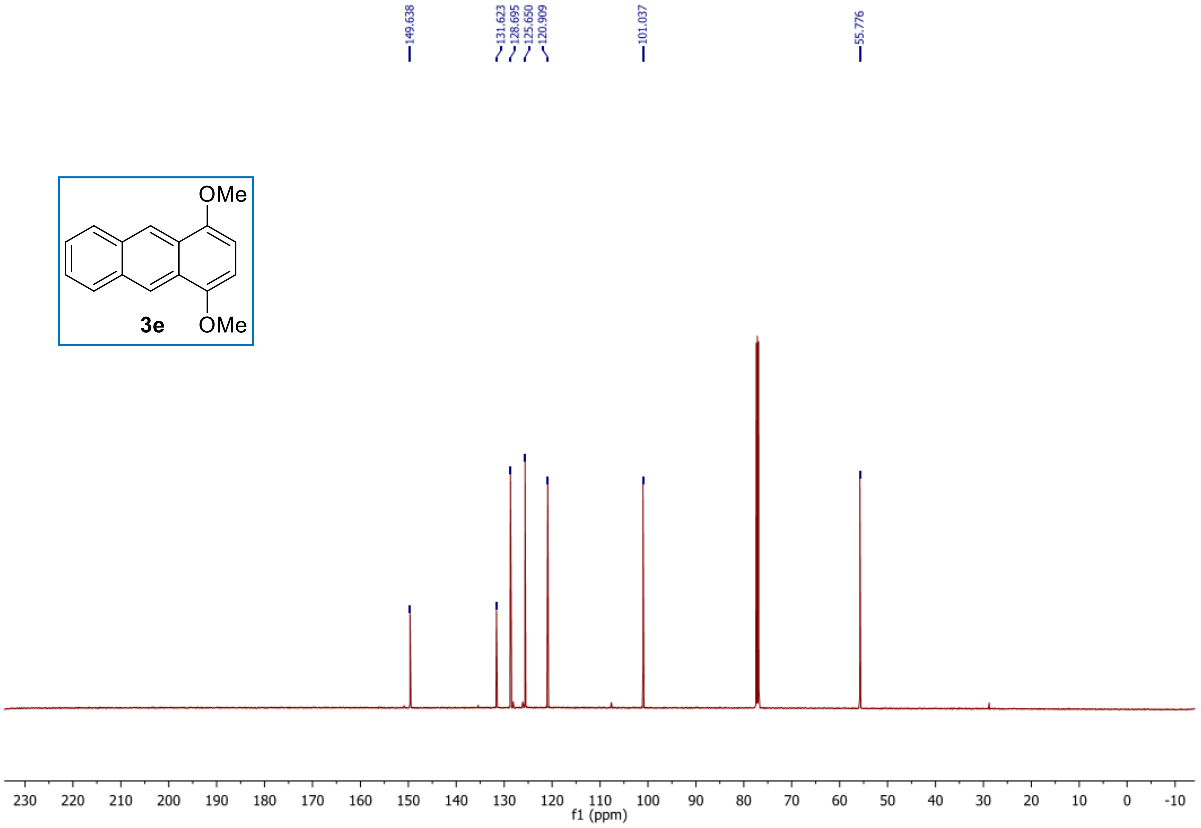

^{1}H and ^{13}C-NMR spectra of -iodo-1,4-dimethoxy-9,10-dihydro-9,10-[1,2]benzenoanthracene (5f)

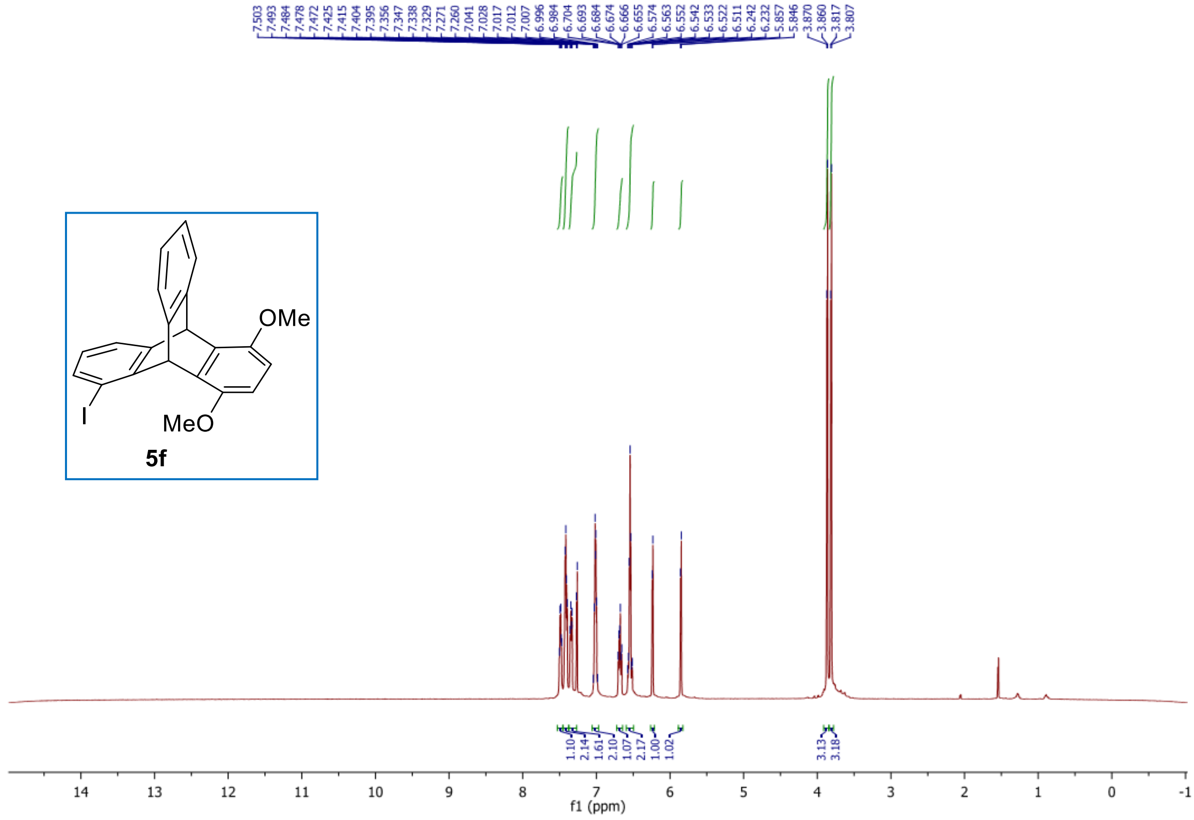

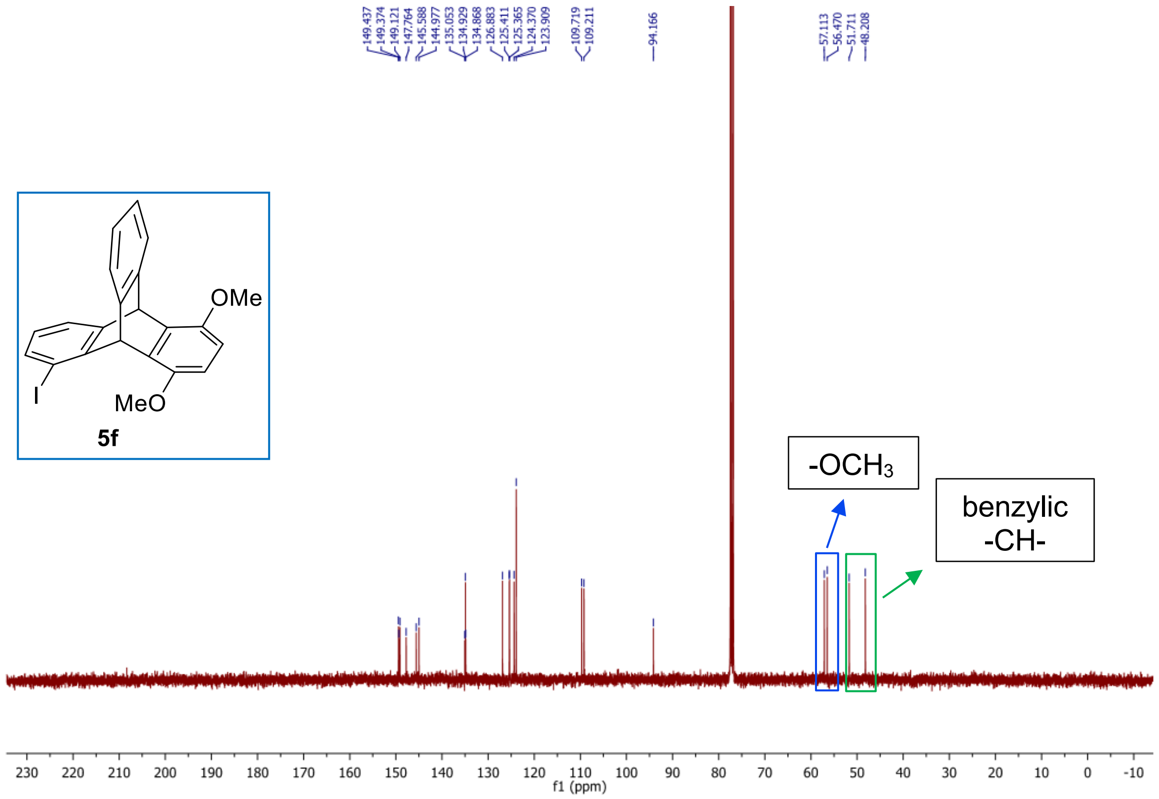

DEPT-90 spectra of 5-iodo-1,4-dimethoxy-9,10-dihydro-9,10-[1,2]benzenoanthracene (5f)

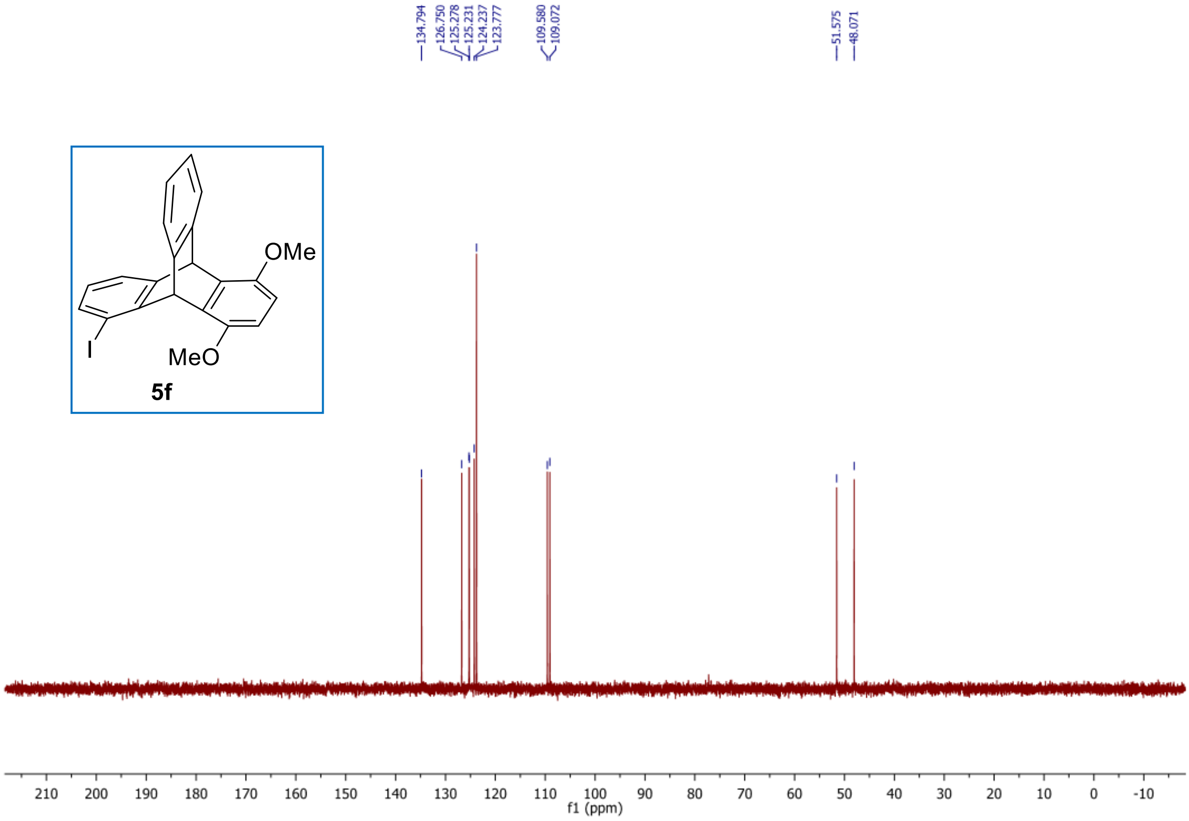

Mass spectra of 5-iodo-1,4-dimethoxy-9,10-dihydro-9,10-[1,2]benzenoanthracene (5f)

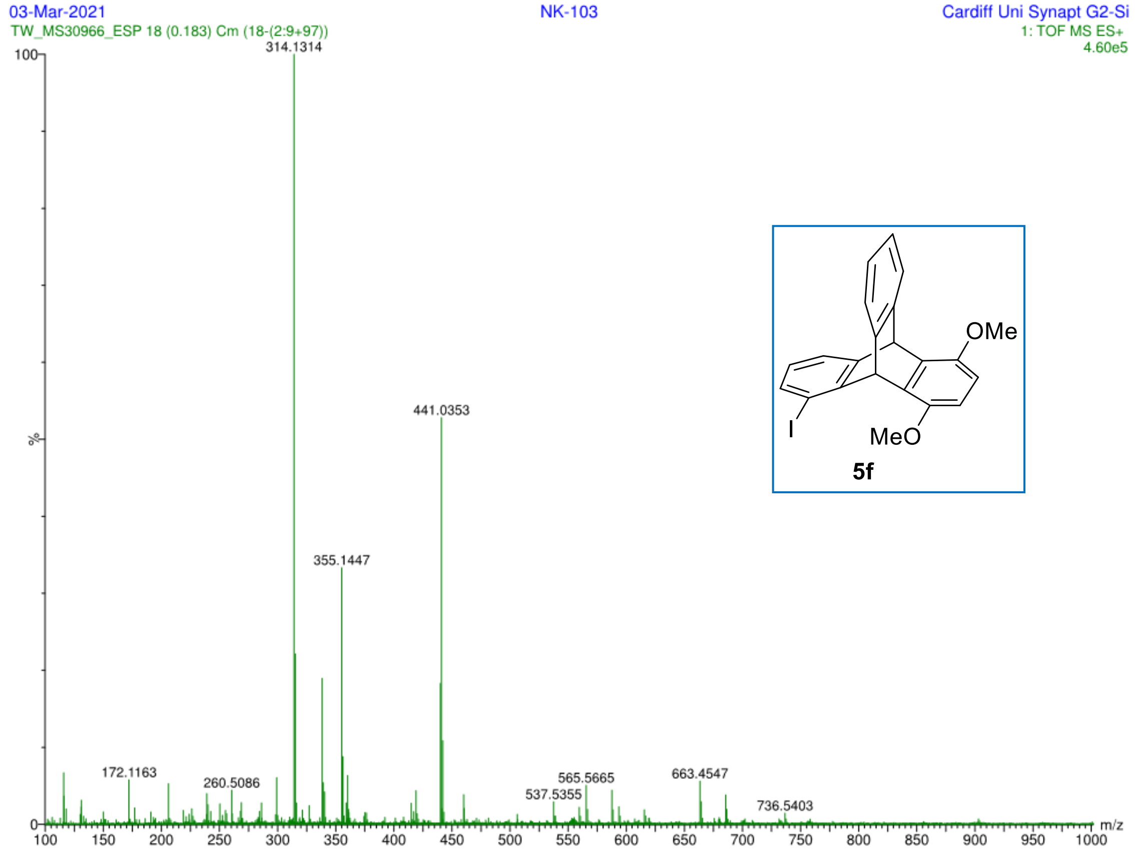

Elemental Composition Report

Page 1

Single Mass Analysis

Tolerance = 5.0 PPM / DBE: min = -1.5, max = 50.0
Element prediction: Off
Number of isotope peaks used for i-FIT = 3

Monoisotopic Mass, Odd and Even Electron Ions
4 formula(e) evaluated with 1 results within limits (up to 50 closest results for each mass)
Elements Used:
C: 0-22 H: 0-18 O: 0-2 127I: 0-1

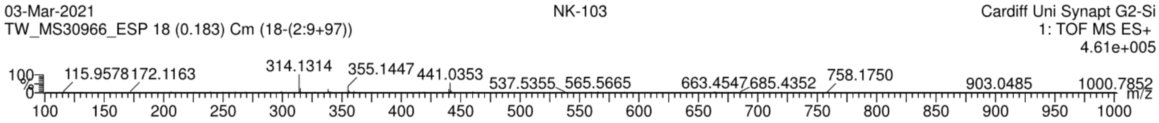

Minimum:				-1.5				
Maximum:		20.0	5.0	50.0				
Mass	Calc. Mass	mDa	PPM	DBE	i-FIT	Norm	Conf(%)	Formula
441.0353	441.0351	0.2	0.5	13.5	589.6	n/a	n/a	C22 H18 O2 127I

^{1}H and ^{13}C-NMR spectra of 5-iodo-9,10-dihydro-9,10-[1,2]benzenoanthracene-1,4-diol (13)

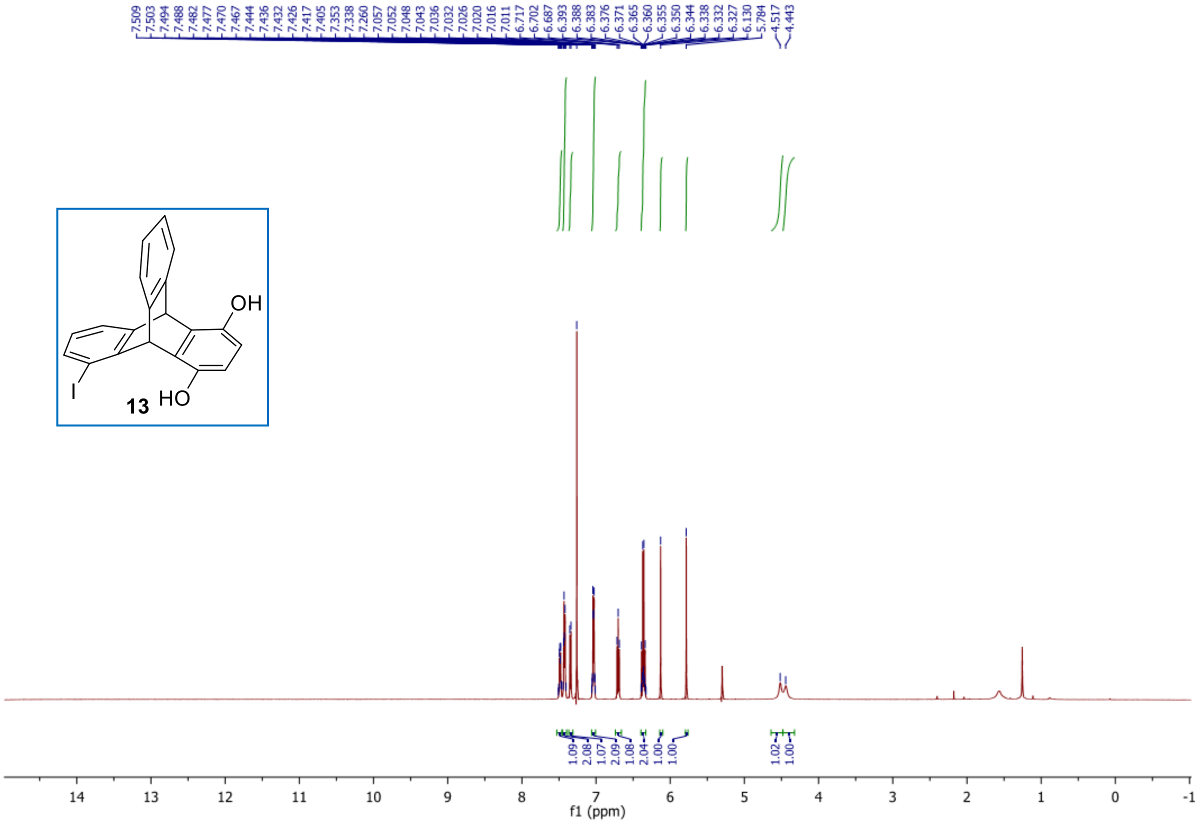

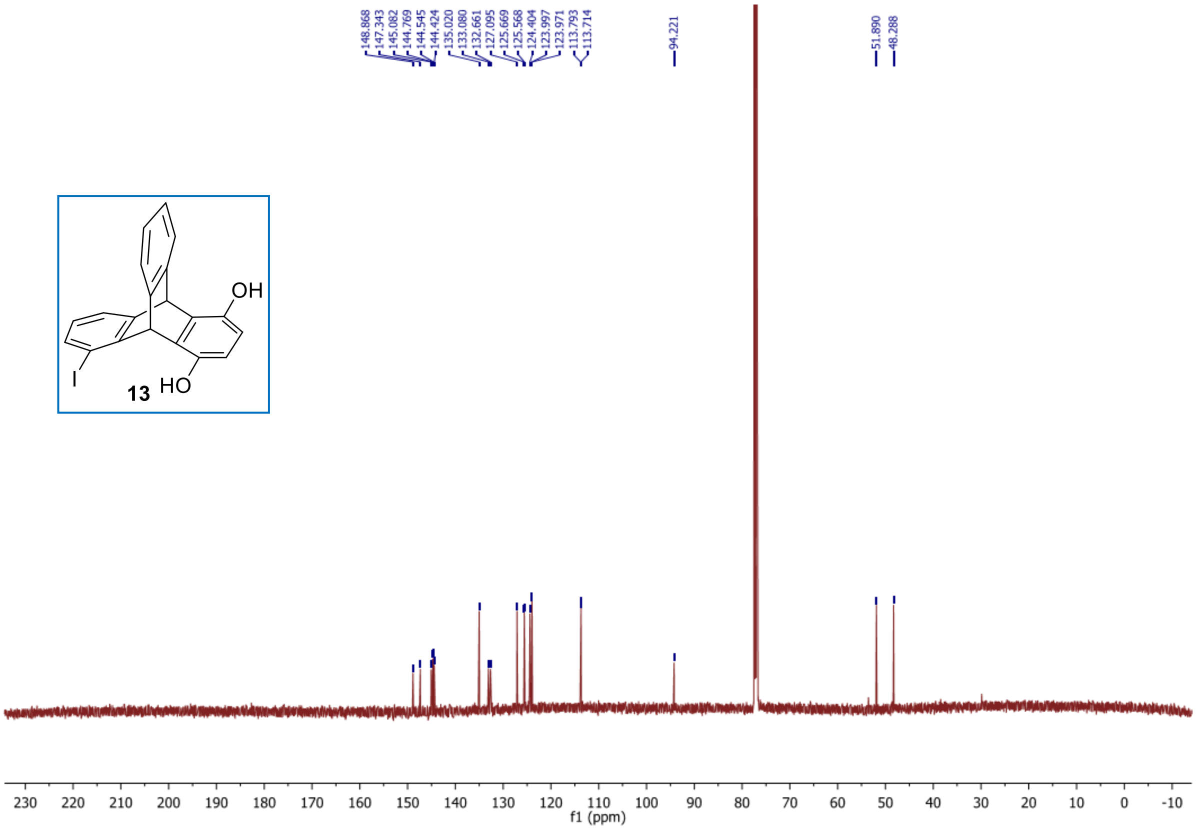

Mass spectra of 5-iodo-9,10-dihydro-9,10-[1,2]benzenoanthracene-1,4-diol (13)

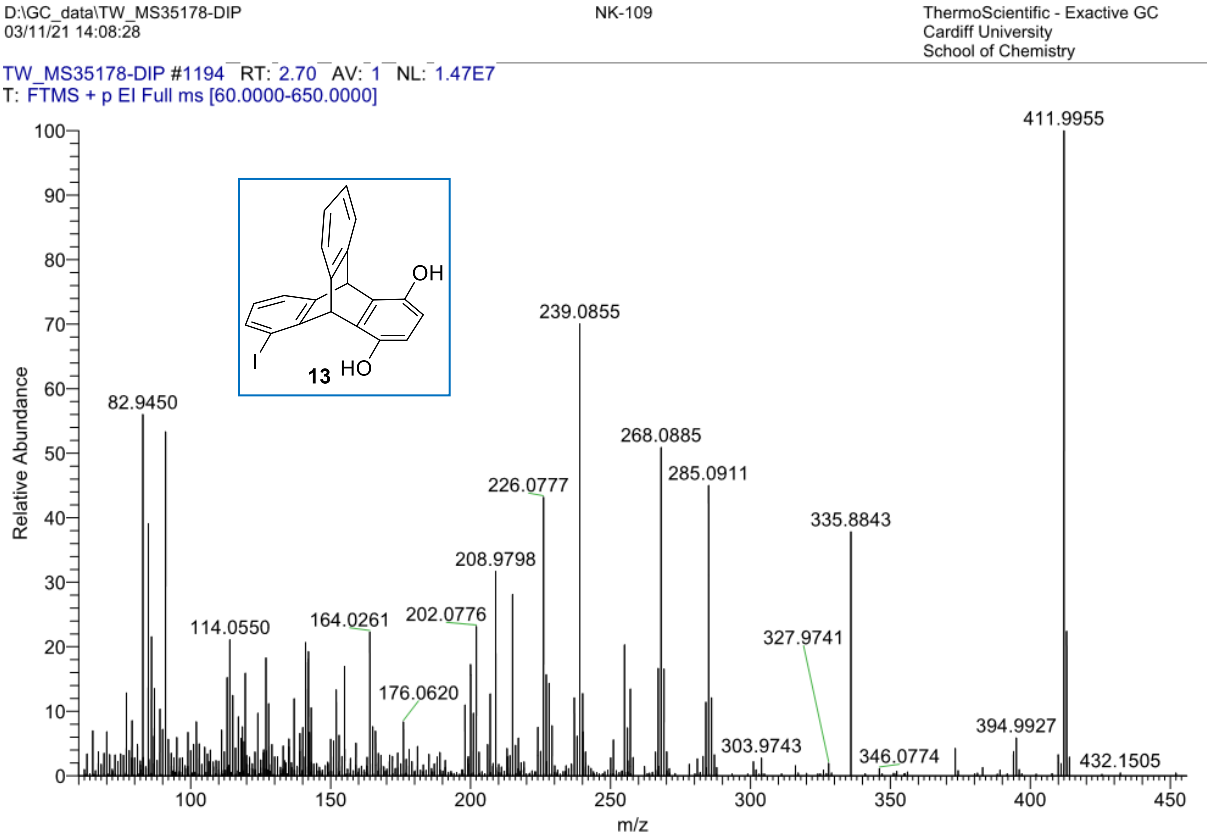

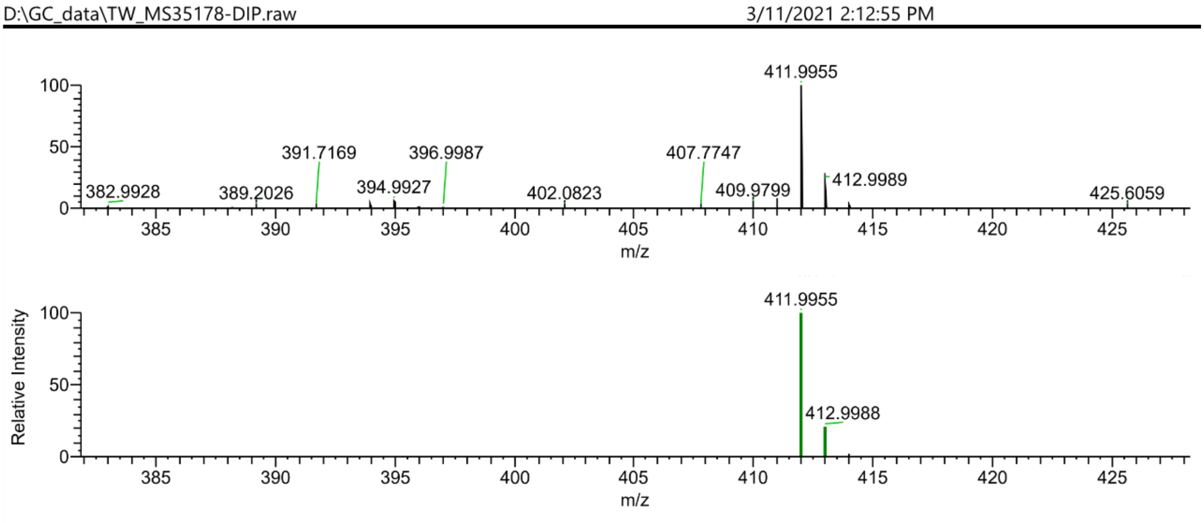

Peak Mass	Display Formula	Delta [ppm]	Theo. mass	Combined Score
411.9955	$C_{20}H_{13}O_2{}^{127}I$	0.10	411.99547	97.2515223513496

^{1}H and ^{13}C-NMR spectra of 5-iodo-9,10-dihydro-9,10-[1,2]benzenoanthracene-1,4-diyl (1S,1'S,4S,4'S)-bis(4,7,7-trimethyl-3-oxo-2-oxabicyclo[2.2.1]heptane-1-carboxylate) (16)

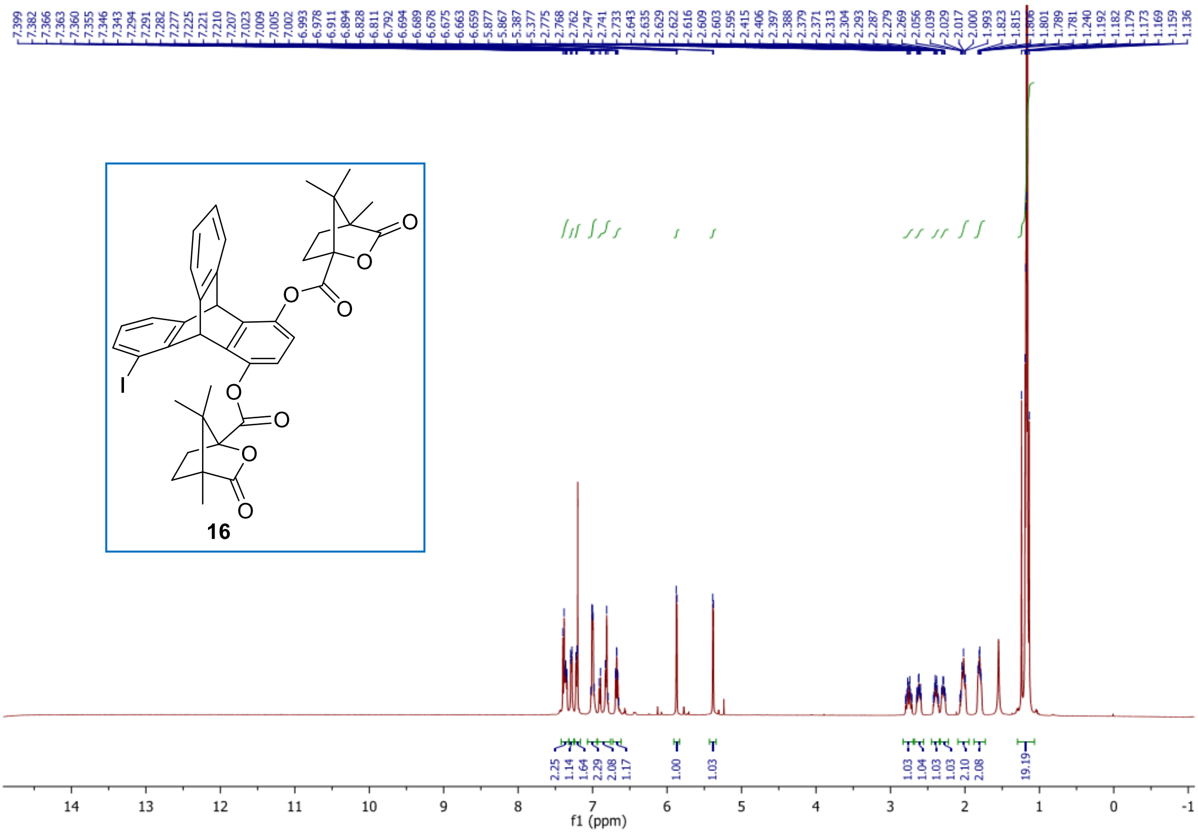

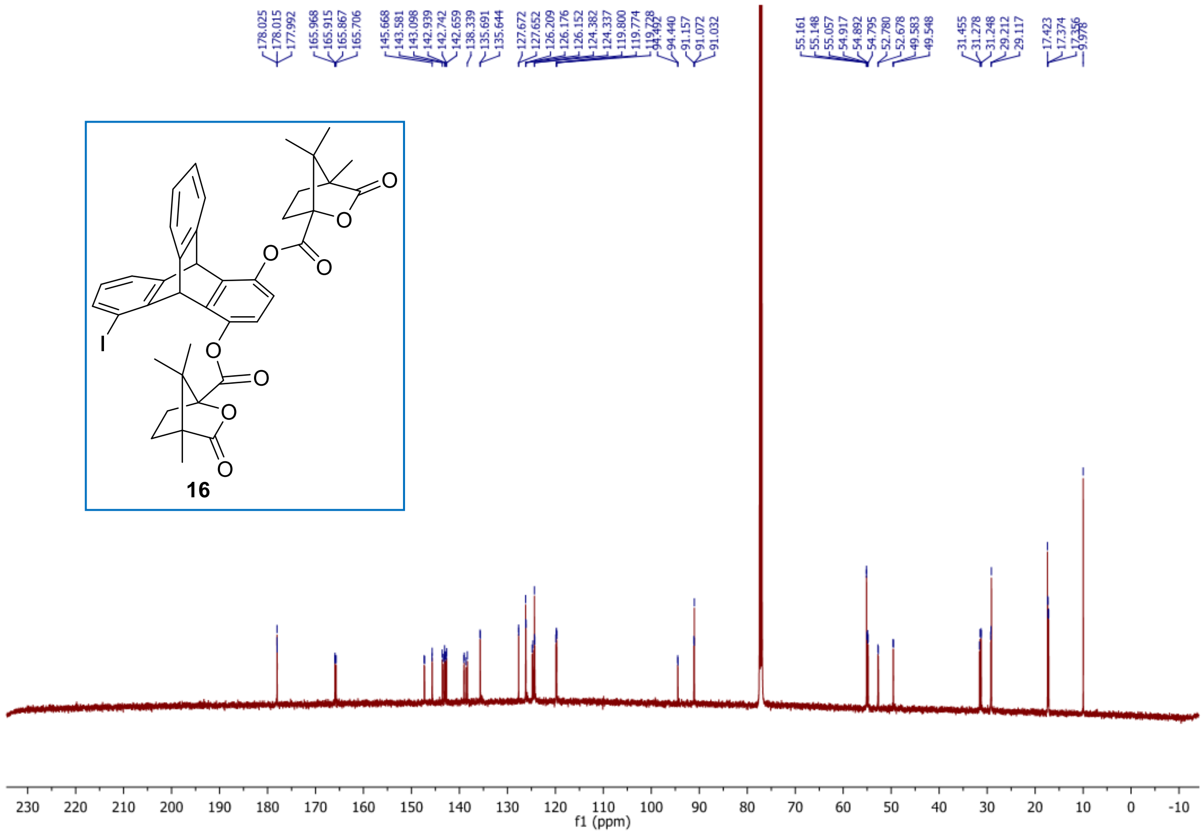

^{1}H and ^{13}C-NMR spectra of 5-iodo-9,10-dihydro-9,10-[1,2]benzenoanthracene-1,4-diyl (1S,1'S,4S,4'S)-bis(4,7,7-trimethyl-3-oxo-2-oxabicyclo[2.2.1]heptane-1-carboxylate) [(-)-16)]

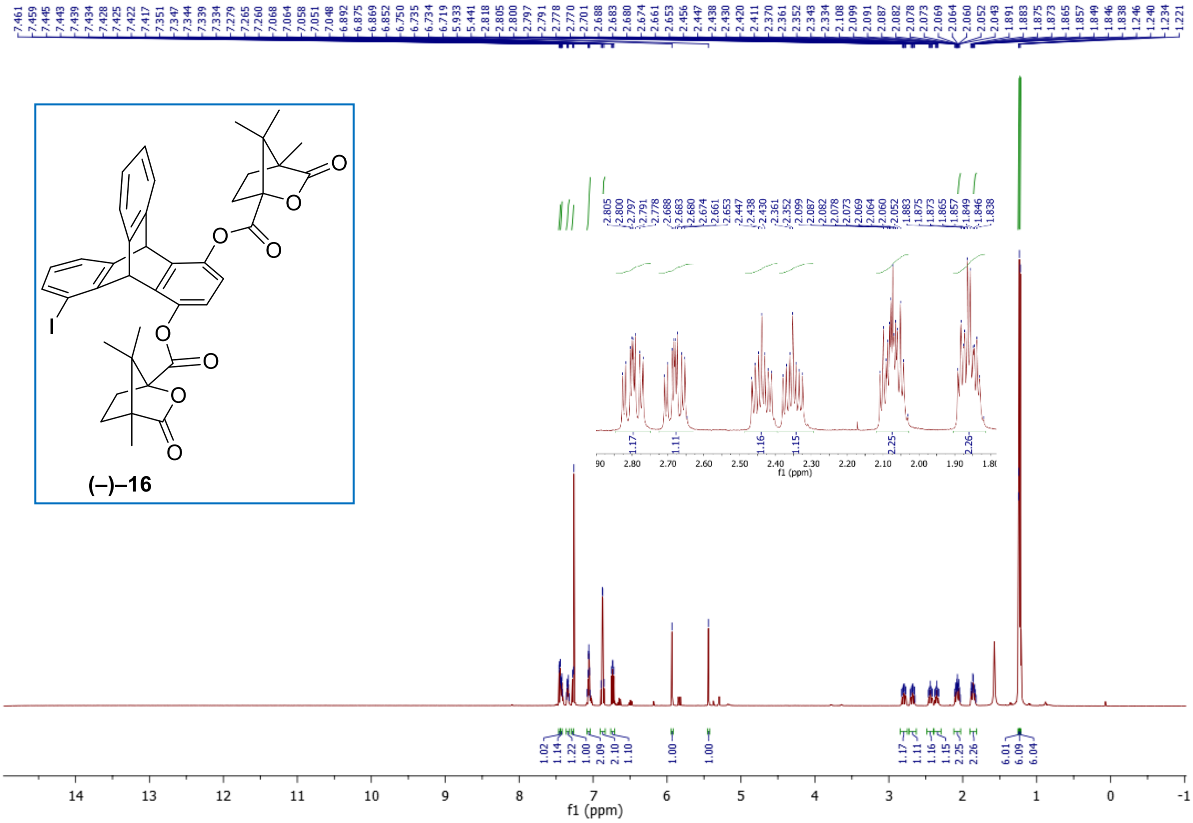

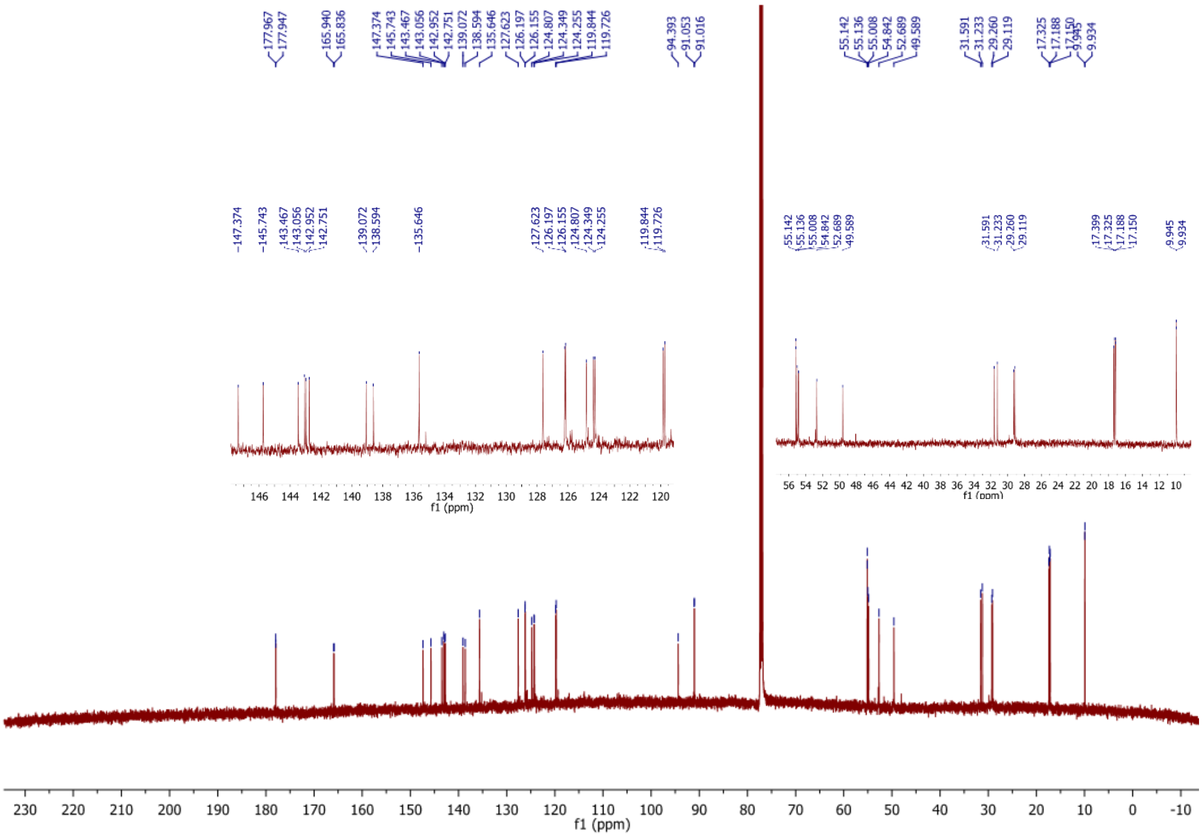

Mass spectra of 5-iodo-9,10-dihydro-9,10-[1,2]benzenoanthracene-1,4-diyl (1S,1'S,4S,4'S)-bis(4,7,7-trimethyl-3-oxo-2-oxabicyclo[2.2.1]heptane-1-carboxylate) [(-)-16)]

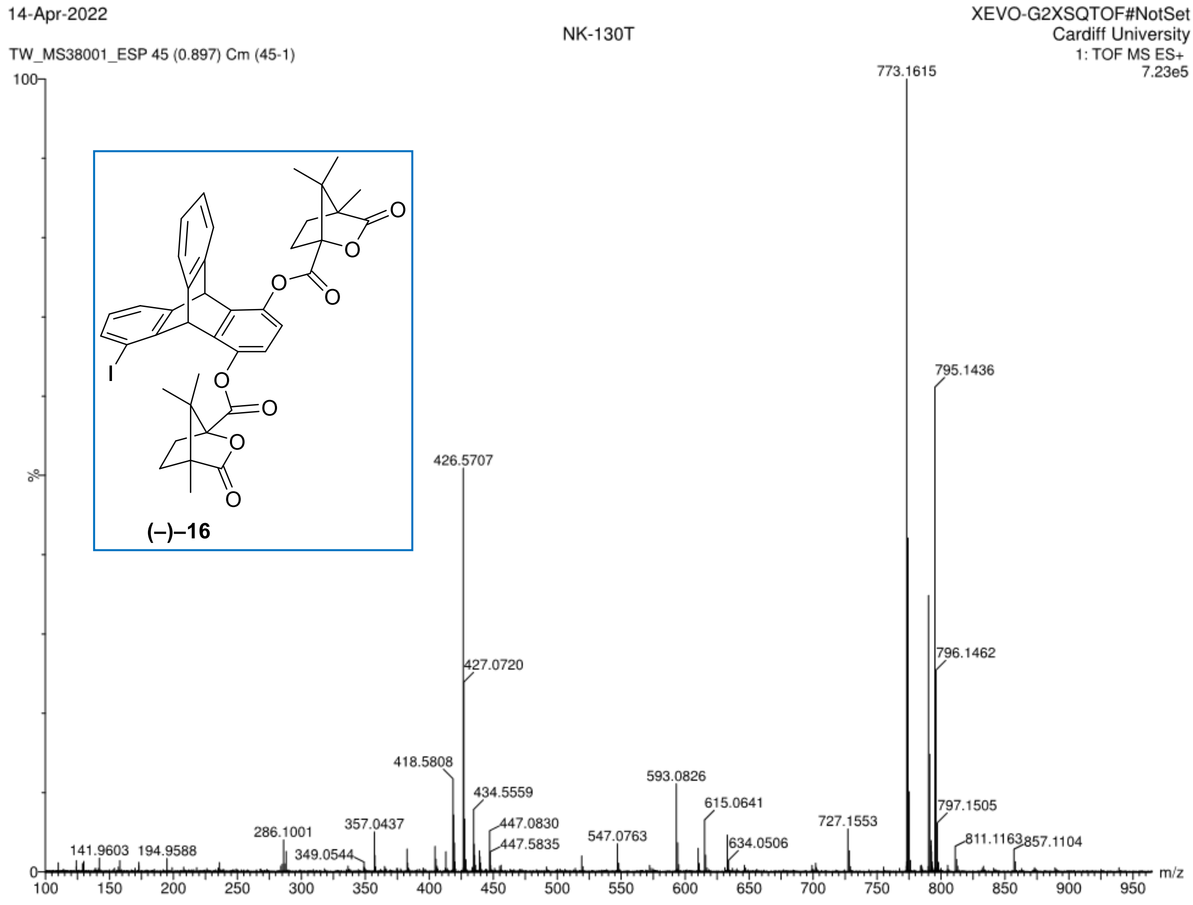

Elemental Composition Report

Page 1

Single Mass Analysis

Tolerance = 5.0 PPM / DBE: min = -1.5, max = 100.0
Element prediction: Off
Number of isotope peaks used for i-FIT = 3

Monoisotopic Mass, Odd and Even Electron Ions
15 formula(e) evaluated with 1 results within limits (up to 50 closest results for each mass)
Elements Used:
C: 0-40 H: 0-38 O: 0-8 I: 0-1

Minimum:				-1.5				
Maximum:		5.0	5.0	100.0				
Mass	Calc. Mass	mDa	PPM	DBE	i-FIT	Norm	Conf(%)	Formula
773.1615	773.1611	0.4	0.5	21.5	441.6	n/a	n/a	C40 H38 O8 I

^{1}H and ^{13}C-NMR spectra of 5-iodo-9,10-dihydro-9,10-[1,2]benzenoanthracene-1,4-diyl (1S,1'S,4S,4'S)-bis(4,7,7-trimethyl-3-oxo-2-oxabicyclo[2.2.1]heptane-1-carboxylate) [(+)–16]

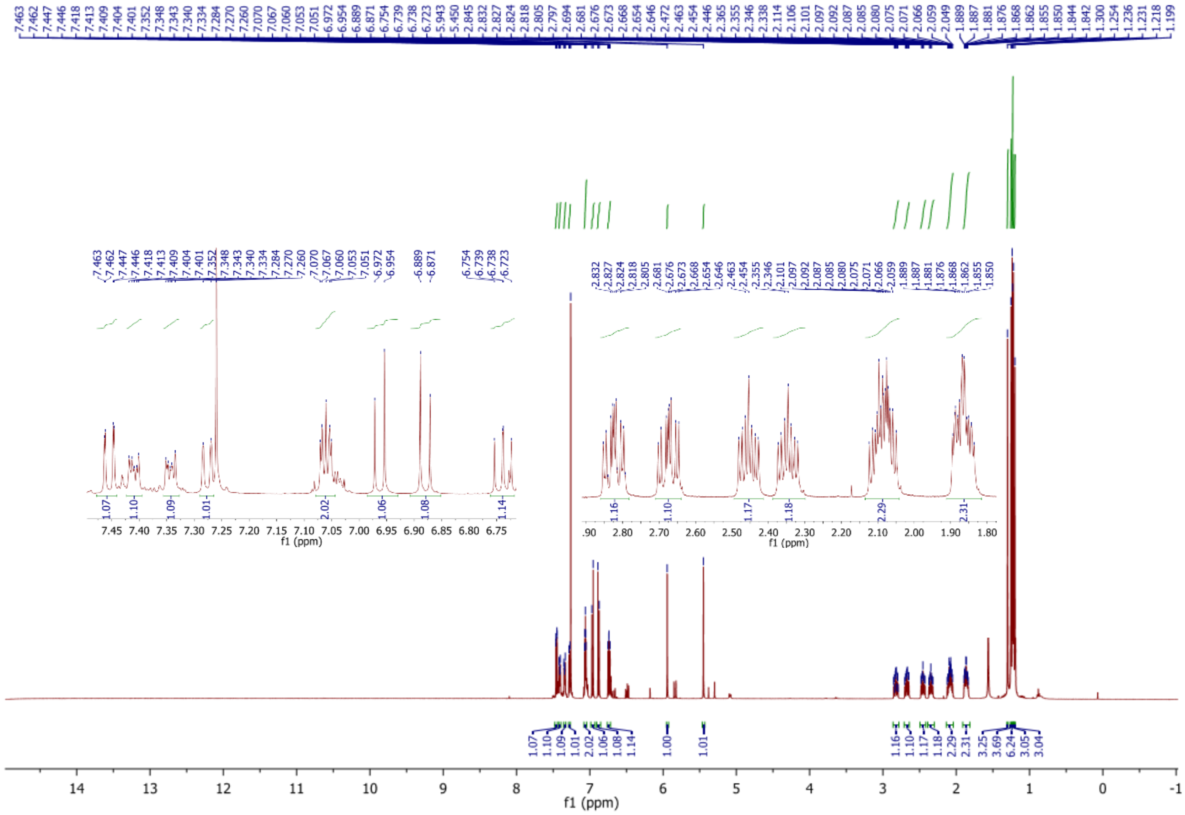

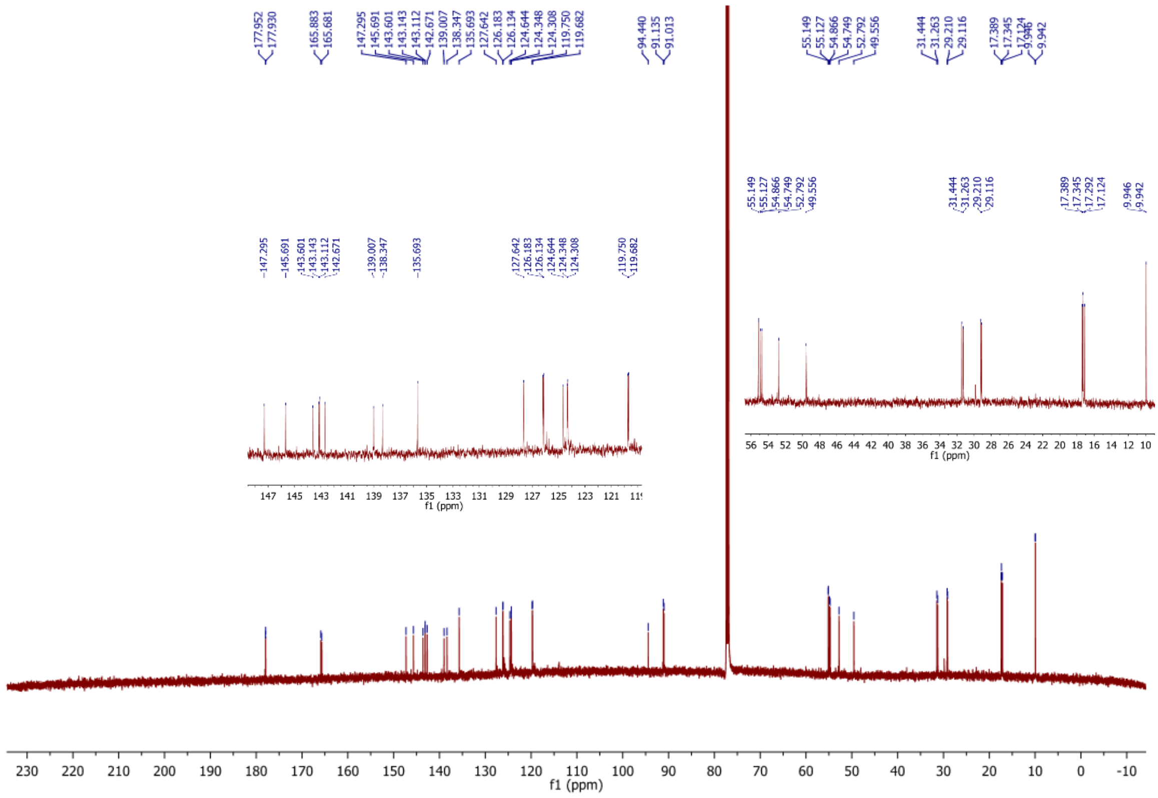

Mass spectra of 5-iodo-9,10-dihydro-9,10-[1,2]benzenoanthracene-1,4-diyl (1S,1'S,4S,4'S)-bis(4,7,7-trimethyl-3-oxo-2-oxabicyclo[2.2.1]heptane-1-carboxylate) [(+)–16]

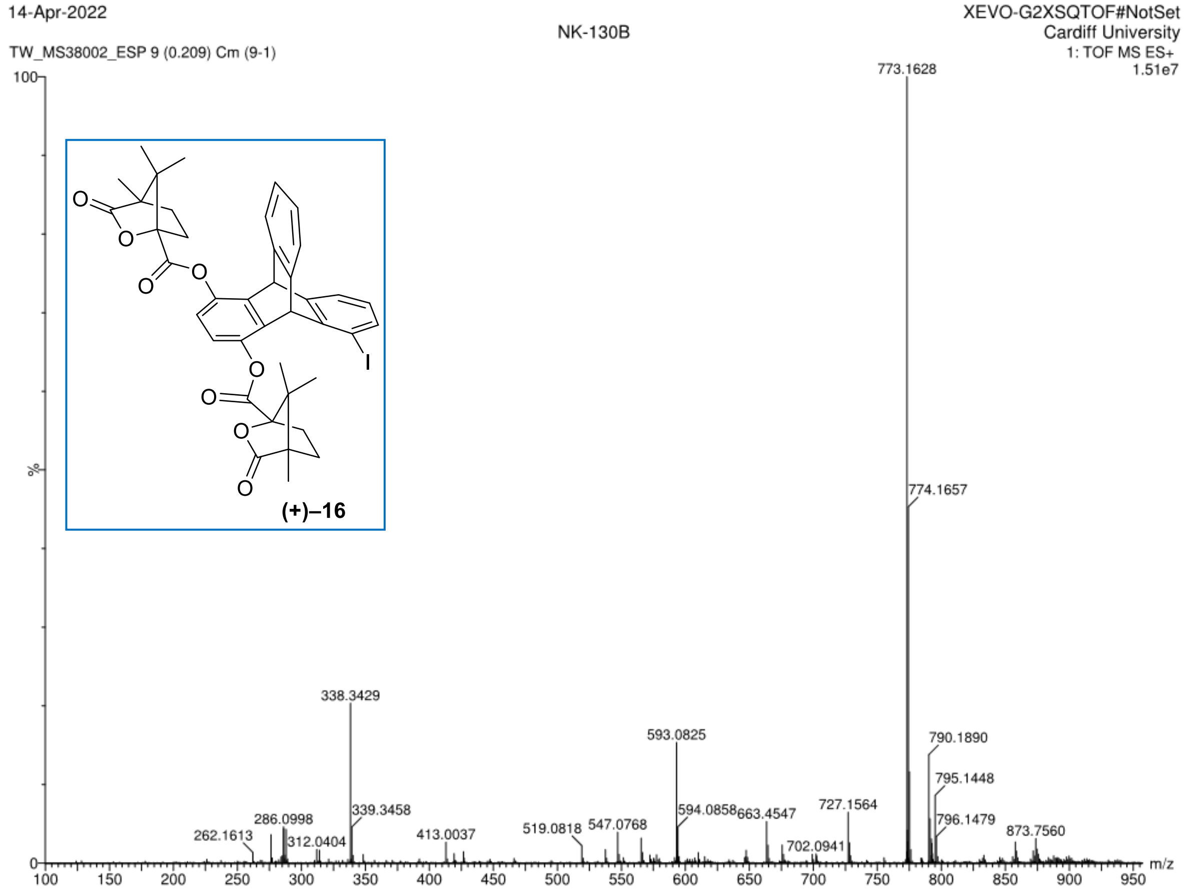

Elemental Composition Report

Page 1

Single Mass Analysis

Tolerance = 5.0 PPM / DBE: min = -1.5, max = 100.0
Element prediction: Off
Number of isotope peaks used for i-FIT = 3

Monoisotopic Mass, Even Electron Ions
15 formula(e) evaluated with 1 results within limits (up to 50 closest results for each mass)
Elements Used:
C: 0-40 H: 0-38 O: 0-8 I: 0-1

Minimum:				-1.5				
Maximum:		5.0	5.0	100.0				
Mass	Calc. Mass	mDa	PPM	DBE	i-FIT	Norm	Conf(%)	Formula
773.1628	773.1611	1.7	2.2	21.5	656.5	n/a	n/a	C40 H38 O8 I

^{1}H and ^{13}C-NMR spectra of 1-iodo-9,10-dihydro-9,10-[1,2]benzenoanthracene-13,16-dione (13a)

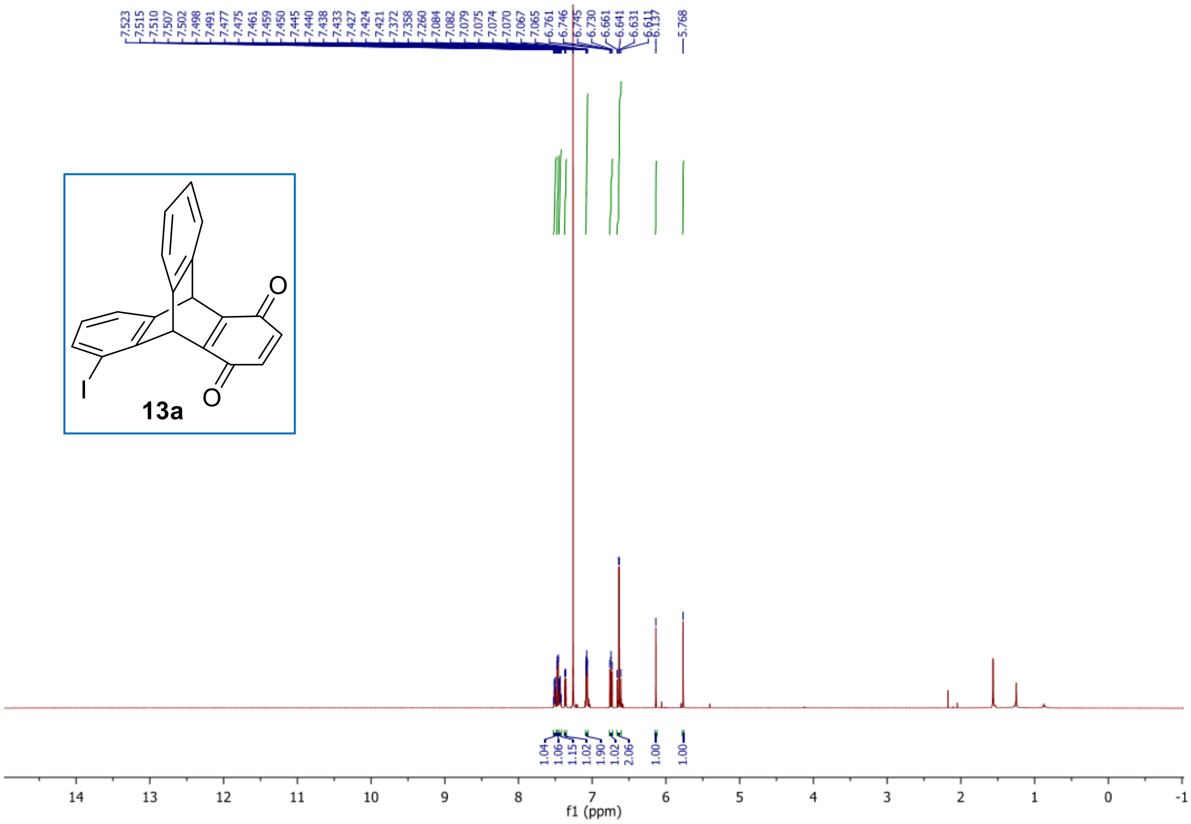

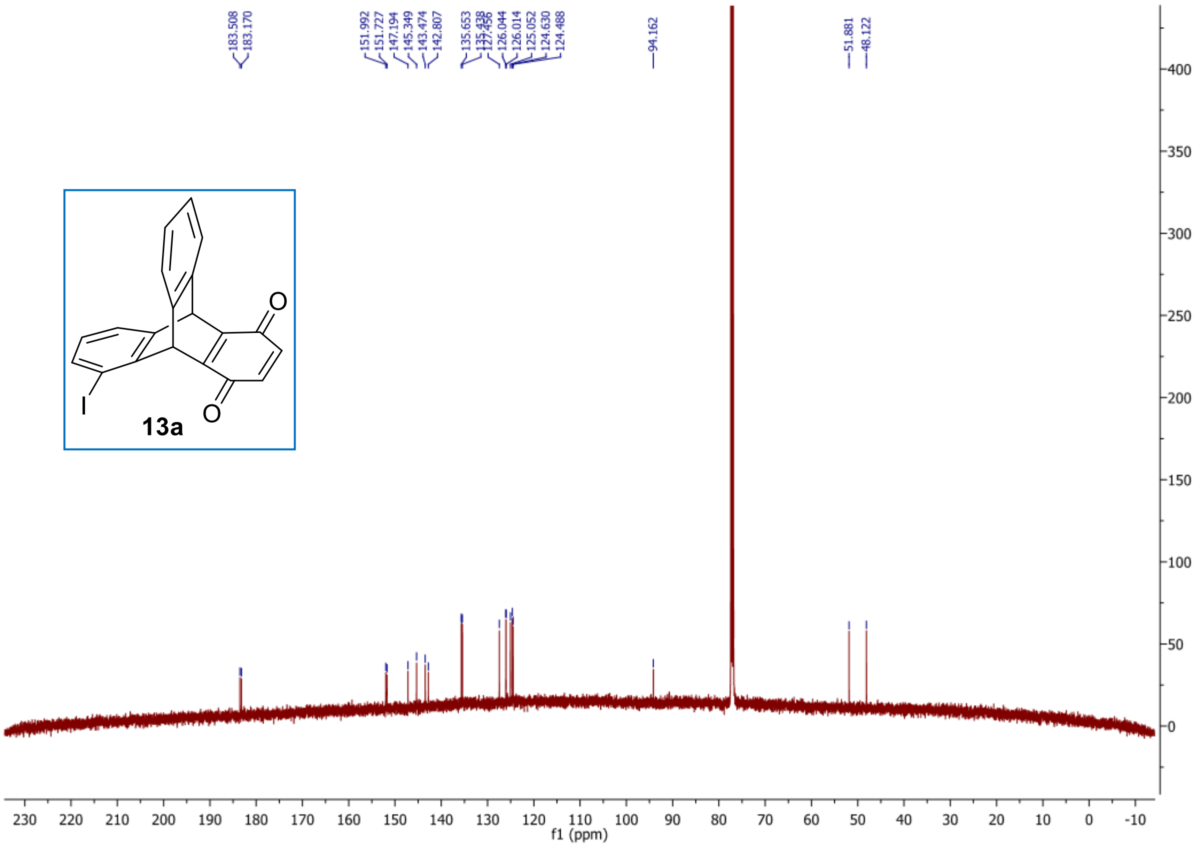

Mass spectra of 1-iodo-9,10-dihydro-9,10-[1,2]benzenoanthracene-13,16-dione (13a)

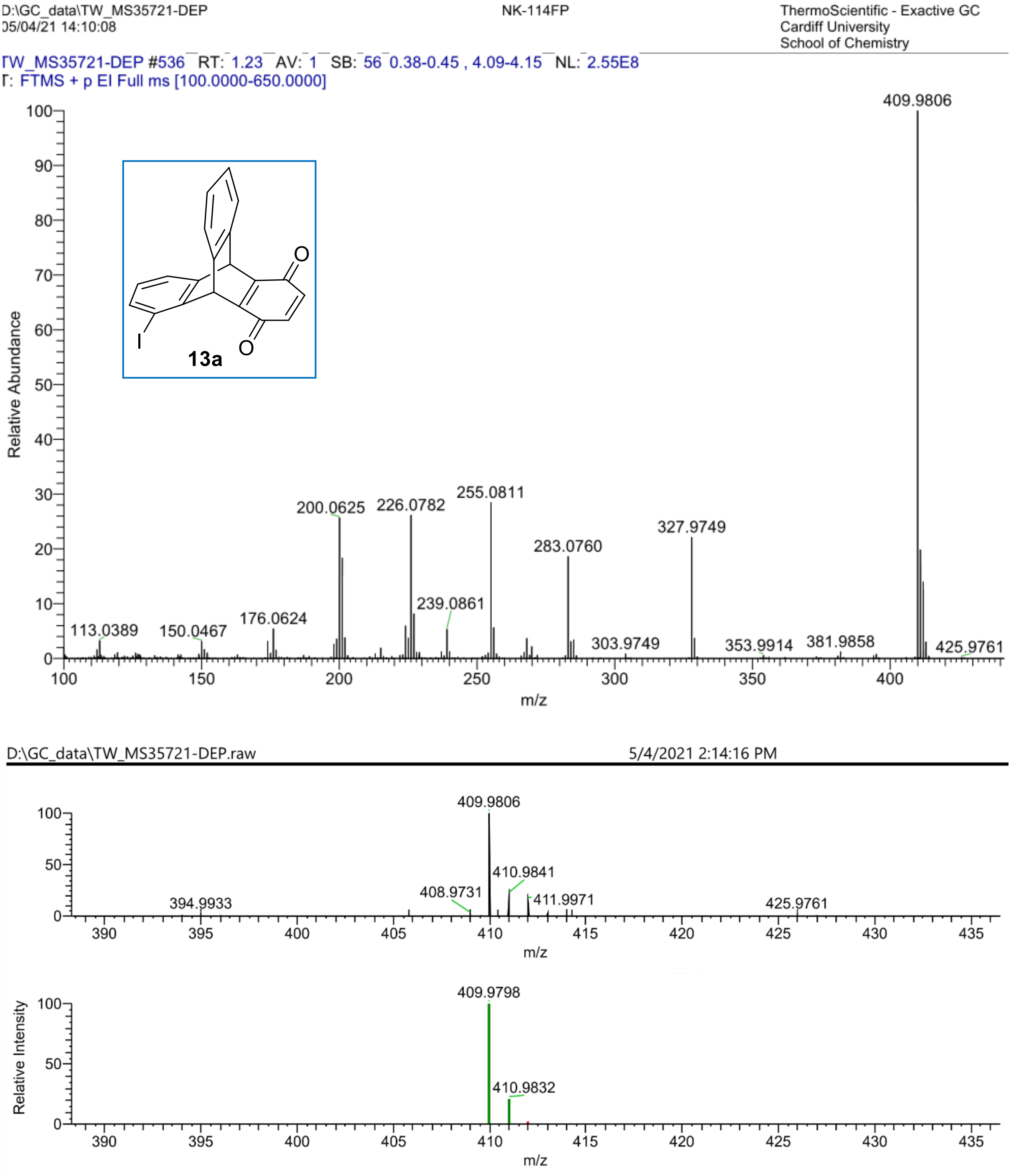

Peak Mass	Display Formula	Delta [ppm]	Theo. mass	Combined Score
409.9806	$C_{20}H_{11}O_2{}^{127}I$	1.80	409.97982	85.3499484459389

^{1}H and ^{13}C-NMR spectra of (1S,4R)-1-((((4-(((1S,4S)-7,7-dimethyl-2-oxobicyclo[2.2.1]heptan-1-yl)methoxy)-5-iodo-9,10-dihydro-9,10-[1,2]benzenoanthracen-1-yl)oxy)methyl)-7,7-dimethylbicyclo[2.2.1]heptan-2-one--sulfur(IV) oxide (1/2)(16a)

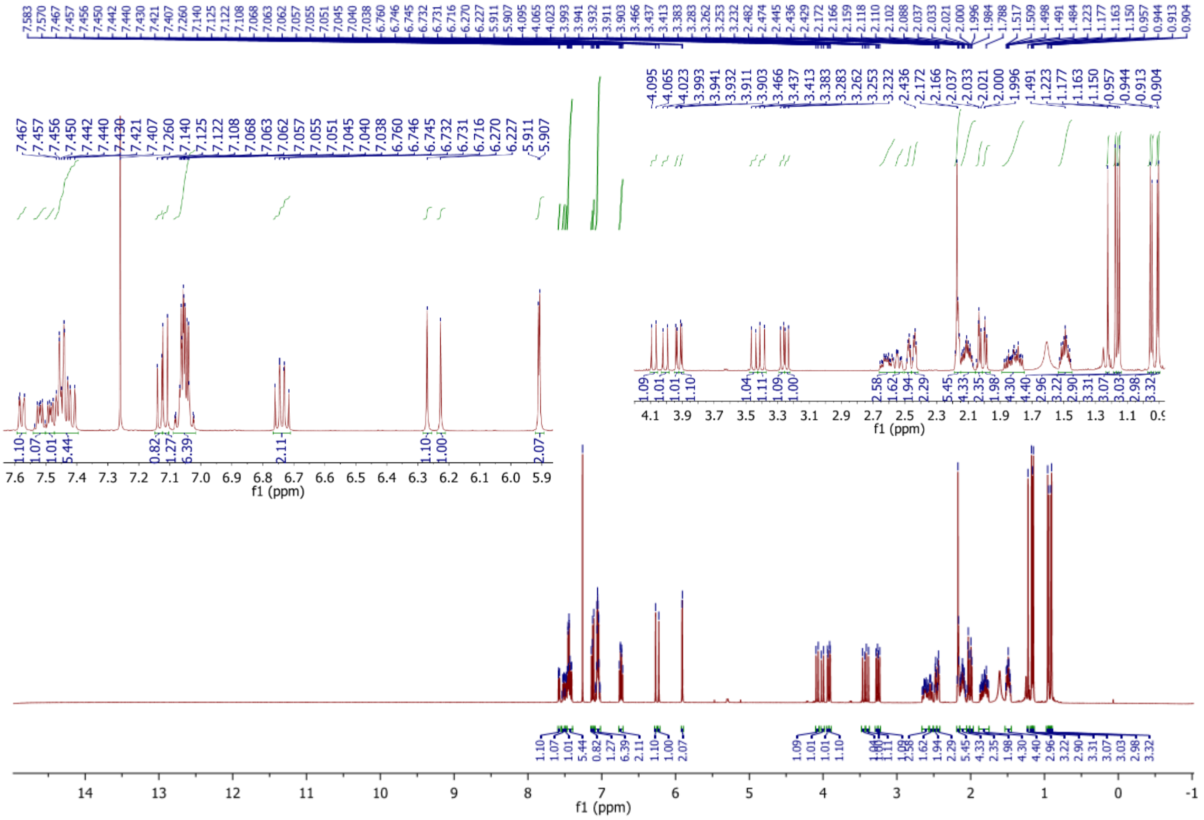

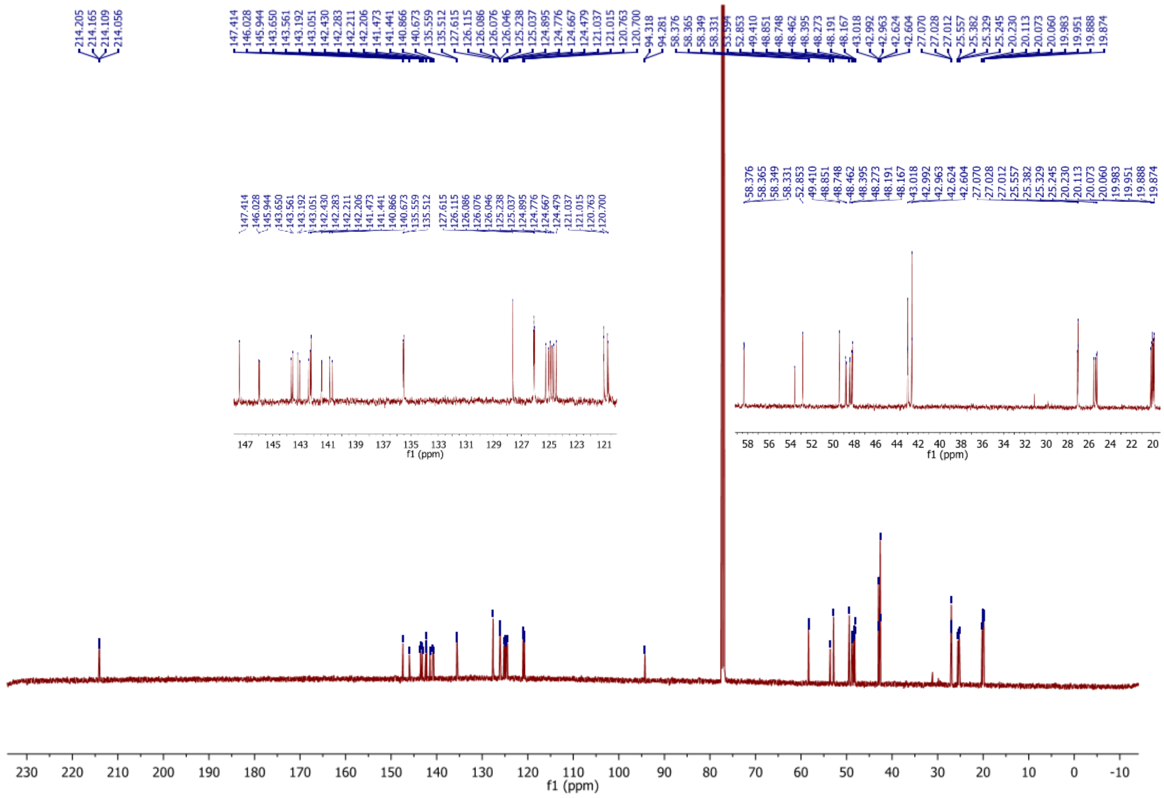

^{1}H and ^{13}C-NMR- spectra of 1-oxo-1-phenylpropan-2-yl 4-methylbenzenesulfonate (18)

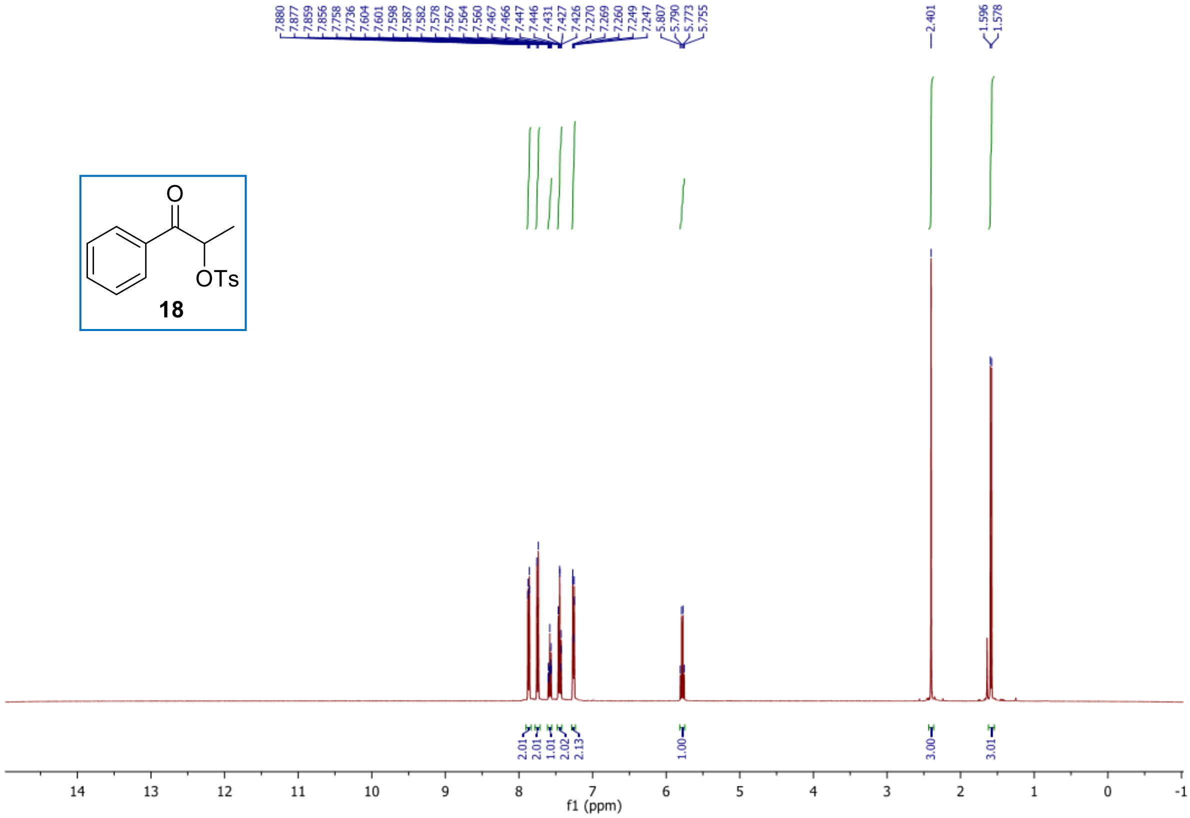

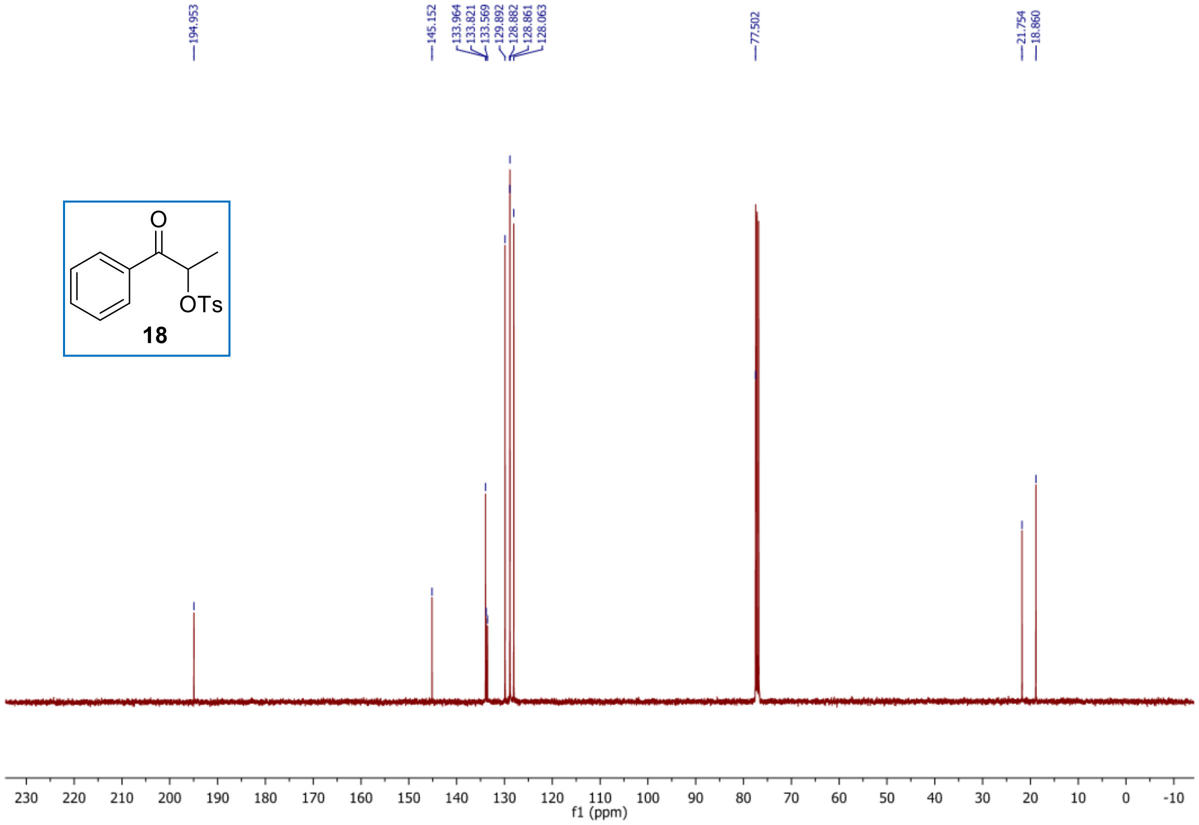

^{1}H and ^{13}C NMR spectra of N-((2-hydroxynaphthalen-1-yl)methyl)benzamide (19)

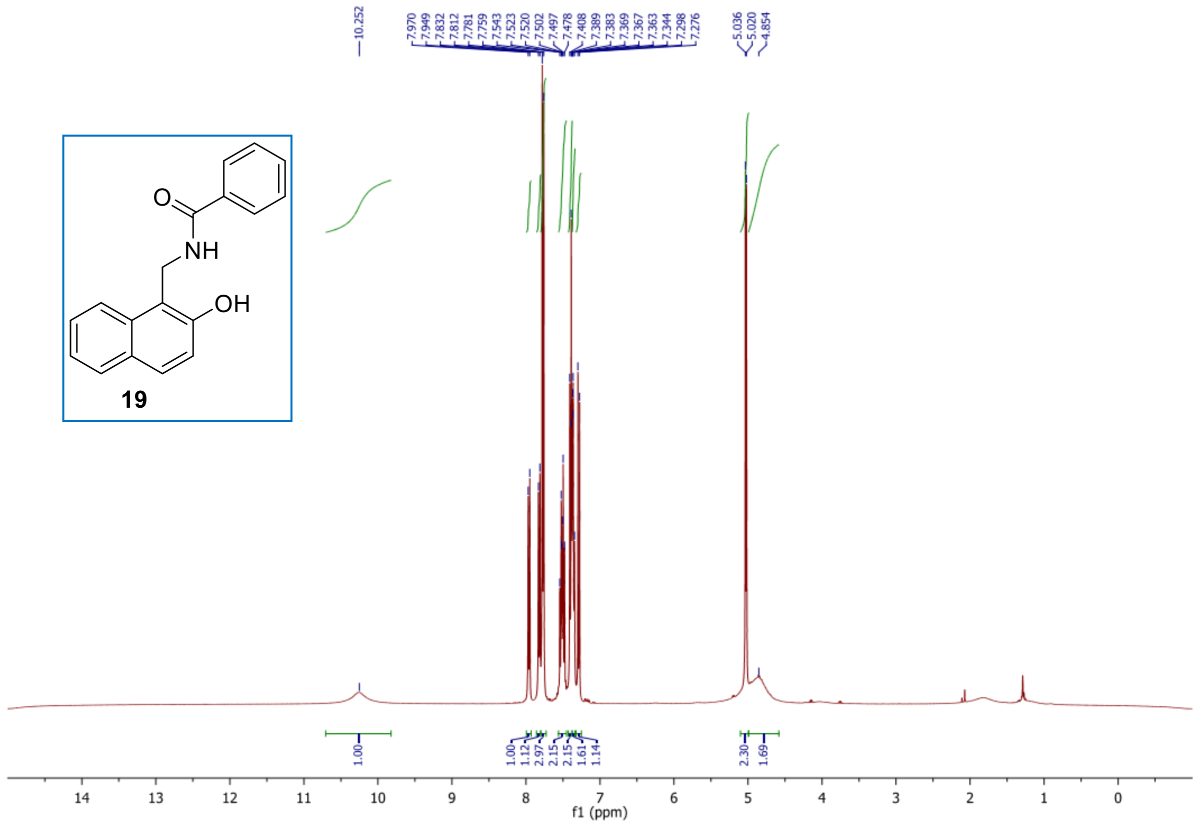

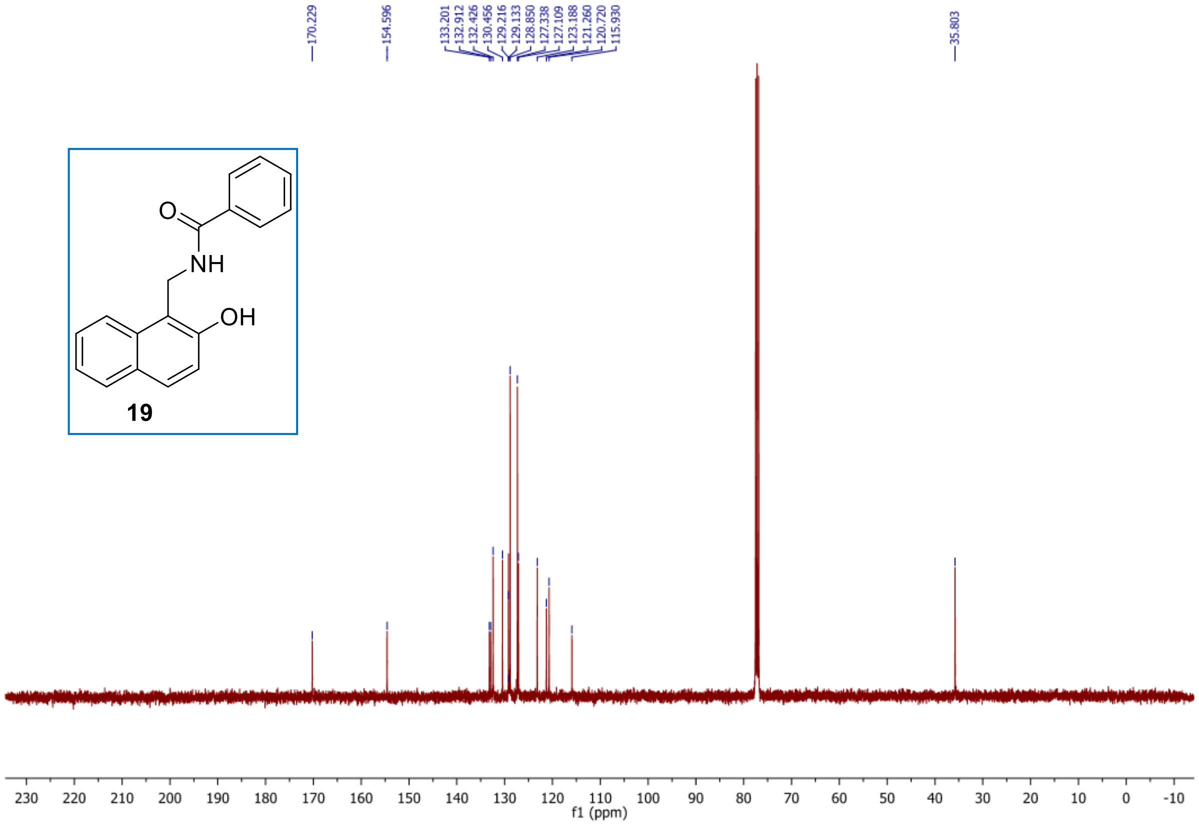

Chiral Iodotriptycenes: Synthesis and Catalytic Applications

^{1}H and ^{13}C NMR spectra of 2'-phenyl-2H,4'H-spiro[naphthalene-1,5'-oxazol]-2-one (20)

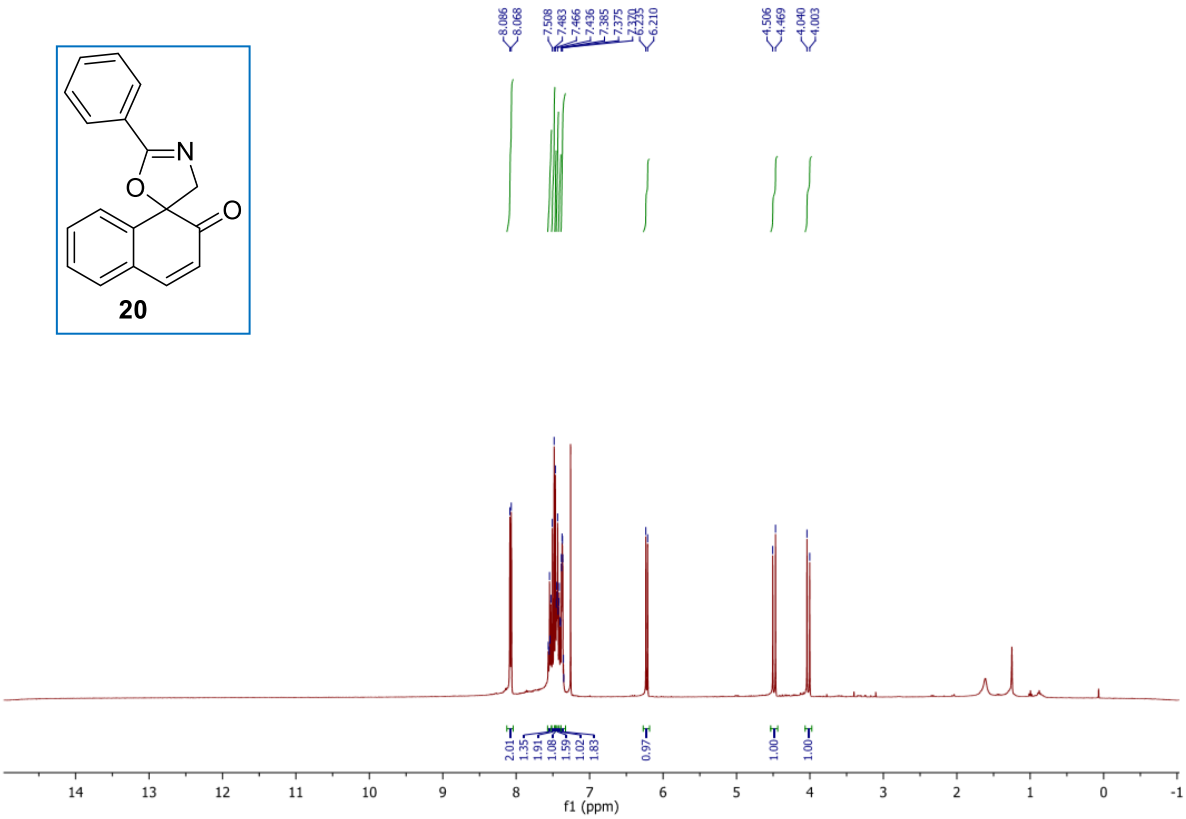

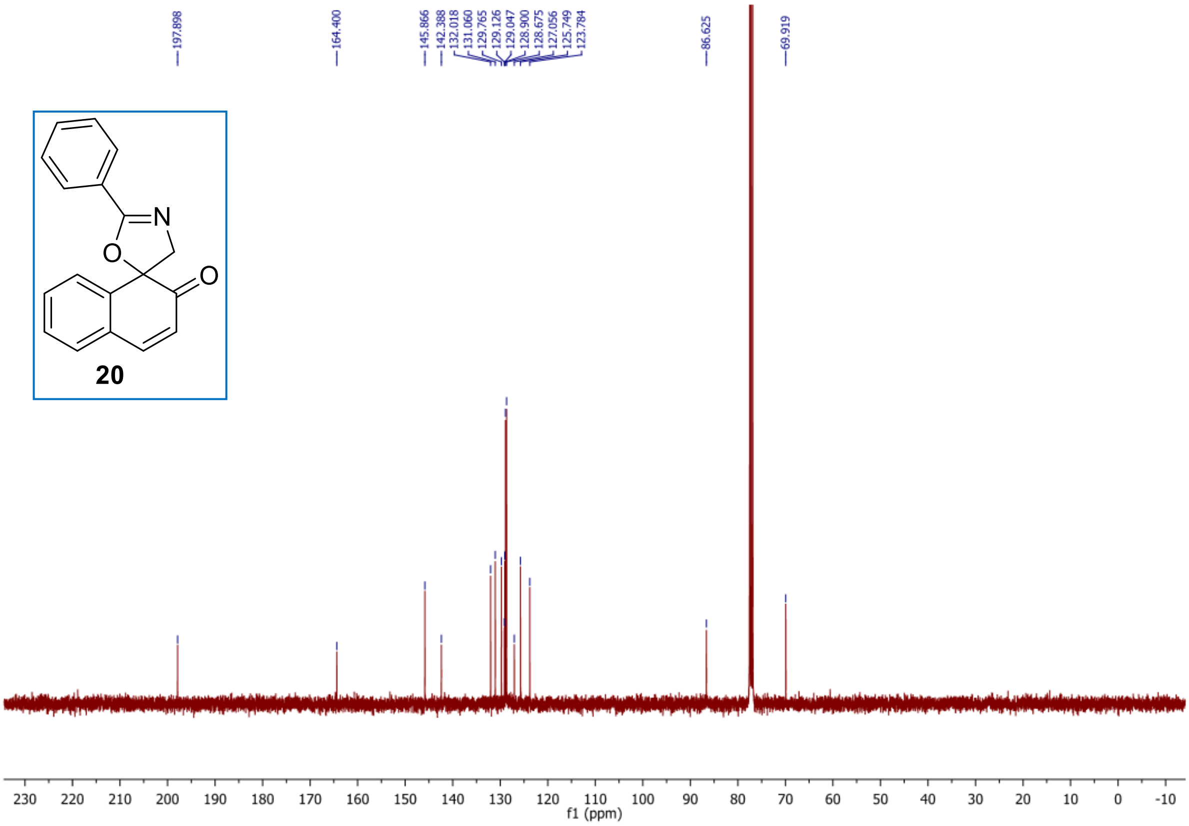

^{1}H and ^{13}C NMR spectra of 3,4-dihydro-2H-benzo[h]chromen-2-one (21a)

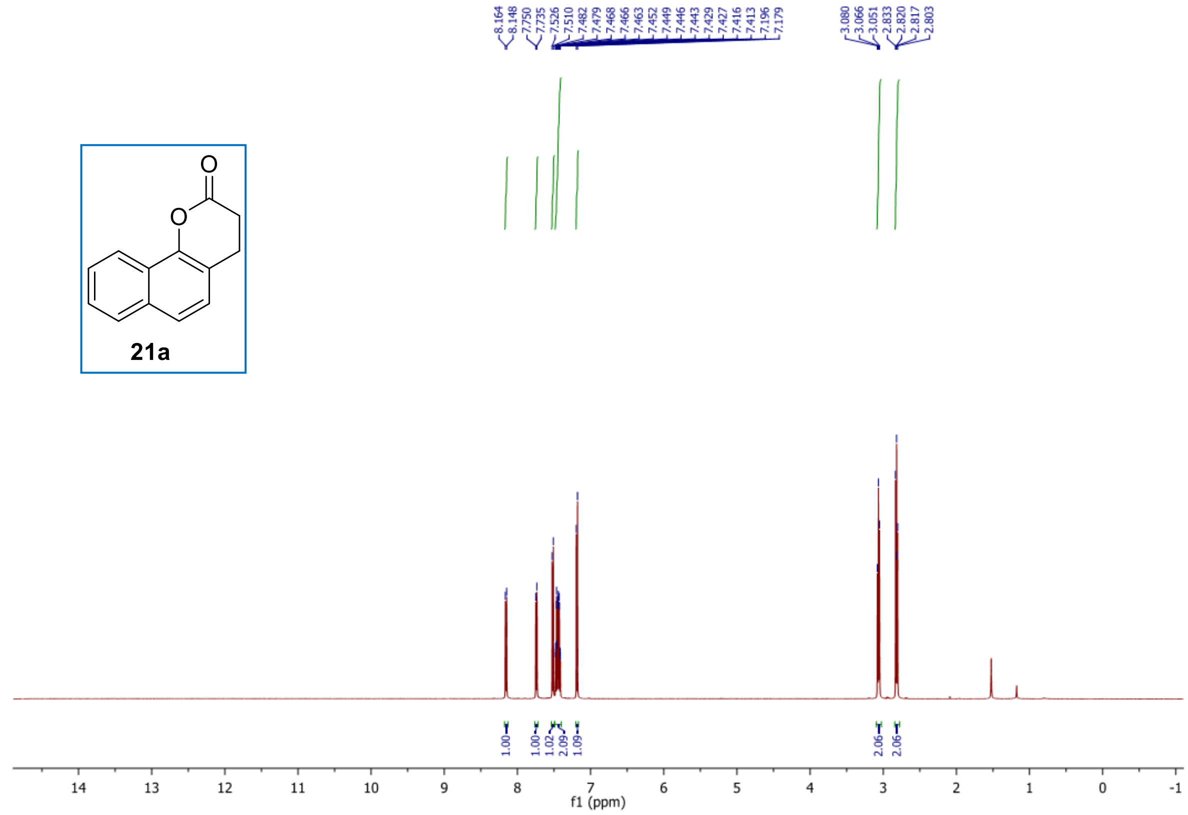

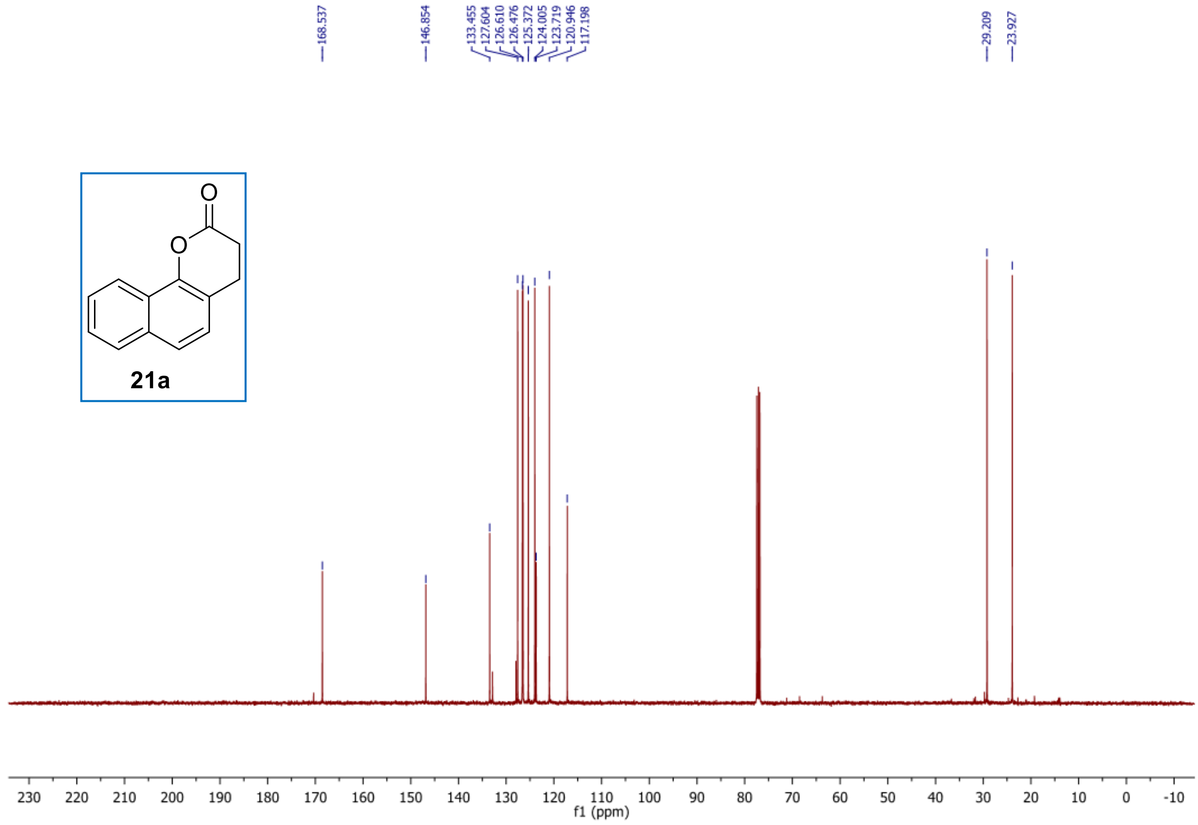

^{1}H and ^{13}C NMR spectra of 3-(1-hydroxynaphthalen-2-yl)propanoic acid (21)

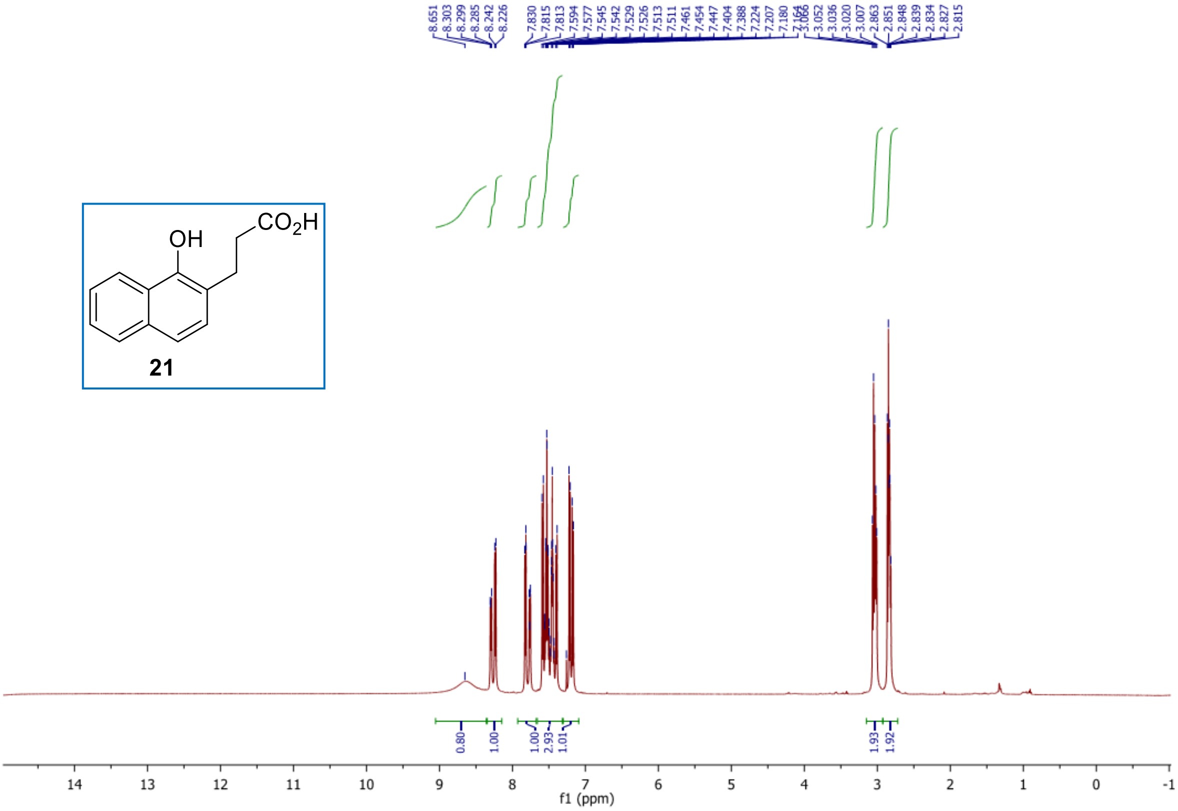

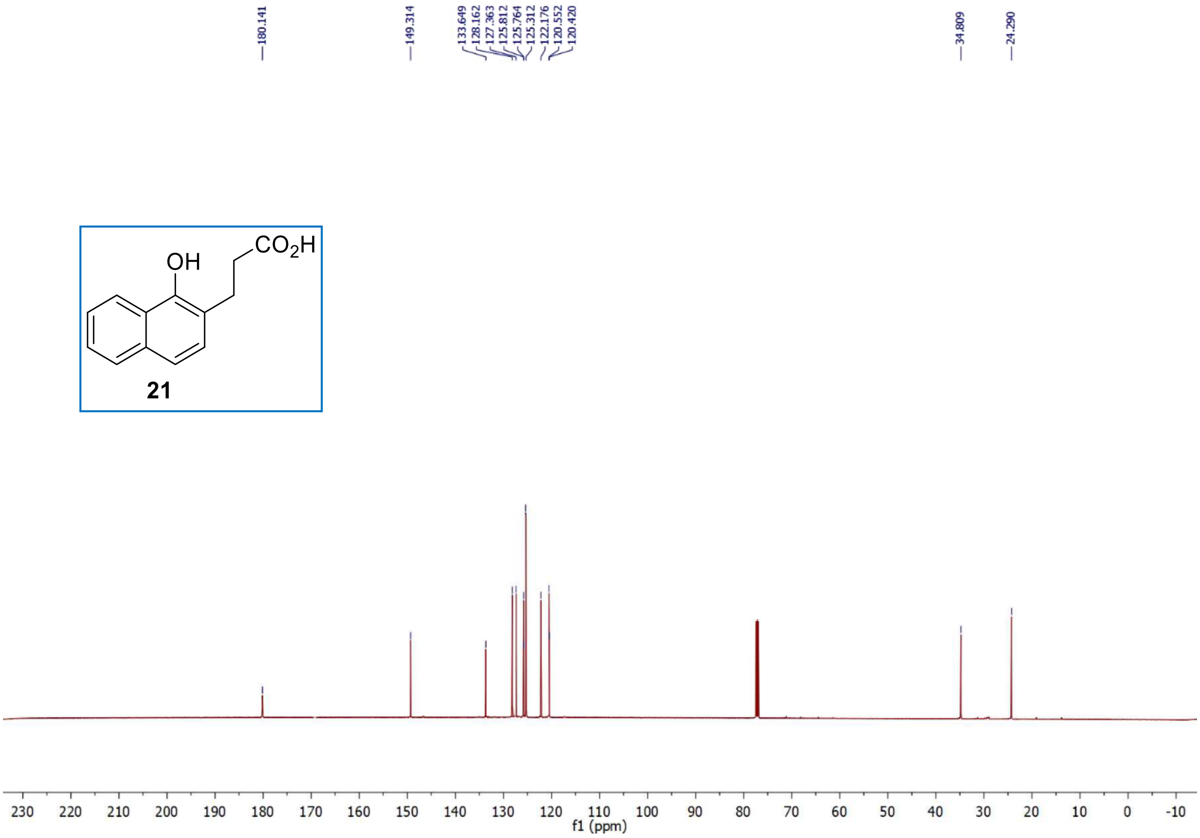

^{1}H and ^{13}C NMR spectra of 3,4-dihydro-1'H,5H-spiro[furan-2,2'-naphthalene]-1',5-dione (22)

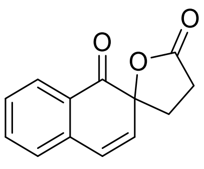

22

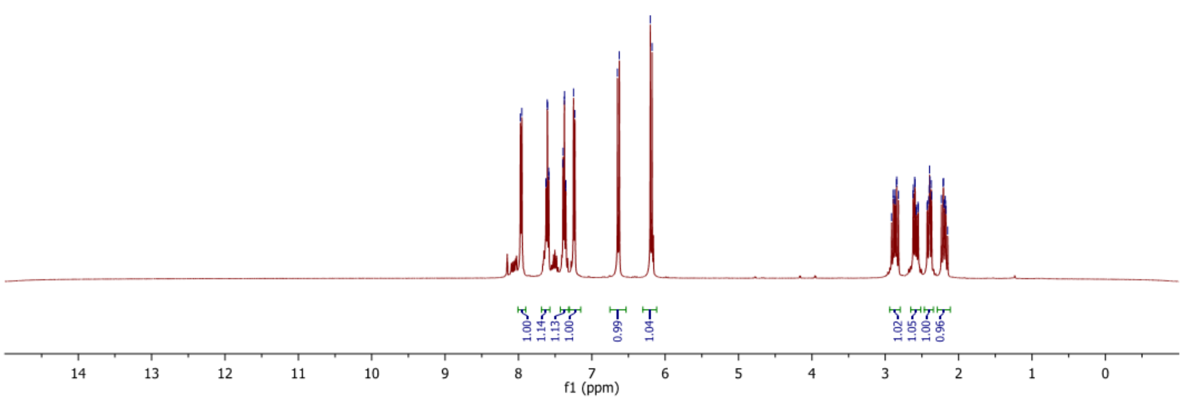

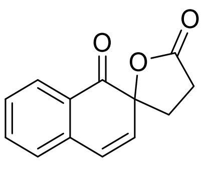

22

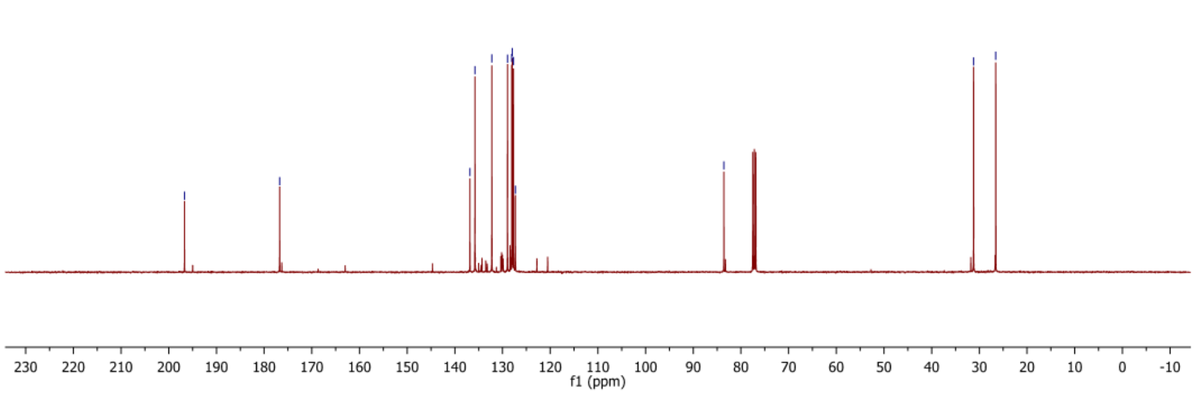

^{1}H and ^{13}C NMR spectra of pent-1-ene-1,1-diyldibenzene (23)

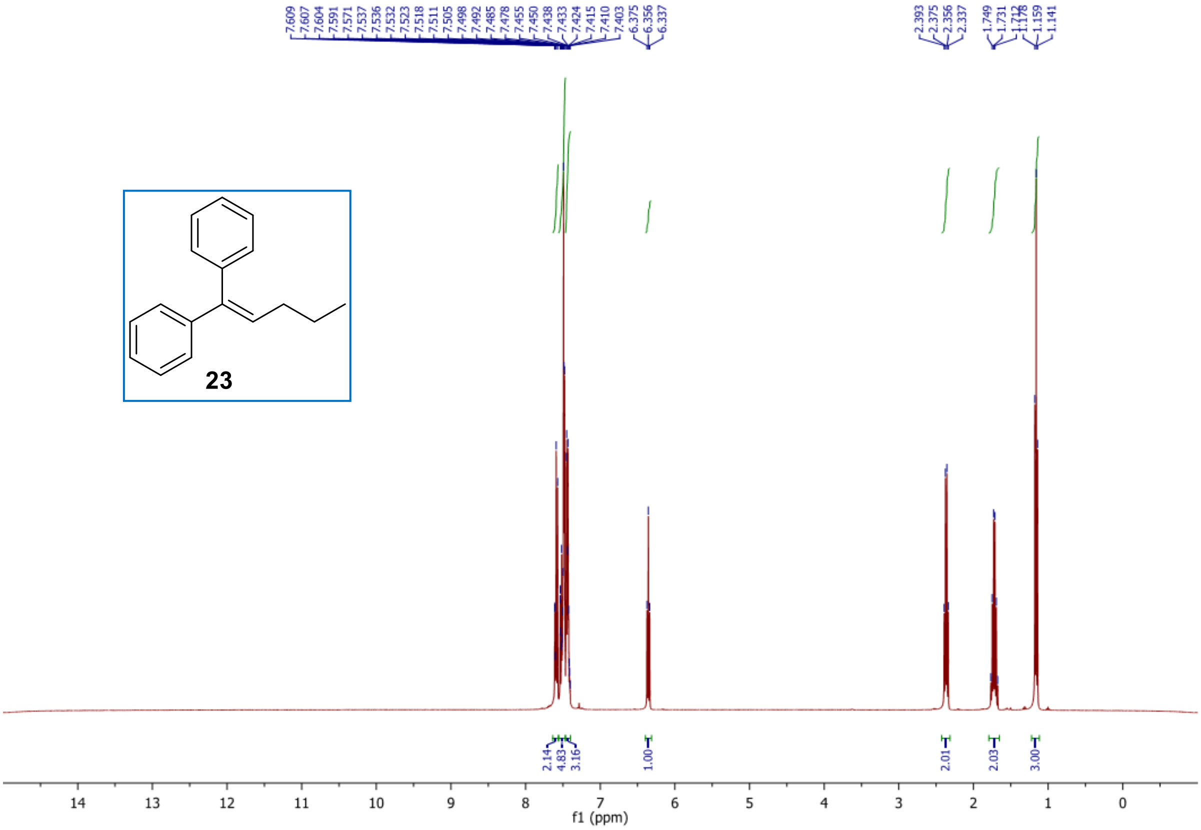

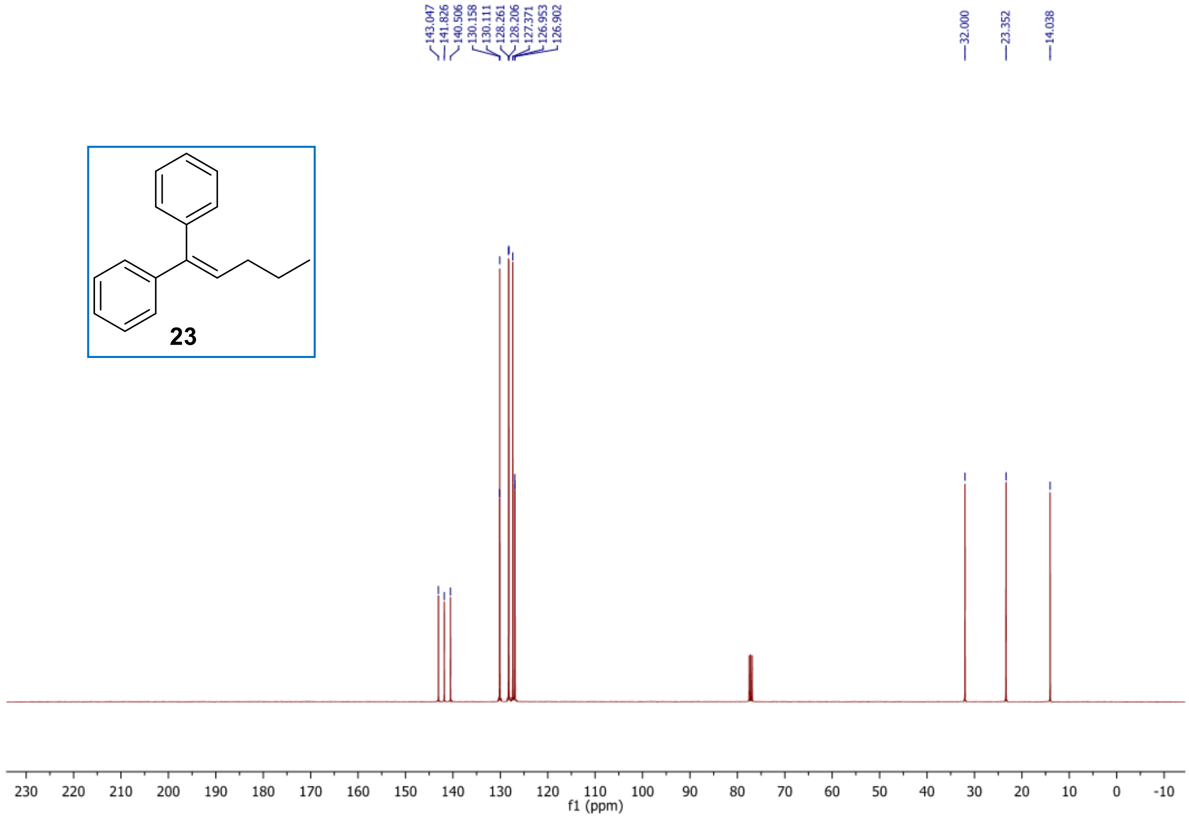

^{1}H and ^{13}C NMR spectra of 1,2-Diphenylpentan-1-one (24)

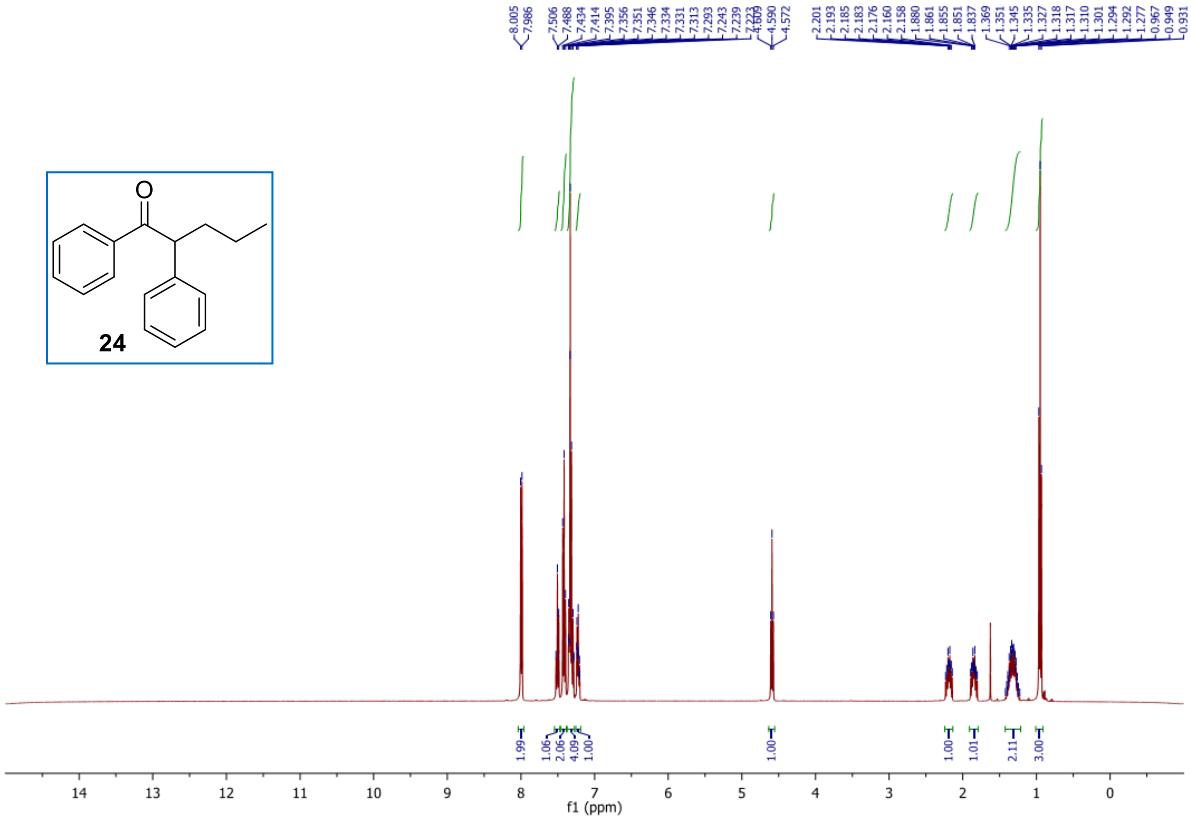

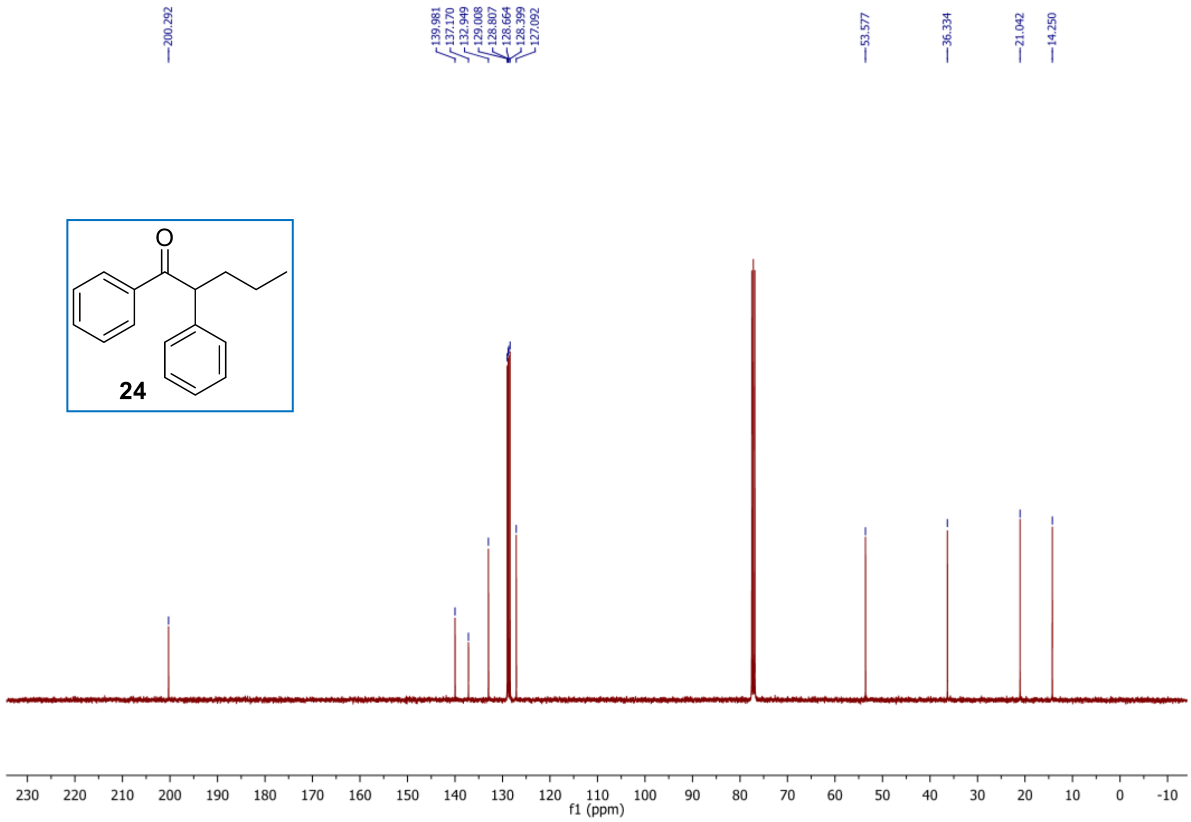

6. HPLC Chromatogram

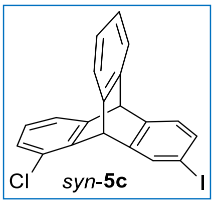

Racemate

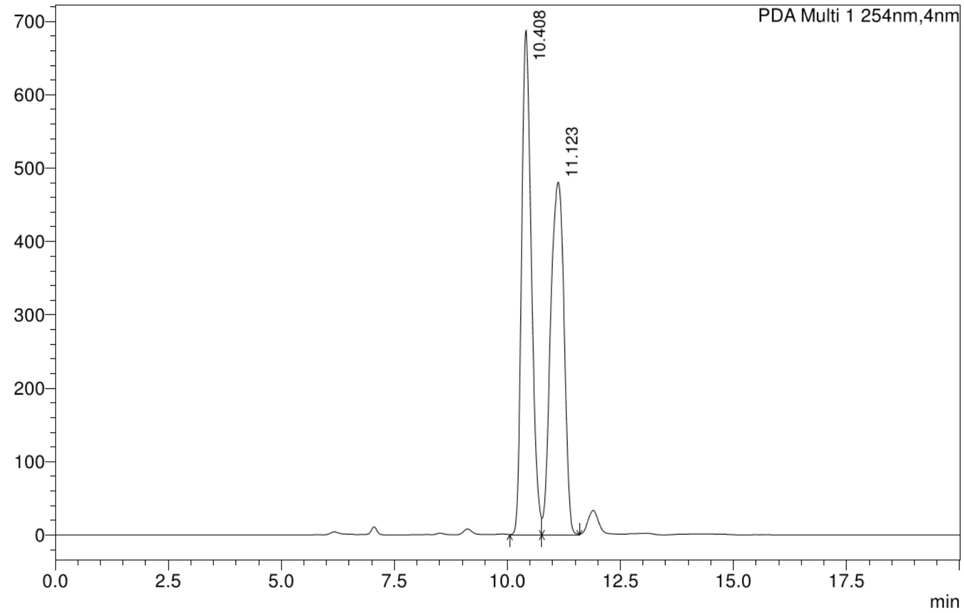

PDA Ch 1 254 nm

Peak#	Ret. Time	Area%
1	10.408	50.965
2	11.123	49.035
Total		100.00

HPLC-analysis: YMC - I.D. S-5 µm, LC Column (250 X 4.6 mm), *n*-hexane/*i*-PrOH = 98:2, flow rate = 0.5 mL/min, λ = 254 nm, t_{R1} = 10.4 min, t_{R2} = 11.1 min.

(–)-*syn*-**5c**

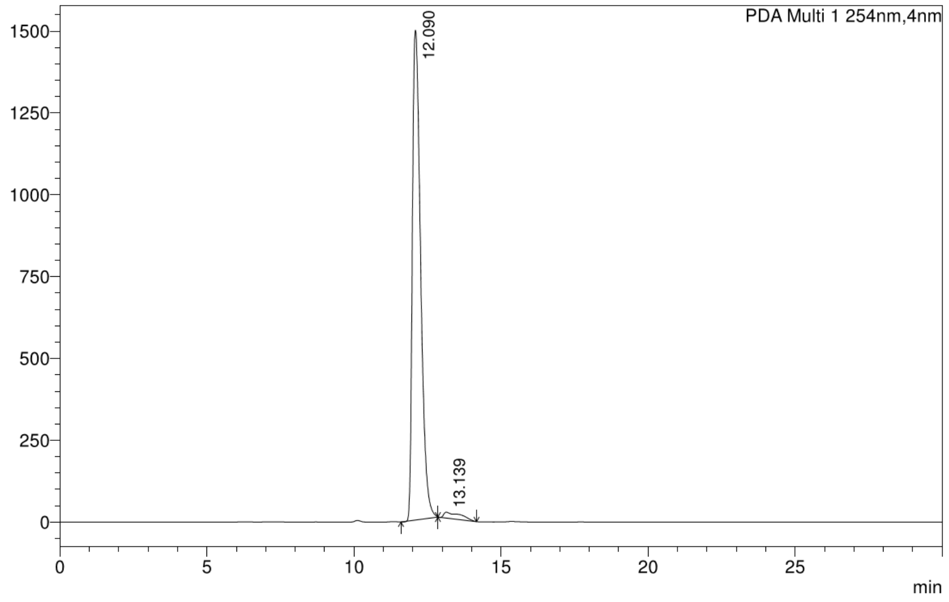

PDA Ch 1 254 nm

Peak#	Ret. Time	Area%
1	12.090	98.850
2	13.139	1.150
Total		100.00

HPLC-analysis: YMC - I.D. S-5 μm, LC Column (250 X 4.6 mm), *n*-hexane/*i*-PrOH = 98:2, flow rate = 0.5 mL/min, λ = 254 nm, t_{R1} (major) = 12.0 min, t_{R2} (minor) = 13.1 min. *ee* = 98%, OD = - 22.85° (c = 0.350, CH_3CN).

(+)-*syn*-**5c**

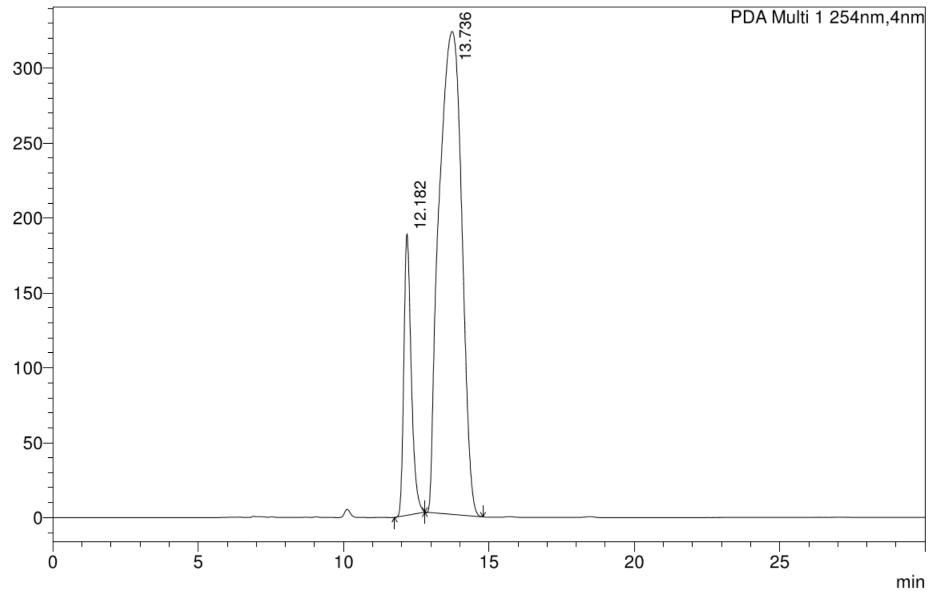

PDA Ch 1 254 nm

Peak#	Ret. Time	Area%
1	12.182	16.376
2	13.736	83.624
Total		100.00

HPLC-analysis: YMC - I.D. S-5 µm, LC Column (250 X 4.6 mm), *n*-hexane/*i*-PrOH = 98:2, flow rate = 0.5 mL/min, λ = 254 nm, t_{R1} (minor) = 12.1 min, t_{R2} (major) = 13.7 min. *ee* = 67%, OD = + 17.39° (c = 0.288, CH_3CN)

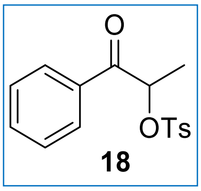

Racemate

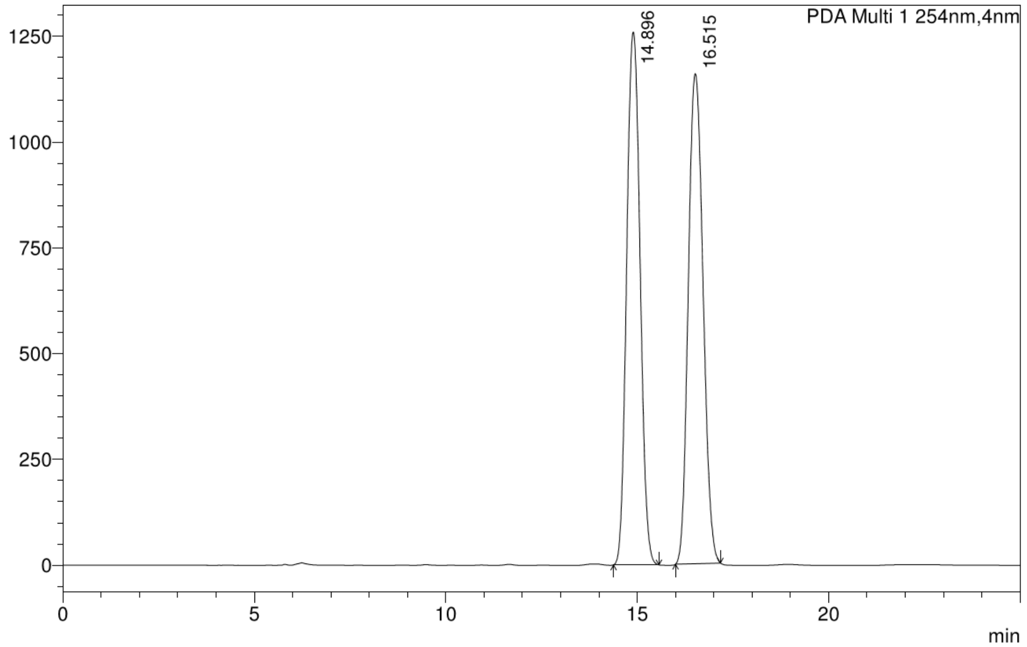

PDA Ch 1 254 nm

Peak#	Ret. Time	Area%
1	14.896	49.519
2	16.515	50.481
Total		100.00

HPLC-analysis: YMC - I.D. S-5 μm, LC Column (250 X 4.6 mm), *n*-hexane/*i*-PrOH = 85:15, flow rate = 0.7 mL/min, λ = 254 nm, t_{R1} = 14.8 min, t_{R2} = 16.5 min.

Catalyzed by (–)-*syn*-**5c**

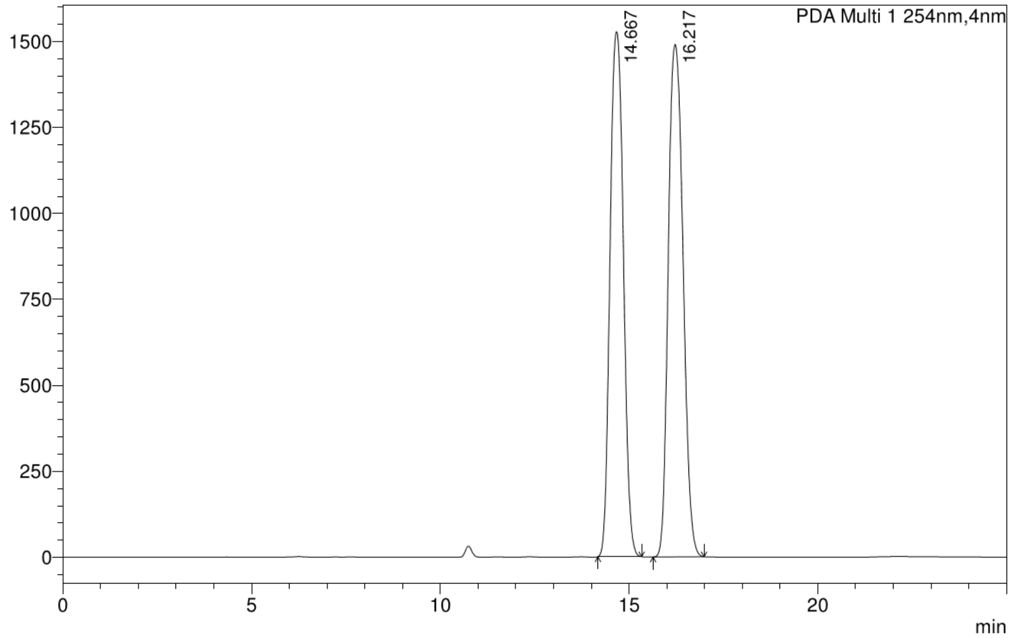

PDA Ch 1 254 nm

Peak#	Ret. Time	Area%
1	14.667	48.044
2	16.217	51.956
Total		100.00

HPLC-analysis: YMC - I.D. S-5 μm, LC Column (250 X 4.6 mm), *n*-hexane/*i*-PrOH = 85:15, flow rate = 0.7 mL/min, λ = 254 nm, t_{R1} (minor) = 14.6 min, t_{R2} (major) = 16.2 min. *ee* = 4%.

Catalyzed by (+)-*syn*-**5c**

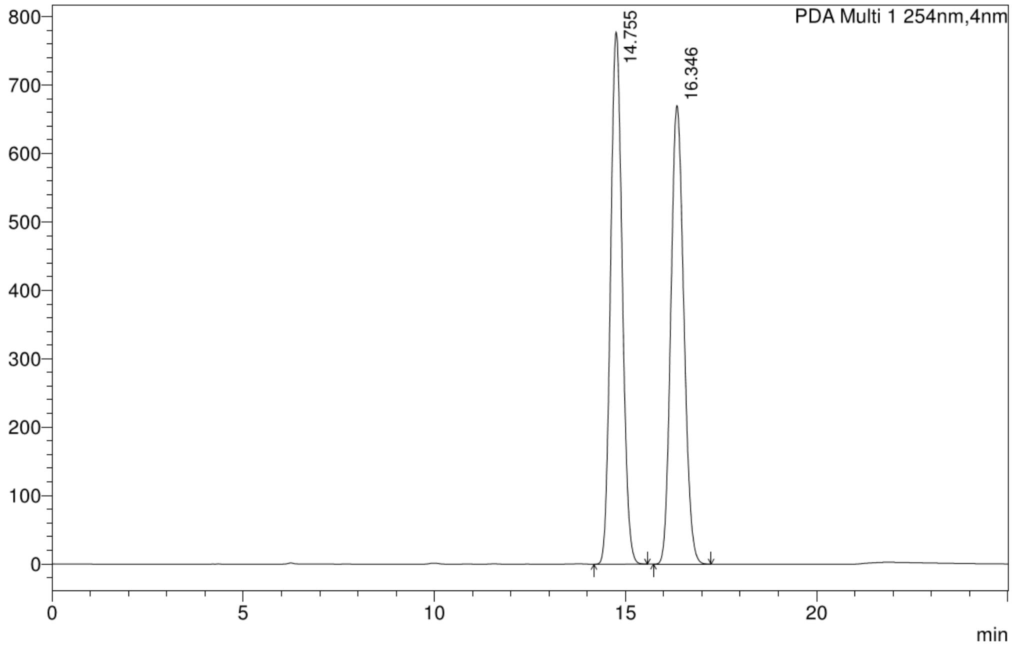

PDA Ch 1 254 nm

Peak#	Ret. Time	Area%
1	14.755	50.964
2	16.346	49.036
Total		100.00

HPLC-analysis: YMC - I.D. S-5 µm, LC Column (250 X 4.6 mm), *n*-hexane/*i*-PrOH = 85:15, flow rate = 0.7 mL/min, λ = 254 nm, t_{R1} (major) = 14.7 min, t_{R2} (minor) = 16.3 min. *ee* = 2%.

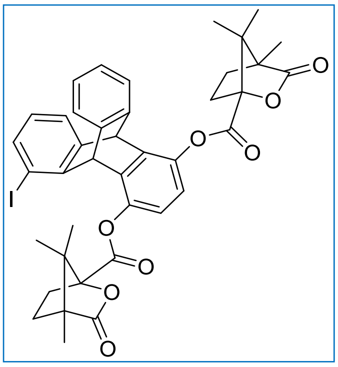

Racemate **16**

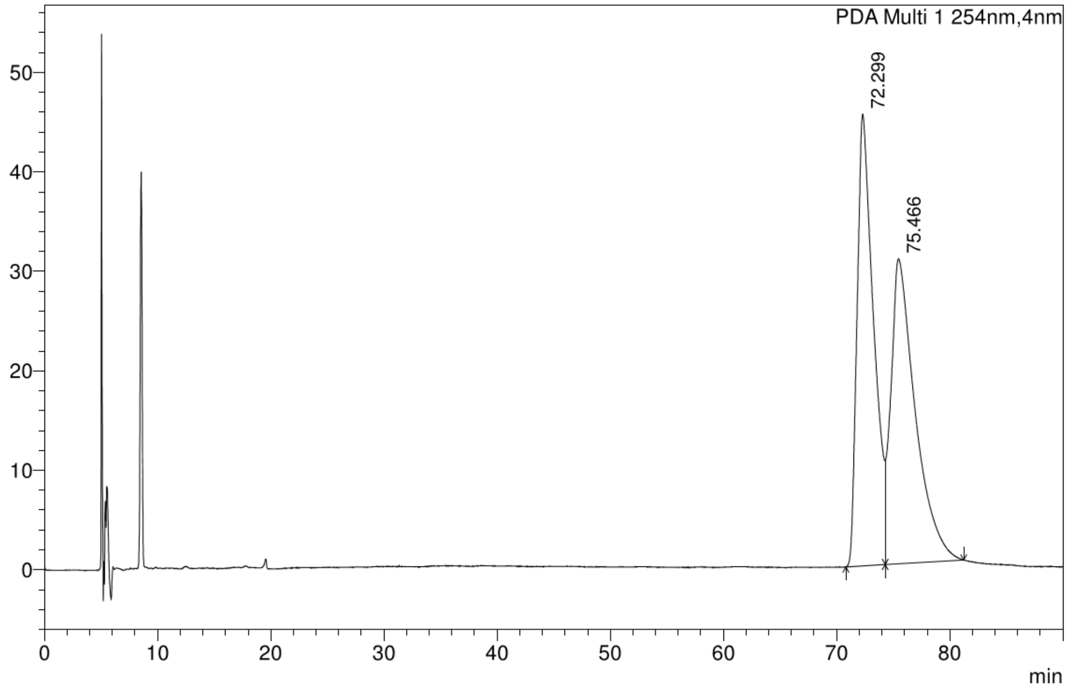

PDA Ch 1 254 nm

Peak#	Ret. Time	Area%
1	72.299	49.906
2	75.466	50.094
Total		100.00

HPLC-analysis: Polaris – Si, I.D. S-5 µm, LC Column (250 X 4.6 mm), *n*-hexane/THF = 90 : 10, flow rate = 0.7mL/min, λ = 254 nm, t_{R1} = 72.2 min, t_{R2} = 75.4 min.

(+)-**16**

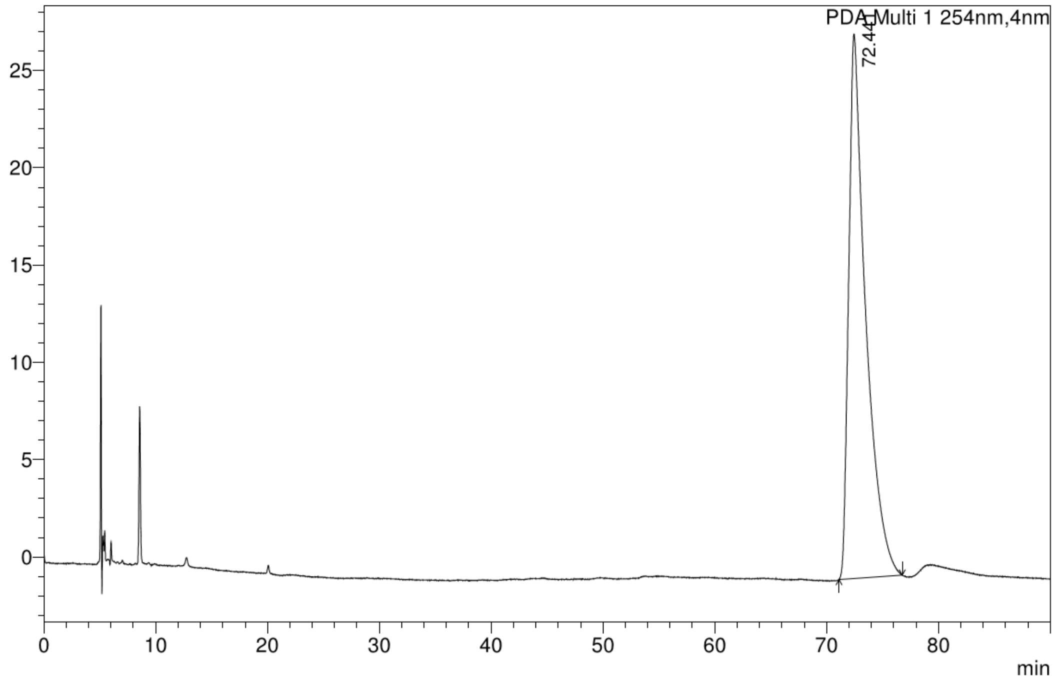

PDA Ch 1 254 nm

Peak#	Ret. Time	Area%
1	72.441	100.000
Total		100.00

HPLC-analysis: Polaris – Si, I.D. S-5 µm, LC Column (250 X 4.6 mm), *n*-hexane/THF = 90 : 10, flow rate = 0.7 mL/min, λ = 254 nm, t_R = 72.4 min.

(–)-**16**

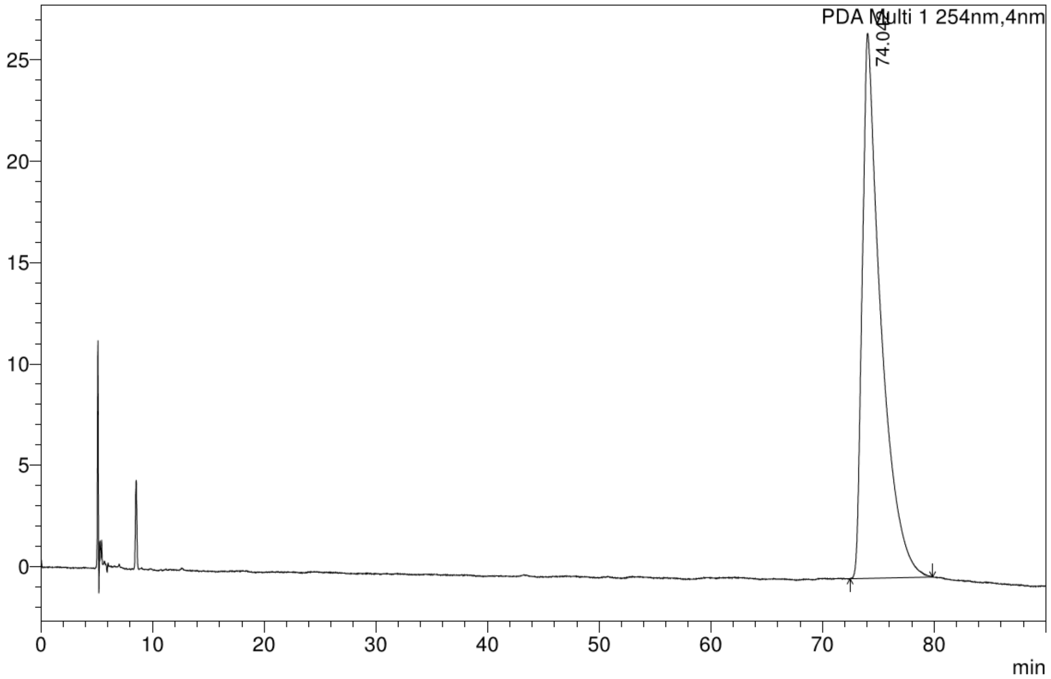

PDA Ch 1 254 nm

Peak#	Ret. Time	Area%
1	74.042	100.000
Total		100.00

HPLC-analysis: Polaris – Si, I.D. S-5 µm, LC Column (250 X 4.6 mm), *n*-hexane/THF = 90 : 10, flow rate = 0.7 mL/min, λ = 254 nm, t_R = 74.0 min.

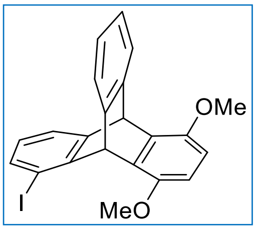

Racemate 5f

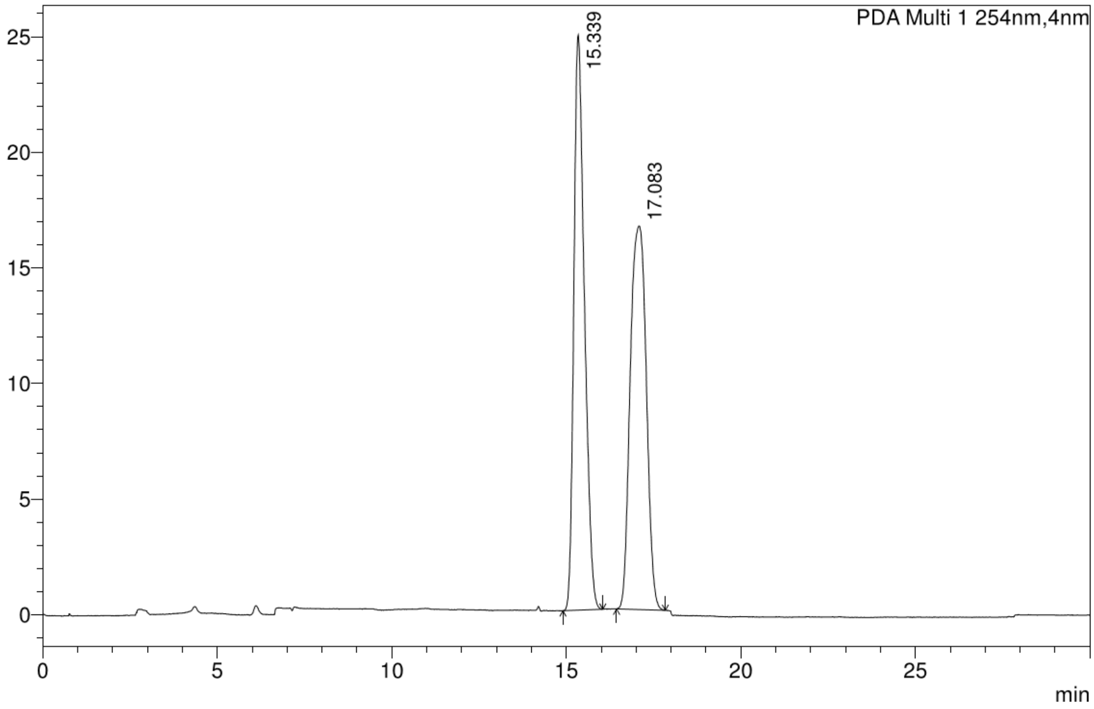

PDA Ch 1 254 nm

Peak#	Ret. Time	Area%
1	15.339	49.789
2	17.083	50.211
Total		100.00

HPLC-analysis: YMC Chiral Amylose-C - I.D. S-5 µm, LC Column (250 X 4.6 mm), *n*-hexane /*i*-PrOH = 98 : 2, flow rate = 0.5 mL/min, λ = 254 nm, t_{R1} = 15.3 min, t_{R2} = 17.0 min.

(+)-(9*R*,10*R*)–**5f**

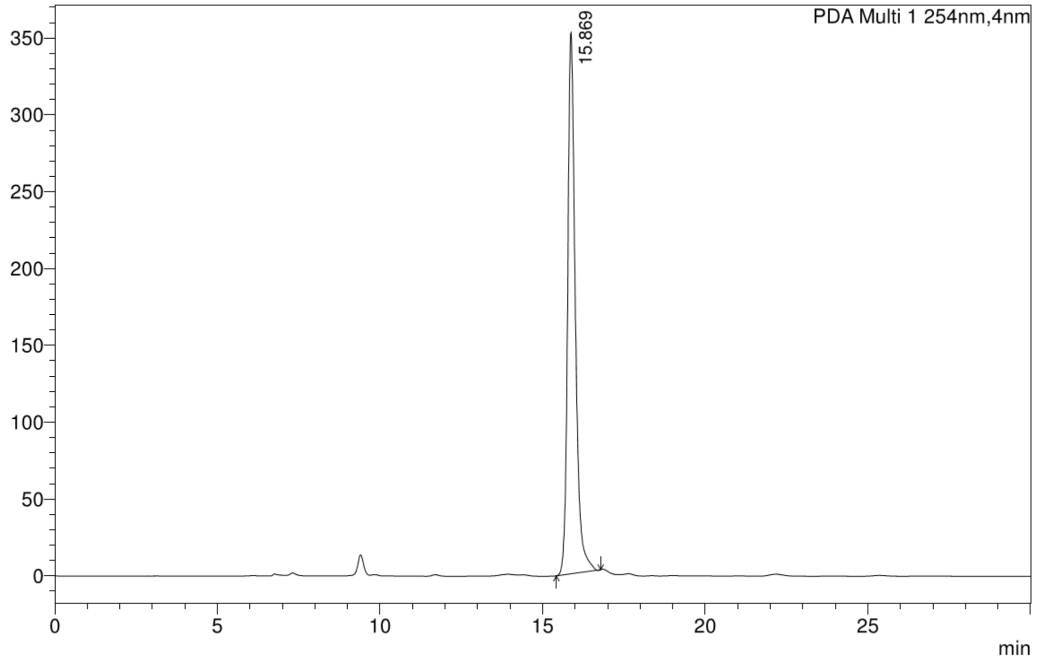

PDA Ch 1 254 nm

Peak#	Ret. Time	Area%
1	15.869	100.00
Total		100.00

HPLC-analysis: YMC Chiral Amylose-C - I.D. S-5 µm, LC Column (250 X 4.6 mm), *n*-hexane/*i*-PrOH = 98 : 2, flow rate = 0.5 mL/min, λ = 254 nm, t_R = 15.8 min; (+)–(9*R*,10*R*)–**5f;** *ee* = 100%; $[\alpha]_D^{20}$ = + 28.88 (c, 0.18 in $CHCl_3$).

(–)-(9*S*,10*S*)–**5f**

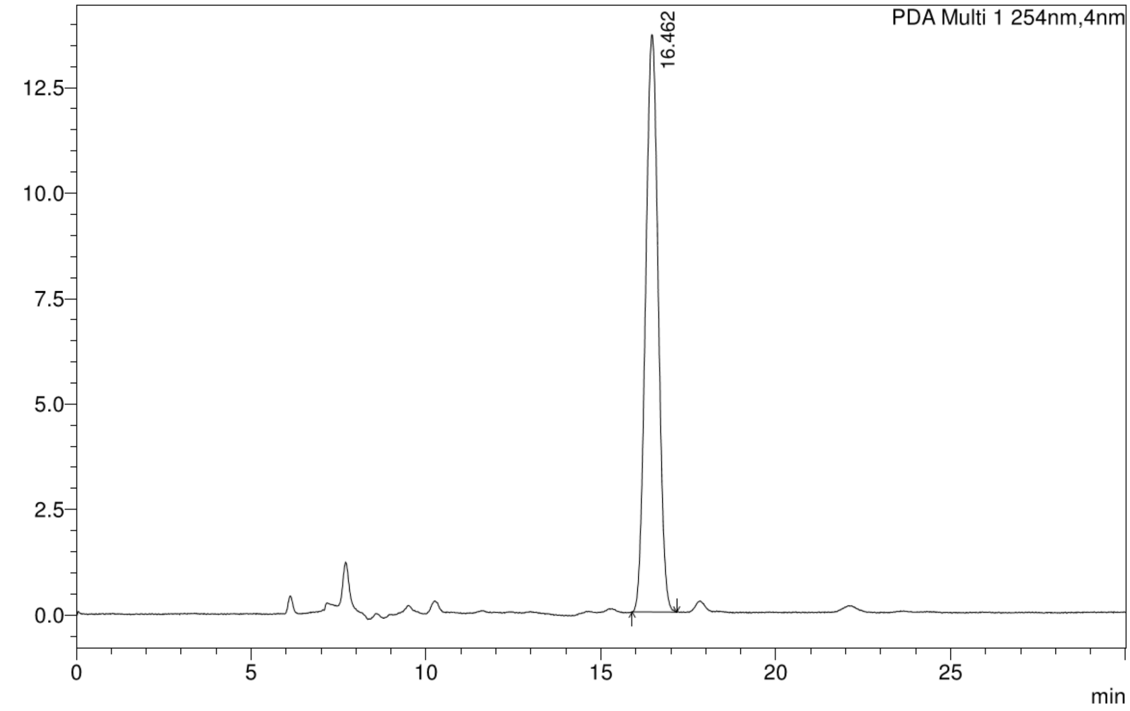

PDA Ch 1 254 nm

Peak#	Ret. Time	Area%
1	16.462	100.00
Total		100.00

HPLC-analysis: YMC Chiral Amylose-C - I.D. S-5 µm, LC Column (250 X 4.6 mm), *n*-hexane /*i*-PrOH = 98 : 2, flow rate = 0.5 mL/min, λ = 254 nm, t_R = 16.4 min; (–)– (9*S*,10*S*)–**5f**; *ee* = 100%; $[\alpha]_D^{20}$ = − 18.57 (c, 0.28 in $CHCl_3$).

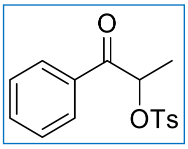

Racemate 18

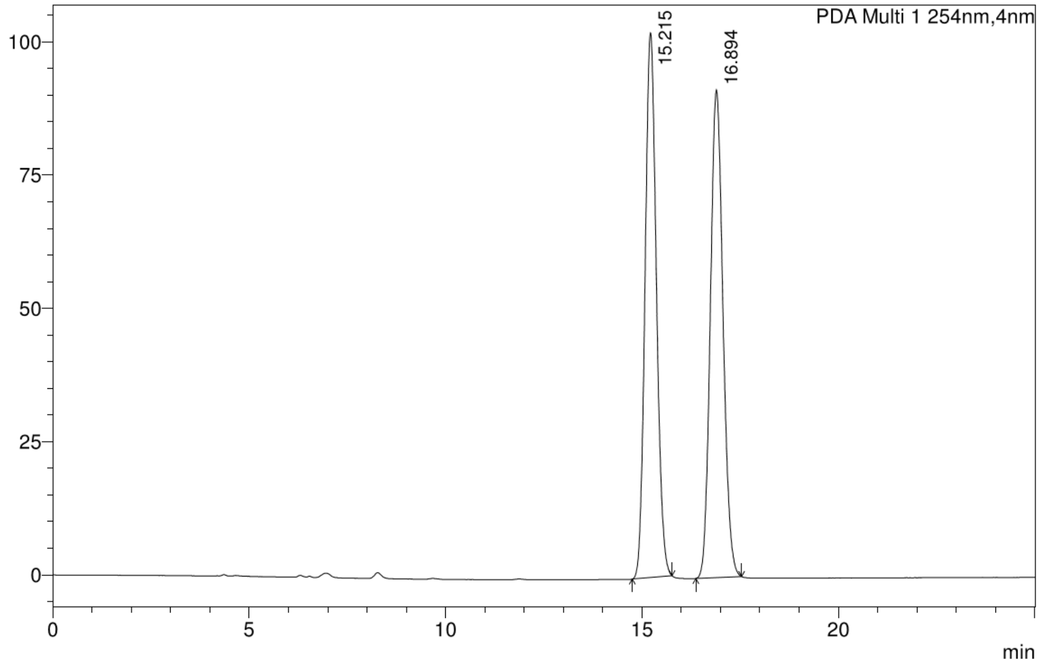

PDA Ch 1 254 nm

Peak#	Ret. Time	Area%
1	15.215	49.922
2	16.894	50.078
Total		100.00

HPLC-analysis: YMC Chiral Amylose-C - I.D. S-5 µm, LC Column (250 X 4.6 mm), *n*-hexane/*i*-PrOH = 85:15, flow rate = 0.7 mL/min, λ = 254 nm, t_{R1} = 15.2 min, t_{R2} = 16.8 min.

Catalyzed by (–)-**5f**

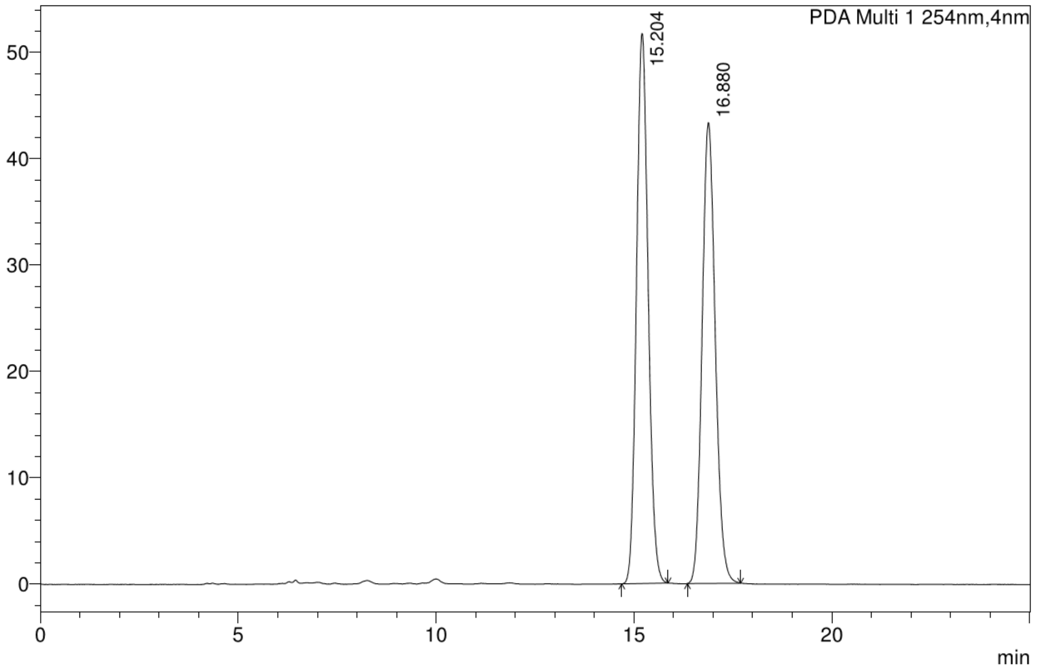

PDA Ch 1 254 nm

Peak#	Ret. Time	Area%
1	15.204	51.405
2	16.880	48.595
Total		100.00

HPLC-analysis: YMC Chiral Amylose-C - I.D. S-5 µm, LC Column (250 X 4.6 mm), *n*-hexane/*i*-PrOH = 85:15, flow rate = 0.7 mL/min, λ = 254 nm, t_{R1} (major) = 15.2 min, t_{R2} (minor) = 16.8 min. *ee* = 3%.

Catalyzed by (+)-**5f**

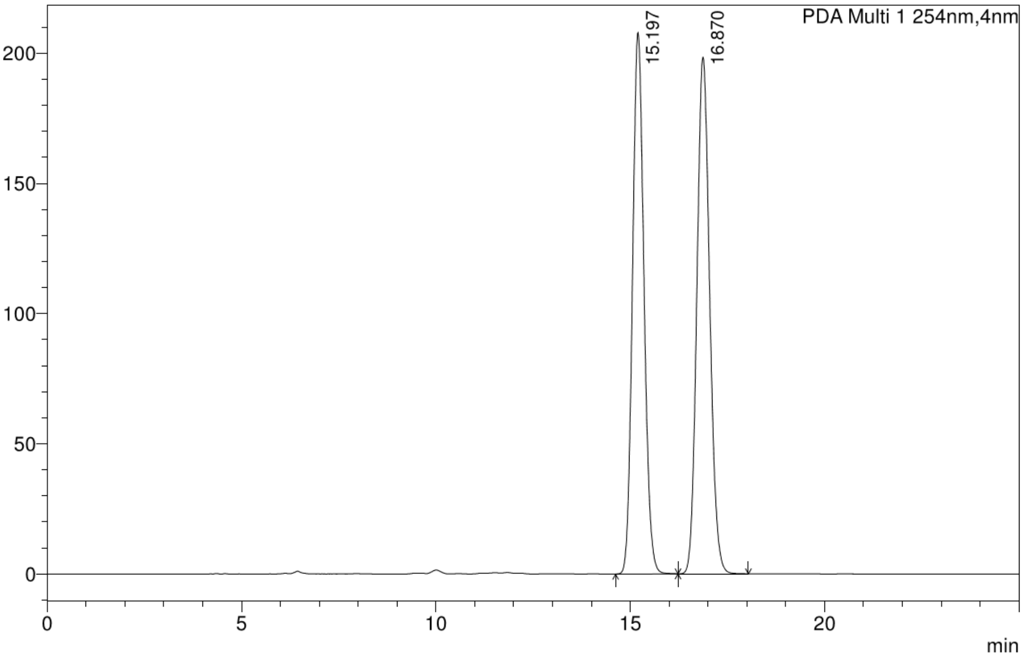

PDA Ch 1 254 nm

Peak#	Ret. Time	Area%
1	15.197	48.363
2	16.870	51.637
Total		100.00

HPLC-analysis: YMC Chiral Amylose-C - I.D. S-5 µm, LC Column (250 X 4.6 mm), *n*-hexane/*i*-PrOH = 85:15, flow rate = 0.7 mL/min, λ = 254 nm, t_{R1} (minor) = 15.1 min, t_{R2} (major) = 16.8 min. *ee* = 3%.

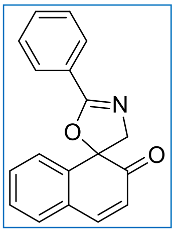

Racemate 20

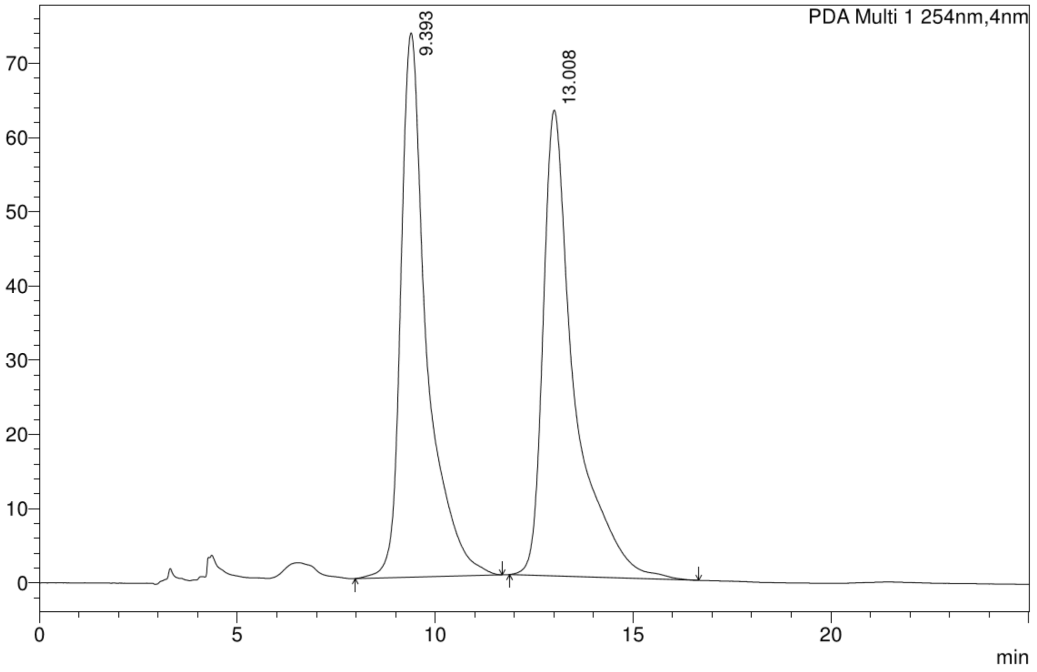

PDA Ch 1 254 nm

Peak#	Ret. Time	Area%
1	9.393	50.103
2	13.008	49.897
Total		100.00

HPLC-analysis: Chiralcel OD-H column - I.D. S-5 µm, LC Column (250 X 4.6 mm), *n*-hexane/*i*-PrOH = 80/20, flow rate = 1.0 mL/min, λ = 254 nm, t_{R1} = 9.3 min, t_{R2} = 13.0 min.

Catalyzed by (+)-**5f**

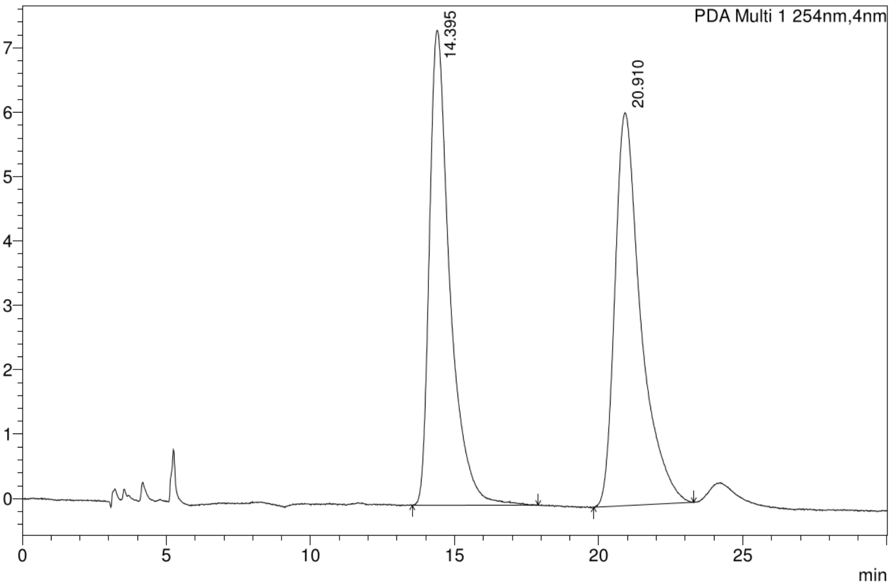

PDA Ch 1 254 nm

Peak#	Ret. Time	Area%
1	14.395	50.896
2	20.910	49.729
Total		100.00

HPLC-analysis: Chiralcel OD-H column - I.D. S-5 μm, LC Column (250 X 4.6 mm), *n*-hexane/*i*-PrOH = 88/12, flow rate = 1.0 mL/min, λ = 254 nm, t_{R1} (major) = 14.3 min, t_{R2} (minor) = 20.9 min. *ee* = 1%.

Catalyzed by (–)-**5f**

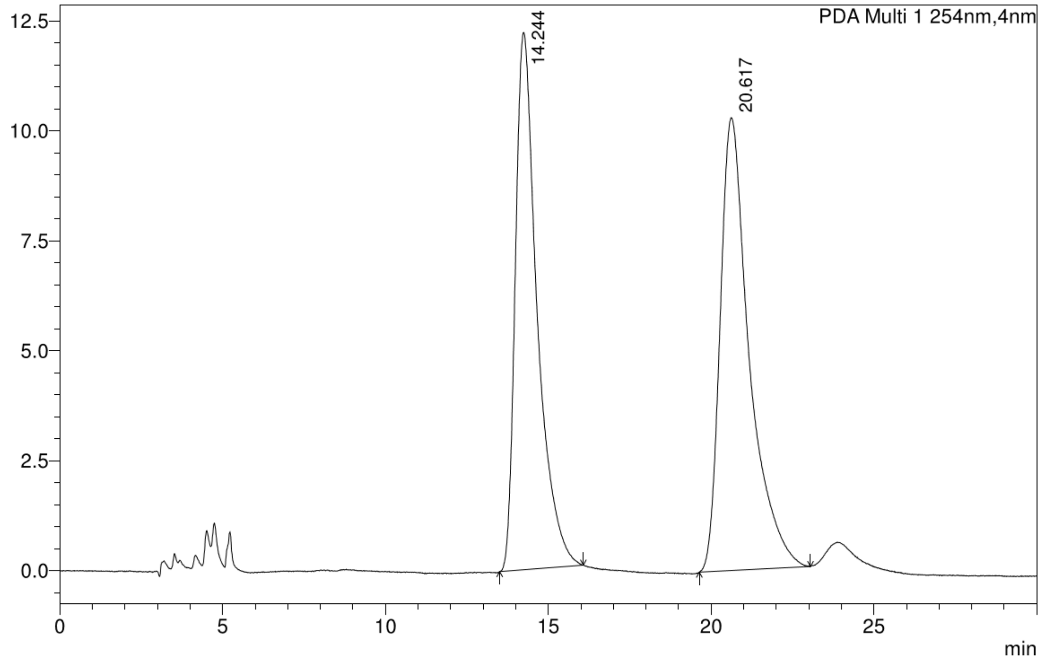

PDA Ch 1 254 nm

Peak#	Ret. Time	Area%
1	14.244	47.039
2	20.617	52.961
Total		100.00

HPLC-analysis: Chiralcel OD-H column - I.D. S-5 µm, LC Column (250 X 4.6 mm), *n*-hexane/*i*-PrOH = 88/12, flow rate = 1.0 mL/min, λ = 254 nm, t_{R1} (minor) = 14.2 min, t_{R2} (major) = 20.6 min. *ee* = 6%.

Catalyzed by (+)-**16**

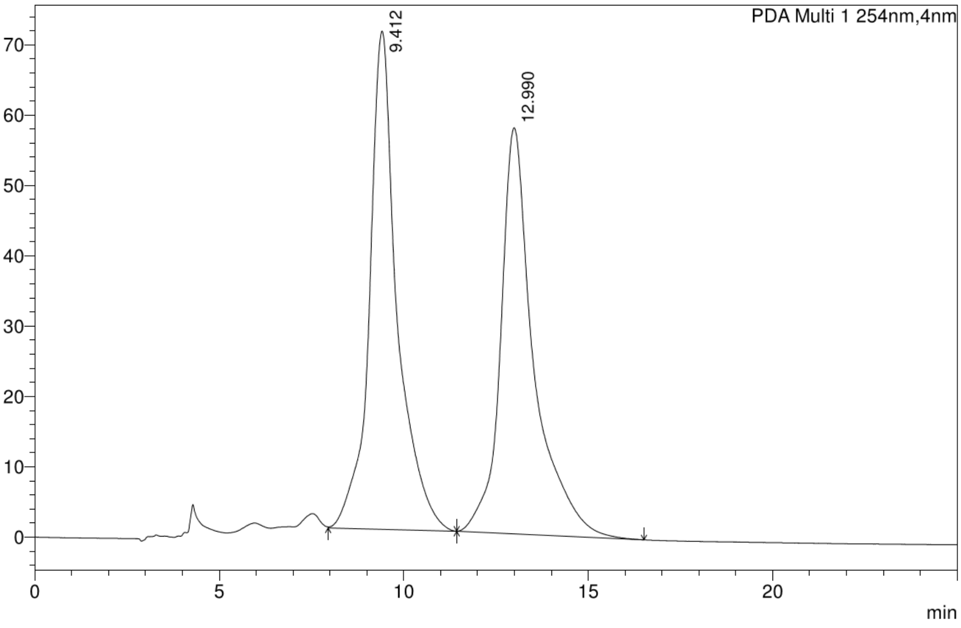

PDA Ch 1 254 nm

Peak#	Ret. Time	Area%
1	9.412	50.655
2	12.990	49.345
Total		100.00

HPLC-analysis: Chiralcel OD-H column - I.D. S-5 µm, LC Column (250 X 4.6 mm), *n*-hexane/*i*-PrOH = 80/20, flow rate = 1.0 mL/min, λ = 254 nm, t_{R1} (major) = 9.4 min, t_{R2} (minor) = 12.9 min. *ee* = 1%.

Catalyzed by (–)-**16**

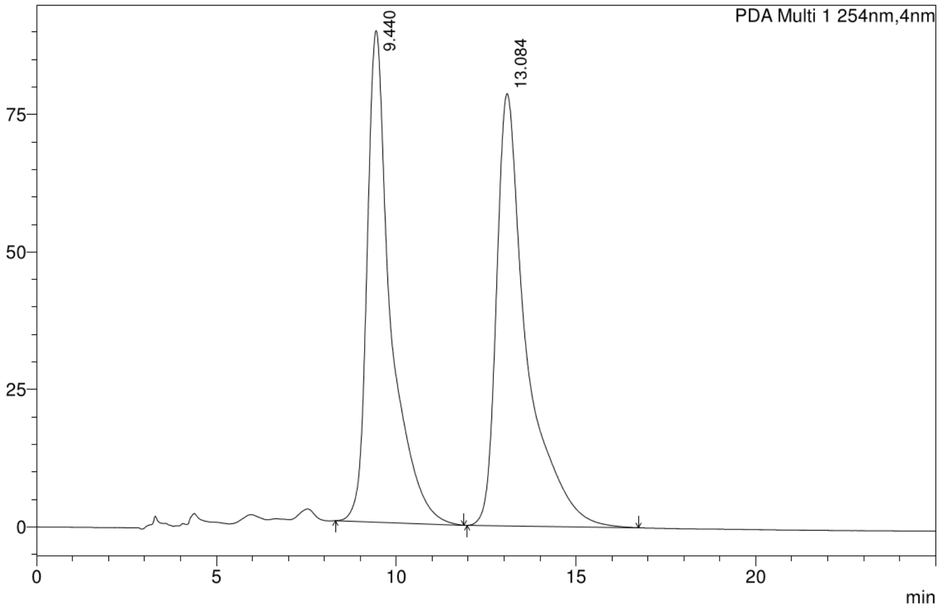

PDA Ch 1 254 nm

Peak#	Ret. Time	Area%
1	9.440	47.288
2	13.084	52.712
Total		100.00

HPLC-analysis: Chiralcel OD-H column - I.D. S-5 µm, LC Column (250 X 4.6 mm), *n*-hexane/*i*-PrOH = 80/20, flow rate = 1.0 mL/min, λ = 254 nm, t_{R1} (minor) = 9.4 min, t_{R2} (major) = 13.0 min. *ee* = 5%.

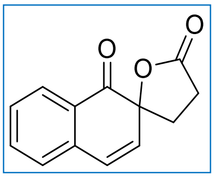

Racemate 22

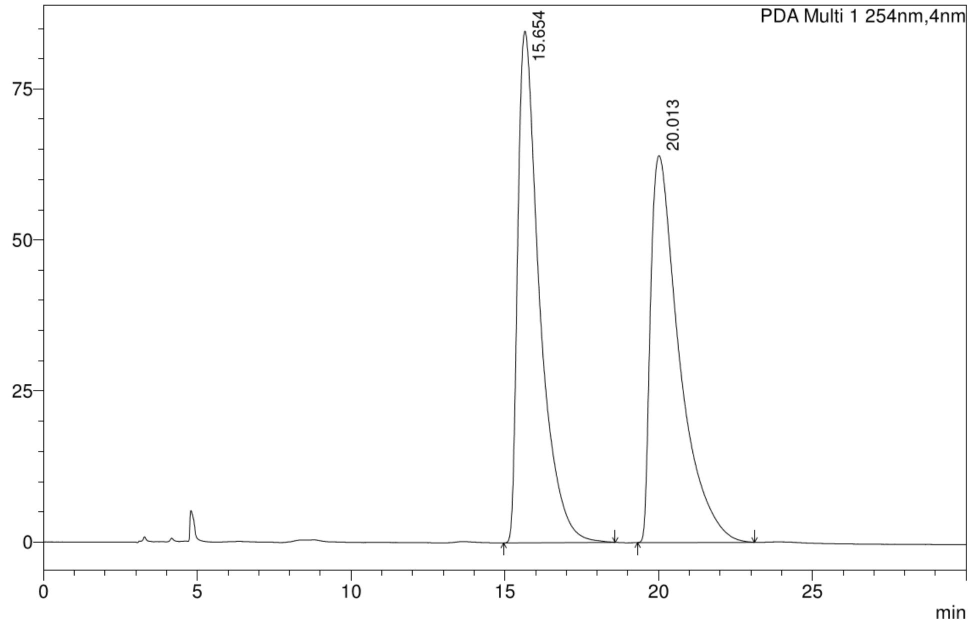

PDA Ch 1 254 nm

Peak#	Ret. Time	Area%
1	15.654	50.097
2	20.013	49.903
Total		100.00

HPLC-analysis: Chiralcel OD-H column - I.D. S-5 µm, LC Column (250 X 4.6 mm), *n*-hexane/*i*-PrOH = 85/15, flow rate = 1.0 mL/min, λ = 254 nm, t_{R1} = 15.6 min, t_{R2} = 20.0 min.

Catalyzed by (–)-**5f**

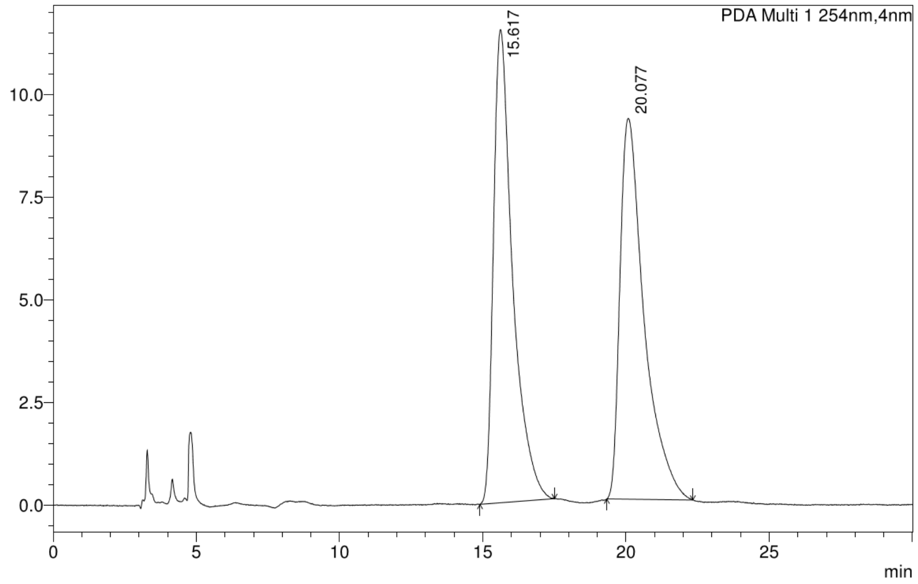

PDA Ch 1 254 nm

Peak#	Ret. Time	Area%
1	15.617	47.073
2	20.077	52.927
Total		100.00

HPLC-analysis: Chiralcel OD-H column - I.D. S-5 µm, LC Column (250 X 4.6 mm), *n*-hexane/*i*-PrOH = 85/15, flow rate = 1.0 mL/min, λ = 254 nm, t_{R1} (minor) = 15.6 min, t_{R2} (major) = 20.0 min. *ee* = 6%.

Catalyzed by (+)-**5f**

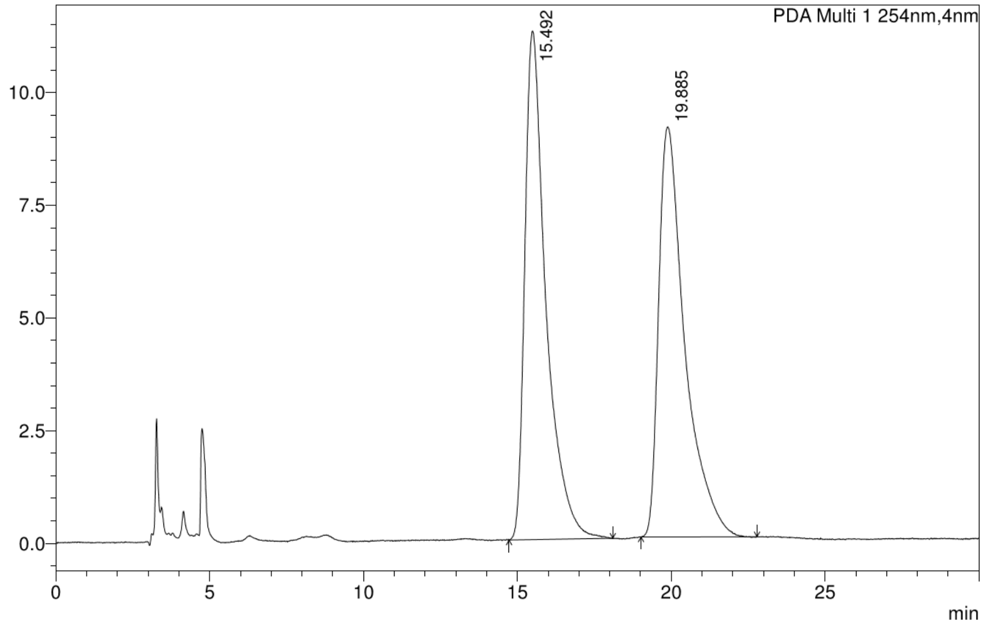

PDA Ch 1 254 nm

Peak#	Ret. Time	Area%
1	15.492	50.793
2	19.885	49.207
Total		100.00

HPLC-analysis: Chiralcel OD-H column - I.D. S-5 µm, LC Column (250 X 4.6 mm), *n*-hexane/*i*-PrOH = 85/15, flow rate = 1.0 mL/min, λ = 254 nm, t_{R1} (major) = 15.4 min, t_{R2} (minor) = 19.8 min. *ee* = 2%.

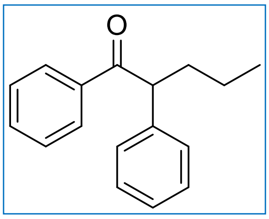

Racemate 24

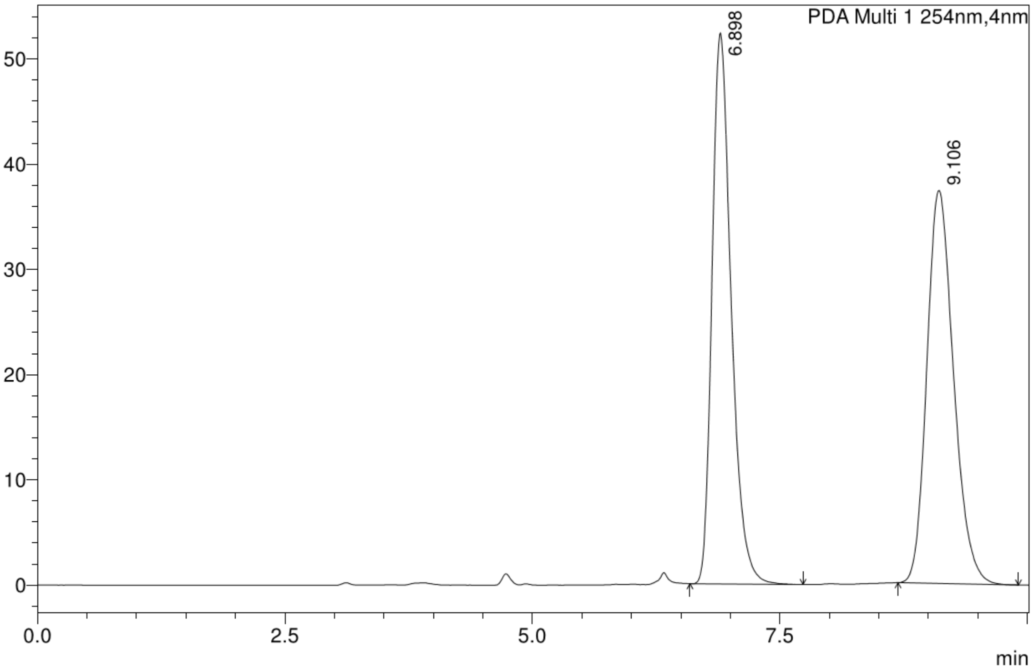

PDA Ch 1 254 nm

Peak#	Ret. Time	Area%
1	6.898	50.074
2	9.106	49.926
Total		100.00

HPLC-analysis: YMC Chiral Amylose-C - I.D. S-5 µm, LC Column (250 X 4.6 mm), *n*-hexane/*i*-PrOH = 99.5/0.5, flow rate = 1.0 mL/min, λ = 254 nm, t_{R1} = 6.8 min, t_{R2} = 9.1 min.

Catalyzed by (–)-**5f**

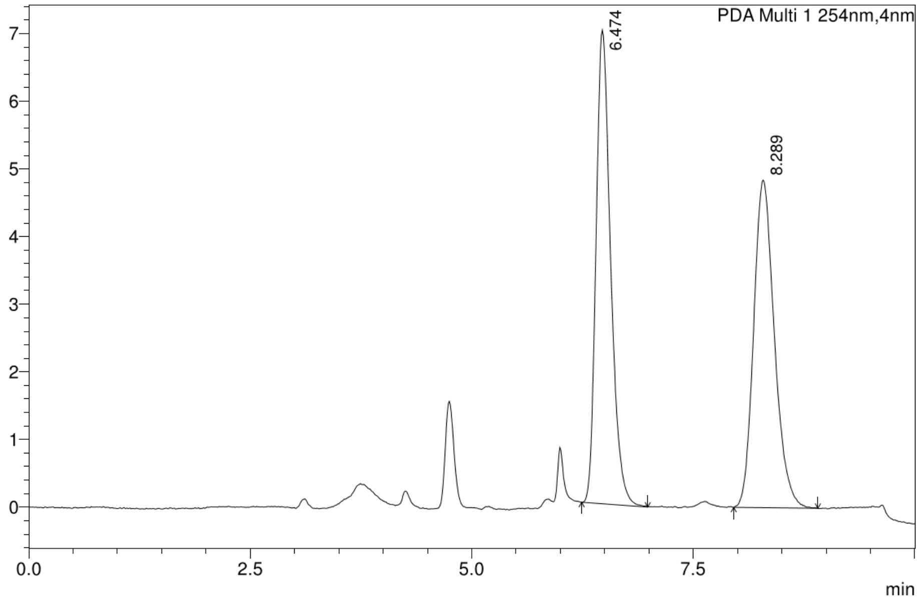

PDA Ch 1 254 nm

Peak#	Ret. Time	Area%
1	6.474	50.410
2	8.289	49.590
Total		100.00

HPLC-analysis: YMC Chiral Amylose-C - I.D. S-5 µm, LC Column (250 X 4.6 mm), *n*-hexane/*i*-PrOH = 99.5/0.5, flow rate = 1.0 mL/min, λ = 254 nm, t_{R1} (major) = 6.4 min, t_{R2} (minor) = 8.2 min. *ee* = 1%.

Catalyzed by (+)-**5f**

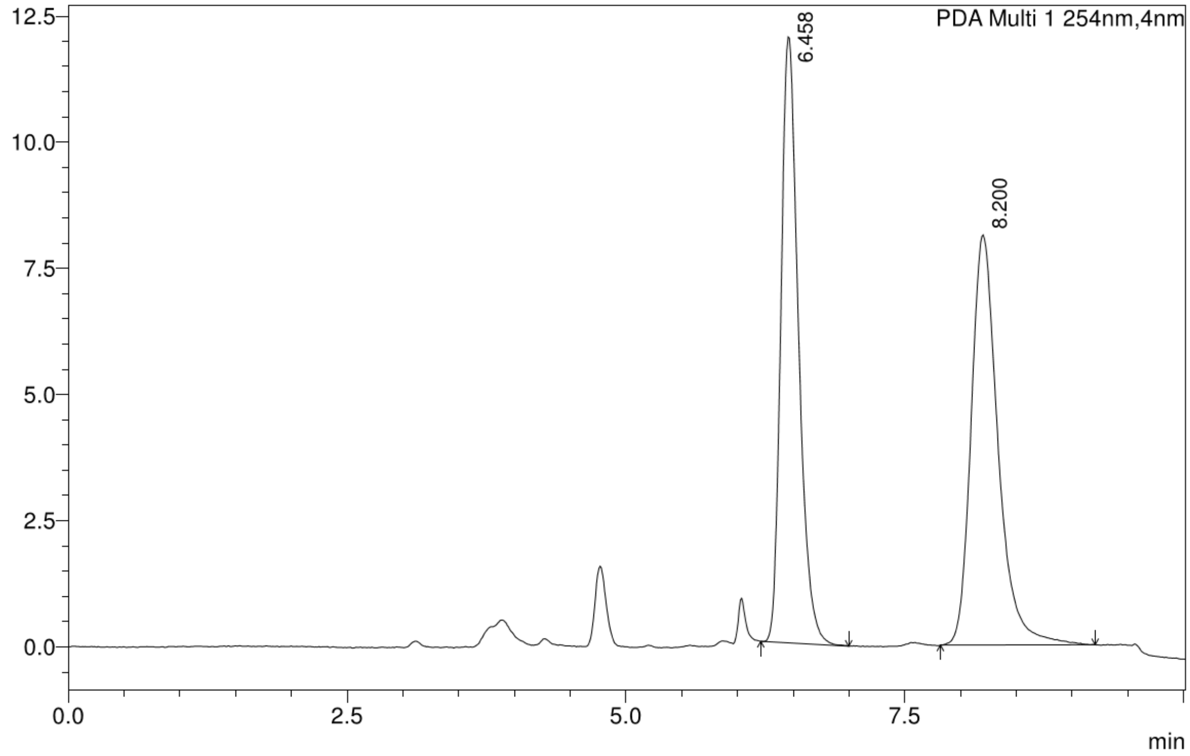

PDA Ch 1 254 nm

Peak#	Ret. Time	Area%
1	6.458	49.597
2	8.200	50.403
Total		100.00

HPLC-analysis: YMC Chiral Amylose-C - I.D. S-5 μm, LC Column (250 X 4.6 mm), n-hexane/*i*-PrOH = 99.5/0.5, flow rate = 1.0 mL/min, λ = 254 nm, t_{R1} = 6.4 (minor) min, t_{R2} (major) = 8.2 min. *ee* = 1%.

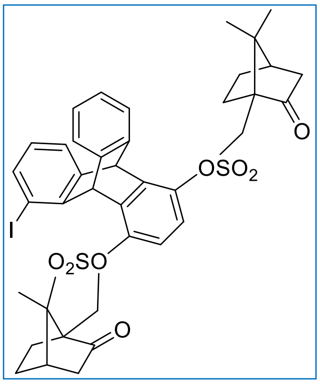

Racemate 16a

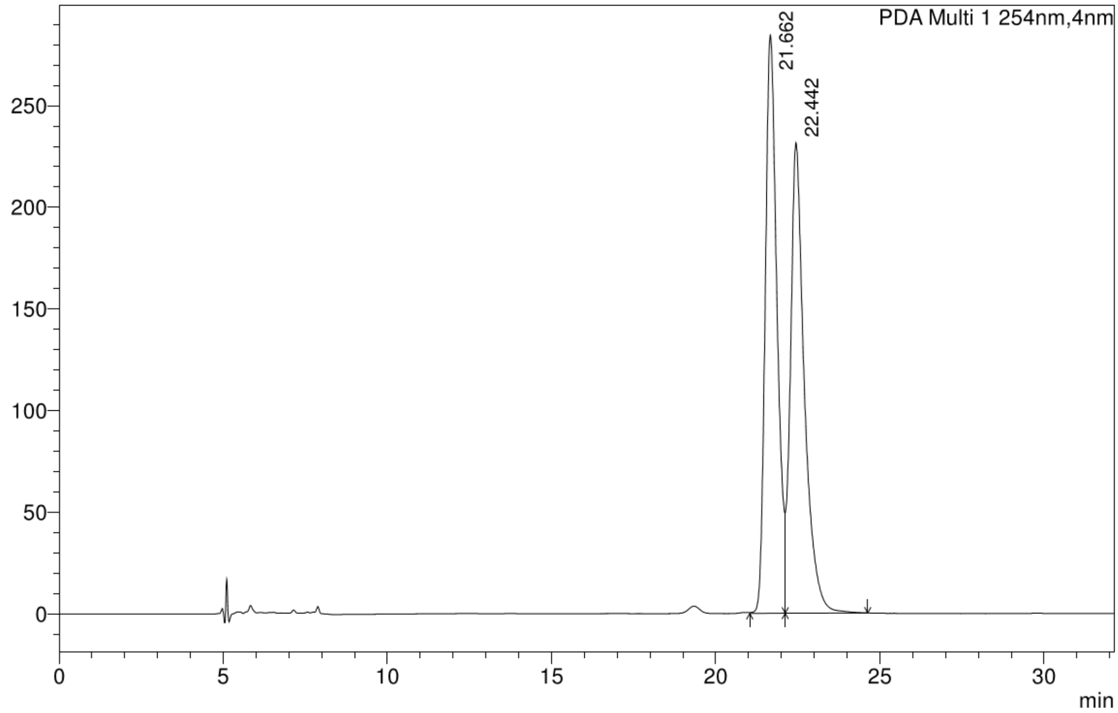

HPLC-analysis: Polaris – Si, I.D. S-5 µm, LC Column (250 X 4.6 mm), *n*-hexane/*i*-PrOH = 85 : 15, flow rate = 0.7 mL/min, λ = 254 nm, t_{R1} = 21.6 min, t_{R2} = 22.4 min.
